# Ninety-eight new species of *Trigonopterus* weevils from Sundaland and the Lesser Sunda Islands

**DOI:** 10.3897/zookeys.467.8206

**Published:** 2014-12-22

**Authors:** Alexander Riedel, Rene Tänzler, Michael Balke, Cahyo Rahmadi, Yayuk R. Suhardjono

**Affiliations:** 1 Museum of Natural History Karlsruhe, Erbprinzenstr. 13, D-76133 Karlsruhe, Germany; 2 SNSB-Zoological State Collection, Münchhausenstr. 21, D-81247 Munich, Germany; 3 GeoBio Center LMU Munich, Germany; 4 Zoological Museum, Cibinong Science Center - LIPI, Jl. Raya Jakarta- Bogor, Indonesia

**Keywords:** Southeast Asia, integrative taxonomy, turbo-taxonomy, weevils, hyperdiverse, morphology, *cox1*, DNA barcoding, conservation, endemism, Coleoptera, Curculionidae, Cryptorhynchinae

## Abstract

The genus *Trigonopterus* Fauvel, 1862 is highly diverse in Melanesia. Only one species, *Trigonopterus amphoralis* Marshall, 1925 was so far recorded West of Wallace’s Line (Eastern Sumatra). Based on focused field-work the fauna from Sundaland (Sumatra, Java, Bali, Palawan) and the Lesser Sunda Islands (Lombok, Sumbawa, Flores) is here revised. We redescribe *Trigonopterus amphoralis* Marshall and describe an additional 98 new species: *Trigonopterus acuminatus* **sp. n.**, *Trigonopterus aeneomicans* **sp. n.**, *Trigonopterus alaspurwensis* **sp. n.**, *Trigonopterus allopatricus* **sp. n.**, *Trigonopterus allotopus* **sp. n.**, *Trigonopterus angulicollis* **sp. n.**, *Trigonopterus argopurensis* **sp. n.**, *Trigonopterus arjunensis* **sp. n.**, *Trigonopterus asper* **sp. n.**, *Trigonopterus attenboroughi* **sp. n.**, *Trigonopterus baliensis* **sp. n.**, *Trigonopterus batukarensis* **sp. n.**, *Trigonopterus bawangensis* **sp. n.**, *Trigonopterus binodulus* **sp. n.**, *Trigonopterus bornensis* **sp. n.**, *Trigonopterus cahyoi* **sp. n.**, *Trigonopterus costipennis* **sp. n.**, *Trigonopterus cuprescens* **sp. n.**, *Trigonopterus cupreus* **sp. n.**, *Trigonopterus dacrycarpi* **sp. n.**, *Trigonopterus delapan* **sp. n.**, *Trigonopterus dentipes* **sp. n.**, *Trigonopterus diengensis* **sp. n.**, *Trigonopterus dimorphus* **sp. n.**, *Trigonopterus disruptus* **sp. n.**, *Trigonopterus dua* **sp. n.**, *Trigonopterus duabelas* **sp. n.**, *Trigonopterus echinatus* **sp. n.**, *Trigonopterus empat* **sp. n.**, *Trigonopterus enam* **sp. n.**, *Trigonopterus fissitarsis* **sp. n.**, *Trigonopterus florensis* **sp. n.**, *Trigonopterus foveatus* **sp. n.**, *Trigonopterus fulgidus* **sp. n.**, *Trigonopterus gedensis* **sp. n.**, *Trigonopterus halimunensis* **sp. n.**, *Trigonopterus honjensis* **sp. n.**, *Trigonopterus ijensis* **sp. n.**, *Trigonopterus javensis* **sp. n.**, *Trigonopterus kalimantanensis* **sp. n.**, *Trigonopterus kintamanensis* **sp. n.**, *Trigonopterus klatakanensis* **sp. n.**, *Trigonopterus lampungensis* **sp. n.**, *Trigonopterus latipes* **sp. n.**, *Trigonopterus lima* **sp. n.**, *Trigonopterus lombokensis* **sp. n.**, *Trigonopterus merubetirensis* **sp. n.**, *Trigonopterus mesehensis* **sp. n.**, *Trigonopterus micans* **sp. n.**, *Trigonopterus misellus* **sp. n.**, *Trigonopterus palawanensis* **sp. n.**, *Trigonopterus pangandaranensis* **sp. n.**, *Trigonopterus paraflorensis* **sp. n.**, *Trigonopterus pararugosus* **sp. n.**, *Trigonopterus parasumbawensis* **sp. n.**, *Trigonopterus pauxillus* **sp. n.**, *Trigonopterus payungensis* **sp. n.**, *Trigonopterus porcatus* **sp. n.**, *Trigonopterus pseudoflorensis* **sp. n.**, *Trigonopterus pseudosumbawensis* **sp. n.**, *Trigonopterus punctatoseriatus* **sp. n.**, *Trigonopterus ranakensis* **sp. n.**, *Trigonopterus relictus* **sp. n.**, *Trigonopterus rinjaniensis* **sp. n.**, *Trigonopterus roensis* **sp. n.**, *Trigonopterus rugosostriatus* **sp. n.**, *Trigonopterus rugosus* **sp. n.**, *Trigonopterus rutengensis* **sp. n.**, *Trigonopterus saltator* **sp. n.**, *Trigonopterus santubongensis* **sp. n.**, *Trigonopterus sasak* **sp. n.**, *Trigonopterus satu* **sp. n.**, *Trigonopterus schulzi* **sp. n.**, *Trigonopterus sebelas* **sp. n.**, *Trigonopterus sembilan* **sp. n.**, *Trigonopterus sepuluh* **sp. n.**, *Trigonopterus seriatus* **sp. n.**, *Trigonopterus serratifemur* **sp. n.**, *Trigonopterus setifer* **sp. n.**, *Trigonopterus silvestris* **sp. n.**, *Trigonopterus singkawangensis* **sp. n.**, *Trigonopterus singularis* **sp. n.**, *Trigonopterus sinuatus* **sp. n.**, *Trigonopterus squalidus* **sp. n.**, *Trigonopterus sumatrensis* **sp. n.**, *Trigonopterus sumbawensis* **sp. n.**, *Trigonopterus sundaicus* **sp. n.**, *Trigonopterus suturalis* **sp. n.**, *Trigonopterus syarbis* **sp. n.**, *Trigonopterus telagensis* **sp. n.**, *Trigonopterus tepalensis* **sp. n.**, *Trigonopterus tiga* **sp. n.**, *Trigonopterus trigonopterus* **sp. n.**, *Trigonopterus tujuh* **sp. n.**, *Trigonopterus ujungkulonensis* **sp. n.**, *Trigonopterus variolosus* **sp. n.**, *Trigonopterus vulcanicus* **sp. n.**, *Trigonopterus wallacei* **sp. n..** All new species are authored by the taxonomist-in-charge, Alexander Riedel. Most species belong to the litter fauna of primary wet evergreen forests. This habitat has become highly fragmented in the study area and many of its remnants harbor endemic species. Conservation measures should be intensified, especially in smaller and less famous sites to minimize the number of species threatened by extinction.

## Introduction

*Trigonopterus* Fauvel, a genus of flightless weevils placed in the subfamily Cryptorhynchinae of Curculionidae (Alonso-Zarazaga and Lyal 1999), is hyperdiverse in the Papuan region (Riedel et al. 2010; Tänzler et al. 2012). However, only a few species have been described from west of the Moluccas to date. Besides three species from the Philippines [*Trigonopterus bakeri* (Hustache, 1925), *Trigonopterus paucisquamosus* (Heller, 1915), *Trigonopterus semirubrus* (Hustache, 1925)] and one from Sulawesi [*Trigonopterus fulvicornis* (Pascoe, 1885)], there is only one more species described from East Sumatra [*Trigonopterus amphoralis* (Marshall)]. The conspicuous gap in distribution is here revealed as a sampling artifact and will be remedied with the present publication.

The area covered herein includes parts of Sundaland (i.e. Sumatra, Java, Borneo, and the Philippine island of Palawan) and the Lesser Sunda Islands. No species of *Trigonopterus* is known to us from the Malay Peninsula and the western and central parts of Sumatra. The intensive field work for this study largely focused on East Sumatra, Java and the Lesser Sunda Islands, while material available from only a few localities in Borneo and Palawan could be included. Thus, many more species can be expected from these islands. The fauna of Sulawesi will be treated in a separate publication.

We have previously established that *Trigonopterus* weevils are suitable for accelerated taxonomic study combining morphology and the DNA barcoding approach using mitochondrial *cox1* data (Riedel et al. 2013a, b; Tänzler et al. 2012); herein we will use the same strategy to provide “faces and names” to an additional 98 undescribed species. While in our earlier study (Riedel et al. 2013b) we tried to name only selected representatives covering all species groups, we now revise all species of a focal region. This newly established taxonomic foundation will be of immediate use to our studies on the evolution and biogeography of *Trigonopterus* (e.g. Tänzler et al. 2014).

## Materials and methods

This study is based on > 4,000 specimens of *Trigonopterus*, collected specifically for a project on this genus. Unless otherwise stated, specimens were collected by sifting the litter of primary forests with subsequent extraction by hand or, more efficiently, using eclectors (Besuchet et al. 1987). Holotypes were selected from the 703 sequenced specimens; their DNA had been extracted nondestructively as described by Riedel et al. (2010). The genitalia of most specimens did not require maceration after DNA-extraction; they could be directly stained with an alcoholic Chlorazol Black solution and stored in glycerol in microvials attached to the pin of the specimens. Genitalia of collection specimens or specimens whose abdominal muscle tissue was not sufficiently digested after DNA extraction were macerated with 10% KOH and rinsed in diluted acetic acid before staining. Illustrations of habitus and genitalia were prepared from holotypes. Finally, type series were supplemented with specimens stored in ethanol and older material from the dry collection. As always the case in paratypes, there is a chance that some of these are incorrectly assigned; this is especially true for specimens without sequence data as an identification based on external morphological characters is more prone to error than an identification based on a *cox1* sequence (Tänzler et al. 2012). Several different species belonging to the *Trigonopterus relictus*-group occur sympatrically in Sumbawa and Flores. Since it was not possible to separate these sibling species without the extraction of male genitalia and / or their sequence data, a substantial number of specimens were not included in any type series. Type depositories are cited using the following codens:

ARC Alexander Riedel Collection, stored in SMNK, Germany.

MZB LIPI Research Center of Biology, Division of Zoology, Museum Zoologicum Bogoriense, Widyasatwaloka, Cibinong, Indonesia.

SMNK Staatliches Museum für Naturkunde, Karlsruhe, Germany.

ZSM Zoologische Staatssammlung, München, Germany.

The methods applied for DNA sequencing and sequence analysis are described by Riedel et al. (2010) and Tänzler et al. (2012). Morphological descriptions are limited to major diagnostic characters as outlined by Riedel et al. (2013a, b). Negative character states (i.e. the absence of a character) are only mentioned explicitly where it appears appropriate. For example, there are some species with scale-bearing punctures in which the insertion of the scale has shifted from the center to the margin of the puncture. In these cases, the character is described, but for the majority of species where it is placed in the center it is not mentioned. Common practice would require stating explicitly “scale inserted in center of puncture”. Although formally accurate, in groups comprising hundreds of species this leads to inflated descriptions that distract the reader from the important information by enumerating the absence of rare character states.

Morphological terminology follows Beutel and Leschen (2005) and Leschen et al. (2009), i.e. the terms “mesoventrite” / “metaventrite” are used instead of “mesosternite” / “metasternite”, and “mesanepisternum” / “metanepisternum” instead of “mesepisternum” / “metepisternum”; “penis” is used instead of “aedeagus” as the tegmen is usually without useful characters in *Trigonopterus* and therefore omitted from species descriptions. Descriptions were prepared using a Leica MZ16 dissecting microscope and a fluorescent desk lamp for illumination. Measurements were taken with the help of an ocular grid. The length of the body was measured in dorsal aspect from the elytral apex to the front of the pronotum. Width of elytra was measured between the humeri at their greatest extent and across *both* elytra. Legs were described in an idealized laterally extended position; there is a dorsal / ventral and an anterior / posterior surface. Habitus illustrations were compiled using the Automontage© software (Syncroscopy, Cambridge, UK) with a JVC KY70 camera (JVC Professional Products) adapted to a Leica Z6 APO (Leica Microsystems, Wetzlar, Germany). Photographic illustrations of genitalia were made using the same software / camera combination adapted to a Zeiss Imager microscope, and for this purpose the genitalia were embedded in glycerol gelatin as described by Riedel (2005). Genitalia were photographed with their longitudinal axis somewhat lifted anteriorly, to adequately illustrate structures of the curved down apex. All photographs were enhanced using Adobe Photoshop CS2. However, care was taken not to obscure or alter any features of the specimens illustrated. Sequence data were submitted to the European Molecular Biology Laboratory (EMBL), and the accession numbers are provided under each species e.g. as “(EMBL # FN429236)”.

## Systematics

### Trigonopterus

Taxon classificationAnimaliaColeopteraCurculionidae

Fauvel, 1862

#### Type species.

*Trigonopterus insignis* Fauvel, 1862, by monotypy.

#### Diagnosis.

Fully apterous genus of Cryptorhynchinae. Length 1.5–6.0 mm. Rostrum in repose not reaching center of mesocoxa. Scutellar shield completely absent externally. Mesothoracic receptacle deep, posteriorly closed. Metanepisternum completely absent externally. Elytra with 9 striae (sometimes superficially effaced). Tarsal claws minute. Usually body largely unclothed, without dense vestiture. For additional information, see http://species-id.net/wiki/Trigonopterus.

### Descriptions of the species

#### 1. Trigonopterus acuminatus

Taxon classificationAnimaliaColeopteraCurculionidae

Riedel sp. n.

http://zoobank.org/25C0F9B8-EAEE-4BA4-AD2C-7D7DBE20E84D

##### Diagnostic description.

**Figure 1.**
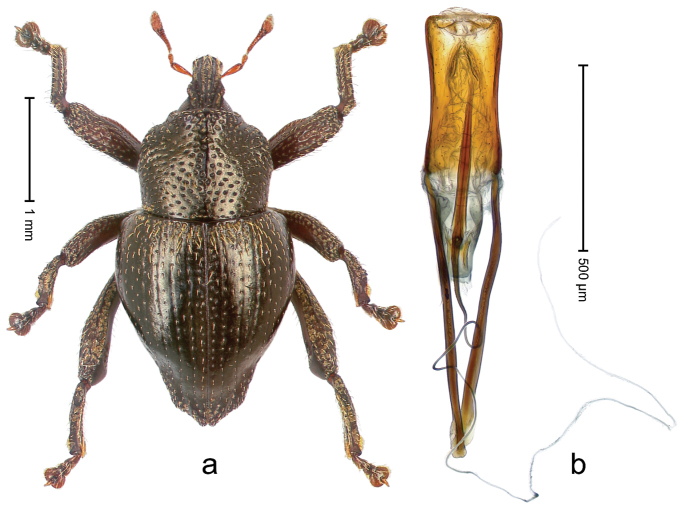
*Trigonopterus acuminatus* Riedel, sp. n., holotype; **a** Habitus **b** Penis.

Holotype, male (Fig. 1a). Length 3.14 mm. Color of legs and antennae ferruginous; remainder black. Body elongate; in dorsal aspect with marked constriction between pronotum and elytron; in profile dorsally convex. Rostrum with median and pair of submedian ridges; intervening furrows each with sparse row of erect piliform scales; epistome with indistinct, transverse, subangulate ridge. Pronotum with indistinct subapical constriction; disk densely punctate, interspaces microreticulate; with recumbent to erect piliform scales. Elytra with striae indistinct, marked by fine lines and rows of small punctures; intervals flat, weakly microreticulate, with rows of small punctures; with sparse, recumbent setae, bordering basal margin with long suberect piliform scales; interval 7 in apical quarter forming lateral edge; sutural interval forming pair of apical protrusions dorsal of truncate elytral apex concealed from above. Femora edentate; anteroventral ridge weakly crenulate. Metafemur subapically with stridulatory patch. Dorsal edge of metatibia in basal third denticulate; mesotibia with subbasal angulation extended as acute tooth. Abdominal ventrites 1–2 concave, subglabrous; ventrite 5 at middle with shallow pit. Penis (Fig. 1b) with sides of body weakly concave; apex rounded; transfer apparatus thick flagelliform, 2.0 × longer than body; apodemes 2.1 × as long as body; ductus ejaculatorius without bulbus. **Intraspecific variation.** Length 2.74–3.14 mm. Female rostrum with median and pair of submedian glabrous costae; epistome simple. Integument of females with microreticulation less distinct, piliform scales shorter and sparser. Female elytral apex slightly shorter, sutural intervals forming pair of tubercles. Female abdominal ventrites 1–2 flat, ventrite 5 flat.

##### Material examined.

Holotype (MZB): ARC2482 (EMBL # LM655819), East Java Prov., Kediri, Mt. Wilis, Besuki, sample 1, S07°51.785', E111°50.188', 1379 m, 21-XI-2011. Paratypes (MZB, SMNK, ZSM): E-Java Prov.: 17 exx, ARC2483 (EMBL # LM655820), ARC2484 (EMBL # LM655821), same data as holotype; 2 exx, Kediri, Besuki, Mt. Wilis, sample 4, S07°51.852', E111°50.185', 1388 m, 22-XI-2011; 4 exx, Kediri, Besuki, Mt. Wilis, sample 5, S07°51.650', E111°50.174', 1485 m, 22-XI-2011; 1 ex, Kediri, Besuki, Mt. Wilis, sample 6, S07°51.709', E111°50.100', 1409 m, 22-XI-2011.

##### Distribution.

E-Java Prov. (Mt. Wilis). Elevation: 1379–1485 m.

##### Etymology.

This epithet is based on the Latin adjective *acuminatus* (pointed) and refers to the shape of the elytra.

##### Notes.

*Trigonopterus acuminatus* Riedel, sp. n. was coded as “*Trigonopterus* sp. 357” by Tänzler et al. (2014).

#### 2. Trigonopterus aeneomicans

Taxon classificationAnimaliaColeopteraCurculionidae

Riedel sp. n.

http://zoobank.org/C8B886E4-5A12-4965-9B71-1405954B7D9A

##### Diagnostic description.

**Figure 2.**
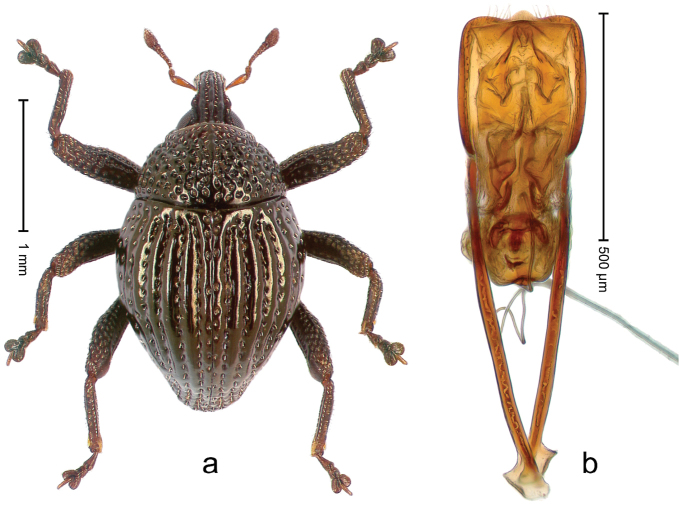
*Trigonopterus aeneomicans* Riedel, sp. n., holotype; **a** Habitus **b** Penis.

Holotype, male (Fig. 2a). Length 2.25 mm. Color of antennae ferruginous, legs dark ferruginous; remainder black, elytra with bronze lustre. Body subovate, in dorsal aspect and in profile with weak constriction between pronotum and elytron. Rostrum with median and pair of submedian ridges, intervening furrows each with sparse row of mesad directed setae; epistome with indistinct transverse, subangulate ridge. Pronotum coarsely punctate, laterally reticulate, submedially interspaces longitudinally rugose, with median costa; with sparse, suberect setae. Elytra with striae deeply impressed; each with sparse row of slender suberect scales; intervals costate, subglabrous; sutural interval with few coarse punctures. Femora with simple, crenate anteroventral ridge. Metafemur subapically with stridulatory patch. Metatibia apically with uncus, without premucro. Abdominal ventrite 5 coarsely punctate, with sparse subrecumbent setae, with indistinct median ridge. Penis (Fig. 2b) with sides of body subparallel; containing pair of sclerites; apex sparsely setose, with median, subtriangular extension; transfer apparatus symmetrical; apodemes 2.4 × as long as body; ductus ejaculatorius without bulbus, basally forming distinct loop, entering transfer apparatus from apically. **Intraspecific variation.** Length 1.90–2.24 mm. Color with bronze lustre more or less distinct; sutural interval ferruginous or black. Female rostrum in apical half dorsally subglabrous, punctate; epistome simple. Elytra with coarse punctures of sutural interval confined to base or passing middle.

##### Material examined.

Holotype (MZB): ARC1472 (EMBL # LM655570), West Nusa Tenggara Prov., Lombok, Gn. Rinjani, Tetebatu, Kokok Belimbing, Rinjani-track, sample 3, S08°28.837', E116°26.553', 1245 m, 02-IV-2010. Paratypes (MZB, SMNK, ZSM): West Nusa Tenggara Prov.: 9 exx, ARC1471 (EMBL # LM655569), same data as holotype; 19 exx, ARC0168, Tetebatu, Rinjani-trail, 1200-1450 m, 07-XII-2004; 6 exx, Tetebatu, Rinjani-trail from Orong Gerisak, sample 5, S08°30.096', E116°25.062', 1010 m, 04-IV-2010; 19 exx, ARC1462 (EMBL # LM655560), ARC1463 (EMBL # LM655561), Lombok, Gn. Rinjani, Tetebatu, Orong Gerisak, Rinjani-track, sample 6, S08°29.577', E116°24.782', 1195 m, 04-IV-2010; 10 exx, Lombok, Gn. Rinjani, Tetebatu, Orong Gerisak, Rinjani-track, sample 7, S08°29.433', E116°24.746', 1240 m, 04-IV-2010; 18 exx, ARC1479 (EMBL # LM655577), ARC1480 (EMBL # LM655578), Lombok, Gn. Rinjani, Tetebatu, Orong Gerisak, Rinjani-track, sample 8, S08°29.173', E116°24.517', 1345 m, 04-IV-2010; 3 exx, Lombok, Gn. Rinjani, Tetebatu, Orong Gerisak, Rinjani-track, sample 9, S08°28.981', E116°24.353', 1430 m, 04-IV-2010; 3 exx, ARC2259 (EMBL # LM655713), Lombok, Senaru, Rinjani-track, sample 1, S08°19.429', E116°24.082', 900 m, 21-III-2011; 30 exx, ARC2261 (EMBL # LM655715), ARC2262 (EMBL # LM655716), Lombok, Senaru, Rinjani-track, sample 2, S08°20.570', E116°23.969', 1320 m, 21-III-2011; 10 exx, Lombok, Senaru, Rinjani-track, sample 3, S08°20.780', E116°23.790', 1465 m, 21-III-2011; 8 exx, Lombok, Senaru, Rinjani-track, sample 4, S08°20.439', E116°24.047', 1240 m, 21-III-2011; 13 exx, Lombok, Senaru, Rinjani-track, sample 5, S08°19.800', E116°24.107', 1015 m, 21-III-2011; 7 exx, Lombok, Senaru, Rinjani-track, sample 6, S08°19.359', E116°24.070', 860 m, 23-III-2011; 12 exx, Senaru, Rinjani-trail, sample 7, S08°19.643', E116°24.033', 935 m, 23-III-2011; 14 exx, Senaru, Rinjani-trail, sample 8, S08°19.719', E116°24.040', 955 m, 23-III-2011; 6 exx, ARC2277 (EMBL # LM655731), ARC2278 (EMBL # LM655732), Santong, Rinjani-track, sample 1, S08°20.715', E116°19.695', 830 m, 24-III-2011; 8 exx, Santong, Rinjani-track, sample 2, S08°20.813', E116°19.778', 870 m, 24-III-2011; 4 exx, Santong, Rinjani-track, sample 3, S08°20.996', E116°20.001', 960 m, 24-III-2011; 10 exx, Santong, Rinjani-track, sample 4, S08°21.160', E116°20.067', 1005 m, 25-III-2011; 2 exx, Santong, Rinjani-track, sample 5, S08°21.351', E116°20.208', 1105 m, 25-III-2011; 6 exx, Santong, Rinjani-track, sample 6, S08°21.754', E116°20.476', 1315 m, 25-III-2011; 17 exx, ARC2293 (EMBL # LM655747), ARC2294 (EMBL # LM655748), Gn. Rinjani, Bawnau, near Sembalun, sample 1, S08°21.136', E116°29.257', 1140 m, 26-III-2011; 3 exx, Mt. Rinjani, Bawnau, near Sembalun, S08°21.136', E116°29.257', 1140 m, collected from foliage, 26-III-2011; 44 exx, ARC1522 (EMBL # LM655620), ARC1523 (EMBL # LM655621), ARC1524 (EMBL # LM655622), Sumbawa, Batu Dulang, Mt. Batu Pasak, sample 2, S08°37.028', E117°15.783', 1305 m, 12-IV-2010; 5 exx, Sumbawa, Batu Dulang, Mt. Batu Pasak, sample 3, S08°37.524', E117°15.423', 1385 m, 12-IV-2010; 12 exx, Sumbawa, Batu Dulang, Mt. Batu Pasak, sample 3, S08°37.524', E117°15.423', 1385 m, 18-IV-2010; 14 exx, Sumbawa, Batu Dulang, Mt. Batu Pasak, sample 4, S08°37.318', E117°15.339', 1280 m, 18-IV-2010; 11 exx, Sumbawa, Batu Dulang, Mt. Batu Pasak, sample 5, S08°37.005', E117°15.790', 1350 m, 18-IV-2010; 23 exx, ARC1502 (EMBL # LM655600), ARC1503 (EMBL # LM655601), ARC1504 (EMBL # LM655602), West Nusa Tenggara Prov., Sumbawa, Tepal, Pc. Nengas, sample 2, S08°35.884', E117°08.384', 1310 m, 15-IV-2010; 7 exx, West Nusa Tenggara Prov., Sumbawa, Tepal, Pc. Nengas, sample 3, S08°35.386', E117°08.251', 1415 m, 15-IV-2010; 1 ex, West Nusa Tenggara Prov., Sumbawa, Tepal, Pc. Nengas, sample 4, S08°35.168', E117°08.175', 1515 m, 15-IV-2010; 8 exx, West Nusa Tenggara Prov., Sumbawa, Tepal, Pc. Nengas, sample 5, S08°35.740', E117°08.721', 1330 m, 16-IV-2010; 17 exx, West Nusa Tenggara Prov., Sumbawa, Tepal, Pc. Nengas, sample 6, S08°35.533', E117°08.605', 1350 m, 16-IV-2010; 30 exx, West Nusa Tenggara Prov., Sumbawa, Tepal, Pc. Nengas, sample 7, S08°35.176', E117°08.295', 1490 m, 16-IV-2010.

##### Distribution.

West Nusa Tenggara Prov., Lombok (Santong, Sembalun, Senaru, Tetebatu), Sumbawa (Batu Dulang, Tepal). Elevation: 830–1350 m.

##### Etymology.

This epithet is a combination of the Latin adjectives *aeneus* (copper, bronze) and *micans* (shining) and refers to the metallic lustre of this species´ elytra.

##### Notes.

*Trigonopterus aeneomicans* Riedel, sp. n. was coded as “*Trigonopterus* sp. 288” by Tänzler et al. (2014).

#### 3. Trigonopterus alaspurwensis

Taxon classificationAnimaliaColeopteraCurculionidae

Riedel sp. n.

http://zoobank.org/3A02E4CA-376A-4332-9DD8-CC2856C2EE95

##### Diagnostic description.

**Figure 3.**
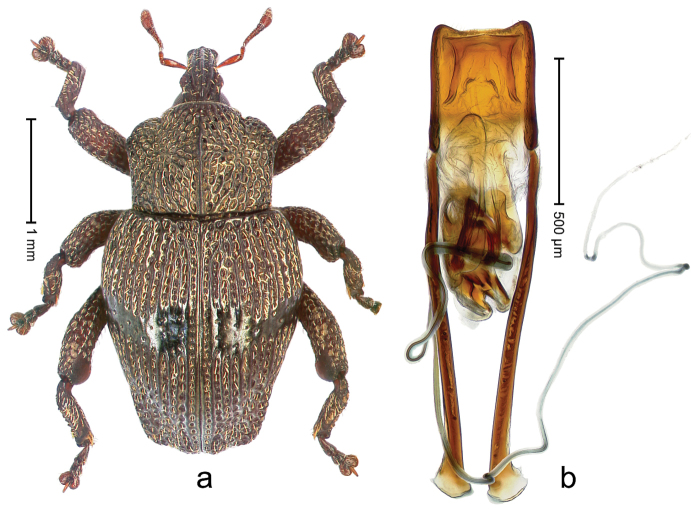
*Trigonopterus alaspurwensis* Riedel, sp. n., holotype; **a** Habitus **b** Penis.

Holotype, male (Fig. 3a). Length 3.47 mm. Color of legs and head ferruginous, transverse band of elytra polished black, remainder with greenish coppery lustre. Body elongate; in dorsal aspect with marked constriction between pronotum and elytron; in profile dorsally flat. Rostrum with median and pair of submedian ridges; intervening furrows each with row of punctures and suberect piliform scales; epistome simple. Pronotum anterolaterally markedly projecting, rounded; with subapical constriction; disk densely punctate; punctures partly elongate and / or arranged in confluent rows forming rhombus-like pattern near midline; each puncture containing piliform white scale; medially with glabrous costa. Elytral intervals bearing each one row of course punctures flanked by glabrous ridges; striae hard to distinguish from secondary rows of punctures; punctures containing long, recumbent, cream-colored scales; at middle with transverse subglabrous band; interval 7 swollen subapically, forming lateral edge; apex subtruncate, sutural interval not protruding. Femora edentate; anteroventral ridge indistinct. Metafemur subapically with stridulatory patch. Dorsal edge of tibiae subbasally with angulation extended as acute tooth. Abdominal ventrite 5 with shallow concave pit, basally and laterally with long suberect scales. Penis (Fig. 3b) with body short, sides subparallel; apex broadly rounded; transfer apparatus complex, rotated to the left; apodemes 3.0 × as long as body; ductus ejaculatorius without bulbus. **Intraspecific variation.** Length 2.97–3.47 mm. Females more slender than males. Female rostrum with median and pair of submedian glabrous costae. Female elytra with lateral contour convex, widest near middle; male elytra widest between humeri; elytral sculpture of females less distinct, longitudinal ridges weak or absent, transverse band near middle indistinct, sparsely punctate. Female abdominal ventrite 5 flat.

##### Material examined.

Holotype (MZB): ARC2326 (EMBL # LM655779), East Java Prov., Banyuwangi Reg., Alas Purwo N.P., Pancur, sample 3, S08°41.533', E114°22.593', 25 m, 09-IV-2011. Paratypes (MZB, SMNK): E-Java Prov., Banyuwangi Reg., Alas Purwo N.P.: 6 exx, ARC2323 (EMBL # LM655776), ARC2324 (EMBL # LM655777), ARC2325 (EMBL # LM655778), Triangulasi, sample 1, S08°39.744', E114°21.976', 25 m, 09-IV-2011; 1 ex, ARC2327 (EMBL # LM655780) same data as holotype; 1 ex, Triangulasi, sample 4, S08°39.335', E114°21.772', 25 m, 10-IV-2011; 3 exx, Triangulasi, sample 5, S08°39.426', E114°21.890', 25 m, 10-IV-2011; 1 ex, Triangulasi, sample 7, S08°39.373', E114°21.842', 25 m, 10-IV-2011; 1 ex, Triangulasi, sample 8, S08°39.559', E114°21.823', 25 m, 10-IV-2011.

##### Distribution.

E-Java Prov. (Alas Purwo N.P.). Elevation: 25 m.

##### Etymology.

This epithet is based on the type locality, Alas Purwo National Park.

##### Notes.

*Trigonopterus alaspurwensis* Riedel, sp. n. was coded as “*Trigonopterus* sp. 345” by Tänzler et al. (2014).

#### 4. Trigonopterus allopatricus

Taxon classificationAnimaliaColeopteraCurculionidae

Riedel sp. n.

http://zoobank.org/CCBB24A3-B23C-46DE-AA97-4BF40D281BB7

##### Diagnostic description.

**Figure 4.**
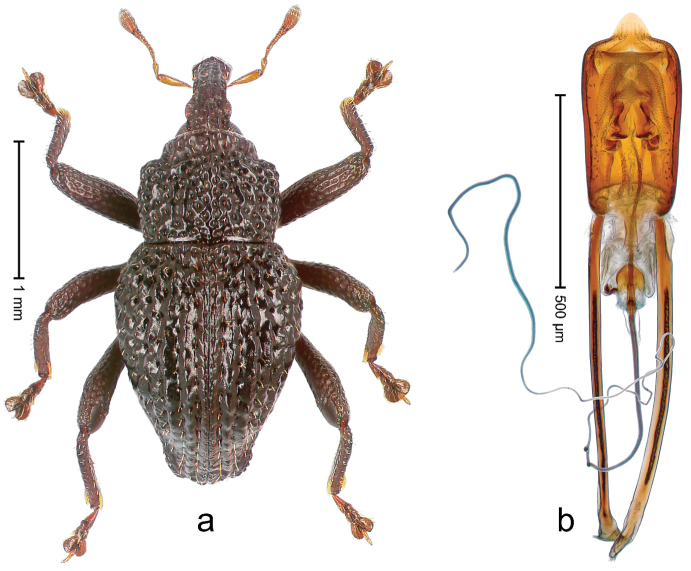
*Trigonopterus allopatricus* Riedel, sp. n., holotype; **a** Habitus **b** Penis.

Holotype, male (Fig. 4a). Length 2.60 mm. Color of legs and head ferruginous, remainder black. Body elongate; in dorsal aspect with marked constriction between pronotum and elytron; with distinct constriction in profile. Rostrum coarsely rugose-punctate, with median ridge and pair of irregular submedian ridges; epistome with indistinct, transverse, subangulate ridge. Pronotum anterolaterally angularly projecting; with distinct subapical constriction; disk scabrous, punctures each with one erect seta; interspaces microreticulate; with median ridge. Elytra with striae deeply impressed, each with row of suberect bristles; intervals irregularly costate, almost tuberculate, nude, weakly microreticulate; interval 7 swollen subapically, laterally weakly projecting. Femora edentate. Metafemur subapically with stridulatory patch and transverse ridge. Abdominal ventrite 5 flat, densely punctate, setose. Penis (Fig. 4b) in profile markedly curved; in dorsal aspect body slender, sides subparallel; apex with median, subtriangular extension; transfer apparatus flagelliform, 1.2 × as long as body; apodemes 2.2 × as long as body; ductus ejaculatorius without bulbus. **Intraspecific variation.** Length 2.23–2.75 mm. Color of sutural interval rarely ferruginous, usually black. Female rostrum dorsally with pairs of lateral and submedian furrows; epistome simple. Elytral apex with interval 7 and sutural interval more or less distinctly projecting. Penis with flagelliform transfer apparatus 1.2–1.5 × longer than body.

##### Material examined.

Holotype (MZB): ARC2680 (EMBL # LM655938), West Java Prov., Sumedang, Sukajadi, Mt. Cakrabuana, sample 6, S07°01.943', E108°08.029', 1593 m, 20-IV-2012. Paratypes (MZB, SMNK, ZSM): W-Java Prov.: 2 exx, ARC3583 (EMBL # LM656016), ARC3584 (EMBL # LM656017), Ciwidey, Mt. Tikukur, sample 2, S07°07.816', E107°24.298', 1784 m, 01-XII-2011; 4 exx, ARC2512 (EMBL # LM655849), ARC2513 (EMBL # LM655850), Ciwidey, Mt. Tikukur, sample 3, S07°07.723', E107°23.898', 1929 m, 01-XII-2011; 2 exx, ARC2516 (EMBL # LM655853), Bandung, Lembang, Pangli, Mt. Bukittinggul, sample 1, S06°48.810', E107°44.216', 1753 m, 03-XII-2011; 2 exx, ARC2517 (EMBL # LM655854), ARC3585 (EMBL # LM656018), Bandung, Lembang, Pangli, Mt. Bukittinggul, sample 2, S06°48.845', E107°44.074', 1898 m, 03-XII-2011; 3 exx, ARC2724 (EMBL # LM655980), Garut, Wanaraja, Talagabodas, sample 1, S07°12.511', E108°03.554', 1741 m, 27-IV-2012; 7 exx, ARC2717 (EMBL # LM655973), ARC2718 (EMBL # LM655974), ARC2719 (EMBL # LM655975), ARC3595 (EMBL # LM656026), ARC3596 (EMBL # LM656027), ARC3597, Garut, Wanaraja, Talagabodas, sample 2, S07°11.966', E108°03.984', 1719 m, 27-IV-2012; 8 exx, ARC2715 (EMBL # LM655971), ARC2716 (EMBL # LM655972), Garut, Cikajang, Mt. Payung, sample 3, S07°25.320', E107°48.259', 1560 m, 26-IV-2012; 3 exx, ARC0186 (EMBL # LM655411), ARC0158, Garut, Cilawu, Mt. Cikuray, sample 2, S07°18.840', E107°52.512', 1800 m, 24-IX-2005; 4 exx, Garut, Cilawu, Mt. Cikuray, sample 4, S07°18.927', E107°52.460', 1930 m, 24-IX-2005; 2 exx, Garut, Cilawu, Mt. Cikuray, sample 3, S07°19.075', E107°52.338', 2050 m, 24-IX-2005; 13 exx, ARC2679 (EMBL # LM655937), same data as holotype; 4 exx, Mt. Sawal, Panjalu, Tembong, sample 3, S07°11.102', E108°16.310', 1569 m, 29-XI-2011; 9 exx, ARC2509 (EMBL # LM655846), ARC2510 (EMBL # LM655847), ARC2511 (EMBL # LM655848), Mt. Sawal, Panjalu, Tembong, sample 4, S07°11.275', E108°16.280', 1723 m, 29-XI-2011.

##### Distribution.

W-Java Prov. (Mt. Bukittinggul, Mt. Cakrabuana, Mt. Cikuray, Mt. Payung, Mt. Sawal, Talagabodas, Mt. Tikukur). Elevation: 1560–2050 m.

##### Etymology.

This epithet is based on the Greek *allos* (other) and the Latin *patria* (homeland) and refers to its fragmented distribution.

##### Notes.

*Trigonopterus allopatricus* Riedel, sp. n. was coded as “*Trigonopterus* sp. 305”.

#### 5. Trigonopterus allotopus

Taxon classificationAnimaliaColeopteraCurculionidae

Riedel sp. n.

http://zoobank.org/811F570F-F8C2-49D6-9139-B6D492ECFBB3

##### Diagnostic description.

**Figure 5.**
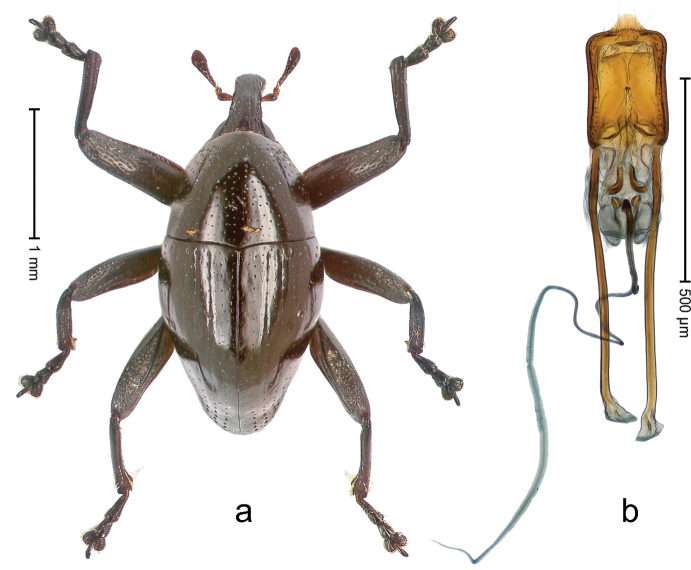
*Trigonopterus allotopus* Riedel, sp. n., holotype; **a** Habitus **b** Penis.

Holotype, male (Fig. 5a). Length 2.40 mm. Color black; antennae ferruginous. Body ovate, almost without constriction between pronotum and elytron; in profile evenly convex. Rostrum punctate, weakly rugose. Eyes with dorsal margin bordered by furrow, continuous with forehead, not carinate. Pronotum subglabrous, sparsely punctate. Elytra subglabrous, striae hardly visible but marked by few deeper punctures along basal margin and near apex. Femora microreticulate, punctate. Metafemur with posteroventral edge rimmed by costa; posterior surface with longitudinal impression; with smooth dorsoposterior edge; subapically without stridulatory patch. Mesotibia basally rounded; subapically with uncus and larger premucro. Metatibia subapically with fringe of curved, white setae; with uncus, without premucro. Abdominal ventrite 2 swollen, with posterior edge projecting, medially forming common cavity with ventrite 1; ventrite 5 dull, microreticulate, punctate, with shallow subapical impression. Penis (Fig. 5b). Apex symmetrical, with median triangular extension; transfer apparatus dentiform, apically bordered by pair of L-shaped sclerites; ductus ejaculatorius with indistinct bulbus.

##### Material examined.

Holotype (MZB): ARC1513 (EMBL # LM655611), West Nusa Tenggara Prov., Sumbawa, Batu Dulang, Mt. Batu Pasak, sample 2, S08°37.028', E117°15.783', 1305 m, 12-IV-2010.

##### Distribution.

West Nusa Tenggara Prov., Sumbawa (Batu Dulang). Elevation: 1305 m.

##### Etymology.

This epithet is the latinized form of a combination of the Greek adjective *allos* (foreign) and the noun *topos* (place). The species marks the Southwestern limit of the *Trigonopterus politus*-group´s distribution which is most diverse in the Papuan region.

##### Notes.

*Trigonopterus allotopus* Riedel, sp. n. was coded as “*Trigonopterus* sp. 331” by Tänzler et al. (2014). The holotype was taken from a sample of sifted leaf litter; however, based on observations of the other species of the *Trigonopterus politus*-group its occurrence on foliage appears more likely.

#### 6. Trigonopterus angulicollis

Taxon classificationAnimaliaColeopteraCurculionidae

Riedel sp. n.

http://zoobank.org/9361D093-B660-4282-AA14-D75CBBC8D67B

##### Diagnostic description.

**Figure 6.**
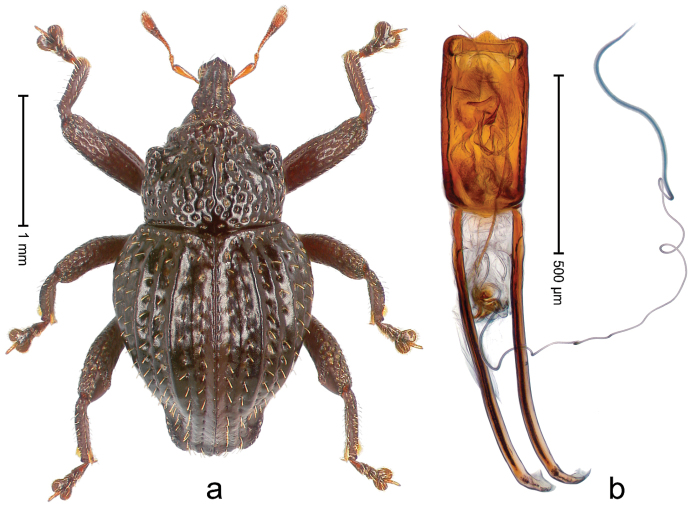
*Trigonopterus angulicollis* Riedel, sp. n., holotype; **a** Habitus **b** Penis.

Holotype, male (Fig. 6a). Length 2.58 mm. Color of head and legs ferruginous, remainder black. Body subhexagonal; in dorsal aspect with marked constriction between pronotum and elytron; with distinct constriction in profile. Rostrum with median ridge and pair of submedian ridges, intervening furrows punctate; punctures each with long erect scale; epistome with transverse, angulate ridge forming median denticle. Pronotum anterolaterally angularly projecting; with distinct subapical constriction; disk rugose-punctate, punctures each with slender suberect to subrecumbent scale; with median ridge. Elytra with striae deeply impressed, each with row of stout suberect bristles; intervals costate to carinate, almost nude; interval 3 most prominent; interval 7 swollen subapically, laterally projecting. Femora edentate. Metafemur subapically with stridulatory patch and transverse rows of denticles. Abdominal ventrite 5 swollen, at middle with shallow impression, sparsely setose. Penis (Fig. 6b) with sides of body subparallel, apex medially pointed; endophallic sclerites asymmetrical; transfer apparatus flagelliform, ca. 2 × as long as body; apodemes 2.0 × as long as body; ductus ejaculatorius without bulbus. **Intraspecific variation.** Length 2.45–2.58 mm. Female rostrum dorsally with pairs of lateral and submedian furrows; epistome simple. Female abdominal ventrite 5 flat, with median tooth markedly projecting from profile.

##### Material examined.

Holotype (MZB): ARC2654 (EMBL # LM655912), West Java Prov., Sukabumi, Mt. Gede, Cisaat, Situ Gunung, sample 1, S06°49.377', E106°55.729', 1281 m, 15-IV-2012. Paratypes (MZB, SMNK): W-Java Prov., Sukabumi, Mt. Gede, Cisaat, Situ Gunung: 3 exx ARC2655 (EMBL # LM655913), same data as holotype; 2 exx, ARC2658 (EMBL # LM655916), ARC2659 (EMBL # LM655917), sample 2, S06°49.038', E106°55.852', 1342 m, 15-IV-2012.

##### Distribution.

W-Java Prov. (Mt. Gede). Elevation: 1281–1342 m.

##### Etymology.

This epithet is a combination of the Latin adjective *angularis* (angular) and the noun *collum* (neck, pronotum).

##### Notes.

*Trigonopterus angulicollis* Riedel, sp. n. was coded as “*Trigonopterus* sp. 314” by Tänzler et al. (2014).

#### 7. Trigonopterus amphoralis

Taxon classificationAnimaliaColeopteraCurculionidae

Marshall

Trigonopterus amphoralis Marshall (1925): p. 217–218.

##### Diagnostic description.

**Figure 7.**
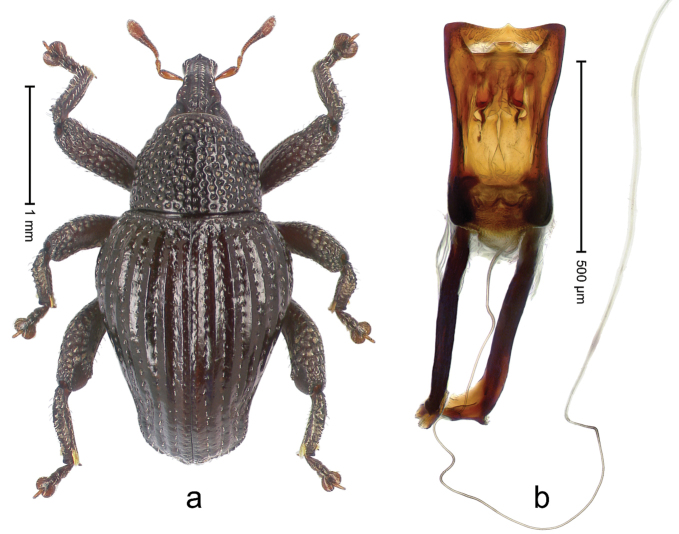
*Trigonopterus amphoralis* Marshall; **a** Habitus **b** Penis.

Selected male (Fig. 7a). Length 3.02 mm. Color of legs and antennae ferruginous, remainder black. Body elongate; in dorsal aspect with marked constriction between pronotum and elytron; in profile dorsally without constriction. Rostrum with median carina terminating abruptly on forehead; with pair of submedian ridges; intervening furrows each with row of erect, piliform scales; epistome with transverse, angulate ridge forming median denticle. Pronotum without distinct subapical constriction; disk densely punctate, interspaces glabrous; each puncture containing one small scale. Elytra with striae deeply impressed; each with row of partly abraded scales, in basal half scales suberect, apically small, recumbent; intervals weakly carinate, nude; interval 7 swollen subapically, laterally weakly projecting; apex extended ventrad, beak-shaped. Meso- and metafemur with crenulate anteroventral ridge. Metafemur subapically with stridulatory patch and transverse row of denticles. Metatibia with dorsal edge denticulate. Abdominal ventrite 5 basally flat, in apical half with shallow pit. Penis (Fig. 7b) with body in profile markedly curved ventrad; in dorsal aspect sides basally subparallel, in apical half diverging; apex with small median denticle; apodemes 1.8 × as long as body; ductus ejaculatorius without bulbus. **Intraspecific variation.** Length 2.53–3.02 mm. Color of elytra ferruginous or black. Female rostrum dorsally with pair of lateral furrows, pair of submedian furrows continued apicad by coarse punctures; epistome simple. Elytral apex in males rounded, in females narrower, subangulate. Female abdominal ventrite 5 flat.

##### Type material.

Syntypes: “Pedada Bay, Lampongs, 2 males, 22.I.1922 (Dr. K.W.Dammerman)”

##### Material examined

(MZB, SMNK, ZSM): East Sumatra, Lampung Prov.: 2 exx, ARC0261 (EMBL # LM655452), ARC0262 (EMBL # LM655453), Bawang, Pedada Bay, Mt. Tanggang, sample 2, S05°43.947', E105°06.480', 659 m, 09-VIII-2006; 1 ex, ARC0273 (EMBL # LM655464), Bawang, Pedada Bay, Mt. Tanggang, sample 4, S05°43.871', E105°06.393', 744 m, 09-VIII-2006; 2 exx, ARC0259 (EMBL # LM655451), ARC0260, Bawang, Pedada Bay, Mt. Tanggang, sample 1, S05°43.933', E105°06.598', 579 m, 09-VIII-2006; 1 ex, Bawang, Pedada Bay, Mt. Tanggang, sample 3, S05°43.938', E105°06.440', 673 m, 09-VIII-2006; 1 ex, Bawang, Pedada Bay, Mt. Tanggang, sample 4, S05°43.871', E105°06.393', 744 m, 09-VIII-2006; 6 exx, ARC2725 (EMBL # LM655981), ARC2726 (EMBL # LM655982), Bukit Barisan Selatan N.P., Kota Agung, Sukaraja, sample 2, S05°30.330', E104°25.838', 660 m, 01-V-2012; 4 exx, ARC2729 (EMBL # LM655985), Bukit Barisan Selatan N.P., Kota Agung, Sukaraja, sample 5, S05°31.044', E104°25.664', 562 m, 02-V-2012; 4 exx, Bukit Barisan Selatan N.P., Kota Agung, Sukaraja, sample 6, S05°31.678', E104°25.508', 608 m, 02-V-2012; 4 exx, Bukit Barisan Selatan N.P., Kota Agung, Sukaraja, sample 7, S05°33.768', E104°25.361', 371 m, 02-V-2012; 3 exx, Bukit Barisan Selatan N.P., Kota Agung, Sukaraja, sample 9, S05°32.611', E104°26.353', 455 m, 02-V-2012; 1 ex, Bukit Barisan Selatan N.P., Liwa, sample 6, S05°04.753', E104°03.183', 809 m, 05-V-2012; 2 exx, ARC2735 (EMBL # LM655991), ARC2736 (EMBL # LM655992), Bukit Barisan Selatan N.P., Liwa, sample 7, S05°04.958', E104°03.379', 813 m, 05-V-2012.

##### Distribution.

Lampung Prov. (Bukit Barisan Selatan, Pedada Bay). Elevation: 371–813 m.

##### Notes.

*Trigonopterus amphoralis* Marshall was coded as “*Trigonopterus* sp. 319”. The original description was based on two syntypes collected by Dammerman who was curator at the Zoological Museum Bogor. A search at MZB was without success, but a specimen bearing the syntypes´ original labels was located at BMNH. However, it is NOT the syntype: the characters (ovate body with smooth elytra) are in stark contrast to the original description and it belongs to a group never recorded west of Wallace Line, but very common to the east of it. Probably, several *Trigonopterus*-specimens in the same box became detached from their cards, and they were glued to the wrong cards in an attempt to restore order. The other specimens in the drawer were checked, but unfortunately none was found resembling the description of *Trigonopterus amphoralis*. Specimens collected at the type locality match the original description perfectly and thus the situation appears resolved sufficiently.

#### 8. Trigonopterus argopurensis

Taxon classificationAnimaliaColeopteraCurculionidae

Riedel sp. n.

http://zoobank.org/8309E929-9AA0-4A54-8356-9EF937828B08

##### Diagnostic description.

**Figure 8.**
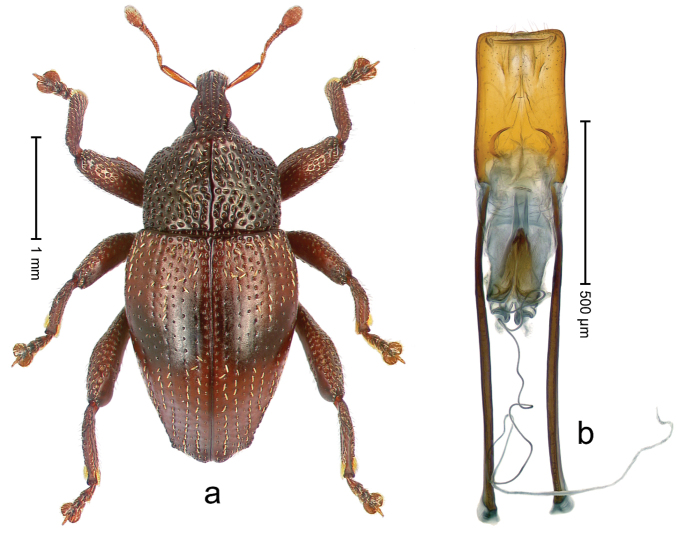
*Trigonopterus argopurensis* Riedel, sp. n., holotype; **a** Habitus **b** Penis.

Holotype, male (Fig. 8a). Length 3.28 mm. Color of legs and head ferruginous, elytra ferruginous with transverse sinuate black band, remainder black. Body elongate; in dorsal aspect with marked constriction between pronotum and elytron; in profile dorsally convex. Rostrum with median and pair of submedian ridges; intervening furrows punctate, each with row of erect setae; epistome simple. Pronotum with subapical constriction; disk densely punctate; punctures partly elongate and / or arranged in confluent rows forming rhombus-like pattern; each puncture containing elongate white scale or seta; medially with glabrous costa. Elytra with striae indistinct; intervals flat; with small, confused punctures; with sparse rows of elongate, recumbent, cream-colored scales; interspaces microreticulate; interval 7 in apical third forming sharp lateral edge; sutural interval at apex swollen, weakly protruding. Anteroventral ridge of femora crenulate, forming blunt tooth. Metafemur subapically with stridulatory patch. Dorsal edge of tibiae subbasally simple, with angulation. Metatibia apically without premucro. Abdominal ventrite 5 flat, coarsely punctate. Penis (Fig. 8b) with sides of body subparallel; apex medially with short median extension rounded; transfer apparatus compact, transfer-processes sickle-shaped, in profile with ventral contour evenly convex; apodemes 2.4 × as long as body; ductus ejaculatorius without bulbus. **Intraspecific variation.** Length 2.88–3.18 mm. Color largely light ferruginous or black except antennae and legs ferruginous. Female rostrum with median and pair of submedian ridges costate, subglabrous.

##### Material examined.

Holotype (MZB): ARC2457 (EMBL # LM655795), East Java Prov., Krucil, Bremi, Mt. Argopuro (trail to Taman Hidup), sample 2, S07°58.478', E113°31.069', 1457 m, 10-XI-2011. Paratypes (MZB, SMNK): E-Java Prov., Krucil, Bremi, Mt. Argopuro (trail to Taman Hidup): 5 exx, ARC2458 (EMBL # LM655796), ARC2459 (EMBL # LM655797), same data as holotype; 3 exx, ARC2464 (EMBL # LM655802), sample 3, S07°58.586', E113°31.345', 1628 m, 10-XI-2011, sample 4, S07°58.680', E113°31.519', 1785 m, 10-XI-2011; 1 ex, ARC2465 (EMBL # LM655803), sample 7, S07°58.449', E113°31.002', 1457 m, 11-XI-2011.

##### Distribution.

E-Java Prov. (Mt. Argopuro). Elevation: 1457–1785 m.

##### Etymology.

This epithet is based on the type locality, Mt. Argopuro.

##### Notes.

*Trigonopterus argopurensis* Riedel, sp. n. was coded as “*Trigonopterus* sp. 360” by Tänzler et al. (2014).

#### 9. Trigonopterus arjunensis

Taxon classificationAnimaliaColeopteraCurculionidae

Riedel sp. n.

http://zoobank.org/C60B9AC7-C79A-4175-ABBC-593DDD6F26ED

##### Diagnostic description.

**Figure 9.**
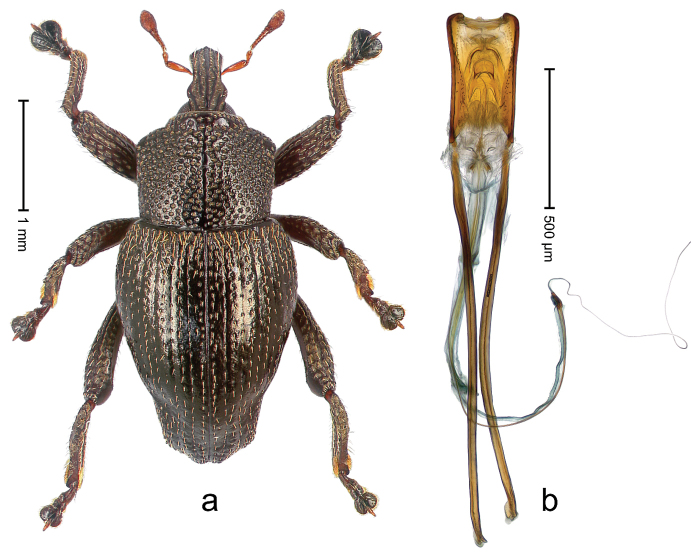
*Trigonopterus arjunensis* Riedel, sp. n., holotype; **a** Habitus **b** Penis.

Holotype, male (Fig. 9a). Length 3.20 mm. Color of legs and antennae ferruginous except tarsi black; remainder black. Body elongate; in dorsal aspect with marked constriction between pronotum and elytron; in profile dorsally flat, apically convex. Rostrum with median and pair of submedian ridges; intervening furrows each with sparse row of erect piliform scales; epistome simple. Pronotum with sides diverging apicad, anteriorly subangularly projecting; with indistinct subapical constriction; disk coarsely punctate; interspaces microgranulate; each puncture containing recumbent piliform scale directed laterad; with median ridge. Elytra with striae indistinct, marked by fine lines and rows of small punctures; intervals flat, microreticulate, with rows of small punctures; with sparse, recumbent, piliform scales, more conspicuous bordering basal margin; interval 7 in apical third forming sharp lateral edge; sutural interval at apex weakly swollen; apex subangulate, at suture with shallow notch. Femora edentate; anteroventral ridge crenulate. Metafemur subapically with stridulatory patch. Dorsal edge of tibiae subbasally angulate, mesotibia with angulation extended as acute tooth. Tarsomere 3 of protarsus slightly larger than of mesotarsus. Abdominal ventrite 5 flat, apically coarsely punctate, sparsely setose. Penis (Fig. 9b) with sides of body weakly diverging; apex medially pointed; transfer apparatus flagelliform, 4.0 × longer than body; apodemes 3.1 × as long as body; ductus ejaculatorius without bulbus. **Intraspecific variation.** Length 3.09–3.34 mm. Females more slender than males. Integument of males rather dull; females with punctures sparser and smaller, interspaces polished. Recumbent piliform scales in some specimens very small and sparse, almost absent in females. Female rostrum with median and pair of submedian glabrous costae.

##### Material examined.

Holotype (MZB): ARC2474 (EMBL # LM655811), East Java Prov., Mt. Arjuno, road Batu - Pacet, Cangar, sample 5, S07°43.400', E112°31.768', 1419 m, 15-XI-2011. Paratypes (MZB, SMNK, ZSM): E-Java Prov.: 4 exx, ARC2473 (EMBL # LM655810), ARC2475 (EMBL # LM655812), same data as holotype; 1 ex, ARC2476 (EMBL # LM655813), Mt. Arjuno, road Batu - Pacet, Cangar, sample 4, S07°43.247', E112°31.641', 1388 m, 15-XI-2011; 4 exx, ARC2477 (EMBL # LM655814), ARC2478 (EMBL # LM655815), ARC2479 (EMBL # LM655816), Kediri, Mt. Wilis, Besuki, sample 1, S07°51.785', E111°50.188', 1379 m, 21-XI-2011; 4 exx, Kediri, Besuki, Mt. Wilis, sample 6, S07°51.709', E111°50.100', 1409 m, 22-XI-2011; 9 exx, ARC2485 (EMBL # LM655822), ARC2486 (EMBL # LM655823), ARC2487 (EMBL # LM655824), ARC2488 (EMBL # LM655825), Kediri, Mt. Wilis, Besuki, sample 7, S07°51.638', E111°50.010', 1432 m, 22-XI-2011.

##### Distribution.

E-Java Prov. (Mt. Arjuno, Mt. Wilis). Elevation: 1388–1432 m.

##### Etymology.

This epithet is based on the type locality Mt. Arjuno.

##### Notes.

*Trigonopterus arjunensis* Riedel, sp. n. was coded as “*Trigonopterus* sp. 356”.

#### 10. Trigonopterus asper

Taxon classificationAnimaliaColeopteraCurculionidae

Riedel sp. n.

http://zoobank.org/04D150F6-D84C-409E-8E66-E753694C3031

##### Diagnostic description.

**Figure 10.**
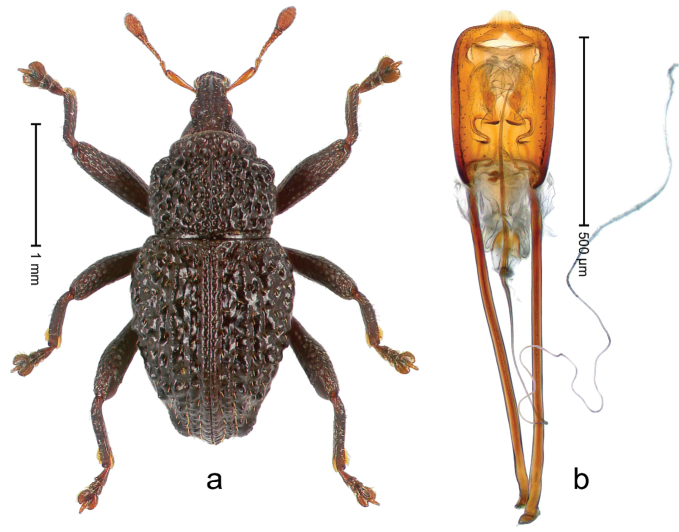
*Trigonopterus asper* Riedel, sp. n., holotype; **a** Habitus **b** Penis.

Holotype, male (Fig. 10a). Length 2.60 mm. Color of legs and head ferruginous, remainder black. Body elongate; in dorsal aspect with marked constriction between pronotum and elytron; with distinct constriction in profile. Rostrum coarsely rugose-punctate, with median ridge and pair of irregular submedian ridges; epistome with indistinct, transverse, subangulate ridge. Pronotum with distinct subapical constriction; disk scabrous, punctures each with one erect seta; with median ridge. Elytra with striae deeply impressed, each with row of stout suberect bristles; intervals tuberculate, almost nude; sutural interval apically extended; interval 7 swollen subapically, laterally weakly projecting. Femora edentate. Metafemur subapically with stridulatory patch. Abdominal ventrite 5 flat, densely punctate, setose. Penis (Fig. 10b) with body flattened, in profile markedly curved; in dorsal aspect sides subparallel; apex with median, subtriangular extension; transfer apparatus flagelliform, 1.5 × longer than body; apodemes 2.6 × as long as body; ductus ejaculatorius without bulbus. **Intraspecific variation.** Length 2.48–2.60 mm. Female rostrum dorsally with pairs of lateral and submedian furrows; epistome simple.

##### Material examined.

Holotype (MZB): ARC2520 (EMBL # LM655857), West Java Prov., Bandung, Lembang, Pangli, Mt. Bukittinggul, sample 3, S06°48.807', E107°43.897', 1983 m, 03-XII-2011. Paratypes (MZB, SMNK, ZSM): Bandung, Lembang, Pangli, Mt. Bukittinggul: 10 exx, ARC2521 (EMBL # LM655858), ARC3639 (EMBL # LM656061), same data as holotype; 4 exx, sample 2, S06°48.845', E107°44.074', 1898 m, 03-XII-2011; 10 exx, ARC2522 (EMBL # LM655859), sample 4, S06°48.660', E107°43.722', 2185 m, 03-XII-2011.

##### Distribution.

W-Java Prov. (Mt. Bukittinggul). Elevation: 1983–2185 m.

##### Etymology.

This epithet is based on the Latin adjective *asper* (rough, coarse) and refers to its body sculpture.

##### Notes.

*Trigonopterus asper* Riedel, sp. n. was coded as “*Trigonopterus* sp. 359”. It is closely related to *Trigonopterus variolosus* Riedel, sp. n. from which it can be separated by a larger penis with a longer flagellum. The minimal p-distance between both species is 9.5%.

#### 11. Trigonopterus attenboroughi

Taxon classificationAnimaliaColeopteraCurculionidae

Riedel sp. n.

http://zoobank.org/658E00BD-7CA8-455B-AAC4-B94BC37ABF28

##### Diagnostic description.

**Figure 11.**
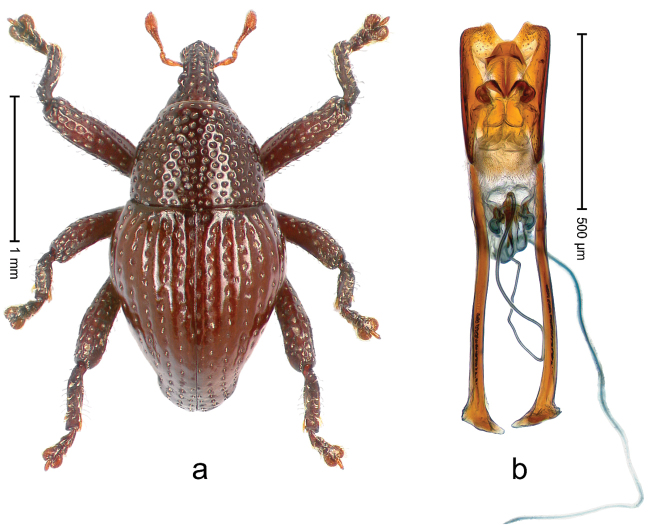
*Trigonopterus attenboroughi* Riedel, sp. n., holotype; **a** Habitus **b** Penis.

Holotype, male (Fig. 11a). Length 2.14 mm. Color ferruginous, head and pronotum darker, almost black. Body in dorsal aspect subovate, with weak constriction between pronotum and elytron; in profile dorsally convex. Rostrum with median and pair of submedian ridges; intervening furrows with rows of sparse suberect scales; epistome with subangulate ridge. Pronotum with disk densely punctate, reticulate; each puncture containing small recumbent scale. Elytra with striae distinct, with small punctures; intervals weakly costate, subglabrous; elytral apex subtruncate. Femora with crenate anteroventral ridge. Profemur in basal third with crenate posteroventral ridge. Metafemur subapically with stridulatory patch. Dorsal edge of tibiae with subbasal angulation, dentate in pro- and mesotibia. Abdominal ventrites 1–2 concave, forming common cavity, subglabrous, anteriorly with sparse row of long erect narrow scales; laterally with distinct rim; abdominal ventrite 2 in profile projecting dentiform; abdominal ventrite 5 with large round concavity, basally and laterally punctate. Penis (Fig. 11b) with sides of body slightly diverging; with pair of large orificial sclerites, slightly more basad with pair of smaller, darker sclerites; apex with distinct median incision; transfer apparatus spiniform; apodemes 2.2 × as long as body; ductus ejaculatorius without distinct bulbus. **Intraspecific variation.** Length 2.14–2.63 mm. Female unknown.

##### Material examined.

Holotype (MZB): ARC2543 (EMBL # LM655880), W-Kalimantan Prov., Bengkayan, Suka-Bangun, Mt. Bawang, sample 5, N00°54.103', E109°22.515', 652 m, 11-XII-2011 (MZB). Paratypes (SMNK): 1 ex, ARC2542 (EMBL # LM655879), same data as holotype.

##### Distribution.

W-Kalimantan Prov. (Mt. Bawang). Elevation: 652 m.

##### Etymology.

This species is named in honour of Sir David F. Attenborough in recognition of his outstanding documentaries on natural history.

##### Notes.

*Trigonopterus attenboroughi* Riedel, sp. n. was coded as “*Trigonopterus* sp. 366” by Tänzler et al. (2014).

#### 12. Trigonopterus baliensis

Taxon classificationAnimaliaColeopteraCurculionidae

Riedel sp. n.

http://zoobank.org/80141D9D-65DC-49D3-B30B-829557575B64

##### Diagnostic description.

**Figure 12.**
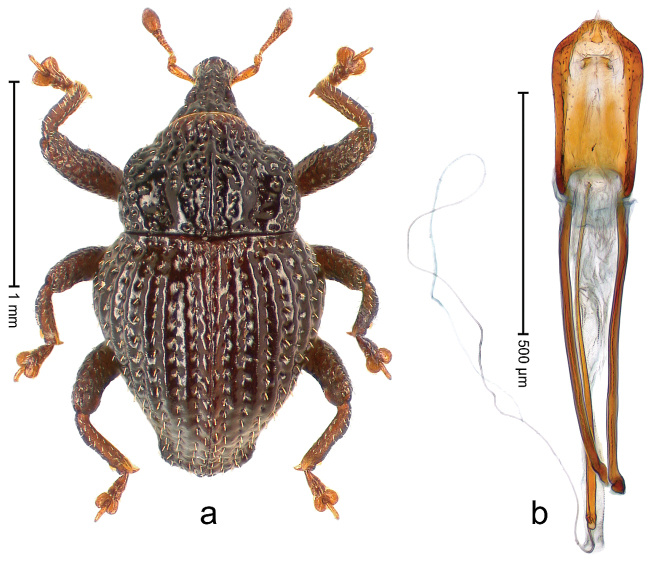
*Trigonopterus baliensis* Riedel, sp. n., holotype; **a** Habitus **b** Penis.

Holotype, male (Fig. 12a). Length 1.79 mm. Color of head and legs ferruginous, elytra near sutural margin deep ferruginous, remainder black. Body in dorsal aspect with marked constriction between pronotum and elytron; with distinct constriction in profile. Rostrum with median ridge and pair of submedian ridges, intervening furrows with sparse rows of setae; epistome with transverse, subangulate ridge. Pronotum with indistinct subapical constriction; disk coarsely punctate-rugose, sparsely setose; in basal half with pair of sublateral, kidney-shaped impressions; with median ridge. Elytra with striae deeply impressed, each with row of slender, suberect scales; intervals costate, subglabrous; apical margin subangulate. Femora each with small to minute tooth. Metafemur subapically with indistinct stridulatory patch. Abdominal ventrite 5 with pair of submedian ridges, punctate, sparsely setose. Penis (Fig. 12b) with sides of body subparallel; in apical quarter markedly converging to rounded apex, with minute median brush of setae; transfer apparatus flagelliform, 2.6 × longer than body; apodemes 2.0 × as long as body; ductus ejaculatorius without bulbus. **Intraspecific variation.** Length 1.46–1.90 mm. Color of elytra ferruginous to black. Female rostrum dorsally in apical half subglabrous, with sparse minute punctures; epistome simple. Pronotum in females and in small males relatively narrow, in large males wider. Elytra in females narrower, humeri evenly rounded; large males with humeri more distinctly, subangularly projecting laterad, in small males similar to females; intervals weakly costate, in larger specimens markedly costate or carinate.

##### Material examined.

Holotype (MZB): ARC0580 (EMBL # LM655516), Bali, Bedugul, Tamblingan, above the village along water pipe, sample 4, S08°15.854', E115°05.312', 1310 m, 06-XI-2007. Paratypes (ARC, MZB, SMNK, ZSM): Bali: 7 exx, ARC166, Bedugul, mountain NW of botanical garden, 1400 m, 04-XII-2004 (ARC); 1 ex, ARC0575 (EMBL # LM655511), Bedugul, Mt. Pohen, S08°16.526', E115°08.634', 1580 m, 01-XI-2007; 10 exx, ARC0576 (EMBL # LM655512), ARC0577 (EMBL # LM655513), ARC0578 (EMBL # LM655514), ARC0579 (EMBL # LM655515), Bedugul, Mt. Pohen, S08°16.643', E115°08.875', 1405 m, 01-XI-2007; 6 exx, Bedugul, Mt. Pohen, sample 6, S08°16.607', E115°08.773', 1460 m, 01-XI-07; 15 exx, ARC0581 (EMBL # LM655517), ARC0582 (EMBL # LM655518), ARC0583 (EMBL # LM655519), same data as holotype; 43 exx, Tamblingan, sample 5, S08°15.108', E115°05.573', 1350 m, 03-IV-2011; 2 exx, Tamblingan, sample 1, S08°16.029', E115°05.854', 1210 m, 06-XI-2007; 2 exx, Tamblingan, sample 2, S08°15.015', E115°06.202', 1255 m, 06-XI-2007; 13 exx, Lake Tamblingan, 1250 m, 18-19-XII-2008; 1 ex, ARC0588 (EMBL # LM655524), Bedugul, Mt. Catur, S08°15.626', E115°11.354', 1690 m, 07-XI-2007; 26 exx, ARC2307 (EMBL # LM655760), ARC2308 (EMBL # LM655761), ARC2309 (EMBL # LM655762), Mt. Batukaru, Wangayagede, sample 3, S08°21.431', E115°05.699', 1170 m, 31-III-2011; 49 exx, Mt. Batukaru, Wangayagede, sample 4, S08°21.373', E115°05.564', 1310 m, 31-III-2011; 53 exx, Mt. Batukaru, Wangayagede, sample 5, S08°21.130', E115°05.321', 1520 m, 31-III-2011; 8 exx, Angseri, Mt. Adeng, sample 1, S08°19.824', E115°08.604', 1190 m, 05-IV-2011; 8 exx, Angseri, Mt. Adeng, sample 2, S08°19.726', E115°08.583', 1280 m, 06-IV-2011; 39 exx, ARC2319 (EMBL # LM655772), ARC2320 (EMBL # LM655773), Angseri, Mt. Adeng, sample 3, S08°19.626', E115°08.554', 1385 m, 06-IV-2011; 1 ex, Angseri, Mt. Adeng, sample 4, S08°19.463', E115°08.537', 1535 m, 06-IV-2011; 4 exx, ARC0730 (EMBL # LM655547), ARC0731 (PCR failed), Pangkung village, trail to Mesehe-waterfall, S08°18.30', E114°41.68', 350-600 m, 14 &17-XII-2008; 3 exx, ARC2252 (EMBL # LM655707), ARC2253 (EMBL # LM655708), ARC2254 (PCR failed), Negara, Kampung Pasatan, Desa Pohsanten, Mt. Mesehe, sample 5, S08°16.589', E114°41.772', 745 m, 18-III-2011; 1 ex, Negara, Kampung Pasatan, Desa Pohsanten, Mt. Mesehe, sample 4, S08°17.466', E114°41.331', 490 m, 17-III-2011; 17 exx, Negara, Kampung Pasatan, Desa Pohsanten, Mt. Mesehe, sample 5, S08°16.589', E114°41.772', 745 m, 18-III-2011; 4 exx, Negara, Kampung Pasatan, Desa Pohsanten, Mt. Mesehe, sample 6, S08°16.234', E114°41.528', 935 m, 18-III-2011; 1 ex, Negara, Kampung Pasatan, Desa Pohsanten, Mt. Mesehe, sample 7, S08°16.314', E114°41.649', 890 m, 18-III-2011; 52 exx, ARC2959 (EMBL # LM656012), ARC2960 (EMBL # LM656013), ARC2961 (EMBL # LM656014), Negara, Kampung Pasatan, Desa Pohsanten, Mt. Mesehe, sample 8, S08°16.454', E114°41.725', 825 m, 18-III-2011.

##### Distribution.

Bali (Mt. Andeng, Mt. Batukaru, Bedugul, Mt. Mesehe). Elevation: 600–1690 m.

##### Etymology.

This epithet is based on the island of Bali.

##### Notes.

*Trigonopterus baliensis* Riedel, sp. n. was coded as “*Trigonopterus* sp. 328” by Tänzler et al. (2014).

#### 13. Trigonopterus batukarensis

Taxon classificationAnimaliaColeopteraCurculionidae

Riedel sp. n.

http://zoobank.org/79CC0B55-E102-4860-A005-3005A0E929BE

##### Diagnostic description.

**Figure 13.**
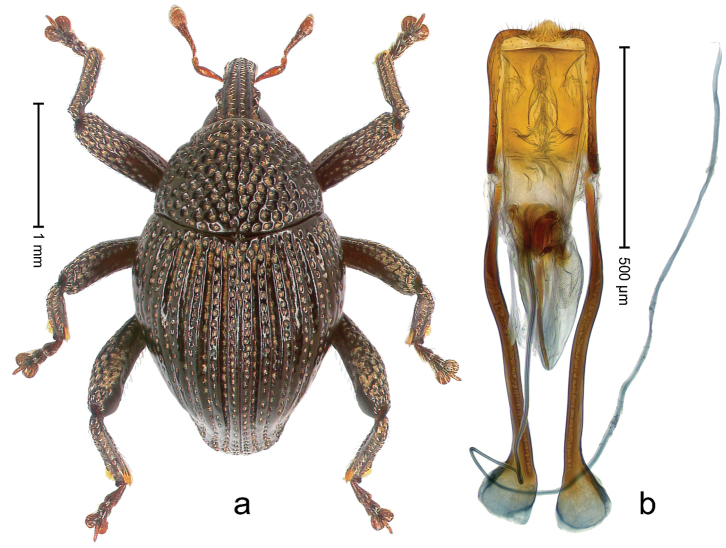
*Trigonopterus batukarensis* Riedel, sp. n., holotype; **a** Habitus **b** Penis.

Holotype, male (Fig. 13a). Length 2.78 mm. Color of antennae and legs ferruginous; remainder black. Body subovate, in dorsal aspect with weak constriction between pronotum and elytron; in profile dorsally convex. Rostrum with median ridge and pair of submedian ridges, intervening furrows each with sparse row of mesad directed scales; epistome with transverse ridge. Pronotum coarsely punctate, reticulate, with indistinct median costa; with sparse, recumbent scales. Elytra with striae dorsally deeply incised, laterally marked by rows of coarse punctures; intervals markedly costate, dorsally flattened, with rows of punctures; sutural interval basally hardly widened; each puncture containing small recumbent scale; elytral apex subtruncate. Femora with simple anteroventral ridge. Metafemur subapically with stridulatory patch. Abdominal ventrite 5 coarsely punctate, with sparse suberect scales. Penis (Fig. 13b) with sides of body subparallel; apex with median angulate extension; transfer apparatus with thin median rod 1.4 × as long as body; median rod projecting basad; apodemes 2.6 × as long as body; ductus ejaculatorius without bulbus. **Intraspecific variation.** Length 2.48–2.78 mm. Color of elytra dark ferruginous to black. Female rostrum in apical half slender, dorsally subglabrous, with submedian rows of punctures, laterally punctate-rugose; epistome simple.

##### Material examined.

Holotype (MZB): ARC2303 (EMBL # LM655757), Bali, Mt. Batukaru, Wangayagede, sample 3, S08°21.431', E115°05.699', 1170 m, 31-III-2011. Paratypes (MZB, SMNK): Bali, Mt. Batukaru, Wangayagede: 3 exx, ARC2304 (EMBL # LM655758), ARC2305 (PCR failed), same data as holotype; 1 ex, ARC2302 (EMBL # LM655756), sample 1, S08°22.295', E115°06.048', 835 m, 31-III-2011; 1 ex, Mt. Batukaru, Wangayagede, sample 4, S08°21.373', E115°05.564', 1310 m, 31-III-2011; 3 exx, ARC2951 (EMBL # LM656004), ARC2952 (EMBL # LM656005), ARC2953 (EMBL # LM656006), sample 4, S08°21.373', E115°05.564', 31-III-2011.

##### Distribution.

Bali (Mt. Batukaru). Elevation: 835–1310 m.

##### Etymology.

This epithet is based on the type locality Mt. Batukaru.

##### Notes.

*Trigonopterus batukarensis* Riedel, sp. n. was coded as “*Trigonopterus* sp. 340” by Tänzler et al. (2014).

#### 14. Trigonopterus bawangensis

Taxon classificationAnimaliaColeopteraCurculionidae

Riedel sp. n.

http://zoobank.org/7BC78CF5-ABEE-43A8-B5A7-B8F8696E51DB

##### Diagnostic description.

**Figure 14.**
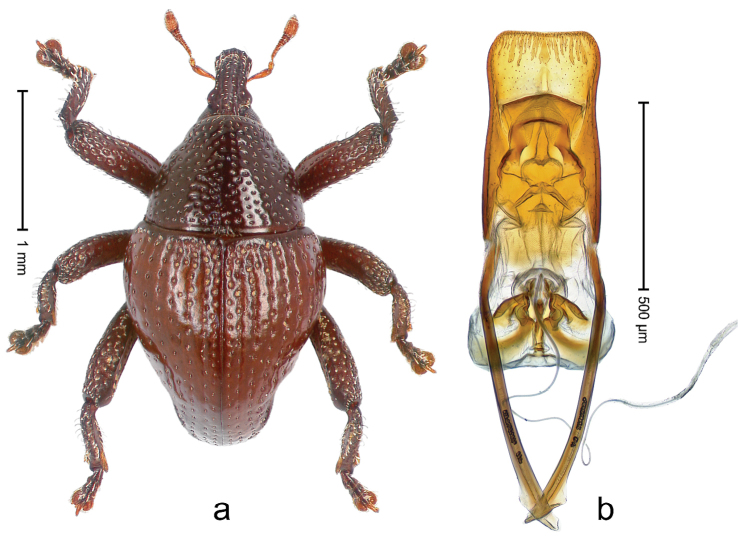
*Trigonopterus bawangensis* Riedel, sp. n., holotype; **a** Habitus **b** Penis.

Holotype, male (Fig. 14a). Length 2.38 mm. Color ferruginous, pronotum dark ferruginous, almost black. Body in dorsal aspect subrhomboid, with weak constriction between pronotum and elytron; in profile dorsally convex. Rostrum with median and pair of indistinct submedian ridges; coarsely punctate-reticulate, with sparse rows of suberect scales; in front of forehead median ridge swollen, projecting subangularly from profile; epistome with subangulate ridge. Pronotum with disk densely punctate; interspaces subglabrous, weakly microreticulate; each puncture containing small recumbent seta. Elytra with striae distinct, marked by coarse punctures; intervals flat, microreticulate; sutural intervals with row of smaller punctures; each puncture containing recumbent seta; elytral apex subtruncate, in apical aspect ventral outline bisinuate. Anteroventral ridge of femora indistinct, terminating in apical third. Metafemur subapically with stridulatory patch. Dorsal edge of tibiae with subbasal angulation, dentate in pro- and mesotibia. Abdominal ventrites 1–2 subglabrous, with few long erect narrow scales, laterally with swollen rim; abdominal ventrite 2 in profile projecting dentiform; abdominal ventrite 5 flat, coarsely punctate, with sparse suberect scales. Penis (Fig. 14b) with sides of body subparallel, near middle with shallow constriction; apex subtruncate, without setae; transfer apparatus complex, wider than long; apodemes 1.5 × as long as body; ductus ejaculatorius without bulbus. **Intraspecific variation.** Length 1.90–2.48 mm. Color of elytra light or dark ferruginous. Female rostrum dorsally medially subglabrous, sublaterally punctate-rugose; epistome simple.

##### Material examined.

Holotype (MZB): ARC2535 (EMBL # LM655872), W-Kalimantan Prov., Bengkayan, Suka-Bangun, Mt. Bawang, sample 1, N00°53.429', E109°22.230', 246 m, 10-XII-2011. Paratypes (MZB, SMNK, ZSM): W-Kalimantan Prov., Bengkayan, Suka-Bangun, Mt. Bawang: 3 exx, ARC2536 (EMBL # LM655873), same data as holotype; 3 exx, ARC2534 (EMBL # LM655871), sample 2, N00°53.514', E109°22.301', 275 m, 10-XII-2011; 3 exx, ARC2537 (EMBL # LM655874), sample 3, N00°53.621', E109°22.475', 400 m, 10-XII-2011; 3 exx, sample 6, N00°53.992', E109°22.502', 556 m, 11-XII-2011; 1 ex, ARC2546 (EMBL # LM655883), sample 8, N00°53.736', E109°22.316', 411 m, 11-XII-2011.

##### Distribution.

W-Kalimantan Prov. (Mt. Bawang). Elevation: 246–556 m.

##### Etymology.

This epithet is based on the type locality.

##### Notes.

*Trigonopterus bawangensis* Riedel, sp. n. was coded as “*Trigonopterus* sp. 364”.

#### 15. Trigonopterus binodulus

Taxon classificationAnimaliaColeopteraCurculionidae

Riedel sp. n.

http://zoobank.org/0199A885-5EB4-417E-AFFA-2366842AB040

##### Diagnostic description.

**Figure 15.**
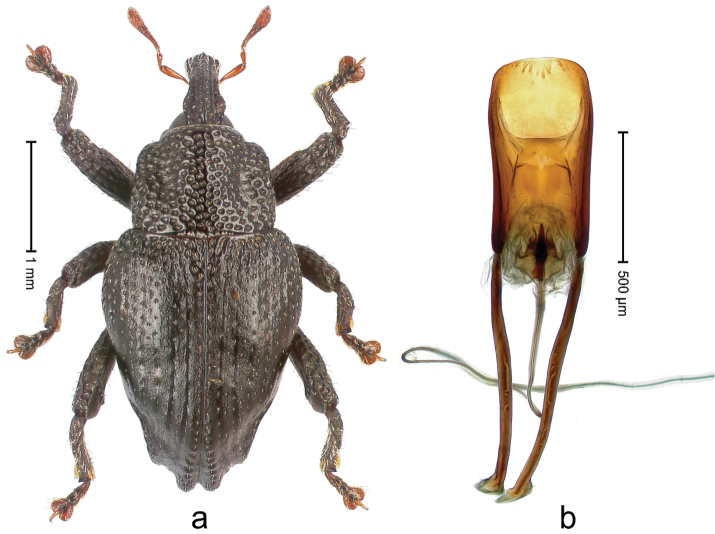
*Trigonopterus binodulus* Riedel, sp. n., holotype; **a** Habitus **b** Penis.

Holotype, male (Fig. 15a). Length 3.38 mm. Color of tarsi and antennae ferruginous, remainder black. Body elongate; in dorsal aspect with marked constriction between pronotum and elytron; in profile dorsally evenly convex. Rostrum with median carina terminating on forehead and pair of submedian ridges; intervening furrows each with row of erect piliform scales; epistome with transverse, angulate ridge forming median denticle. Pronotum anterolaterally subangularly projecting; with subapical constriction; disk coarsely punctate, interspaces dull, microreticulate; each puncture containing small seta; disk with pair of submedian, shallow impressions. Elytra with striae indistinct; intervals flat; punctation confused; each puncture containing small recumbent seta; interspaces dull, coriaceous, microreticulate; interval 7 swollen subapically, weakly projecting laterally; sutural interval at apex markedly swollen, forming pair of rounded apical protrusions. Anteroventral ridge of femora forming blunt tooth. Metafemur subapically with stridulatory patch and transverse row of denticles. Abdominal ventrite 5 with broad impression, sparsely setose with long erect setae. Penis (Fig. 15b) with sides of body subparallel; apex broadly rounded; transfer apparatus small, spiniform; apodemes 1.5 × as long as body; ductus ejaculatorius without bulbus. **Intraspecific variation.** Length 2.78–4.20 mm. Integument of males dull, coriaceous-microreticulate; females with interspaces between punctures polished. Female rostrum dorsally with glabrous median costa bordered by rows of punctures and sublateral pair of furrows; epistome simple. Female elytra with lateral contour in basal half evenly convex; intervals 2–3 subapically costate. Male elytra with lateral contour in basal half sinuate, concave at level of hind legs; intervals 2–3 subapically with indistinct ridge.

##### Material examined.

Holotype (MZB): ARC0202 (EMBL # LM655424), West Java Prov., Ciamis, Mt. Sawal, Batu Cakra, sample 1, S07°14.920', E108°15.762', 990 m, 01-X-2005 (MZB). Paratypes (MZB, SMNK, ZSM): W-Java Prov., Ciamis: 13 exx, ARC0160, ARC0171, ARC0201 (EMBL # LM655423), ARC0203 (EMBL # LM655425), ARC0218 (EMBL # LM655440), same data as holotype; 2 exx, ARC2688 (EMBL # LM655946), ARC2689 (EMBL # LM655947), Pangandaran, sample 3, S07°43.070', E108°39.634', 156 m, 22-IV-2012.

##### Distribution.

W-Java Prov. (Pangandaran, Mt. Sawal). Elevation: 156–990 m.

##### Etymology.

This epithet is a combination of the Latin prefix *bi*- (two) and the noun *nodulus* (small swelling) and refers to the structure of the elytral apex.

##### Notes.

*Trigonopterus binodulus* Riedel, sp. n. was coded as “*Trigonopterus* sp. 315”.

#### 16. Trigonopterus bornensis

Taxon classificationAnimaliaColeopteraCurculionidae

Riedel sp. n.

http://zoobank.org/6781C63F-24E6-4189-BFCD-9D81C1D3B69D

##### Diagnostic description.

**Figure 16.**
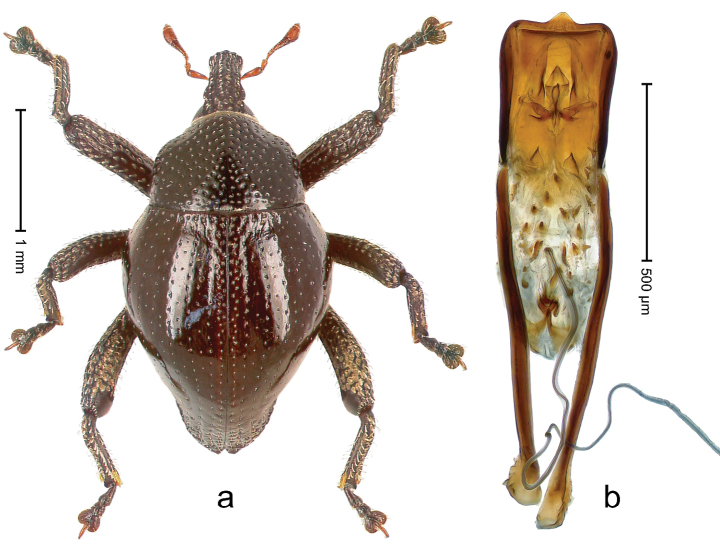
*Trigonopterus bornensis* Riedel, sp. n., holotype; **a** Habitus **b** Penis.

Holotype, male (Fig. 16a). Length 2.81 mm. Color of antennae light ferruginous, pronotum black, remainder dark ferruginous. Body in dorsal aspect with marked constriction between pronotum and elytron; in profile dorsally convex. Rostrum with median and pair of indistinct submedian ridges; intervening furrows containing row of coarse punctures, with rows of erect scales; epistome with transverse, irregular ridge. Pronotum with sides subparallel in basal half, anteriorly rounded to indistinct subapical constriction; disk densely punctate; interspaces subglabrous; each puncture containing small recumbent seta. Elytra with striae marked by small punctures, each containing minute seta; intervals flat, subglabrous, with few interspersed punctures; sutural intervals with additional row of punctures; elytral apex pointed, densely coarsely punctate, suture incised. Femora with crenate anteroventral ridge. Metafemur subapically with stridulatory patch. Dorsal edge of tibiae with subbasal angulation, dentate in pro- and mesotibia. Abdominal ventrites 1–2 weakly concave to flat, subglabrous, with sparse erect scales; abdominal ventrite 5 flat, basally subglabrous, apically sparsely punctate, microreticulate, with sparse erect scales. Penis (Fig. 16b) with sides of body subparallel, slightly diverging; apex subtruncate, with median, triangular extension; endophallus containing numerous coarse denticles, apically with pair of sclerites; transfer apparatus digiform, slightly curved; apodemes 2.5 × as long as body; ductus ejaculatorius without distinct bulbus. **Intraspecific variation.** Length 2.35–2.81 mm. Female rostrum dorsally subglabrous, with submedian row of coarse punctures, subapically punctate, sublaterally with sparse rows of subrecumbent scales; epistome simple. Female elytral apex simple.

##### Material examined.

Holotype (MZB): ARC0710 (EMBL # LM655535), E-Kalimantan Prov., Berau Dist., Hutan Wisata Sei Tangap, ca. 8 km W of Tanjungredeb, N02°08.07', E117°24.65', 30 m, 02-X-2008. Paratypes (MZB, SMNK): E-Kalimantan Prov., Berau Dist.: 9 exx, ARC0711 (EMBL # LM655536), ARC0712 (EMBL # LM655537), ARC0713 (EMBL # LM655538), same data as holotype; 1 ex, Hutan Mayang Mangurai, ca. 15 km SW of Tanjungredeb, N02°06.217', E117°24.003‘, 20m, 30-XI-2008.

##### Distribution.

E-Kalimantan Prov. (Tanjungredeb). Elevation: 20–30 m.

##### Etymology.

This epithet is based on the island of Borneo.

##### Notes.

*Trigonopterus bornensis* Riedel, sp. n. was coded as “*Trigonopterus* sp. 311”.

#### 17. Trigonopterus cahyoi

Taxon classificationAnimaliaColeopteraCurculionidae

Riedel sp. n.

http://zoobank.org/94508408-EEF0-4480-931B-6FF5D218FB29

##### Diagnostic description.

**Figure 17.**
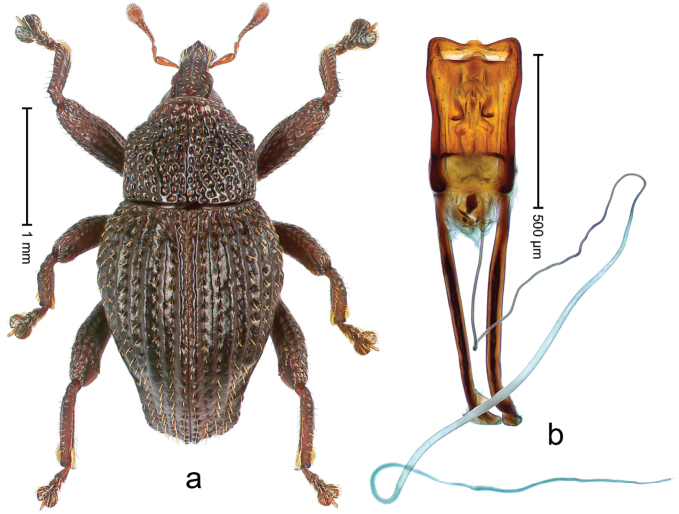
*Trigonopterus cahyoi* Riedel, sp. n., holotype; **a** Habitus **b** Penis.

Holotype, male (Fig. 17a). Length 3.03 mm. Color of legs and head ferruginous, remainder black. Body elongate; in dorsal aspect with marked constriction between pronotum and elytron; in profile dorsally with indistinct constriction. Rostrum with median carina terminating on forehead; with pair of submedian ridges; intervening furrows each with row of erect, piliform scales; anteriorly behind epistome ridges shortened; epistome with transverse, angulate ridge forming distinct median denticle. Pronotum anterolaterally subangularly projecting; with indistinct subapical constriction; disk coarsely rugose-punctate; each puncture containing suberect piliform scale; with median ridge. Elytra with striae deeply impressed; each with row of suberect piliform scales; intervals costate, ridges transversely rugose; costa of interval 2 shortened at base; interval 7 swollen subapically, laterally weakly projecting; apex extended ventrad, beak-shaped. Meso- and metafemur with anteroventral ridge forming indistinct blunt tooth. Metafemur subapically with stridulatory patch and transverse row of denticles. Abdominal ventrite 5 subapically with shallow pit. Penis (Fig. 17b) with body in profile moderately curved ventrad; in dorsal aspect sides subparallel in basal half, anteriorly gently diverging; apex medially with small rounded extension; apodemes 2.0 × as long as body; ductus ejaculatorius without bulbus. **Intraspecific variation.** Length 2.05–3.19 mm. Color of elytra completely black or base and sutural interval ferruginous. Body of females slender; males slightly wider, especially between humeri. Female rostrum dorsally with pair of lateral furrows, pair of submedian furrows continued apicad by coarse punctures; epistome simple. Female abdominal ventrite 5 flat.

##### Material examined.

Holotype (MZB): ARC2678 (EMBL # LM655936), West Java, Prov., Sumedang, Sukajadi, Mt. Cakrabuana, sample 6, S07°01.943', E108°08.029', 1593 m, 20-IV-2012. Paratypes (MZB, SMNK, ZSM): W-Java Prov.: 9 exx, Sumedang, Sukajadi, Mt. Cakrabuana, sample 4, S07°01.734', E108°08.059', 1530 m, 19-IV-2012; 8 exx, same data as holotype; 59 exx, ARC0155, ARC0172, ARC0193 (EMBL # LM655417), ARC0194 (EMBL # LM655418), ARC0195 (EMBL # LM655419), Ciamis, Mt. Sawal, Batu Cakra, sample 1, S07°14.920', E108°15.762', 990 m, 01-X-2005 (MZB); 13 exx, sample 3, S07°14.900', E108°15.893', 940 m, 01-X-2005; 11 exx, Ciamis, Mt. Sawal, Blok Cireong, sample 2, S07°14.127', E108°15.568', 1120 m, 01-X-2005; 3 exx, ARC2504 (EMBL # LM655841), Ciamis, Mt. Sawal, Panjalu, Tembong, sample 1, S07°10.528', E108°16.423', 1314 m, 29-XI-2011; 8 exx, ARC2508 (EMBL # LM655845), Ciamis, Mt. Sawal, Panjalu, Tembong, sample 3, S07°11.102', E108°16.310', 1569 m, 29-XI-2011.

##### Distribution.

W-Java Prov. (Mt. Cakrabuana, Mt. Sawal). Elevation: 990–1593 m.

##### Etymology.

The species is named for arachnologist Cahyo Rahmadi, who collected the first specimens of this species. The epithet is a noun in the genitive case.

##### Notes.

*Trigonopterus cahyoi* Riedel, sp. n. was coded as “*Trigonopterus* sp. 320” by Tänzler et al. (2014).

#### 18. Trigonopterus costipennis

Taxon classificationAnimaliaColeopteraCurculionidae

Riedel sp. n.

http://zoobank.org/22BB17BD-B217-49A3-8F13-DF9977A37AFD

##### Diagnostic description.

**Figure 18.**
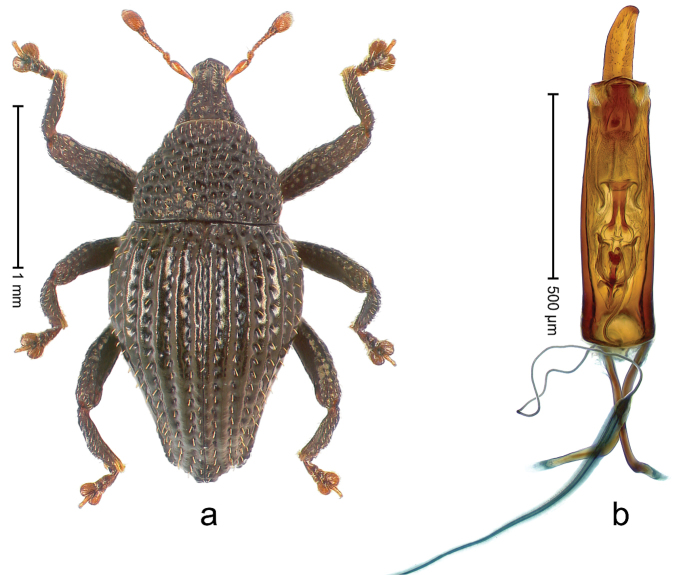
*Trigonopterus costipennis* Riedel, sp. n., holotype; **a** Habitus **b** Penis.

Holotype, male (Fig. 18a). Length 2.32 mm. Color of tarsi and antennae ferruginous; remainder black. Body elongate subovate; in dorsal aspect and in profile with distinct constriction between pronotum and elytron. Rostrum with median ridge and pair of submedian ridges, anteriorly behind epistome scabrous; with sparse rows of suberect setae; epistome with transverse, angulate ridge. Pronotum with subapical constriction; disk coarsely punctate, scabrous; each puncture containing suberect, weakly clavate scale inserting at posterior rim. Elytra with striae marked by deep punctures; in front of each puncture with piliform, erect scale; intervals costate, subglabrous, weakly microreticulate; sutural interval and interval 7 swollen subapically, projecting from outline. Femora edentate. Metafemur subapically without stridulatory patch. Dorsal edge of meso- and metatibia denticulate. Abdominal ventrite 5 flat, microreticulate, almost nude, with few short setae. Penis (Fig. 18b) with apex asymmetrical, tip slightly upcurved and extended to the left; apical extension long; basal orifice ventrally simple; apodemes short, 0.5 × as long as body; ductus ejaculatorius with bulbus. **Intraspecific variation.** Length 2.04–2.51 mm. Female rostrum dorsally in apical half subglabrous, densely punctate; epistome simple.

##### Material examined.

Holotype (MZB): ARC2470 (EMBL # LM655807), E-Java, Mt. Semeru, road Senduro - Ranupani, sample 4, S08°01.801', E112°59.931', 1430 m, 13-XI-2011. Paratypes (MZB, SMNK, ZSM): E-Java Prov.: 2 exx, ARC2469 (EMBL # LM655806), ARC2472 (EMBL # LM655809), same data as holotype; 2 exx, ARC2471 (EMBL # LM655808), Mt. Semeru, road Senduro - Ranupani, sample 3, S08°02.052', E112°59.356', 1572 m, 13-XI-2011; 6 exx, Mt. Semeru, road Senduro - Ranupani, sample 4, S08°01.801', E112°59.931', 1430 m, 13-XI-2011; 1 ex, Mt. Semeru, road Senduro - Ranupani, sample 5, S08°01.952', E113°00.379', 1345 m, 13-XI-2011; 18 exx, ARC2480 (EMBL # LM655817), ARC2481 (EMBL # LM655818), Kediri, Besuki, Mt. Wilis, sample 1, S07°51.785', E111°50.188', 1379 m, 21-XI-2011; 12 exx, Kediri, Besuki, Mt. Wilis, sample 4, S07°51.852', E111°50.185', 1388 m, 22-XI-2011; 4 exx, ARC2489 (EMBL # LM655826), ARC2490 (EMBL # LM655827), Kediri, Besuki, Mt. Wilis, sample 5, S07°51.650', E111°50.174', 1485 m, 22-XI-2011; 10 exx, Kediri, Besuki, Mt. Wilis, sample 6, S07°51.709', E111°50.100', 1409 m, 22-XI-2011; 15 exx, Kediri, Besuki, Mt. Wilis, sample 7, S07°51.638', E111°50.010', 1432 m, 22-XI-2011.

##### Distribution.

E-Java Prov. (Mt. Semeru, Mt. Wilis). Elevation: 1345–1572 m.

##### Etymology.

This epithet is a combination of the Latin nouns *costa* (rib, ridge) and *penna* (elytron) refers to its elytral sculpture.

##### Notes.

*Trigonopterus costipennis* Riedel, sp. n. was coded as “*Trigonopterus* sp. 358”.

#### 19. Trigonopterus cuprescens

Taxon classificationAnimaliaColeopteraCurculionidae

Riedel sp. n.

http://zoobank.org/25F66BF2-F190-4E36-ADDF-2AD8DE91E94D

##### Diagnostic description.

**Figure 19.**
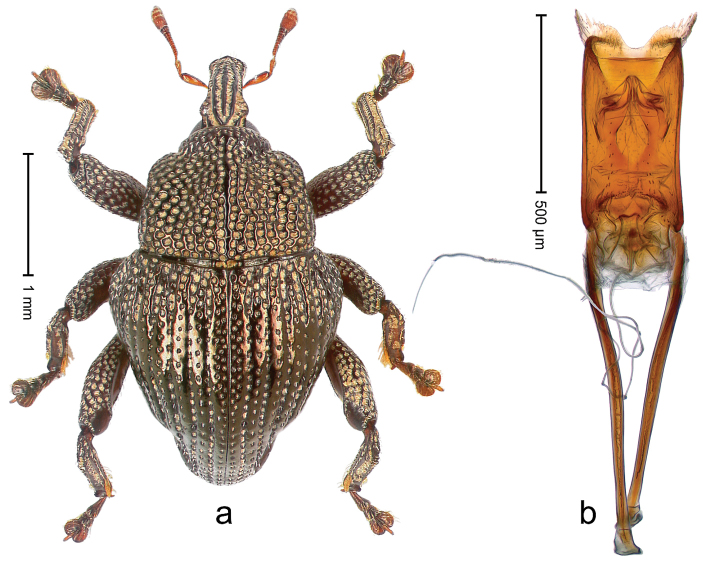
*Trigonopterus cuprescens* Riedel, sp. n., holotype; **a** Habitus **b** Penis.

Holotype, male (Fig. 19a). Length 3.00 mm. Color of legs and antennae ferruginous; remainder black, pronotum and elytron bronze-coppery. Body in dorsal aspect with marked constriction between pronotum and elytron; in profile convex. Rostrum with median ridge; pair of submedian ridges converging and uniting on forehead; intervening furrows each with sparse row of suberect elongate scales; in apical third coarsely punctate; epistome with transverse, angulate ridge. Pronotum with sides subparallel, slightly converging; with distinct, subapical constriction; disk coarsely punctate, reticulate; each puncture containing brown recumbent seta; with pair of submedian impressions, medially swollen, with indistinct median ridge. Elytra with humeri swollen, laterally projecting; striae distinct, deeply impressed; intervals costate, punctate; punctures near base coarse and dense; near middle transverse band polished. Profemur simple; meso- and metafemur with anteroventral ridge forming large, acute tooth. Metafemur subapically with stridulatory patch. Dorsal tibial edge subbasally with indistinct angulation. Abdominal ventrite 5 flat, densely punctate, with erect setae. Penis (Fig. 19b) with sides of body subparallel; apex rounded, with fringe of conglutinate flattened setae, interrupted by glabrous median notch; transfer apparatus compact; apodemes 2.0 × as long as body; ductus ejaculatorius without bulbus. **Intraspecific variation.** Length 2.72–3.05 mm. Bronze color of pronotum and elytron with slightly greenish or reddish lustre. Female body more slender. Female rostrum dorsally punctate-rugose, with median and pair of submedian subglabrous costae; epistome simple. Female elytron with humeri less prominent, with subapical constriction and apex extended, suture with notch. Female abdominal ventrite 5 with sparse subrecumbent setae.

##### Material examined.

Holotype (MZB): ARC2235 (EMBL # LM655691), East Nusa Tenggara Prov., Flores, Labuhan Bajo, Tebedo, sample 1, S08°29.848', E119°59.633', 525 m, lowland forest, 14-III-2011. Paratypes (MZB, SMNK): Flores, Labuhan Bajo: 3 exx, ARC2233 (EMBL # LM655689), ARC2234 (EMBL # LM655690), same data as holotype; 1 ex, Roe, sample 4, S08°35.442', E120°00.366', 785 m, thick litter under *Ficus*-tree, 13-III-2011; 1 ex, ARC2215 (EMBL # LM655671), Roe, earthworm-dominated habitat, sample 1, S08°36.540', E120°01.871', 955 m, 13-III-2011.

##### Distribution.

East Nusa Tenggara Prov., Flores (Labuhan Bajo). Elevation: 525–955 m.

##### Etymology.

This epithet is based on the Latin adjective *cuprescens* (coppery).

##### Notes.

*Trigonopterus cuprescens* Riedel, sp. n. was coded as “*Trigonopterus* sp. 347”.

#### 20. Trigonopterus cupreus

Taxon classificationAnimaliaColeopteraCurculionidae

Riedel sp. n.

http://zoobank.org/0B67C9C1-52A5-4942-A840-9EE2A787D8D3

##### Diagnostic description.

**Figure 20.**
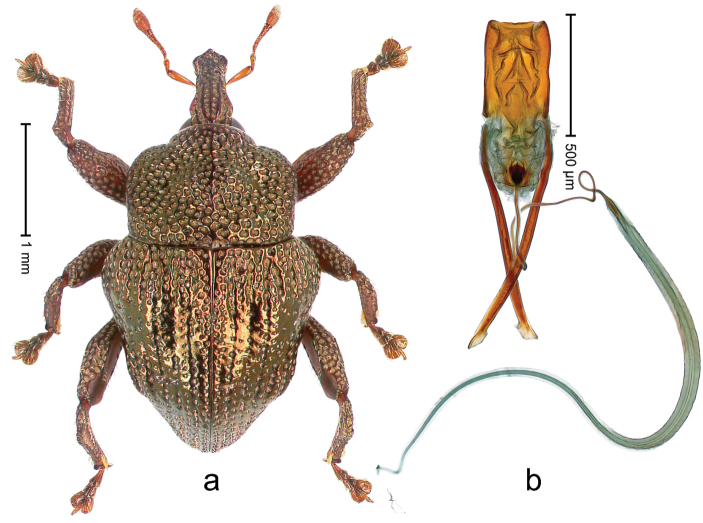
*Trigonopterus cupreus* Riedel, sp. n., holotype; **a** Habitus **b** Penis.

Holotype, male (Fig. 20a). Length 2.95 mm. Color of legs, head and ventral surface ferruginous; pronotum and elytron reddish coppery. Body in dorsal aspect with marked constriction between pronotum and elytron; in profile dorsally convex. Rostrum in apical half scabrous, coarsely punctate; in basal half with median and pair of submedian ridges; intervening furrows punctate, each with sparse row of erect setae; epistome with transverse, angulate ridge forming distinct median denticle. Pronotum with sides slightly diverging, anterolaterally rounded to subapical constriction; disk with pair of submedian impressions, coarsely punctate, reticulate; each puncture containing inconspicuous seta; with indistinct median ridge. Elytra with humeri swollen, laterally projecting; interval 5 and 6 behind middle swollen, gently projecting from lateral outline; striae indistinct; intervals flat, punctate; at base and apex densely coarsely punctate; transverse band between humeral and subapical swelling less densely punctate. Anteroventral ridge of femora distinct, in meso- and metafemur forming large tooth. Metafemur subapically with stridulatory patch. Dorsal edge of tibiae subbasally with angulation, in protibia with blunt tooth, in mesotibia with acute tooth, in metatibia denticulate. Abdominal ventrite 5 flat, coarsely punctate. Penis (Fig. 20b) with sides of body subparallel; apex subtruncate, weakly rounded; transfer apparatus compact; apodemes 2.4 × as long as body; ductus ejaculatorius with bulbus. **Intraspecific variation.** Length 2.43–3.05 mm. Color of pronotum bronze, reddish coppery, or greenish. Female rostrum dorsally with glabrous median costa bordered by rows of punctures and sublateral pair of furrows; epistome simple. Female pronotum ca. 1.1 × as wide as long, male pronotum ca. 1.3 × as wide as long. Female elytra subovate, with lateral contour convex to apex, humeri and intervals 5–6 simple.

##### Material examined.

Holotype (MZB): ARC1506 (EMBL # LM655604), West Nusa Tenggara Prov., Sumbawa, Batu Dulang, Mt. Batu Pasak, sample 2, S08°37.028', E117°15.783', 1305 m, 12-IV-2010. Paratypes (MZB, SMNK, ZSM): West Nusa Tenggara Prov., Sumbawa: 6 exx, ARC1505 (EMBL # LM655603), ARC1507 (EMBL # LM655605), ARC1508 (EMBL # LM655606), same data as holotype; 6 exx, Batu Dulang, Mt. Batu Pasak, sample 5, S08°37.005', E117°15.790', 1350 m, 18-IV-2010; 1 ex, ARC3589 (PCR failed), Tepal, Pc. Nengas, sample 7, S08°35.176', E117°08.295', 1490 m, 16-IV-2010.

##### Distribution.

West Nusa Tenggara Prov., Sumbawa (Batu Dulang, Tepal). Elevation: 1305–1350 m.

##### Etymology.

This epithet is based on the Latin adjective *cupreus* (copper-colored).

##### Notes.

*Trigonopterus cupreus* Riedel, sp. n. was coded as “*Trigonopterus* sp. 332”.

#### 21. Trigonopterus dacrycarpi

Taxon classificationAnimaliaColeopteraCurculionidae

Riedel sp. n.

http://zoobank.org/FDBF096E-8191-4C66-8DF8-5BF6F5F1BE01

##### Diagnostic description.

**Figure 21.**
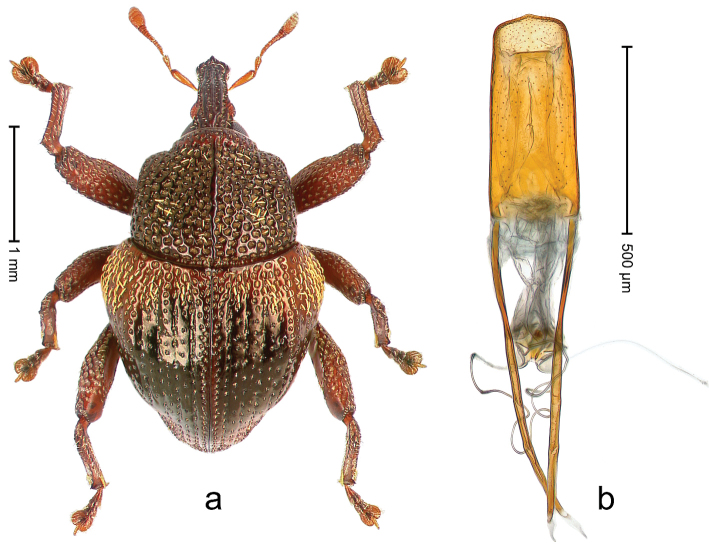
*Trigonopterus dacrycarpi* Riedel, sp. n., holotype; **a** Habitus **b** Penis.

Holotype, male (Fig. 21a). Length 2.88 mm. Color ferruginous; dorsal surface of head and pronotum dark ferruginous, center of elytron black with bronze lustre. Body in dorsal aspect with marked constriction between pronotum and elytron; in profile dorsally convex. Rostrum in apical third coarsely punctate; in basal half with median and pair of submedian ridges; intervening furrows punctate, each with sparse row of suberect setae; epistome posteriorly with transverse, angulate ridge forming distinct median denticle. Pronotum with sides subparallel, anteriorly abruptly rounded to subapical constriction; disk coarsely punctate, reticulate; each puncture containing inconspicuous seta; sublaterally each with two sparse clusters of recumbent, elongate, cream-colored scales; with distinct median ridge. Elytra with humeri swollen, laterally projecting; interval 5 in front of middle swollen, dorsally projecting; interval 6 simple; striae indistinct; intervals flat, punctate; subbasally coarsely punctate, with recumbent, yellowish, almond-shaped scales; center less densely punctate with punctures containing minute setae, interspaces polished. Anteroventral ridge of femora distinct, in meso- and metafemur forming large blunt tooth. Metafemur subapically with stridulatory patch. Dorsal edge of tibiae subbasally with angulation. Abdominal ventrite 5 flat, coarsely punctate, basally with rows of upcurved piliform scales. Penis (Fig. 21b) with sides of body subparallel; apex subangulate; transfer apparatus compact; apodemes 1.8 × as long as body; ductus ejaculatorius without bulbus. **Intraspecific variation.** Length 2.36–3.06 mm. Color of legs and elytral base ferruginous or dark ferruginous, almost black. Female body more slender. Female rostrum dorsally punctate-rugose, with median and pair of submedian subglabrous costae; epistome simple. Female elytra subovate, without swellings, lateral contour convex, humeri simple; scales near base indistinct.

##### Material examined.

Holotype (MZB): ARC1527 (EMBL # LM655625), West Nusa Tenggara Prov., Sumbawa, Batu Dulang, Mt. Batu Pasak, sample 3, S08°37.524', E117°15.423', 1385 m, 18-IV-2010. Paratypes (MZB, SMNK, ZSM): 2 exx, ARC1525 (EMBL # LM655623), ARC1526 (EMBL # LM655624), same data as holotype; 1 ex, Batu Dulang, Mt. Batu Pasak, sample 5, S08°37.005', E117°15.790', 1350 m, 18-IV-2010; 1 ex, ARC1532 (EMBL # LM655630), Sumbawa, Tepal, Pc. Nengas, sample 7, S08°35.176', E117°08.295', 1490 m, 16-IV-2010; 13 exx, ARC2200 (EMBL # LM655656), ARC2201 (EMBL # LM655657), ARC2202 (EMBL # LM655658), ARC2203 (EMBL # LM655659), Flores, Mt. Ranaka, sample 8, S08°37.781', E120°31.184', 1730 m, 10-III-2011; 1 ex, ARC2194 (EMBL # LM655650), Flores, Lake Ranamese, sample 3, S08°37.024', E120°33.400', 1270 m, 08-III-2011.

##### Distribution.

West Nusa Tenggara Prov., Sumbawa (Batu Dulang, Tepal), East Nusa Tenggara Prov., Flores (Mt. Ranaka, L. Ranamese). Elevation: 1270–1730 m.

##### Etymology.

This epithet is based on the name of *Dacrycarpus* trees under which this weevil was usually sifted – it may however feed on other plants occurring in the same habitat.

##### Notes.

*Trigonopterus dacrycarpi* Riedel, sp. n. was coded as “*Trigonopterus* sp. 329”. Cox1 sequences of the populations from Sumbawa and Flores differ 7.5–9.7% *p*-distance, but morphologically no differences could be found.

#### 22. Trigonopterus delapan

Taxon classificationAnimaliaColeopteraCurculionidae

Riedel sp. n.

http://zoobank.org/59669DF0-410D-4A9D-B8AF-239E93651713

##### Diagnostic description.

**Figure 22.**
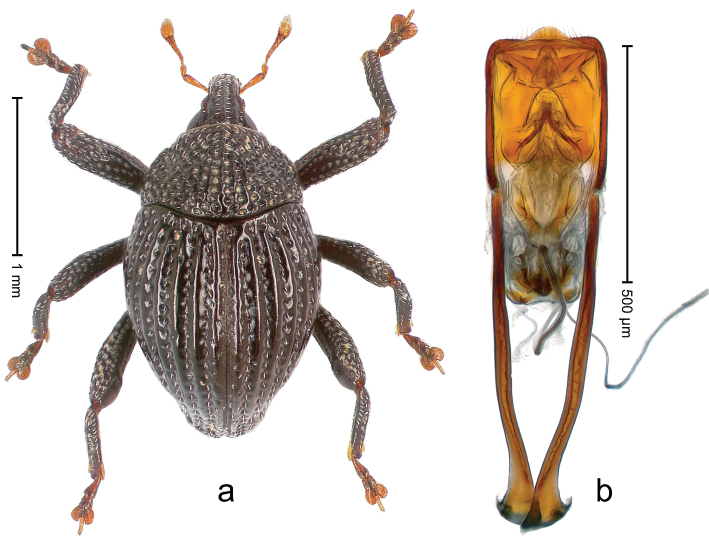
*Trigonopterus delapan* Riedel, sp. n., holotype; **a** Habitus **b** Penis.

Holotype, male (Fig. 22a). Length 2.04 mm. Color of antennae and tarsi ferruginous; remainder black. Body subovate, in dorsal aspect and in profile with weak constriction between pronotum and elytron. Rostrum with median ridge and pair of indistinct submedian ridges, intervening furrows each with sparse row of mesad directed setae; anterior third scabrous; epistome with transverse ridge. Pronotum coarsely punctate, laterally reticulate, submedially interspaces longitudinally rugose, with median costa; with sparse, suberect, slender scales. Elytra with striae deeply impressed; each with row of slender suberect setae; suture broadly sunk-in, dull microreticulate; intervals costate, subglabrous. Femora with simple, crenate anteroventral ridge. Metafemur subapically with stridulatory patch. Metatibia apically with uncus, without premucro. Abdominal ventrite 5 sparsely punctate, microreticulate, in basal third with median carina. Penis (Fig. 22b) with sides of body subparallel; containing sclerite of an inverted V; apex sparsely setose, with median, subtriangular extension; transfer apparatus compact, symmetrical; transfer process spiniform, pointing to apex of endophallus; apodemes 2.6 × as long as body; ductus ejaculatorius without bulbus. **Intraspecific variation.** Length 2.04–2.08 mm. Color of body dark ferruginous to black. Female rostrum in apical half dorsally subglabrous, punctate; epistome simple. Female abdominal ventrite 5 near base weakly swollen.

##### Material examined.

Holotype (MZB): ARC2242 (EMBL # LM655697), East Nusa Tenggara Prov., Flores Isl., Labuhan Bajo, Tebedo, sample 1, S08°29.848', E119°59.633', 525 m, 14-III-2011. Paratypes (MZB, SMNK): 7 exx, ARC2591 (EMBL # LM655900), ARC2592 (EMBL # LM655901), ARC3636 (EMBL # LM656058), same data as holotype.

##### Distribution.

East Nusa Tenggara Prov., Flores (Labuhan Bajo). Elevation: 525 m.

##### Etymology.

This epithet is based on the Indonesian word for “eight” and is treated as a noun in apposition.

##### Notes.

*Trigonopterus delapan* Riedel, sp. n. was coded as “*Trigonopterus* sp. 298” by Tänzler et al. (2014).

#### 23. Trigonopterus dentipes

Taxon classificationAnimaliaColeopteraCurculionidae

Riedel sp. n.

http://zoobank.org/550B7704-235F-40E4-B006-49B32D239AF6

##### Diagnostic description.

**Figure 23.**
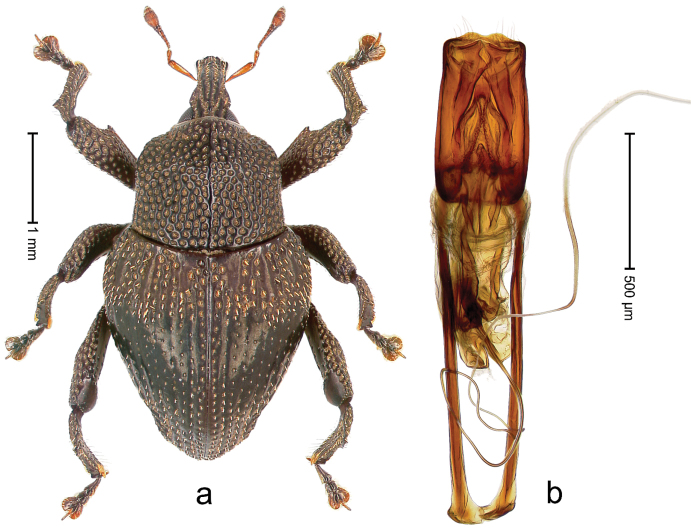
*Trigonopterus dentipes* Riedel, sp. n., holotype; **a** Habitus **b** Penis.

Holotype, male (Fig. 23a). Length 3.84 mm. Color of antennae ferruginous; basal third of elytra and legs dark ferruginous; remainder black. Body in dorsal aspect with marked constriction between pronotum and elytron; in profile dorsally flat, apically convex. Rostrum with median and pair of submedian ridges; intervening furrows each with sparse row of erect piliform scales; epistome with transverse ridge forming submedian pair of small denticles. Pronotum with sides subparallel, rounded to indistinct subapical constriction; disk densely punctate, reticulate; with indistinct median ridge; with swelling in anterior half. Elytra with striae near base and apex distinct; intervals partly swollen or with coarse punctures each bearing small, suberect, cream-colored scale; transverse band at middle with indistinct striae, intervals subglabrous, sparsely punctate; apex subangulate. Anteroventral ridge of femora forming large tooth. Metafemur subapically with stridulatory patch. Dorsal edge of tibiae with subbasal angulation dentate. Abdominal ventrites 1–2 concave, subglabrous; abdominal ventrite 5 flat, coarsely punctate, with sparse erect piliform scales. Penis (Fig. 23b) with sides of body subparallel; apex bisinuate, with median notch; transfer apparatus compact, symmetrical, but slightly tilted to the left; apodemes 2.5 × as long as body; ductus ejaculatorius without bulbus. **Intraspecific variation.** Length 3.04–4.04 mm. Coloration of elytral base ranging from orange-ferruginous to dark ferruginous and almost black. Female rostrum with median and pair of submedian glabrous costae; epistome simple. Female elytra more slender, with lateral contour convex to apex; punctures sparser and smaller than in male; male elytra relatively broad between humeri, converging in almost straight line to apex.

##### Material examined.

Holotype (MZB): ARC1468 (EMBL # LM655566), West Nusa Tenggara Prov., Lombok, Tetebatu, Rinjani-trail from Orong Gerisak, sample 7, S08°29.433', E116°24.746', 1240 m, 04-IV-2010 (MZB). Paratypes (ARC, MZB, SMNK, ZSM): Lombok: 4 exx, ARC0163, N Tetebatu, 1200-1450 m, 07-XII-2004; 1 ex, ARC1461 (EMBL # LM655559), Tetebatu, Rinjani-trail from Orong Gerisak, sample 6, S08°29.577', E116°24.782', 1195 m, 04-IV-2010; 2 exx, ARC1474 (EMBL # LM655572), ARC1475 (EMBL # LM655573), Tetebatu, Rinjani-trail from Orong Gerisak, sample 5, S08°30.096', E116°25.062', 1010 m, 04-IV-2010; 1 ex, ARC2270 (EMBL # LM655724), Senaru, Rinjani-track, sample 6, S08°19.359', E116°24.070', 860 m, 23-III-2011; 4 exx, ARC2271 (EMBL # LM655725), ARC2272 (EMBL # LM655726), ARC2273 (EMBL # LM655727), Santong, Rinjani-track, sample 1, S08°20.715', E116°19.695', 830 m, 24-III-2011; 1 ex, Santong, Rinjani-track, sample 2, S08°20.813', E116°19.778', 870 m, 24-III-2011; 1 ex, ARC2283 (EMBL # LM655737), Santong, Rinjani-track, sample 3, S08°20.996', E116°20.001', 960 m, 25-III-2011; 2 exx, ARC2284 (EMBL # LM655738), Santong, Rinjani-track, sample 4, S08°21.160', E116°20.067', 1005 m, 25-III-2011.

##### Distribution.

West Nusa Tenggara Prov., Lombok (Santong, Senaru, Tetebatu). Elevation: 830–1240 m.

##### Etymology.

This epithet is based on the combination of the Latin nouns *dens* (tooth) and *pes* (foot).

##### Notes.

*Trigonopterus dentipes* Riedel, sp. n. was coded as “*Trigonopterus* sp. 322” by Tänzler et al. (2014).

#### 24. Trigonopterus diengensis

Taxon classificationAnimaliaColeopteraCurculionidae

Riedel sp. n.

http://zoobank.org/E14C9035-0C32-49B0-BAA5-F23715A72CF6

##### Diagnostic description.

**Figure 24.**
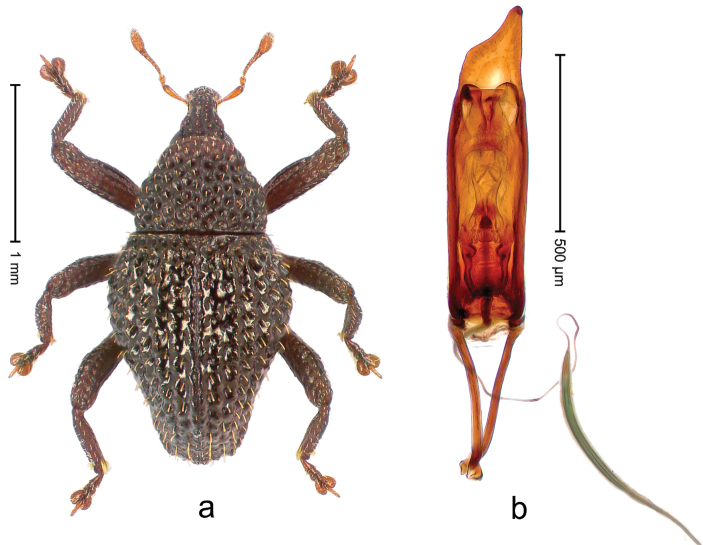
*Trigonopterus diengensis* Riedel, sp. n., holotype; **a** Habitus **b** Penis.

Holotype, male (Fig. 24a). Length 2.14 mm. Color of antennae and legs ferruginous; remainder black. Body subovate; in dorsal aspect and in profile with distinct constriction between pronotum and elytron. Rostrum in apical half rugose-punctate, in basal half with median ridge and pair of submedian ridges; with sparse suberect scales; epistome simple. Pronotum with shallow subapical constriction; disk coarsely punctate, scabrous; each puncture containing suberect spatulate scale inserting at posterior rim. Elytra with striae marked by deep punctures; in front of each puncture with elongate-claviform, erect scale; intervals tuberculate, partly transversely corrugate, subglabrous; interval 7 subapically costate; sutural interval more distinctly raised. Meso- and metafemur ventrally weakly dentate. Metafemur subapically without stridulatory patch. Abdominal ventrite 5 flat, with sparse transverse rows of setae. Penis (Fig. 24b) with apex asymmetrically extended to the left; apical extension of medium-size; basal orifice ventrally with rim; apodemes short, 0.5 × as long as body; ductus ejaculatorius with bulbus. **Intraspecific variation.** Length 1.92–2.36 mm. Female rostrum dorsally in apical half subglabrous, densely punctate; epistome simple.

##### Material examined.

Holotype (MZB): ARC0290 (EMBL # LM655480), C-Java Prov., N slopes of Dieng figau, Petungkriyono, mountain N Tinalum, sample 1, S07°06.418', E109°44.514', 1115 m, 22-VIII-2006. Paratypes (MZB, SMNK, ZSM): 13 exx, ARC0288 (EMBL # LM655478), ARC0289 (EMBL # LM655479), ARC0291 (EMBL # LM655481), ARC0292 (EMBL # LM655482), same data as holotype.

##### Distribution.

C-Java Prov. (Dieng). Elevation: 1115 m.

##### Etymology.

This epithet is based on the type locality.

##### Notes.

*Trigonopterus diengensis* Riedel, sp. n. was coded as “*Trigonopterus* sp. 335”.

#### 25. Trigonopterus dimorphus

Taxon classificationAnimaliaColeopteraCurculionidae

Riedel sp. n.

http://zoobank.org/65E77834-0FD3-4A27-9EE0-E7DA25BB9594

##### Diagnostic description.

**Figure 25.**
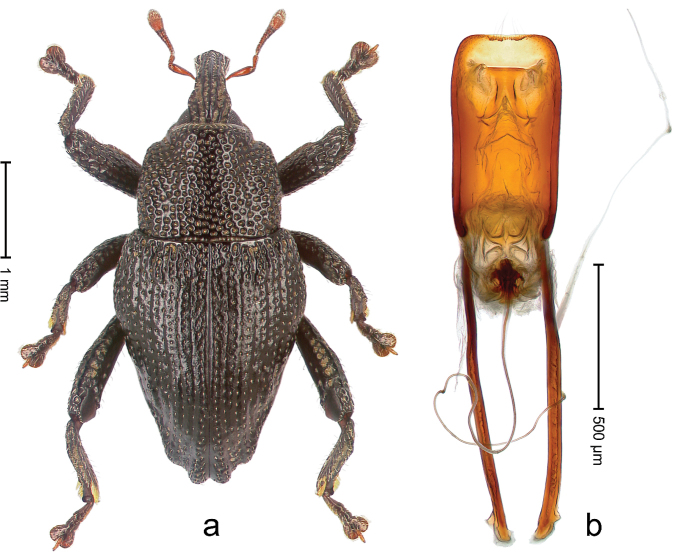
*Trigonopterus dimorphus* Riedel, sp. n., holotype; **a** Habitus **b** Penis.

Holotype, male (Fig. 25a). Length 3.80 mm. Color of tarsi, tibiae and antennae ferruginous, remainder black. Body elongate; in dorsal aspect with marked constriction between pronotum and elytron; in profile dorsally evenly convex. Rostrum with median carina terminating on forehead and pair of submedian ridges; intervening furrows each with row of erect piliform scales; epistome with transverse, angulate ridge. Pronotum anterolaterally projecting, rounded; with subapical constriction; disk densely punctate, interspaces subglabrous; each puncture containing recumbent seta; disk with pair of submedian, shallow impressions. Elytra with striae weakly impressed, partly indistinct; intervals flat; punctation confused, more dense towards base, near basal margin punctures partly confluent; each puncture containing small recumbent seta; interspaces microreticulate; interval 7 swollen subapically, laterally weakly projecting; sutural interval at apex markedly swollen, forming pair of rounded apical protrusions. Anteroventral ridge of femora forming blunt tooth. Metafemur subapically with stridulatory patch. Abdominal ventrite 5 with broad impression, densely punctate, setose with erect setae. Penis (Fig. 25b) with sides of body subparallel; apex broadly rounded; transfer apparatus distinct, compact; apodemes 1.6 × as long as body; ductus ejaculatorius without bulbus. **Intraspecific variation.** Length 2.94–3.80 mm. Integument of males dull, microreticulate; females with punctures sparser and smaller, interspaces polished. Female rostrum dorsally with glabrous median costa bordered by submedian and sublateral pairs of furrows; epistome simple. Female elytra with lateral contour evenly convex to apex; male elytra with lateral contour at humeri subangulate, in straight line to apex. Female abdominal ventrite 5 flat.

##### Material examined.

Holotype (MZB): ARC0211 (EMBL # LM655433), West Java Prov., Mt. Halimun N.P., Citalahab, sample 2, S06°44.503', E106°31.550', 1200 m, 12-IX-2005. Paratypes (ARC, MZB, SMNK): W-Java Prov., Mt. Halimun N.P.: 3 exx, ARC0101, W-Java Prov., Mt. Halimun N.P., Citalahab, S06°44.503', E106°31.550', 1150 m, 20-21-IX-2004; 10 exx, ARC0212 (EMBL # LM655434), ARC0213 (EMBL # LM655435), same data as holotype; 1 ex, Citalahab, sample 1, S06°44.423', E106°31.738', 1100 m, 12-IX-2005; 2 exx, ARC0170, N Ciptarasa, sample 3, S06°49.867', E106°30.085', 1100 m, 19-IX-2005; 2 exx, ARC0216 (EMBL # LM655438), ARC0217 (EMBL # LM655439), Panguyangan, Mt. Talaga, ca.1000 m, 17-IX-2005.

##### Distribution.

W-Java Prov. (Mt. Halimun N.P.). Elevation: 1000–1200 m.

##### Etymology.

This epithet is a combination of the prefix *di*- (two, double) and the adjective derived from the Greek *morphe* (gestalt).

##### Notes.

*Trigonopterus dimorphus* Riedel, sp. n. was coded as “*Trigonopterus* sp. 293”.

#### 26. Trigonopterus disruptus

Taxon classificationAnimaliaColeopteraCurculionidae

Riedel sp. n.

http://zoobank.org/0F1402DF-2CDC-4601-9037-0D1B81665305

##### Diagnostic description.

**Figure 26.**
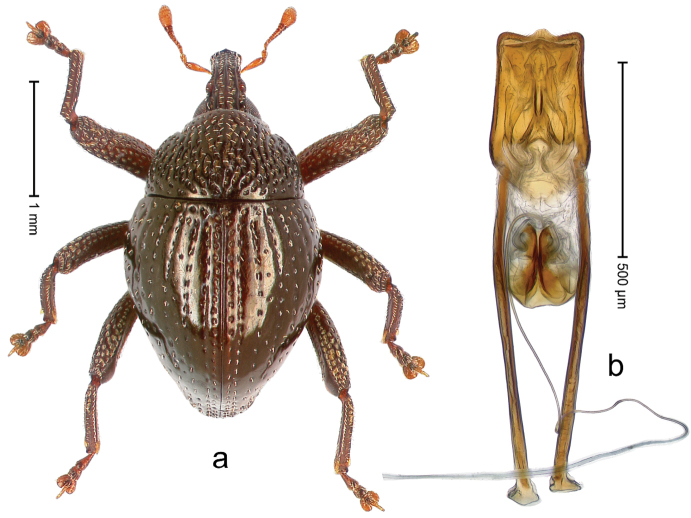
*Trigonopterus disruptus* Riedel, sp. n., holotype; **a** Habitus **b** Penis.

Holotype, male (Fig. 26a). Length 2.78 mm. Color of antennae light ferruginous, legs dark ferruginous; remainder black. Body subovate, in dorsal aspect with weak constriction between pronotum and elytron; profile dorsally convex. Rostrum with median ridge and pair of submedian ridges, intervening furrows each with row of coarse punctures and sparse row of mesad directed setae; epistome with transverse subangulate ridge. Pronotum dorsally longitudinally rugose, with median costa; furrows containing sparse rows of mesad directed slender scales; laterally coarsely punctate. Elytra with striae distinct, marked by rows of punctures; each containing small white bristle, partly abraded; laterally punctures larger; intervals flat, subglabrous. Femora with simple anteroventral ridge. Metafemur subapically with stridulatory patch. Abdominal ventrite 5 at middle with shallow, densely punctate impression, with suberect scales. Penis (Fig. 26b) with sides of body slightly converging; apex subangulate, with median, subtriangular extension; dorsally behind orifice with distinct median carina, projecting convex in profile; transfer apparatus with pair of elongate, crescent-shaped sclerites; apodemes 2.8 × as long as body; ductus ejaculatorius without bulbus. **Intraspecific variation.** Length 2.40–2.95 mm. Color of elytra with more or less distinct bronze lustre. Female rostrum in apical half dorsally subglabrous, with submedian row of minute punctures; epistome simple. Elytra with striae as in holotype, or punctures of striae subbasally corrugate (two specimens from Tetebatu), or striae markedly impressed and intervals costate (two specimens from Senaru). Female abdominal ventrite 5 flat.

##### Material examined.

Holotype (MZB): ARC1490 (EMBL # LM655588), West Nusa Tenggara Prov., Lombok, road between Sembalun and Sapit, sample 5, S08°26.453', E116°31.859', 1195 m, 31-III-2010. Paratypes (MZB, SMNK, ZSM): West Nusa Tenggara Prov., Lombok: 1 ex, ARC1491 (EMBL # LM655589), same data as holotype; 1 ex, ARC1486 (EMBL # LM655584), road between Sembalun and Sapit, sample 1, S08°25.110', E116°31.947', 1450 m, 30-III-2010; 2 exx, ARC1488 (EMBL # LM655586), road between Sembalun and Sapit, sample 4, S08°25.331', E116°31.841', 1405 m, 31-III-2010; 1 ex, ARC1460 (EMBL # LM655558), Tetebatu, Rinjani-trail from Orong Gerisak, sample 6, S08°29.577', E116°24.782', 1195 m, 04-IV-2010; 5 exx, ARC0164, Tetebatu, Rinjani-trail, 1200-1450 m, 07-XII-2004; 2 exx, ARC1466 (EMBL # LM655564), ARC1467 (EMBL # LM655565), sample 7, S08°29.433', E116°24.746', 1240 m, 04-IV-2010; 1 ex, ARC1485 (EMBL # LM655583), Sesaot, Rinjani-trail, sample 3, S08°29.841', E116°13.679', 625 m, 28-III-2010; 1 ex, ARC2265 (EMBL # LM655719), Senaru, Rinjani-trail, sample 4, S08°20.439', E116°24.047', 1240 m, 21-III-2011; 1 ex, ARC2266 (EMBL # LM655720), Senaru, Rinjani-trail, sample 5, S08°19.800', E116°24.107', 1015 m, 21-III-2011; 2 exx, Lombok, Senaru, Rinjani-track, sample 6, S08°19.359', E116°24.070', 860 m, 23-III-2011; 5 exx, ARC2269 (EMBL # LM655723), Senaru, Rinjani-trail, sample 7, S08°19.643', E116°24.033', 935 m, 23-III-2011; 7 exx, Senaru, Rinjani-trail, sample 8, S08°19.719', E116°24.040', 955 m, 23-III-2011; 7 exx, ARC2275 (EMBL # LM655729), ARC2276 (EMBL # LM655730), Santong, Rinjani-trail, sample 1, S08°20.715', E116°19.695', 830 m, 24-III-2011; 2 exx, Santong, Rinjani-track, sample 2, S08°20.813', E116°19.778', 870 m, 24-III-2011; 2 exx, Santong, Rinjani-track, sample 3, S08°20.996', E116°20.001', 960 m, 24-III-2011; 2 exx, Santong, Rinjani-track, sample 4, S08°21.160', E116°20.067', 1005 m, 25-III-2011; 1 ex, Santong, Rinjani-track, sample 6, S08°21.754', E116°20.476', 1315 m, 25-III-2011; 2 exx, ARC2286 (EMBL # LM655740), ARC2287 (EMBL # LM655741), Sajang, sample 1, S08°19.370', E116°29.570', 845 m, 26-III-2011; 4 exx, Mt. Rinjani, Bawnau, near Sembalun, sample 1, S08°21.136', E116°29.257', 1140 m, 26-III-2011; 3 exx, Mt. Rinjani, Bawnau, near Sembalun, S08°21.136', E116°29.257', 1140 m, collected from foliage, 26-III-2011; 2 exx, ARC2291 (EMBL # LM655745), ARC2292 (EMBL # LM655746), Sajang, sample 1, S08°21.136', E116°29.257', 1140 m, 26-III-2011; 4 exx, Mt. Pengasingan, SE Sembalun, sample 2, S08°19.354', E116°30.530', 945 m, 27-III-2011; 1 ex, Mt. Pengasingan, SE Sembalun, sample 3, S08°19.445', E116°30.603', 1005 m, 27-III-2011; 4 exx, ARC2299 (EMBL # LM655753), ARC2300 (EMBL # LM655754), Gn. Pengasingan, SE Sembalun, sample 4, S08°19.504', E116°30.767', 1035 m, 27-III-2011.

##### Distribution.

West Nusa Tenggara Prov., Lombok (Mt. Pengasingan, Sajang, Santong, Sembalun, Senaru, Sesaot, Tetebatu). Elevation: 625–1450 m.

##### Etymology.

This epithet is based on the Latin participle *disruptus* (broken apart) and refers to its phylogeography which appears to reflect the division of the species in two populations.

##### Notes.

*Trigonopterus disruptus* Riedel, sp. n. was coded as “*Trigonopterus* sp. 292” by Tänzler et al. (2014).

#### 27. Trigonopterus dua

Taxon classificationAnimaliaColeopteraCurculionidae

Riedel sp. n.

http://zoobank.org/CF312224-7D32-4057-A0D3-0238FF8F7478

##### Diagnostic description.

**Figure 27.**
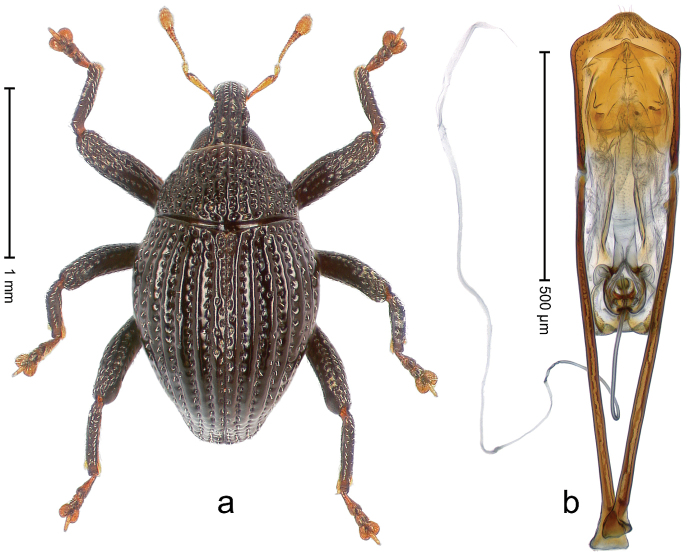
*Trigonopterus dua* Riedel, sp. n., holotype; **a** Habitus **b** Penis.

Holotype, male (Fig. 27a). Length 1.82 mm. Color of antennae and tarsi ferruginous; remainder black. Body subovate, in dorsal aspect and in profile with weak constriction between pronotum and elytron. Rostrum with median ridge and pair of indistinct submedian ridges, intervening furrows each with sparse row of mesad directed setae; epistome simple. Pronotum coarsely punctate, rugose, with median costa; with sparse, slender, subrecumbent scales. Elytra with striae deeply impressed, each with row of short suberect scales; intervals costate, subglabrous; in basal half incised suture bordered by row of punctures. Femora with simple, crenate anteroventral ridge. Metafemur subapically with stridulatory patch. Metatibia apically with slender, slightly posteriad curved uncus; with premucro. Abdominal ventrite 5 flat, coarsely punctate. Penis (Fig. 27b) with sides of body subparallel; apical 1/5 broadly angulate, subglabrous, medially rounded and sparsely setose; transfer apparatus compact, symmetrical; apodemes 2.6 × as long as body; ductus ejaculatorius without bulbus. **Intraspecific variation.** Length 1.82–1.88 mm. Female rostrum in apical half dorsally subglabrous, sparsely punctate.

##### Material examined.

Holotype (MZB): ARC2232 (EMBL # LM655688), East Nusa Tenggara Prov., Flores Isl., Labuhan Bajo, Roe, sample 3, S08°36.259', E120°01.539', 975 m, 13-III-2011. Paratypes (MZB, SMNK, ZSM): Flores Isl., Labuhan Bajo: 1 ex, ARC2217 (EMBL # LM655673), Roe, sample 1, S08°36.540', E120°01.871', 955 m, earthworm-dominated habitat, 13-III-2011; 2 exx, ARC2589 (EMBL # LM655898), ARC3626 (EMBL # LM656056), Roe, sample 3, S08°36.259', E120°01.539', 975 m, 13-III-2011; 1 ex, ARC2226 (EMBL # LM655682), Roe, sample 5, S08°35.395', E120°00.383', 790 m, 13-III-2011; 1 ex, same data as holotype; 3 exx, ARC2244 (EMBL # LM655699), ARC2593 (EMBL # LM655902), ARC2594 (EMBL # LM655903), Tebedo, sample 1, S08°29.848', E119°59.633', 525 m, 14-III-2011.

##### Distribution.

East Nusa Tenggara Prov., Flores (Labuhan Bajo). Elevation: 525–975 m.

##### Etymology.

This epithet is based on the Indonesian word for “two” and is treated as a noun in apposition.

##### Notes.

*Trigonopterus dua* Riedel, sp. n. was coded as “*Trigonopterus* sp. 284”.

#### 28. Trigonopterus duabelas

Taxon classificationAnimaliaColeopteraCurculionidae

Riedel sp. n.

http://zoobank.org/5BE6A9EF-9228-4BF8-9EAF-B8B9DB1692A5

##### Diagnostic description.

**Figure 28.**
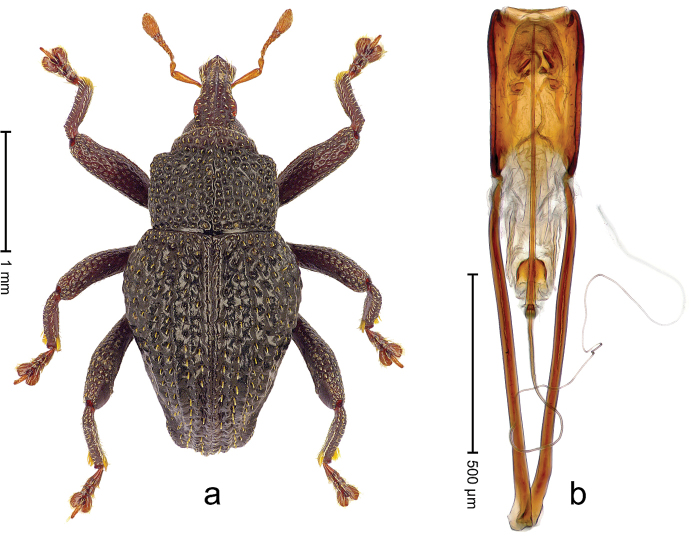
*Trigonopterus duabelas* Riedel, sp. n., holotype; **a** Habitus **b** Penis.

Holotype, male (Fig. 28a). Length 2.80 mm. Color of tarsi and antenna ferruginous, remainder black. Body elongate; in dorsal aspect with marked constriction between pronotum and elytron; with distinct constriction in profile. Rostrum dorsally microreticulate, dull; with median ridge and pair of indistinct submedian ridges; intervening furrows each with row of erect scales; epistome with transverse, angulate ridge forming distinct median denticle. Pronotum anterolaterally angularly projecting; with distinct subapical constriction; disk coarsely punctate, punctures each with one erect seta; interspaces microreticulate; with median ridge. Elytra with striae deeply impressed, each with row of suberect scales; intervals irregularly costate, partly tuberculate, nude, weakly microreticulate; interval 7 swollen subapically, laterally weakly projecting; sutural interval subapically with minute tooth. Femora edentate. Metafemur subapically with stridulatory patch and transverse ridge. Abdominal ventrite 5 densely punctate, setose, in apical half with impression. Penis (Fig. 28b) in profile markedly curved; in dorsal aspect body slender, sides subparallel; ventral edge of apex retracted, with median extension small, dentiform; transfer apparatus flagelliform, 1.9 × as long as body; apodemes 2.2 × as long as body; ductus ejaculatorius without bulbus. **Intraspecific variation.** Length 2.2–2.8 mm. Color of legs entirely ferruginous in specimens from Mt. Slamet; sutural interval ferruginous or black. Female rostrum dorsally with pairs of lateral and submedian furrows; epistome simple. Elytral interval 3 more or less swollen. Female abdominal ventrite 5 flat. Penis with flagelliform transfer apparatus 1.7–1.9 × longer than body.

##### Material examined.

Holotype (MZB): ARC0284 (EMBL # LM655474), C-Java Prov., N slopes of Dieng figau, Petungkriyono, Mt. Deles, sample 1, S07°08.221', E109°43.599', 1505 m, 24-VIII-2006. Paratypes (MZB, SMNK): C-Java Prov.: ARC0283 (EMBL # LM655473), same data as holotype; 2 exx, ARC2491 (EMBL # LM655828), ARC2492 (EMBL # LM655829), Mt. Slamet, Guci, sample 2, S07°13.255', E109°11.005', 1858 m, 26-XI-2011; 4 exx, ARC2493 (EMBL # LM655830), ARC2494 (EMBL # LM655831), ARC2495 (EMBL # LM655832), Mt. Slamet, Guci, sample 6, S07°11.983', E109°10.556', 1671 m, 27-XI-2011; 1 ex, Mt. Slamet, Guci, sample 7, S07°11.906', E109°10.486', 1653 m, 27-XI-2011.

##### Distribution.

C-Java Prov. (Dieng, Mt. Slamet). Elevation: 1505–1858 m.

##### Etymology.

This epithet is based on the Indonesian word for “twelve” and is treated as a noun in apposition.

##### Notes.

*Trigonopterus duabelas* Riedel, sp. n. was coded as “*Trigonopterus* sp. 444”. Cox1 sequences of the populations from Dieng and Mt. Slamet differ 4.6–5.4 % *p*-distance, but no distinct morphological differences could be found.

#### 29. Trigonopterus echinatus

Taxon classificationAnimaliaColeopteraCurculionidae

Riedel sp. n.

http://zoobank.org/354FC9B5-0CC9-416F-8679-4A7860911EF4

##### Diagnostic description.

**Figure 29.**
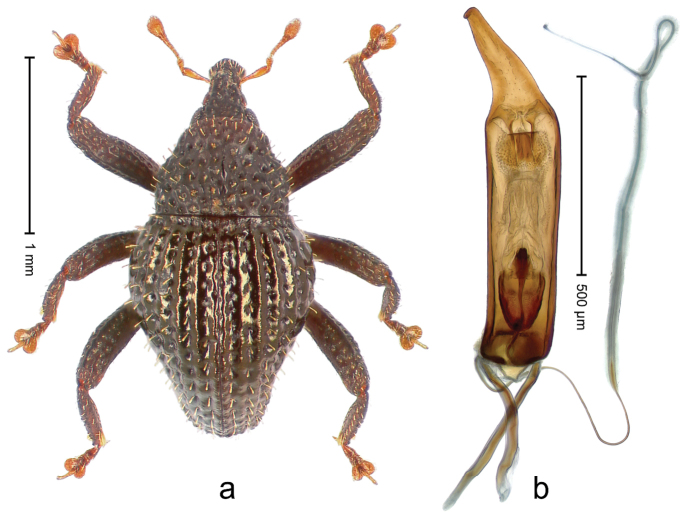
*Trigonopterus echinatus* Riedel, sp. n., holotype; **a** Habitus **b** Penis.

Holotype, male (Fig. 29a). Length 1.88 mm. Color of legs and head ferruginous; remainder black, pronotum dull, elytra with bronze lustre. Body subovate; in dorsal aspect and in profile with distinct constriction between pronotum and elytron. Rostrum in apical half granulate-punctate, in basal half with irregular median ridge and pair of submedian ridges; with sparse suberect scales; epistome with transverse, angulate ridge. Pronotum with shallow subapical constriction; disk coarsely punctate; each puncture containing erect clavate scale inserting at posterior rim; with short median ridge. Elytra with striae marked by deep punctures; in front of each puncture with elongate-claviform, erect scale; intervals weakly costate, subglabrous; interval 7 subapically forming denticulate ridge; sutural interval simple. Femora edentate. Metafemur subapically without stridulatory patch. Abdominal ventrite 5 flat, microreticulate, sparsely setose. Penis (Fig. 29b) with apex asymmetrically extended to the right; apical extension long; basal orifice ventrally without rim; apodemes short, 0.5 × as long as body; ductus ejaculatorius with bulbus. **Intraspecific variation.** Length 1.72–2.04 mm. Color of elytra with bronze lustre more or less distinct. Female rostrum dorsally in apical half subglabrous, with small punctures; epistome simple.

##### Material examined.

Holotype (MZB): ARC0204 (EMBL # LM655426), W-Java Prov., Ciamis, Mt. Sawal, Batu Cakra, sample 1, S07°14.920', E108°15.762', 990 m, 01-X-2005. Paratypes (MZB, SMNK): W-Java Prov.: 1 ex, ARC2503 (EMBL # LM655840), Ciamis, Panjalu, Tembong, Mt. Sawal, sample 1, S07°10.528', E108°16.423', 1314 m, sifted; 8 exx, ARC2696 (EMBL # LM655952), ARC2697 (EMBL # LM655953), ARC2698 (EMBL # LM655954), Garut, Cikajang, Mt. Payung, sample 1, S07°25.345', E107°48.825', 1085 m, 26-IV-2012.

##### Distribution.

W-Java Prov. (Mt. Payung, Mt. Sawal). Elevation: 990–1314 m.

##### Etymology.

This epithet is based on the Latin adjective *echinatus* (prickly) and refers to the species´ erect scales that resemble spines.

##### Notes.

*Trigonopterus echinatus* Riedel, sp. n. was coded as “*Trigonopterus* sp. 337”.

#### 30. Trigonopterus empat

Taxon classificationAnimaliaColeopteraCurculionidae

Riedel sp. n.

http://zoobank.org/D9101D8E-AE49-4C01-8DB7-53ABA8170CB1

##### Diagnostic description.

**Figure 30.**
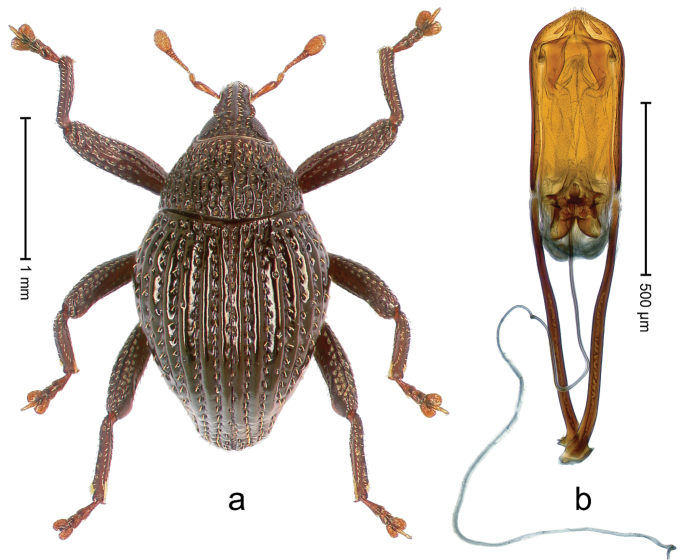
*Trigonopterus empat* Riedel, sp. n., holotype; **a** Habitus **b** Penis.

Holotype, male (Fig. 30a). Length 2.24 mm. Color of antennae and tarsi ferruginous; remainder dark ferruginous to black; elytra with slight bronze lustre. Body subovate, in dorsal aspect and in profile with weak constriction between pronotum and elytron. Rostrum with median ridge and pair of submedian ridges, intervening furrows each with sparse row of mesad directed scales; epistome with transverse subangulate ridge. Pronotum coarsely punctate, interspaces longitudinally rugose; with median costa; with sparse, suberect scales. Elytra with striae deeply impressed, each with row of slender suberect scales; intervals costate, subglabrous; in basal half incised suture bordered by row of punctures. Femora with simple, crenate anteroventral ridge. Metafemur subapically with stridulatory patch. Metatibia apically with uncus, without premucro. Abdominal ventrite 5 flat, coarsely punctate. Penis (Fig. 30b) with sides of body subparallel; apex broadly angulate, medially rounded and sparsely setose; in apical third with pair of elongate sclerites converging to orifice; transfer apparatus compact, symmetrical; apodemes 1.8 × as long as body; ductus ejaculatorius without bulbus. **Intraspecific variation.** Length 2.18–2.24 mm. Female unknown.

##### Material examined.

Holotype (MZB): ARC2192 (EMBL # LM655648), East Nusa Tenggara Prov., Flores Isl., Ruteng, Danau Ranamese, sample 1, S08°37.705', E120°33.702', 1320 m, 08-III-2011. Paratypes (SMNK): Flores Isl., Ruteng, Mt. Ranaka: 1 ex, ARC2583 (EMBL # LM655892), sample 9, S08°37.641', E120°31.204', 1685 m, 10-III-2011.

##### Distribution.

East Nusa Tenggara Prov., Flores (Ruteng). Elevation: 1320–1685 m.

##### Etymology.

This epithet is based on the Indonesian word for “four” and is treated as a noun in apposition.

##### Notes.

*Trigonopterus empat* Riedel, sp. n. was coded as “*Trigonopterus* sp. 303” by Tänzler et al. (2014).

#### 31. Trigonopterus enam

Taxon classificationAnimaliaColeopteraCurculionidae

Riedel sp. n.

http://zoobank.org/F541E096-1E30-4C72-A211-36F357CEE881

##### Diagnostic description.

**Figure 31.**
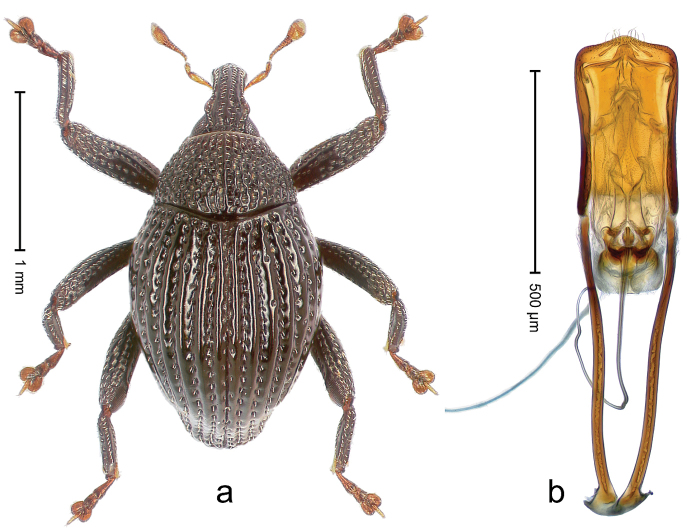
*Trigonopterus enam* Riedel, sp. n., holotype; **a** Habitus **b** Penis.

Holotype, male (Fig. 31a). Length 2.06 mm. Color of antennae, tarsi and tibiae ferruginous; remainder black. Body subovate, in dorsal aspect and in profile with weak constriction between pronotum and elytron. Rostrum with median ridge and pair of indistinct submedian ridges, intervening furrows each with sparse row of mesad directed setae; epistome with transverse subangulate ridge. Pronotum coarsely punctate, rugose, with median costa; with sparse, subrecumbent scales. Elytra with striae deeply impressed, each with row of slender suberect scales; intervals costate, subglabrous; incised suture bordered by row of punctures. Femora with simple, crenate anteroventral ridge. Metafemur subapically with stridulatory patch. Metatibia apically with uncus, without premucro. Abdominal ventrite 5 coarsely punctate. Penis (Fig. 31b) with sides of body subparallel; apex broadly angulate, medially rounded and sparsely setose; transfer apparatus compact, symmetrical; apodemes 1.9 × as long as body; ductus ejaculatorius without bulbus. **Intraspecific variation.** Length 2.06–2.24 mm. Color of body partly ferruginous or black. Female rostrum in apical half dorsally subglabrous, punctate; epistome simple.

##### Material examined.

Holotype (MZB): ARC2224 (EMBL # LM655680), East Nusa Tenggara Prov., Flores Isl., Labuhan Bajo, Roe, sample 5, S08°35.395', E120°00.383', 790 m, 13-III-2011 (MZB). Paratypes (SMNK, ZSM): Flores Isl., Labuhan Bajo: 2 exx, ARC2587 (EMBL # LM655896), ARC2588 (EMBL # LM655897), Roe, sample 2, S08°36.338', E120°01.637', 965 m, 13-III-2011; 1 ex, ARC3625 (EMBL # LM656055), Roe, sample 3, S08°36.259', E120°01.539', 975 m, 13-III-2011.

##### Distribution.

East Nusa Tenggara Prov., Flores (Labuhan Bajo). Elevation: 790–965 m.

##### Etymology.

This epithet is based on the Indonesian word for “six” and is treated as a noun in apposition.

##### Notes.

*Trigonopterus enam* Riedel, sp. n. was coded as “*Trigonopterus* sp. 295” by Tänzler et al. (2014).

#### 32. Trigonopterus fissitarsis

Taxon classificationAnimaliaColeopteraCurculionidae

Riedel sp. n.

http://zoobank.org/935EA83F-0707-4FB1-A87B-29C3C04D1399

##### Diagnostic description.

**Figure 32.**
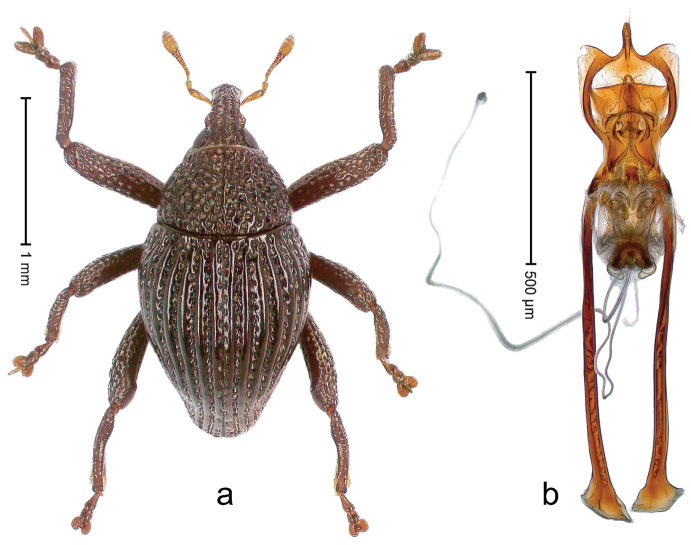
*Trigonopterus fissitarsis* Riedel, sp. n., holotype; **a** Habitus **b** Penis.

Holotype, male (Fig. 32a). Length 2.04 mm. Color of antennae ferruginous, legs dark ferruginous; remainder black. Body subovate, in dorsal aspect and in profile with weak constriction between pronotum and elytron. Rostrum in basal half with median ridge and pair of submedian ridges, intervening furrows each with sparse row of mesad directed setae; in apical third weakly scabrous; epistome simple. Pronotum coarsely punctate, laterally reticulate, submedially interspaces longitudinally rugose, with median costa; with sparse, slender, subrecumbent scales. Elytra with striae deeply impressed, with row of coarse punctures, each puncture containing suberect seta; intervals costate, subglabrous; incised suture bordered by row of punctures. Femora with simple, crenate anteroventral ridge. Metafemur subapically with stridulatory patch. Meso- and metatibia with posterior surface subglabrous, sparsely setose. Protarsi with tarsomere 3 enlarged, medially deeply incised. Abdominal ventrite 5 flat, coarsely punctate, medially and subapically subglabrous. Penis (Fig. 32b) in basal half with marked constriction, in apical half sides of body subparallel; apex medially with distinct spine, with anterolateral subangular flanges; transfer apparatus compact, symmetrical; apodemes 2.3 × as long as body; ductus ejaculatorius without bulbus. **Intraspecific variation.** Length 2.04–2.14 mm. Female unknown.

##### Material examined.

Holotype (MZB): ARC2211 (EMBL # LM655667), East Nusa Tenggara Prov., Flores Isl., Ruteng, Danau Ranamese, sample 6, S08°38.370', E120°33.798', 1215 m, 11-III-2011. Paratypes (SMNK): 1 ex, ARC2212 (EMBL # LM655668), same data as holotype.

##### Distribution.

East Nusa Tenggara Prov., Flores (Lake Ranamese). Elevation: 1215 m.

##### Etymology.

This epithet is based on a combination of the Latin adjective *fissus* (cleft) and the Greek noun *tarsos* and refers to the shape of the protarsi.

##### Notes.

*Trigonopterus fissitarsis* Riedel, sp. n. was coded as “*Trigonopterus* sp. 290” by Tänzler et al. (2014).

#### 33. Trigonopterus florensis

Taxon classificationAnimaliaColeopteraCurculionidae

Riedel sp. n.

http://zoobank.org/40D2DD79-FF31-49D5-B45A-A89B47F5A0F1

##### Diagnostic description.

**Figure 33.**
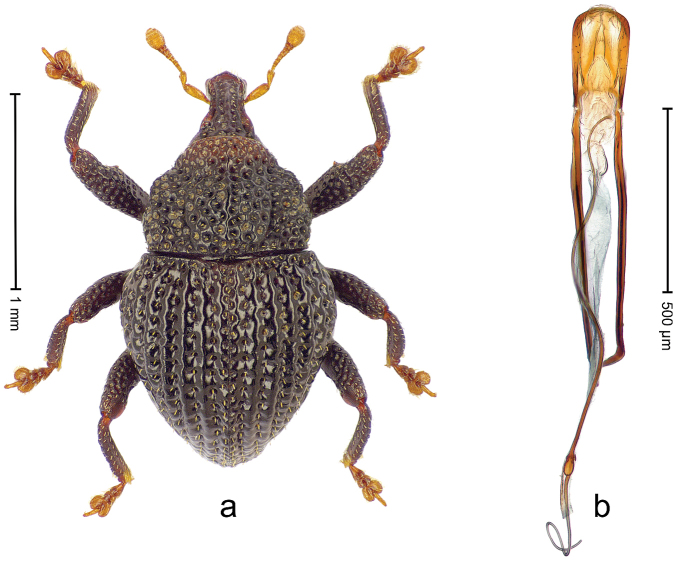
*Trigonopterus florensis* Riedel, sp. n., holotype; **a** Habitus **b** Penis.

Holotype, male (Fig. 33a). Length 1.72 mm. Color of antennae and tarsi ferruginous, remainder black. Body in dorsal aspect with marked constriction between pronotum and elytron; with distinct constriction in profile. Rostrum with median ridge and pair of submedian ridges, anteriorly coarsely punctate-scabrous; epistome with transverse, subangulate ridge. Pronotum with subapical constriction; disk coarsely punctate-reticulate, sparsely setose; with pair of curved sublateral impressions; medially swollen. Elytra relatively compact; with striae deeply impressed, each with row of short suberect bristles; intervals costate, subglabrous; apex narrow, rounded. Femora each with small tooth. Metafemur subapically with stridulatory patch. Abdominal ventrite 5 coarsely punctate, with shallow median impression bordered by pair of weak longitudinal costae. Penis (Fig. 33b) with sides subparallel, in apical third markedly converging to narrow subglabrous apex; transfer apparatus flagelliform, 3.9 × as long as body; apodemes 3.5 × as long as body; ductus ejaculatorius torn and apical portion missing. **Intraspecific variation.** Length 1.48–2.04 mm. Color of elytra dark ferruginous to black. Female rostrum dorsally in apical half subglabrous, with submedian rows of sparse minute punctures, with dorsolateral pair of furrows; epistome simple. Pronotum in smaller specimens narrower, in large specimens wider. Elytra in larger specimens with humeri more distinctly subangularly projecting laterad, in smaller specimens evenly rounded; intervals weakly costate in smaller specimens, markedly costate or carinate in larger specimens. Penis with flagelliform transfer apparatus 3.9–4.0 × as long as body; apodemes 3.2–3.7 × as long as body.

##### Material examined.

Holotype (MZB): ARC3618 (EMBL # LM656048), East Nusa Tenggara Prov., Flores, Ruteng, Mt. Ranaka, sample 1, S08°37.321', E120°31.463' 1535 m, 08-III-2011. Paratypes (MZB, SMNK, ZSM): Flores: 3 exx, ARC3616 (EMBL # LM656046), ARC3617 (EMBL # LM656047), ARC3619 (EMBL # LM656049), same data as holotype; 2 exx, ARC2189 (EMBL # LM655646), ARC2190 (EMBL # LM655647), Ruteng, Lake Ranamese, sample 1, S08°37.705', E120°33.702', 1320 m, 08-III-2011; 1 ex, ARC3621 (EMBL # LM656051), Ruteng, Lake Ranamese, sample 4, S08°37.705', E120°33.702', 1250 m, 08-III-2011; 2 exx, ARC2210 (EMBL # LM655666), ARC3620 (EMBL # LM656050), Ruteng, Golo Lusang, sample 1, S08°39.864', E120°27.322', 1590 m, 11-III-2011.

##### Distribution.

East Nusa Tenggara Prov., Flores (Golo Lusang, Mt. Ranaka, Lake Ranamese). Elevation: 1250–1590 m.

##### Etymology.

This epithet is based on the island of Flores.

##### Notes.

*Trigonopterus florensis* Riedel, sp. n. was coded as “*Trigonopterus* sp. 440”. Morphologically it is very similar to *Trigonopterus pseudoflorensis* Riedel, sp. n. and *Trigonopterus paraflorensis* Riedel, sp. n. but its cox1 sequences differ 6.69%, respectively 7.75% smallest interspecific *p*-distance.

#### 34. Trigonopterus foveatus

Taxon classificationAnimaliaColeopteraCurculionidae

Riedel sp. n.

http://zoobank.org/9453D91A-979E-4F24-9E4A-8F687E6122F2

##### Diagnostic description.

**Figure 34.**
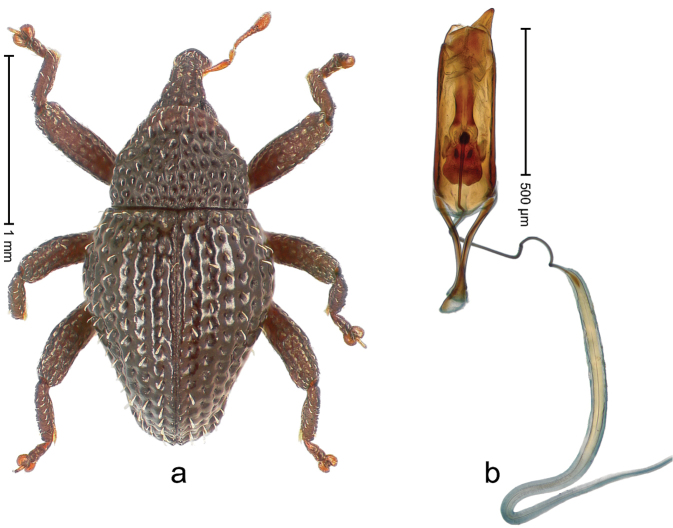
*Trigonopterus foveatus* Riedel, sp. n., holotype; **a** Habitus **b** Penis.

Holotype, male (Fig. 34a). Length 2.06 mm. Color of head and legs ferruginous; remainder black. Body subovate; in dorsal aspect and in profile with distinct constriction between pronotum and elytron. Rostrum in apical half scabrous, in basal half with median ridge and pair of submedian ridges; with sparse suberect scales; epistome with transverse, angulate ridge. Pronotum with shallow subapical constriction; disk scabrous, coarsely punctate; interspaces dull, microreticulate; each puncture containing suberect clavate scale inserting at posterior rim. Elytra with striae marked by deep punctures; in front of each puncture with elongate-claviform, erect scale; intervals costate, subglabrous; interval 7 subapically costate; sutural interval simple. Femora edentate. Metafemur subapically without stridulatory patch. Abdominal ventrite 5 flat, microreticulate, with sparse scales. Penis (Fig. 34b) with apex asymmetrically extended to the left; apical extension short; endophallus at middle of body with pair of elongate parallel sclerites; basal orifice ventrally with rim; apodemes short, 0.5 × as long as body; ductus ejaculatorius with bulbus. **Intraspecific variation.** Length 2.02–2.20 mm. Female rostrum dorsally in apical half subglabrous, densely punctate; epistome simple. Endophallic sclerites in specimens from Leuweung Sancang with subbasal sclerite shorter, pair of subapical sclerites less distinct.

##### Material examined.

Holotype (MZB): ARC2690 (EMBL # LM655948), W-Java, Ciamis, Pangandaran, sample 1, S07°42.836', E108°39.478', 157 m, 22-IV-2012. Paratypes (MZB, SMNK, ZSM): W-Java: 4 exx, ARC2684 (EMBL # LM655942), ARC2685 (EMBL # LM655943), same data as holotype; 1 ex, Ciamis, Pangandaran, sample 3, S07°43.070', E108°39.634', 156 m, 22-IV-2012; 4 exx, ARC2693 (EMBL # LM655949), ARC2694 (EMBL # LM655950), ARC2695 (EMBL # LM655951), Garut, Pameungpeuk, Leuweung Sancang, sample 2, S07°43.807', E107°53.700', 74 m, 24-IV-2012.

##### Distribution.

W-Java Prov. (Leuweung Sancang, Pangandaran). Elevation: 74–157 m.

##### Etymology.

This epithet is based on the Latin adjective *foveatus* (pitted) and refers to its sculpture.

##### Notes.

*Trigonopterus foveatus* Riedel, sp. n. was coded as “*Trigonopterus* sp. 375”.

#### 35. Trigonopterus fulgidus

Taxon classificationAnimaliaColeopteraCurculionidae

Riedel sp. n.

http://zoobank.org/CB109322-B3C5-4108-B272-5F27B08F5ACB

##### Diagnostic description.

**Figure 35.**
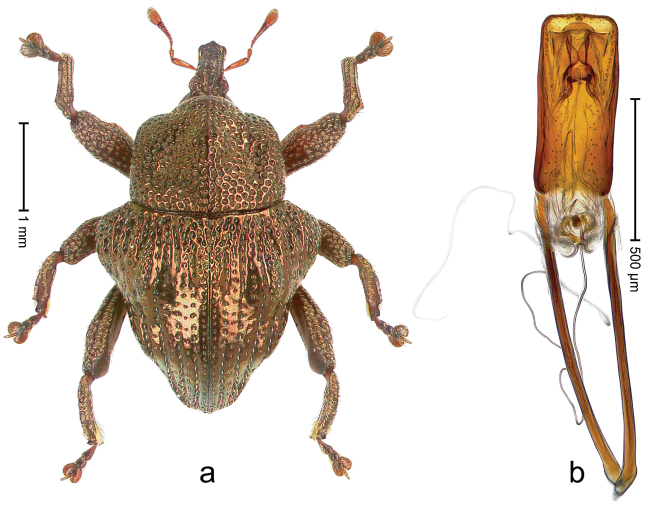
*Trigonopterus fulgidus* Riedel, sp. n., holotype; **a** Habitus **b** Penis.

Holotype, male (Fig. 35a). Length 3.66 mm. Color of legs, head and ventral surface ferruginous; pronotum and elytron reddish coppery, partly with slight greenish lustre. Body in dorsal aspect with marked constriction between pronotum and elytron; in profile dorsally convex. Rostrum in apical third scabrous; in basal half with median and pair of submedian ridges; intervening furrows each with row of coarse punctures and suberect piliform scales; epistome with transverse, angulate ridge forming distinct median denticle. Pronotum with sides subparallel; anteriorly abruptly rounded to subapical constriction; disk with pair of submedian impressions, coarsely punctate, reticulate; each puncture containing inconspicuous seta; medially swollen. Elytra with humeri markedly swollen, laterally projecting; interval 4 subbasally swollen, forming dorsal protrusion; interval 5 behind middle weakly swollen, gently projecting from lateral outline; striae distinct; intervals flat, each with row of small punctures; subbasally striae 2–6 deeply incised, intervals and humeral callus densely coarsely punctate; punctures each bearing small inconspicuous scale. Anteroventral ridge of femora distinct, in meso- and metafemur forming large tooth. Metafemur subapically with stridulatory patch. Dorsal edge of pro- and mesotibia subbasally with angulation; meso- and metatibia subapically with dorsal margin concave; mesotibia in apical 1/3 with tooth on dorsal edge. Abdominal ventrite 5 flat, coarsely punctate; with long, suberect setae. Penis (Fig. 35b) with sides of body subparallel; apex subangulate; transfer apparatus small, compact; apodemes 2.0 × as long as body; ductus ejaculatorius without bulbus. **Intraspecific variation.** Length 2.38–3.78 mm. Color of pronotum and elytron ranging from reddish to greenish coppery. Female body more slender. Female rostrum dorsally punctate-rugose, with median and pair of submedian subglabrous costae; epistome simple. Female elytra subovate, without swellings, lateral contour convex, humeri simple.

##### Material examined.

Holotype (MZB): ARC2274 (EMBL # LM655728), West Nusa Tenggara Prov., Lombok, Santong, Rinjani-track, sample 1, S08°20.715', E116°19.695', 830 m, 24-III-2011. Paratypes (MZB, SMNK, ZSM): 3 exx, same data as holotype; 4 exx, Santong, Rinjani-track, sample 2, S08°20.813', E116°19.778', 870 m, 24-III-2011; 10 exx, ARC2281 (EMBL # LM655735), ARC2282 (EMBL # LM655736), Lombok, Santong, Rinjani-track, sample 3, S08°20.996', E116°20.001', 960 m, 25-III-2011; 1 ex, Santong, Rinjani-track, sample 4, S08°21.160', E116°20.067', 1005 m, 25-III-2011; 1 ex, Santong, Rinjani-track, sample 5, S08°21.351', E116°20.208', 1105 m, 25-III-2011; 1 ex, Santong, Rinjani-track, sample 6, S08°21.754', E116°20.476', 1315 m, 25-III-2011; 9 exx, ARC2256 (EMBL # LM655710), ARC2257 (EMBL # LM655711), ARC2258 (EMBL # LM655712), Lombok, Senaru, Rinjani-track, sample 1, S08°19.429', E116°24.082', 900 m, 21-III-2011; 2 exx, Lombok, Senaru, Rinjani-track, sample 4, S08°20.439', E116°24.047', 1240 m, 21-III-2011; 16 exx, Lombok, Senaru, Rinjani-track, sample 6, S08°19.359', E116°24.070', 860 m, 23-III-2011; 3 exx, Senaru, Rinjani-trail, sample 7, S08°19.643', E116°24.033', 935 m, 23-III-2011; 7 exx, Senaru, Rinjani-trail, sample 8, S08°19.719', E116°24.040', 955 m, 23-III-2011; 12 exx, ARC2288 (EMBL # LM655742), ARC2289 (EMBL # LM655743), ARC2290 (EMBL # LM655744), Lombok, Mt. Rinjani, Bawnau, near Sembalun, sample 1, S08°21.136', E116°29.257', 1140 m, 26-III-2011.

##### Distribution.

West Nusa Tenggara Prov., Lombok (Santong, Sembalun, Senaru). Elevation: 830–1240 m.

##### Etymology.

This epithet is based on the Latin adjective *fulgidus* (shining, gleaming) and refers to its vivid coloration.

##### Notes.

*Trigonopterus fulgidus* Riedel, sp. n. was coded as “*Trigonopterus* sp. 349” by Tänzler et al. (2014).

#### 36. Trigonopterus gedensis

Taxon classificationAnimaliaColeopteraCurculionidae

Riedel sp. n.

http://zoobank.org/ACB733AD-E1F4-4BCF-A493-B828231CE337

##### Diagnostic description.

**Figure 36.**
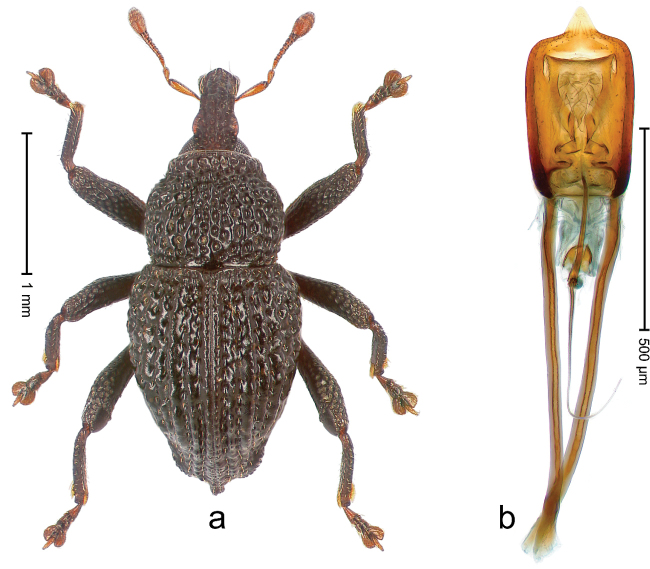
*Trigonopterus gedensis* Riedel, sp. n., holotype; **a** Habitus **b** Penis.

Holotype, male (Fig. 36a). Length 2.53 mm. Color black, tarsi and antennae ferruginous. Body elongate; in dorsal aspect with marked constriction between pronotum and elytron; with distinct constriction in profile. Rostrum coarsely rugose-punctate, with median ridge and pair of irregular submedian ridges; epistome with indistinct, transverse, subangulate ridge. Pronotum with disk scabrous; with median ridge; with indistinct subapical constriction. Elytra with striae deeply impressed, each with row of short subrecumbent bristles; intervals costate-tuberculate, almost nude; interval 7 swollen subapically, laterally weakly projecting; sutural interval subapically with tooth. Femora edentate. Metafemur subapically with stridulatory patch and transverse rows of denticles. Abdominal ventrite 5 flat, punctate, sparsely setose. Penis (Fig. 36b) with body in profile moderately curved; in dorsal aspect sides subparallel; apex with median, acute triangular extension; transfer apparatus flagelliform, as long as body; apodemes 2.0 × as long as body; ductus ejaculatorius without bulbus. **Intraspecific variation.** Length 2.30–2.55 mm. Female rostrum dorsally with pair of lateral furrows, in apical half subglabrous, coarsely punctate; epistome simple. Female elytra with sutural interval subapically with relatively indistinct knob.

##### Material examined.

Holotype (MZB): ARC0249 (EMBL # LM655441), West Java Prov., Cianjur, above kebun Gede, sample 2, S06°47.735', E107°00.658', 2005 m, sifted. Paratypes (MZB, SMNK, ZSM): W-Java Prov., Mt. Gede: 5 exx, ARC0250 (EMBL # LM655442), ARC0251 (EMBL # LM655443), ARC0252 (EMBL # LM655444), ARC0253 (EMBL # LM655445), same data as holotype; 22 exx, ARC0003, Cibodas, 2000 m, 07-VI-2002; 4 exx, ARC2664 (EMBL # LM655922), Sukabumi, Cisaat, Situ Gunung, sample 5, S06°47.912', E106°56.457', 1940 m, 16-IV-2012; 5 exx, ARC2665 (EMBL # LM655923), Sukabumi, Cisaat, Situ Gunung, sample 6, S06°47.706', E106°56.560', 2117 m, 16-IV-2012.

##### Distribution.

W-Java Prov., (Mt. Gede). Elevation: 1940–2117 m.

##### Etymology.

This epithet is based on the type locality.

##### Notes.

*Trigonopterus gedensis* Riedel, sp. n. was coded as “*Trigonopterus* sp. 307” by Tänzler et al. (2014).

#### 37. Trigonopterus halimunensis

Taxon classificationAnimaliaColeopteraCurculionidae

Riedel sp. n.

http://zoobank.org/FEEBFB61-BDFD-402F-992D-C21CC6BA2AC0

##### Diagnostic description.

**Figure 37.**
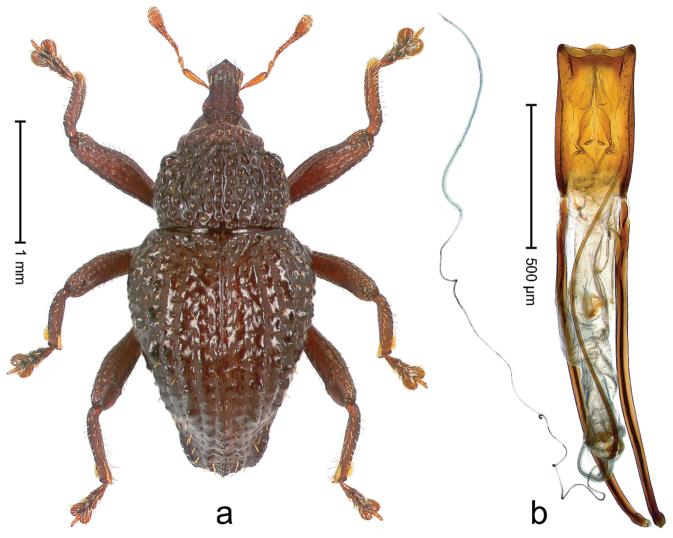
*Trigonopterus halimunensis* Riedel, sp. n., holotype; **a** Habitus **b** Penis.

Holotype, male (Fig. 37a). Length 2.90 mm. Color of head, disk of elytra, legs and antennae ferruginous; remainder black. Body elongate; in dorsal aspect with marked constriction between pronotum and elytron; with distinct constriction in profile. Rostrum with median ridge and pair of submedian ridges, intervening furrows punctate; epistome with indistinct, transverse, subangulate ridge, at middle with denticle. Pronotum anterolaterally weakly angularly projecting; with distinct subapical constriction; disk coarsely punctate-rugose; punctures each with slender suberect scale; with median ridge. Elytra with striae deeply impressed, each with partly abraded row of slender, suberect scales; intervals tuberculate, almost nude; interval 7 swollen subapically, laterally weakly projecting. Femora edentate. Metafemur subapically with stridulatory patch and transverse row of denticles. Metatibia ventrally with sparse row of long erect setae, apically with uncus and distinct premucro. Abdominal ventrite 5 with shallow median impression, punctate, microreticulate. Penis (Fig. 37b) with body in profile curved, basal half flattened; in dorsal aspect sides subparallel; apex medially weakly extended; transfer apparatus flagelliform, ca. 2.4 × as long as body; apodemes 2.6 × as long as body; ductus ejaculatorius without bulbus. **Intraspecific variation.** Length 2.53–3.00 mm. Color of body black to ferruginous. Female rostrum dorsally with longitudinal furrows; epistome simple. Pronotum with anterolateral knobs blunt, somewhat rounded, or more distinct angularly projecting. Elytral striae with erect scales slightly shorter or longer and, rather dense or sparse depending on state of abrasion. Male metatibia ventrally with fringe of long setae dense, sparse or almost absent. Female abdominal ventrite 5 flat or slightly convex.

##### Material examined.

Holotype (MZB): ARC2647 (EMBL # LM655905), West Java Prov., Bogor, Cidahu, Mt. Salak (near Javana Spa), sample 1, S06°43.733', E106°42.711', 1429 m, 13-IV-2012. Paratypes (ARC, MZB, SMNK, ZSM): W-Java Prov.: 1 ex, ARC2646 (EMBL # LM655904), same data as holotype; 1 ex, Bogor, Cidahu, Mt. Salak (near Javana Spa), sample 2, S06°43.499', E106°42.970', 1550 m, 13-IV-2012; 5 exx, ARC2650 (EMBL # LM655908), ARC2651 (EMBL # LM655909), Bogor, Cidahu, Mt. Salak (near Javana Spa), sample 3, S06°43.425', E106°43.227', 1756 m, 13-IV-2012; 4 exx, ARC0103, Mt. Halimun N.P., Pasir Banteng, Mt. Botol, 1550 m, 17-XI-2004; 3 exx, ARC0183 (EMBL # LM655408), Mt. Halimun N.P., Citalahab, Mt. Kendeng, 1550 m, 19-XI-2004; 5 exx, ARC0185 (EMBL # LM655410), Mt. Halimun N.P., Citalahab, Mt. Kendeng, sample 2, S06°45.523', E106°31.435', 1600 m, 10-IX-2005; 1 ex, ARC0188 (PCR failed), Mt. Halimun N.P., Citalahab, Mt. Kendeng, sample 1B, S06°45.723', E106°31.473', 1695 m, 10-IX-2005.

##### Distribution.

W-Java Prov. (Mt. Halimun Salak N.P.). Elevation: 1429–1756 m.

##### Etymology.

This epithet is based on the type locality.

##### Notes.

*Trigonopterus halimunensis* Riedel, sp. n. was coded as “*Trigonopterus* sp. 299” by Tänzler et al. (2014).

#### 38. Trigonopterus honjensis

Taxon classificationAnimaliaColeopteraCurculionidae

Riedel sp. n.

http://zoobank.org/6198FEC4-730D-4207-AA7A-AED444EE0FDF

##### Diagnostic description.

**Figure 38.**
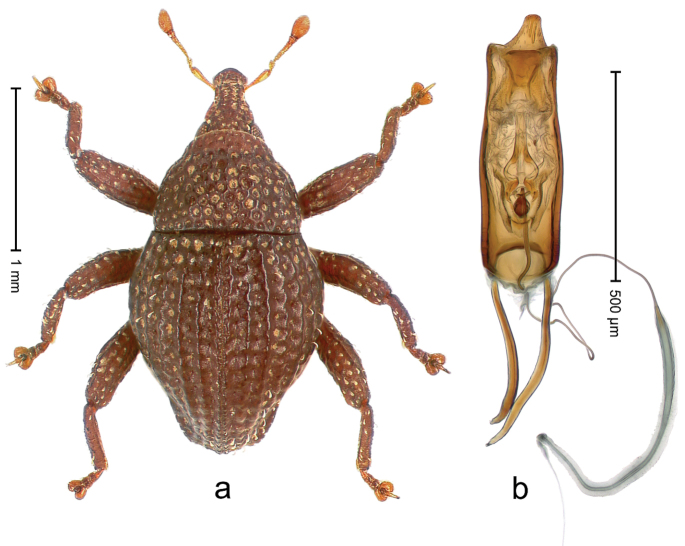
*Trigonopterus honjensis* Riedel, sp. n., holotype; **a** Habitus **b** Penis.

Holotype, male (Fig. 38a). Length 2.02 mm. Color ferruginous. Body subovate; in dorsal aspect and in profile with distinct constriction between pronotum and elytron. Rostrum irregularly rugose-punctate; epistome with transverse, curved ridge. Pronotum with shallow subapical constriction; disk reticulate-punctate; punctures with flat, apparently elevated bottom; scales abraded; surface dull, microreticulate. Elytra with striae deeply impressed, in front of each puncture with claviform, suberect scale unless abraded; intervals costate; surface dull, microreticulate; interval 7 in apical half forming distinct ridge. Femora edentate. Metafemur subapically without stridulatory patch. Abdominal ventrite 5 flat, microreticulate, with sparse setae. Penis (Fig. 38b) with apex asymmetrically extended on the left; apical extension short; basal orifice ventrally with rim; apodemes short, 0.7 × as long as body; ductus ejaculatorius with bulbus. **Intraspecific variation.** Length 1.99–2.22 mm. Color light to deep ferruginous. Female rostrum dorsally in apical half subglabrous, with small punctures; epistome simple. Pronotum with erect clavate scale in each puncture if not abraded.

##### Material examined.

Holotype (MZB): ARC1547 (EMBL # LM655645), Java, Banten-Prov., Ujung Kulon N.P., Tama Jaya, Mt. Honje, sample 4, S06°46.141', E105°31.649', 395 m, 23-IV-2010. Paratypes (MZB, SMNK, ZSM): Java, Banten-Prov., Ujung Kulon N.P., Tama Jaya, Mt. Honje: 6 exx, ARC1542 (EMBL # LM655640), ARC1543 (EMBL # LM655641), ARC1544 (EMBL # LM655642), sample 2, S06°45.973', E105°31.915', 540 m, 23-IV-2010; 16 exx, sample 4, S06°46.141', E105°31.649', 395 m, 23-IV-2010; 2 exx, sample 3, S06°46.009', E105°31.761', 465 m, 23-IV-2010.

##### Distribution.

Banten Prov. (Ujung Kulon N.P.). Elevation: 395–540 m.

##### Etymology.

This epithet is based on the type locality, Mt. Honje.

##### Notes.

*Trigonopterus honjensis* Riedel, sp. n. was coded as “*Trigonopterus* sp. 338”.

#### 39. Trigonopterus ijensis

Taxon classificationAnimaliaColeopteraCurculionidae

Riedel sp. n.

http://zoobank.org/2B46FBC3-E3C4-4D62-8073-9E442F9D1679

##### Diagnostic description.

**Figure 39.**
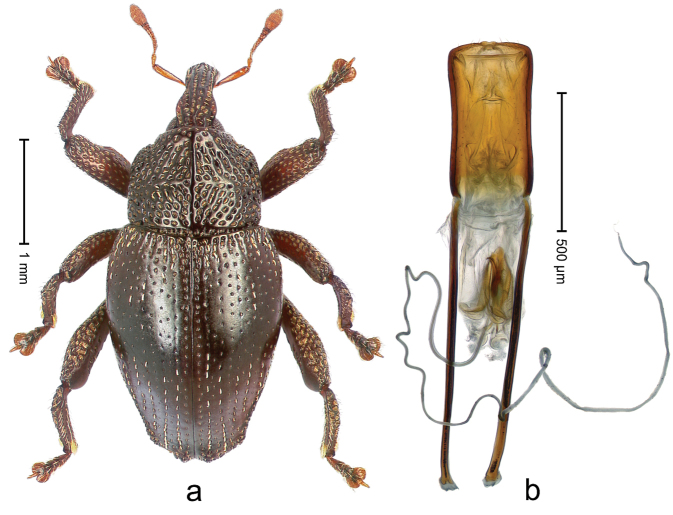
*Trigonopterus ijensis* Riedel, sp. n., holotype; **a** Habitus **b** Penis.

Holotype, male (Fig. 39a). Length 3.25 mm. Color of legs and head ferruginous, elytra deep ferruginous with transverse black band, remainder black. Body elongate; in dorsal aspect with marked constriction between pronotum and elytron; in profile dorsally convex. Rostrum with median and pair of submedian ridges; intervening furrows punctate, each with row of erect piliform scales; epistome simple. Pronotum with subapical constriction; disk densely punctate; punctures partly elongate and / or arranged in confluent rows forming rhombus-like pattern; each puncture containing elongate white scale or seta; medially with glabrous costa. Elytra with striae indistinct; intervals flat; with small, confused punctures; with sparse rows of elongate, recumbent, cream-colored scales; interspaces microreticulate; interval 7 in apical half forming sharp lateral edge; sutural interval at apex swollen, weakly protruding. Anteroventral ridge of femora crenulate, forming blunt tooth. Metafemur subapically with stridulatory patch. Dorsal edge of tibiae subbasally with angulation. Metatibia apically with angular premucro. Abdominal ventrite 5 flat, coarsely punctate. Penis (Fig. 39b) with sides of body subparallel; apex rounded, medially truncate; transfer apparatus compact, transfer-processes sickle-shaped, in profile ventrally with subangular extension; apodemes 2.0 × as long as body; ductus ejaculatorius without bulbus. **Intraspecific variation.** Length 2.75–3.31 mm. Body with or without bronze lustre. Female rostrum with median and pair of submedian ridges costate, subglabrous.

##### Material examined.

Holotype (MZB): ARC0305 (EMBL # LM655495), East Java Prov., Banyuwangi, Mt. Ijen, Licin, sample 4, S08°06.673', E114°14.488', 1225 m, 31-VIII-2006. Paratypes (MZB, SMNK, ZSM): E-Java Prov., Banyuwangi, Mt. Ijen, Licin: 11 exx, ARC0304 (EMBL # LM655494), ARC0306 (EMBL # LM655496), ARC0307 (EMBL # LM655497), same data as holotype; 1 ex, ARC0303 (EMBL # LM655493), sample 2, S08°05.603', E114°14.366', 1575 m, 28-VIII-2006; 2 exx, sample 3, S08°06.056', E114°14.621', 1400 m, 28-VIII-2006; 5 exx, Banyuwangi, Mt. Ijen, Licin, sample 4, S08°06.673', E114°14.488', 1255 m, 28-VIII-2006.

##### Distribution.

E-Java Prov. (Mt. Ijen). Elevation: 1225–1575 m.

##### Etymology.

This epithet is based on the type locality.

##### Notes.

*Trigonopterus ijensis* Riedel, sp. n. was coded as “*Trigonopterus* sp. 316”.

#### 40. Trigonopterus javensis

Taxon classificationAnimaliaColeopteraCurculionidae

Riedel sp. n.

http://zoobank.org/BBC21FDB-BB0C-496F-AD3E-DF83CFE21F62

##### Diagnostic description.

**Figure 40.**
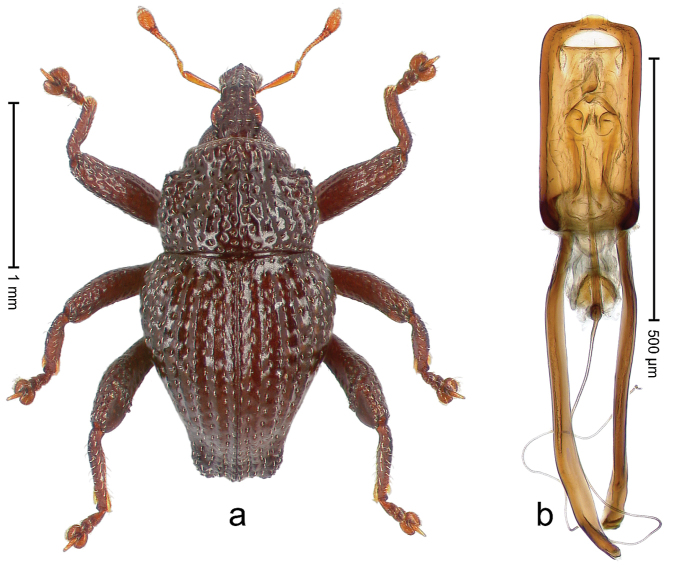
*Trigonopterus javensis* Riedel, sp. n., holotype; **a** Habitus **b** Penis.

Holotype, male (Fig. 40a). Length 2.08 mm. Color ferruginous. Body subhexagonal; in dorsal aspect with marked constriction between pronotum and elytron; with distinct constriction in profile. Rostrum coarsely rugose-punctate, with irregular median ridge; epistome with indistinct transverse ridge forming median denticle. Pronotum anterolaterally angularly projecting; with distinct subapical constriction; disk coarsely punctate, punctures each with one slender suberect scale; with median ridge. Elytra with striae deeply impressed, intervals costate to subcarinate; striae and intervals each with row of subrecumbent, short bristles; interval 7 swollen subapically, laterally projecting. Femora edentate. Metafemur subapically with stridulatory patch and transverse rows of denticles. Abdominal ventrite 5 somewhat swollen, at middle with shallow impression, sparsely setose. Penis (Fig. 40b) with body flattened, sides subparallel; apex subangulate; transfer apparatus spiniform, subequal to body; ductus ejaculatorius without bulbus. **Intraspecific variation.** Length 1.76–2.09 mm. Color of elytra black or ferruginous. Female rostrum dorsally with longitudinal furrows; epistome simple. Pronotum anterolaterally more or less distinctly angularly projecting. Female abdominal ventrite 5 flat.

##### Material examined.

Holotype (MZB): ARC0192 (EMBL # LM655416), West Java Prov., Mt. Halimun N.P., N Ciptarasa, sample 3, S06°49.867', E106°30.085', 1100 m, 19-IX-2005. Paratypes (MZB, SMNK, ZSM): 17 exx, ARC0156, ARC0190 (EMBL # LM655414), ARC0191 (EMBL # LM655415), same data as holotype; 4 exx, W-Java Prov., Citalahab, sample 2, S06°44.503', E106°31.550', 1200 m, 12-IX-2005; 6 exx, ARC2656 (EMBL # LM655914), ARC2657 (EMBL # LM655915), W-Java Prov., Sukabumi, Mt. Gede, Cisaat, Situ Gunung, sample 1, S06°49.377', E106°55.729', 1281 m, 15-IV-2012; 3 exx, ARC2660 (EMBL # LM655918), ARC2661 (EMBL # LM655919), W-Java Prov., Sukabumi, Mt. Gede, Cisaat, Situ Gunung, sample 2, S06°49.038', E106°55.852', 1342 m, 15-IV-2012; 4 exx, ARC0196, ARC0197 (EMBL # LM655420), ARC0198, W-Java Prov., Ciamis, Mt. Sawal, Batu Cakra, sample 1, S07°14.920', E108°15.762', 990 m, 01-X-2005; 2 exx, W-Java Prov., Ciamis, Mt. Sawal, Blok Cireong, sample 2, S07°14.127', E108°15.568', 1120 m, 01-X-2005; 1 ex, ARC3593 (EMBL # LM656024), Sumedang, Sukajadi, Mt. Cakrabuana, sample 5, S07°01.804', E108°08.044', 1563 m, 20-IV-2012; 4 exx, ARC0285 (EMBL # LM655475), ARC0286 (EMBL # LM655476), ARC0287 (EMBL # LM655477), C-Java Prov., N slopes of Dieng figau, Petungkriyono, Mt. Deles, sample 1, S07°08.221', E109°43.599', 1505 m, 24-VIII-2006; 4 exx, ARC0297 (EMBL # LM655487), C-Java Prov., N slopes of Dieng figau, Petungkriyono, Mt. Deles, sample 3, S07°08.225', E109°43.555', 1495 m, 24-VIII-2006; 4 exx, ARC0296 (EMBL # LM655486), C-Java Prov., N slopes of Dieng figau, Petungkriyono, mountain N Tinalum, sample 1, S07°06.418', E109°44.514', 1115 m, 22-VIII-2006; 1 ex, C-Java Prov., Mt. Slamet, Guci, sample 5, S07°11.953', E109°10.497', 1620 m, 27-XI-2011; 6 exx, ARC2496 (EMBL # LM655833), ARC2497 (EMBL # LM655834), ARC2498 (EMBL # LM655835), C-Java Prov., Mt. Slamet, Guci, sample 6, S07°11.983', E109°10.556', 1671 m, 27-XI-11.

##### Distribution.

W-Java Prov. (Mt. Gede, Mt. Halimun N.P., Mt. Cakrabuana, Mt. Sawal), C-Java Prov. (Dieng, Mt. Slamet). Elevation: 990–1671 m.

##### Etymology.

This epithet is based on the name of Java Island.

##### Notes.

*Trigonopterus javensis* Riedel, sp. n. was coded as “*Trigonopterus* sp. 294”.

#### 41. Trigonopterus kalimantanensis

Taxon classificationAnimaliaColeopteraCurculionidae

Riedel sp. n.

http://zoobank.org/CB7D90D9-2E5D-42E9-977C-11095E91E11D

##### Diagnostic description.

**Figure 41.**
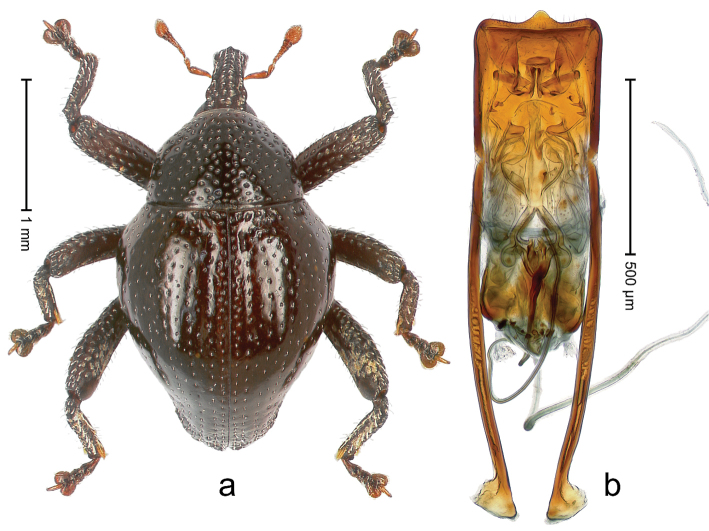
*Trigonopterus kalimantanensis* Riedel, sp. n., holotype; **a** Habitus **b** Penis.

Holotype, male (Fig. 41a). Length 2.65 mm. Color of antennae light ferruginous, pronotum black, remainder dark ferruginous. Body in dorsal aspect with marked constriction between pronotum and elytron; in profile dorsally convex, with shallow constriction. Rostrum with median and pair of submedian ridges; intervening furrows containing row of punctures, with rows of erect scales; epistome with transverse, irregular ridge. Pronotum without subapical constriction; disk densely punctate; interspaces subglabrous; each puncture containing small recumbent seta. Elytra with striae marked by small punctures, each containing minute seta; intervals flat, subglabrous, with interspersed punctures; elytral apex subangulate, densely coarsely punctate, suture incised. Femora with crenate anteroventral ridge. Metafemur subapically with stridulatory patch. Dorsal edge of tibiae with subbasal angulation, dentate in pro- and mesotibia. Abdominal ventrites 1–2 weakly concave, subglabrous, with sparse erect scales; abdominal ventrite 5 flat, with coarse shallow punctures, microreticulate. Penis (Fig. 41b) with sides of body subparallel; apex subangulate, with median triangular extension; endophallus with complex structures, several sclerites, containing few denticles; transfer apparatus complex; apodemes 2.6 × as long as body; ductus ejaculatorius without distinct bulbus. **Intraspecific variation.** Length 2.35–2.65 mm. Female rostrum in apical half subglabrous, sparsely punctate-rugose; epistome simple. Female elytral apex simple, suture very weekly incised.

##### Material examined.

Holotype (MZB): ARC1421 (EMBL # LM655548), E-Kalimantan Prov., Berau Dist., 1 km off road Tanjungredeb - Tanjungselor, ca. 45 km N of Tanjungredeb, N02°29.55', E117°28.77', 190 m, 29-IX/03-X-2008 (MZB). Paratypes (SMNK): 2 exx, ARC1422 (EMBL # LM655549), ARC1423 (EMBL # LM655550), same data as holotype.

##### Distribution.

E-Kalimantan Prov. (Tanjungredeb). Elevation: 190 m.

##### Etymology.

This epithet is based on the Indonesian name for Borneo, Kalimantan.

##### Notes.

*Trigonopterus kalimantanensis* Riedel, sp. n. was coded as “*Trigonopterus* sp. 310” by Tänzler et al. (2014).

#### 42. Trigonopterus kintamanensis

Taxon classificationAnimaliaColeopteraCurculionidae

Riedel sp. n.

http://zoobank.org/F0437FFA-E4F5-40D6-BEA8-35DAFEA30C05

##### Diagnostic description.

**Figure 42.**
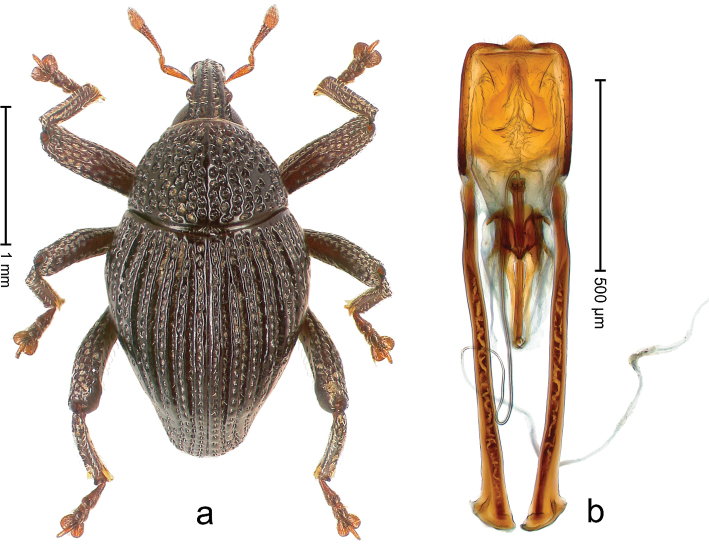
*Trigonopterus kintamanensis* Riedel, sp. n., holotype; **a** Habitus **b** Penis.

Holotype, male (Fig. 42a). Length 2.45 mm. Color of antennae and legs ferruginous; remainder black. Body subovate, in dorsal aspect with weak constriction between pronotum and elytron; in profile dorsally convex. Rostrum with median ridge and pair of submedian ridges, intervening furrows each with sparse row of mesad directed scales; epistome with transverse ridge. Pronotum coarsely punctate, reticulate, with median costa; with sparse, recumbent scales. Elytra with striae dorsally deeply incised, laterally marked by rows of coarse punctures; intervals dorsally markedly costate, flattened, each with row of partly confluent punctures; each puncture containing small recumbent scale; elytral apex subtruncate. Femora with simple anteroventral ridge. Metafemur subapically with stridulatory patch. Abdominal ventrite 5 coarsely punctate, with sparse suberect scales. Penis (Fig. 42b) with sides of body subparallel, before apex weakly converging in straight line; apex with median angulate extension; transfer apparatus with median rod 1.6 × as long as body; median rod projecting basad further than apicad; apodemes 2.9 × as long as body; ductus ejaculatorius without bulbus. **Intraspecific variation.** Length 2.28–2.45 mm. Female rostrum in apical half dorsally subglabrous, with submedian rows of punctures, laterally punctate-rugose; epistome simple.

##### Material examined.

Holotype (MZB): ARC0589 (EMBL # LM655525), Bali, Kintamani, Mt. Abang, sample 1, S08°17.118', E115°24.868', 1440 m, 09-XI-2007. Paratypes (SMNK): Bali: 1 ex, ARC0590 (EMBL # LM655526), same data as holotype.

##### Distribution.

Bali (Kintamani). Elevation: 1440 m.

##### Etymology.

This epithet is based on the type locality, Kintamani.

##### Notes.

*Trigonopterus kintamanensis* Riedel, sp. n. was coded as “*Trigonopterus* sp. 286” by Tänzler et al. (2014).

#### 43. Trigonopterus klatakanensis

Taxon classificationAnimaliaColeopteraCurculionidae

Riedel sp. n.

http://zoobank.org/8B4380C6-AD7A-47EC-9D63-555500933153

##### Diagnostic description.

**Figure 43.**
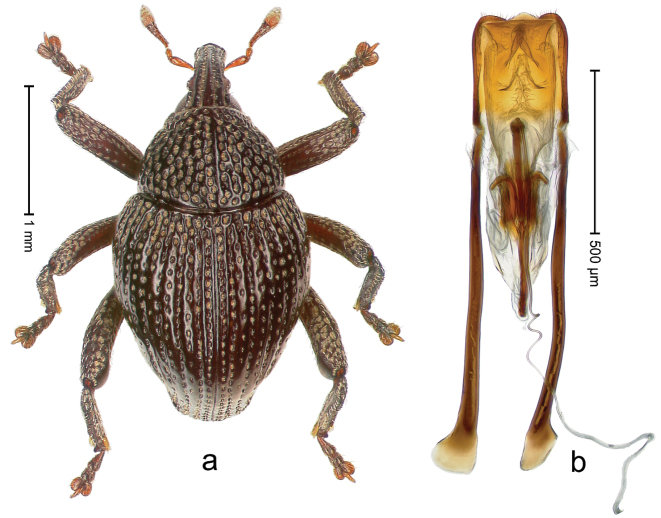
*Trigonopterus klatakanensis* Riedel, sp. n., holotype; **a** Habitus **b** Penis.

Holotype, male (Fig. 43a). Length 2.50 mm. Color of antennae and legs ferruginous; remainder dark ferruginous. Body subovate, in dorsal aspect with weak constriction between pronotum and elytron; in profile dorsally convex. Rostrum with median ridge and pair of submedian ridges, intervening furrows each with sparse row of mesad directed scales; epistome with irregular transverse ridge. Pronotum coarsely punctate, reticulate, with median costa; with sparse, recumbent scales. Elytra with striae distinct, marked by rows of punctures; intervals weakly costate, with rows of punctures; sutural interval basally swollen, coarsely punctate; each puncture containing minute recumbent seta; elytral apex subtruncate, rounded. Femora with simple anteroventral ridge. Metafemur subapically with stridulatory patch. Abdominal ventrite 5 coarsely punctate, with sparse subrecumbent scales. Penis (Fig. 43b) with sides of body subparallel; apex with median angulate extension; transfer apparatus with thick median rod 2.0 × as long as body; median rod projecting basad further than apicad; apodemes 3.1 × as long as body; ductus ejaculatorius without bulbus. **Intraspecific variation.** Length 2.22–2.53 mm. Color of elytra ferruginous to black. Female rostrum in apical half dorsally subglabrous, sparsely punctate; epistome simple.

##### Material examined.

Holotype (MZB): ARC0595 (EMBL # LM655531), Bali, National Park, Mt. Klatakan, sample 1, S08°13.220', E114°29.509', 545 m, 12-XI-2007. Paratypes (MZB, SMNK): Bali National Park, Mt. Klatakan: 3 exx, ARC0596 (EMBL # LM655532), ARC0597 (EMBL # LM655533), ARC0598 (EMBL # LM655534), same data as holotype; 2 exx, ARC2954 (EMBL # LM656007), ARC2955 (EMBL # LM656008), sample 2, S08°13.029', E114°29.466', 600 m, 12-XI-2007; 7 exx, sample 3, S08°13.325', E114°29.477', 500 m, 12-XI-2007.

##### Distribution.

Bali (Mt. Klatakan). Elevation: 500–600 m.

##### Etymology.

This epithet is based on the type locality, Mt. Klatakan.

##### Notes.

*Trigonopterus klatakanensis* Riedel, sp. n. was coded as “*Trigonopterus* sp. 285” by Tänzler et al. (2014).

#### 44. Trigonopterus lampungensis

Taxon classificationAnimaliaColeopteraCurculionidae

Riedel sp. n.

http://zoobank.org/81083C44-6CDD-4D45-BC51-2322506A0F43

##### Diagnostic description.

**Figure 44.**
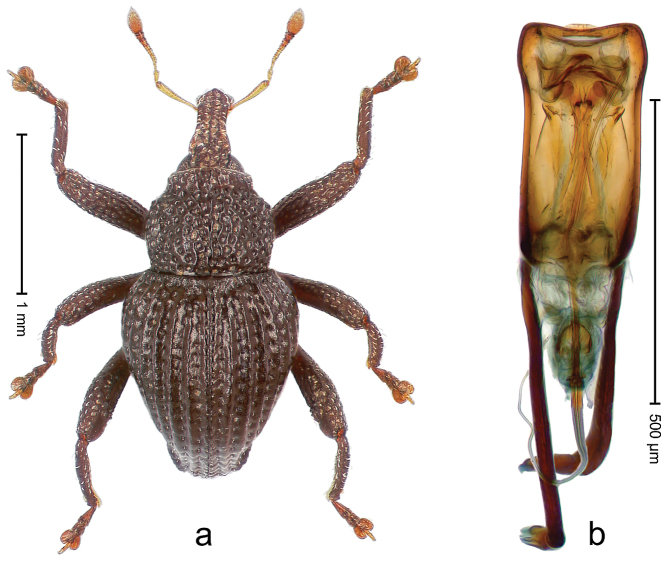
*Trigonopterus lampungensis* Riedel, sp. n., holotype; **a** Habitus **b** Penis.

Holotype, male (Fig. 44a). Length 2.06 mm. Color of antennae light ferruginous, remainder dark ferruginous to black. Body in dorsal aspect with marked constriction between pronotum and elytron; with distinct constriction in profile. Rostrum dorsally dull, rugulose; with median ridge and pair of indistinct submedian ridges, intervening furrows punctate, sparsely squamose with small suberect scales; epistome simple. Pronotum anterolaterally very weakly angularly projecting; with distinct subapical constriction; disk coarsely punctate, each puncture containing minute scale; interspaces dull, microreticulate; indistinct median ridge subglabrous. Elytra with striae deeply impressed, each with row of minute transparent scales; intervals costate to subcarinate, microreticulate; interval 7 swollen subapically, laterally projecting. Profemur edentate, meso- and metafemur ventrally with denticle. Metafemur subapically with stridulatory patch and transverse rows of denticles. Abdominal ventrite 5 with shallow impression. Penis (Fig. 44b) with body in profile markedly curved ventrad; basal half flattened, in dorsal aspect sides subparallel; towards apex slightly widened; apex with median, subtriangular extension; transfer apparatus flagelliform, ca. 1.3 × as long as body; apodemes 2.2 × as long as body; ductus ejaculatorius without bulbus. **Intraspecific variation.** Length 1.96–2.40 mm. Color of body black or ferruginous. Female rostrum dorsally subglabrous, with pairs of lateral and submedian furrows; epistome simple. Pronotum with anterolateral knobs indistinct or more distinctly projecting. Female abdominal ventrite 5 flat.

##### Material examined.

Holotype (MZB): ARC0266 (EMBL # LM655457), East Sumatra, Lampung Prov., Bawang, Pedada Bay, Mt. Tanggang, sample 2, S05°43.947', E105°06.480', 659 m, 09-VIII-2006. Paratypes (MZB, SMNK, ZSM): E-Sumatra, Lampung Prov.: 1 ex, ARC0267 (EMBL # LM655458), same data as holotype; 1 ex, ARC0274 (EMBL # LM655465), Bawang, Pedada Bay, Mt. Tanggang, sample 3, S05°43.938', E105°06.440', 673 m, 09-VIII-2006; 1 ex, Bawang, Pedada Bay, Mt. Tanggang, sample 4, S05°43.871', E105°06.393', 744 m, 09-VIII-2006; 1 ex, Bukit Barisan Selatan N.P., Kota Agung, Sukaraja, sample 5, S05°31.044', E104°25.664', 562 m, 02-V-2012; 1 ex, ARC2731 (EMBL # LM655987), Bukit Barisan Selatan N.P., Kota Agung, Sukaraja, sample 8, S05°33.032', E104°25.985', 421 m, 02-V-2012; 2 exx, ARC2732 (EMBL # LM655988), ARC2733 (EMBL # LM655989), Bukit Barisan Selatan N.P., Liwa, sample 1, S05°04.409', E104°03.265', 670 m, 04-V-2012; 1 ex, Bukit Barisan Selatan N.P., Liwa, sample 6, S05°04.753', E104°03.183', 809 m, 05-V-2012; 1 ex, ARC2734 (EMBL # LM655990), Bukit Barisan Selatan N.P., Liwa, sample 7, S05°04.958', E104°03.379', 813 m, 05-V-2012; 1 ex, Bukit Barisan Selatan N.P., Liwa, sample 8, S05°05.015', E104°03.442', 801 m, 05-V-2012.

##### Distribution.

Lampung Prov. (Bukit Barisan Selatan N.P., Pedada Bay). Elevation: 421–813 m.

##### Etymology.

This epithet is based on the Indonesian province of Lampung.

##### Notes.

*Trigonopterus lampungensis* Riedel, sp. n. was coded as “*Trigonopterus* sp. 301”.

#### 45. Trigonopterus latipes

Taxon classificationAnimaliaColeopteraCurculionidae

Riedel sp. n.

http://zoobank.org/8587BF5C-D65D-459D-8FC2-1C9145DB2E51

##### Diagnostic description.

**Figure 45.**
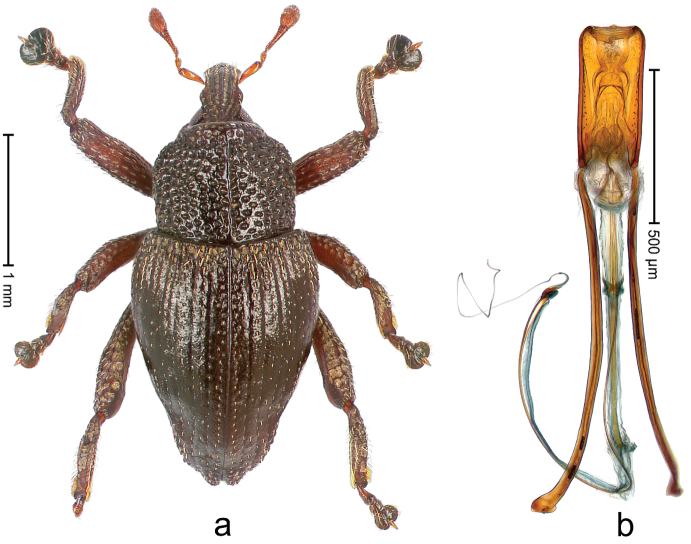
*Trigonopterus latipes* Riedel, sp. n., holotype; **a** Habitus **b** Penis.

Holotype, male (Fig. 45a). Length 2.86 mm. Color of legs and antennae ferruginous except protarsus black; remainder black. Body elongate; in dorsal aspect with marked constriction between pronotum and elytron; in profile dorsally flat, apically convex. Rostrum with median and pair of submedian ridges; intervening furrows each with sparse row of erect piliform scales; epistome simple. Pronotum laterally with subapical constriction; disk coarsely punctate to scabrous; submedially each puncture containing elongate white scale, towards sides with minute setae; with median ridge. Elytra with striae indistinct; intervals flat; surface dull, microreticulate, microgranulate; with sparse minute punctures and scattered lanceolate scales; interval 7 in apical third forming sharp lateral edge; sutural interval at apex swollen, forming pair of rounded apical protrusions. Femora edentate; anteroventral ridge crenulate. Metafemur subapically with stridulatory patch. Dorsal edge of pro- and metatibia subbasally simple, mesotibia subbasally with angulation extended as acute tooth. Tarsomere 3 of protarsus markedly larger than of mesotarsus. Abdominal ventrite 5 with shallow concave pit, apically with dense short suberect setae. Penis (Fig. 45b) with sides of body subparallel; apex medially pointed; transfer apparatus flagelliform, 4.0 × longer than body; apodemes 2.8 × as long as body; ductus ejaculatorius without bulbus. **Intraspecific variation.** Length 2.86–3.03 mm. Integument of males dull; females with punctures sparser and smaller, interspaces polished. Female rostrum with median and pair of submedian glabrous costae. Female abdominal ventrite 5 flat.

##### Material examined.

Holotype (MZB): ARC2466 (EMBL # LM655804), East Java Prov., Mt. Semeru, road Senduro - Ranupani, sample 4, S08°01.801', E112°59.931', 1430 m, 13-XI-2011. Paratypes (SMNK, ZSM): E-Java Prov., Mt. Semeru, road Senduro - Ranupani: 2 exx, ARC2467 (EMBL # LM655805), ARC2468, same data as holotype; 1 ex, sample 3, S08°02.052', E112°59.356', 1572 m, 13-XI-2011.

##### Distribution.

E-Java Prov. (Mt. Semeru). Elevation: 1430 m.

##### Etymology.

This epithet is based on a combination of the Latin adjective *latus* (wide) and the noun *pes* (foot) and refers to the unusual size of the protarsi.

##### Notes.

*Trigonopterus latipes* Riedel, sp. n. was coded as “*Trigonopterus* sp. 355”.

#### 46. Trigonopterus lima

Taxon classificationAnimaliaColeopteraCurculionidae

Riedel sp. n.

http://zoobank.org/C9E74D3C-2B07-490E-B60B-5B90FD9B52C6

##### Diagnostic description.

**Figure 46.**
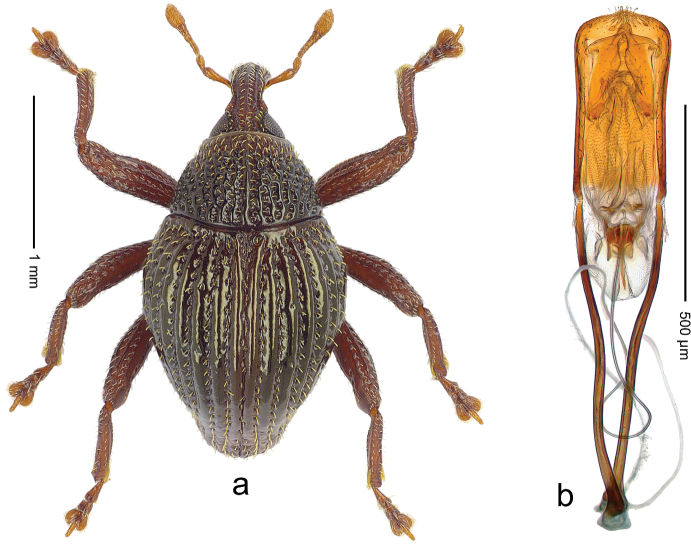
*Trigonopterus lima* Riedel, sp. n., holotype; **a** Habitus **b** Penis.

Holotype, male (Fig. 46a). Length 2.14 mm. Color of antennae, legs and head ferruginous; remainder dark ferruginous to black; elytra with slight bronze lustre. Body subovate, in dorsal aspect and in profile with weak constriction between pronotum and elytron. Rostrum with median ridge and pair of submedian ridges, intervening furrows each with sparse row of mesad directed scales; epistome simple. Pronotum coarsely punctate, laterally reticulate, dorsally interspaces longitudinally rugose; with median costa; with sparse, suberect, slender scales. Elytra with striae deeply impressed, each with row of slender suberect scales; intervals costate, subglabrous; incised suture bordered by row of squamiferous punctures, partly overgrown by costate sutural interval. Femora with simple anteroventral ridge. Metafemur subapically with stridulatory patch. Metatibia apically with uncus, without premucro. Abdominal ventrite 5 flat, coarsely punctate. Penis (Fig. 46b) with sides of body subparallel; apex broadly angulate; in apical half with pair of elongate sclerites converging to orifice; transfer apparatus small, symmetrical, with median spine projecting to apex of endophallus; apodemes 1.9 × as long as body; ductus ejaculatorius without bulbus. **Intraspecific variation.** Length 2.03–2.17 mm. Female rostrum in apical half dorsally subglabrous, with longitudinally confluent punctures; epistome simple.

##### Material examined.

Holotype (MZB): ARC2199 (EMBL # LM655655), East Nusa Tenggara Prov., Flores Isl., Ruteng, Mt. Ranaka, sample 3, S08°38.099', E120°31.745', summit area, 2205 m, 09-III-2011. Paratypes (MZB, SMNK): Flores Isl., Ruteng, Mt. Ranaka: 2 exx, ARC2579 (EMBL # LM655888), ARC2580 (EMBL # LM655889), sample 5, S08°38.277', E120°31.616', 2010 m, 09-III-2011; 1 ex, ARC2581 (EMBL # LM655890), sample 6, S08°38.243', E120°31.569', 1995 m, 09-III-2011; 2 exx, ARC2204 (EMBL # LM655660), sample 8, S08°37.781', E120°31.184', 1730 m, 10-III-2011.

##### Distribution.

East Nusa Tenggara Prov., Flores (Ruteng). Elevation: 1730–2205 m.

##### Etymology.

This epithet is based on the Indonesian word for “five” and is treated as a noun in apposition.

##### Notes.

*Trigonopterus lima* Riedel, sp. n. was coded as “*Trigonopterus* sp. 297”.

#### 47. Trigonopterus lombokensis

Taxon classificationAnimaliaColeopteraCurculionidae

Riedel sp. n.

http://zoobank.org/62A5DE3E-3F48-474D-B930-E7270FA08F42

##### Diagnostic description.

**Figure 47.**
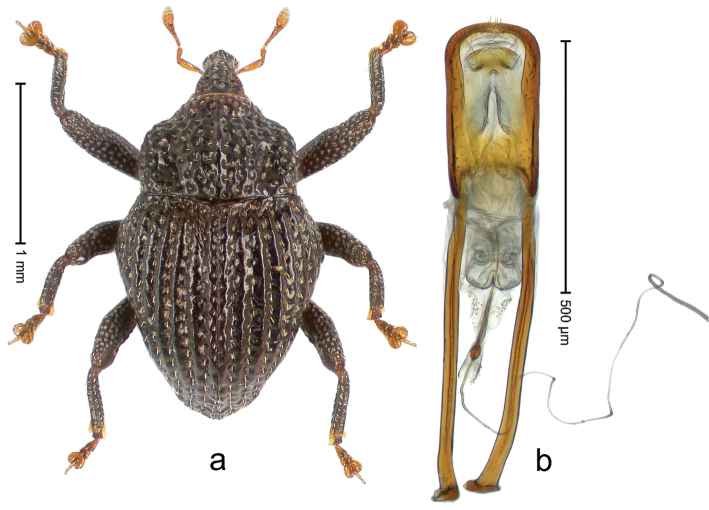
*Trigonopterus lombokensis* Riedel, sp. n., holotype; **a** Habitus **b** Penis.

Holotype, male (Fig. 47a). Length 2.01 mm. Color of antennae and tarsi ferruginous, remainder black. Body in dorsal aspect with marked constriction between pronotum and elytron; with distinct constriction in profile. Rostrum with pair of dorsolateral furrows, medially coarsely punctate-rugose; dorsal profile at middle with angulation; epistome with transverse, subangulate ridge. Pronotum with indistinct subapical constriction; disk coarsely punctate-rugose, sparsely setose; in basal half with pair of sublateral, kidney-shaped impressions; with indistinct median ridge. Elytra with striae deeply impressed, each with row of slender, suberect scales; intervals costate-carinate, subglabrous; apex narrow, rounded. Meso- and metafemur each with small tooth, profemur simple, relatively long. Metafemur subapically with stridulatory patch. Abdominal ventrite 5 subbasally at middle swollen; in apical half with round depression; coarsely punctate. Penis (Fig. 47b) with sides of body subparallel; apex rounded, medially with sparse setae; transfer apparatus flagelliform, subequal to body; apodemes 2.3 × as long as body; ductus ejaculatorius with indistinct bulbus. **Intraspecific variation.** Length 1.71–2.06 mm. Color of elytra ferruginous to black. Female rostrum dorsally in apical half subglabrous, with sparse minute punctures; epistome simple. Pronotum in females and in small males relatively narrow, in large males wider. Elytra in females narrower, humeri evenly rounded; large males with humeri more distinctly, subangularly projecting laterad, in small males similar to females; intervals weakly costate, in larger specimens markedly costate or carinate.

##### Material examined.

Holotype (MZB): ARC1458 (EMBL # LM655556), West Nusa Tenggara Prov., Lombok, Tetebatu, Rinjani-trail from Orong Gerisak, sample 6, S08°29.577', E116°24.782', 1195 m, 04-IV-2010. Paratypes (ARC, MZB, SMNK, ZSM): Lombok: 11 exx, ARC1459 (EMBL # LM655557), same data as holotype; 23 exx, Tetebatu, Rinjani-trail from Orong Gerisak, sample 5, S08°30.096', E116°25.062', 1010 m, 04-IV-2010; 13 exx, Tetebatu, Rinjani-trail from Orong Gerisak, sample 7, S08°29.433', E116°24.746', 1240 m, 04-IV-2010; 10 exx, ARC1476 (EMBL # LM655574), ARC1477 (EMBL # LM655575), ARC1478 (EMBL # LM655576), Tetebatu, Rinjani-trail from Orong Gerisak, sample 8, S08°29.173', E116°24.517', 1345 m, 04-IV-2010; 10 exx, N Tetebatu, 1200-1450 m, 07-XII-2004; 1 ex, ARC1483 (EMBL # LM655581), Sesaot, Rinjani-trail, sample 3, S08°29.841', E116°13.679', 625 m, 28-III-2010; 7 exx, ARC2260 (EMBL # LM655714), Senaru, Rinjani-track, sample 1, S08°19.429', E116°24.082', 900 m, 21-III-2011; 12 exx, Senaru, Rinjani-track, sample 2, S08°20.570', E116°23.969', 1320 m, 21-III-2011; 17 exx, Lombok, Senaru, Rinjani-track, sample 3, S08°20.780', E116°23.790', 1465 m, 21-III-2011; 48 exx, Lombok, Senaru, Rinjani-track, sample 4, S08°20.439', E116°24.047', 1240 m, 21-III-2011; 47 exx, ARC2267 (EMBL # LM655721), ARC2268 (EMBL # LM655722), Senaru, Rinjani-track, sample 5, S08°19.800', E116°24.107', 1015 m, 21-III-2011; 31 exx, Lombok, Senaru, Rinjani-track, sample 6, S08°19.359', E116°24.070', 860 m, 23-III-2011; 40 exx, Senaru, Rinjani-trail, sample 7, S08°19.643', E116°24.033', 935 m, 23-III-2011; 48 exx, Senaru, Rinjani-trail, sample 8, S08°19.719', E116°24.040', 955 m, 23-III-2011; 87 exx, ARC2279 (EMBL # LM655733), ARC2280 (EMBL # LM655734), Santong, Rinjani-track, sample 1, S08°20.715', E116°19.695', 830 m, 24-III-2011; 150 exx, Santong, Rinjani-track, sample 2, S08°20.813', E116°19.778', 870 m, 24-III-2011; 67 exx, Santong, Rinjani-track, sample 3, S08°20.996', E116°20.001', 960 m, 24-III-2011; 27 exx, Santong, Rinjani-track, sample 4, S08°21.160', E116°20.067', 1005 m, 25-III-2011; 28 exx, Santong, Rinjani-track, sample 5, S08°21.351', E116°20.208', 1105 m, 25-III-2011; 27 exx, Santong, Rinjani-track, sample 6, S08°21.754', E116°20.476', 1315 m, 25-III-2011.

##### Distribution.

West Nusa Tenggara Prov., Lombok (Santong, Senaru, Sesaot, Tetebatu). Elevation: 625–1345 m.

##### Etymology.

This epithet is based on the island of Lombok.

##### Notes.

*Trigonopterus lombokensis* Riedel, sp. n. was coded as “*Trigonopterus* sp. 323” by Tänzler et al. (2014).

#### 48. Trigonopterus merubetirensis

Taxon classificationAnimaliaColeopteraCurculionidae

Riedel sp. n.

http://zoobank.org/464CE6CB-6538-41EA-AC4E-96F71C087DD1

##### Diagnostic description.

**Figure 48.**
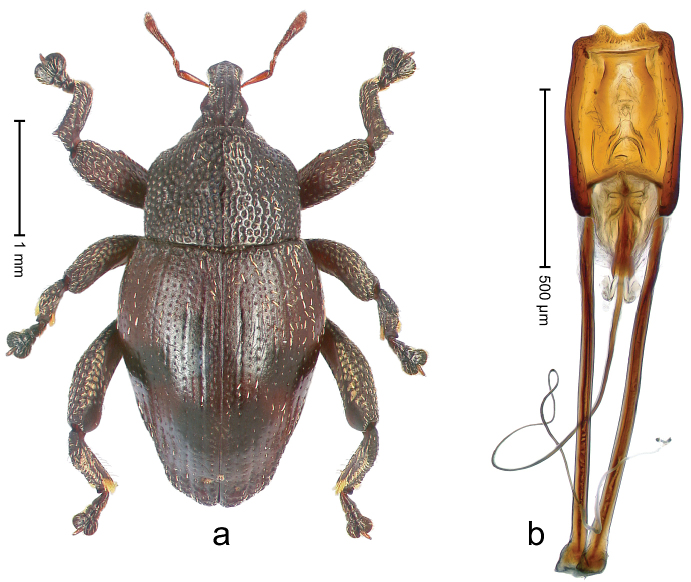
*Trigonopterus merubetirensis* Riedel, sp. n., holotype; **a** Habitus **b** Penis.

Holotype, male (Fig. 48a). Length 3.31 mm. Color of legs and head ferruginous, elytra deep ferruginous with indistinct transverse black band near middle and black mark near apex; remainder black. Body elongate; in dorsal aspect with marked constriction between pronotum and elytron; in profile dorsally flat, apically convex. Rostrum with median and pair of submedian ridges; intervening furrows with each with partly abraded row of erect piliform scales; epistome simple. Pronotum with indistinct subapical constriction; disk densely punctate. Elytra with striae indistinct; intervals flat; with small, confused punctures; especially basally with scattered elongate, recumbent, cream-colored scales; interspaces microreticulate; interval 7 in apical third forming sharp lateral edge; apex subtruncate, sutural interval not protruding. Anteroventral ridge of femora crenulate, forming blunt tooth. Metafemur subapically with stridulatory patch. Dorsal edge of tibiae subbasally with angulation extended as acute tooth in pro- and mesotibia. Abdominal ventrite 5 with shallow concave pit laterally bordered by ridges. Penis (Fig. 48b) with sides of body subparallel; apex bidentate, with median notch; transfer apparatus spiniform, ca. as long as body; apodemes 2.3 × as long as body; ductus ejaculatorius without bulbus. **Intraspecific variation.** Length 2.88–3.31 mm. Color of elytra with black marking more or less extensive, sometimes with apical half almost completely black. Female rostrum with median and pair of submedian glabrous costae. Female abdominal ventrite 5 flat.

##### Material examined.

Holotype (MZB): ARC2332 (EMBL # LM655785), East Java Prov., Banyuwangi Reg., Meru Betiri N.P., Sukamade, sample 2, S08°32.221', E113°51.985', 260 m, 12/13-IV-2011. Paratypes (MZB, SMNK, ZSM): E-Java Prov., Banyuwangi Reg., Meru Betiri N.P., Sukamade: 5 exx, ARC2333 (EMBL # LM655786), same data as holotype; 2 exx, ARC2334 (EMBL # LM655787), ARC2335 (EMBL # LM655788), sample 4, S08°32.163', E113°51.904', 340 m, 13-IV-2011.

##### Distribution.

E-Java Prov. (Meru Betiri N.P.). Elevation: 260–340 m.

##### Etymology.

This epithet is based on the type locality, Meru Betiri National Park.

##### Notes.

*Trigonopterus merubetirensis* Riedel, sp. n. was coded as “*Trigonopterus* sp. 346”.

#### 49. Trigonopterus mesehensis

Taxon classificationAnimaliaColeopteraCurculionidae

Riedel sp. n.

http://zoobank.org/1576F335-48DA-4B33-9E51-856FD868D2C7

##### Diagnostic description.

**Figure 49.**
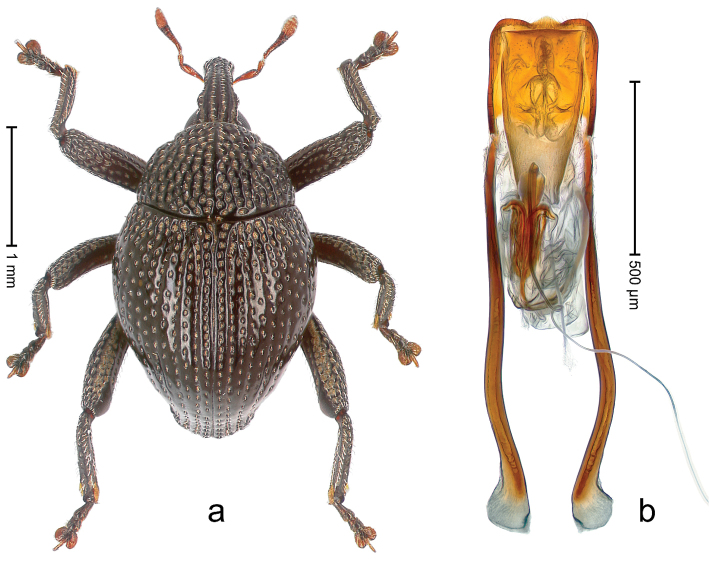
*Trigonopterus mesehensis* Riedel, sp. n., holotype; **a** Habitus **b** Penis.

Holotype, male (Fig. 49a). Length 2.70 mm. Color of antennae ferruginous; legs dark ferruginous; remainder black. Body subovate, in dorsal aspect with weak constriction between pronotum and elytron; in profile dorsally convex. Rostrum with median ridge and pair of submedian ridges, intervening furrows each with sparse row of mesad directed scales; epistome with irregular transverse ridge. Pronotum coarsely punctate, reticulate-costate, with median costa; with sparse, recumbent scales. Elytra with dense, confused punctation; intervals flat, each with row of punctures; each puncture containing small recumbent scale; elytral apex subtruncate. Femora with simple anteroventral ridge, crenate in metafemur. Metafemur subapically with stridulatory patch. Abdominal ventrite 5 coarsely punctate, with sparse suberect scales. Penis (Fig. 49b) with sides of body subparallel; apex with median angulate extension; transfer apparatus with flagelliform median rod forming almost full coil; median rod projecting basad further than apicad, ca 2.7 × as long as body; apodemes 3.4 × as long as body; ductus ejaculatorius without bulbus. **Intraspecific variation.** Length 2.11–2.88 mm. Color of elytra dark ferruginous to black. Female rostrum in apical half slender, dorsally subglabrous, with submedian rows of punctures, laterally punctate-rugose; epistome simple.

##### Material examined.

Holotype (MZB): ARC2250 (EMBL # LM655705), Bali, Negara, Kampung Pasatan, Desa Pohsanten, Mt. Mesehe, sample 2, S08°17.299', E114°41.494', 460 m, 17-III-2011. Paratypes (MZB, SMNK, ZSM): Bali, Negara, Kampung Pasatan, Desa Pohsanten, Mt. Mesehe: 5 exx, ARC2251 (EMBL # LM655706), same data as holotype; 6 exx, ARC2248 (EMBL # LM655703), ARC2249 (EMBL # LM655704), sample 1, S08°17.369', E114°41.392', 550 m, 17-III-2011; 15 exx, sample 3, S08°17.403', E114°41.424', 515 m, 17-III-2011; 5 exx, sample 4, S08°17.466', E114°41.331', 490 m, 17-III-2011; 1 ex, sample 6, S08°16.234', E114°41.528', 935 m, 18-III-2011; 3 exx, ARC2247 (EMBL # LM655702), S08°16.454', E114°41.725', 840 m, hand-collected from foliage, 18-III-2011; 26 exx, ARC0727, ARC0728, ARC0729, ARC0732 (PCR failed), trail to Mesehe waterfall, S08°18.30', E114°41.68', 350–600 m, 14.&17.XII.2008; 8 exx, ARC2962 (EMBL # LM656015), sample 8, S08°16.454', E114°41.725', 825 m, 18-III-2011.

##### Distribution.

Bali (Mt. Mesehe). Elevation: 460–840 m.

##### Etymology.

This epithet is based on the type locality Mt. Mesehe.

##### Notes.

*Trigonopterus mesehensis* Riedel, sp. n. was coded as “*Trigonopterus* sp. 334” by Tänzler et al. (2014).

#### 50. Trigonopterus micans

Taxon classificationAnimaliaColeopteraCurculionidae

Riedel sp. n.

http://zoobank.org/16236EF7-BDF5-41DC-9F19-A8334D7A4363

##### Diagnostic description.

**Figure 50.**
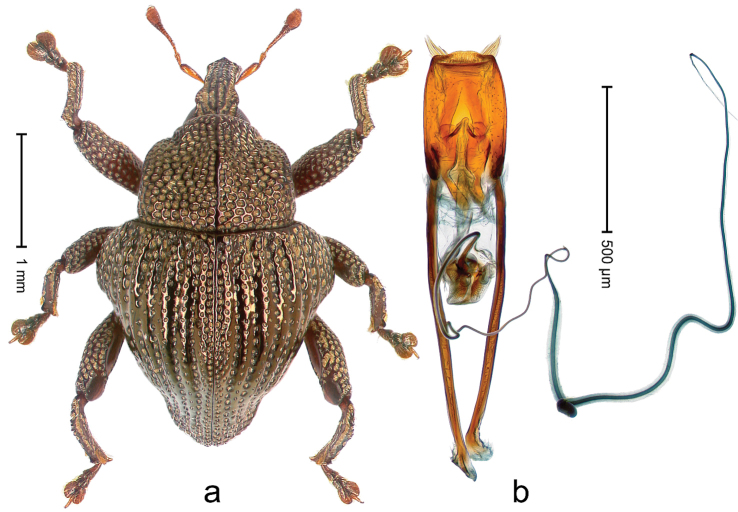
*Trigonopterus micans* Riedel, sp. n., holotype; **a** Habitus **b** Penis.

Holotype, male (Fig. 50a). Length 2.97 mm. Color of legs and antennae ferruginous; pronotum and elytron reddish coppery; remainder dark ferruginous to black. Body in dorsal aspect with marked constriction between pronotum and elytron; in profile with weak constriction. Rostrum with median and pair of indistinct submedian ridges; intervening furrows each with row of coarse punctures and suberect elongate scales; epistome with indistinct transverse ridge forming small median denticle. Pronotum with sides subparallel; anteriorly rounded to subapical constriction; disk coarsely punctate, reticulate; with sparse recumbent, elongate, cream-colored scales, especially anteriorly; medially swollen, with median ridge. Elytra with humeri markedly swollen, laterally projecting; striae distinct, basally deeply impressed; intervals weakly costate, punctate; sutural interval in basal half distinctly swollen; punctures near base coarse and dense, near middle sparser with interspaces polished; each puncture containing minute, inconspicuous seta. Profemur with anteroventral ridge indistinct; in meso- and metafemur forming large, acute tooth. Metafemur subapically with stridulatory patch. Dorsal tibial edge subbasally with angulation. Thoracic and abdominal venter setose with long erect setae. Abdominal ventrite 5 weakly concave, densely punctate-rugose, with dense erect setae. Penis (Fig. 50b) subapically converging, with pair of setose brushes; subtruncate at middle; transfer apparatus small, compact; apodemes 2.7 × as long as body; ductus ejaculatorius with bulbus. **Intraspecific variation.** Length 2.20–2.97 mm. Color of pronotum and elytron ranging from dark bronze to a more vivid coppery. Female body more slender, humeri less prominent. Female rostrum dorsally punctate-rugose, with median and pair of submedian subglabrous costae; epistome simple.

##### Material examined.

Holotype (MZB): ARC2213 (EMBL # LM655669), East Nusa Tenggara Prov., Flores, Labuhan Bajo, Roe, earthworm-dominated habitat, sample 1, S08°36.540', E120°01.871', 955 m, 13-III-2011. Paratypes (MZB, SMNK, ZSM): 3 exx, ARC2214 (EMBL # LM655670), ARC2216 (EMBL # LM655672), same data as holotype; 3 exx, ARC2220 (EMBL # LM655676), ARC2221 (EMBL # LM655677), ARC2222 (EMBL # LM655678), Flores, Labuhan Bajo, Roe, sample 5, S08°35.395', E120°00.383', 790 m, 13-III-2011; 1 ex, ARC2230 (EMBL # LM655686), Flores, Labuhan Bajo, Roe, sample 3, S08°36.259', E120°01.539', 975 m, 13-III-2011.

##### Distribution.

East Nusa Tenggara Prov., Flores (Labuhan Bajo). Elevation: 790–975 m.

##### Etymology.

This epithet is based on the Latin adjective *micans* (shining) and refers to the metallic coloration of this species.

##### Notes.

*Trigonopterus micans* Riedel, sp. n. was coded as “*Trigonopterus* sp. 348” by Tänzler et al. (2014).

#### 51. Trigonopterus misellus

Taxon classificationAnimaliaColeopteraCurculionidae

Riedel sp. n.

http://zoobank.org/2355CE1B-8EDA-4826-9A9F-826D9B7FFBB1

##### Diagnostic description.

**Figure 51.**
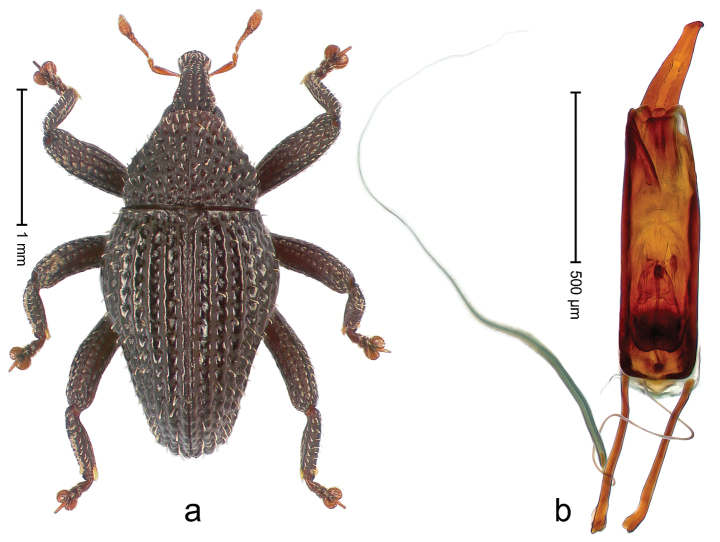
*Trigonopterus misellus* Riedel, sp. n., holotype; **a** Habitus **b** Penis.

Holotype, male (Fig. 51a). Length 2.65 mm. Color of tarsi and antennae ferruginous; remainder black. Body elongate subovate; in dorsal aspect and in profile with distinct constriction between pronotum and elytron. Rostrum in apical half scabrous, in basal half with median and pair of submedian ridges; with elongate, clavate, suberect scales; epistome with transverse, angulate ridge. Pronotum with indistinct subapical constriction; disk coarsely punctate; with median ridge; each puncture containing erect clavate scale inserting at posterior rim; surface dull, microreticulate. Elytra elongate; in profile with slight depression behind middle; with striae deeply impressed, in front of each puncture with claviform, suberect scale; intervals costate, subglabrous. Femora edentate. Metafemur subapically without stridulatory patch. Abdominal ventrite 5 flat, microreticulate, with sparse scales. Penis (Fig. 51b) with apex asymmetrically extended to the left; apical extension long; basal orifice ventrally with rim; apodemes short, 0.5 × as long as body; ductus ejaculatorius with bulbus. **Intraspecific variation.** Length 2.30–2.65 mm. Female rostrum dorsally in apical half subglabrous, with small punctures; epistome simple. Profile of elytral apex in females more slender than in males, more distinctly curved ventrad.

##### Material examined.

Holotype (MZB): ARC0263 (EMBL # LM655454), E-Sumatra, Lampung Prov., Bawang, Pedada Bay, Mt. Tanggang, sample 1, S05°43.933', E105°06.598', 579 m, 09-VIII-2006. Paratypes (MZB, SMNK, ZSM): E-Sumatra, Lampung Prov., Bawang, Pedada Bay, Mt. Tanggang: 2 exx, ARC0264 (EMBL # LM655455), ARC0265 (EMBL # LM655456), same data as holotype; 2 exx, sample 2, S05°43.947', E105°06.480', 659 m, 09-VIII-2006.

##### Distribution.

Lampung Prov. (Pedada Bay). Elevation: 579–744 m.

##### Etymology.

This epithet is based on the Latin adjective *misellus* (poor).

##### Notes.

*Trigonopterus misellus* Riedel, sp. n. was coded as “*Trigonopterus* sp. 336”.

#### 52. Trigonopterus palawanensis

Taxon classificationAnimaliaColeopteraCurculionidae

Riedel sp. n.

http://zoobank.org/E0A4D64A-3F49-44AE-BB6C-1BB40C39CA73

##### Diagnostic description.

**Figure 52.**
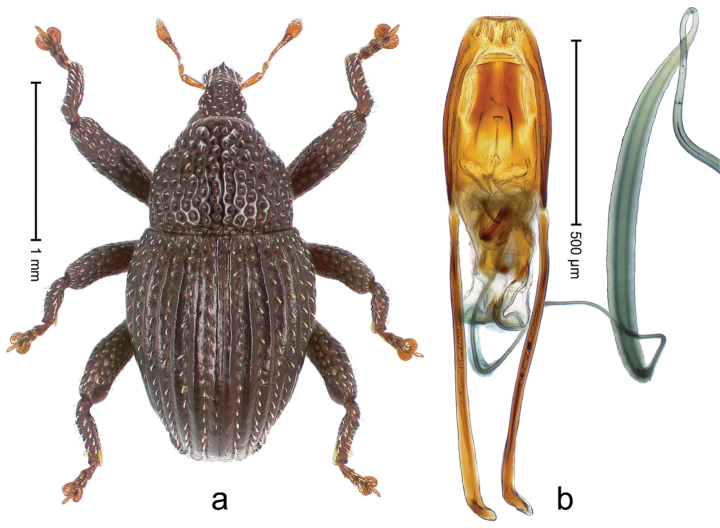
*Trigonopterus palawanensis* Riedel, sp. n., holotype; **a** Habitus **b** Penis.

Holotype, male (Fig. 52a). Length 2.21 mm. Color of antennae and tarsi light ferruginous; remainder dark ferruginous to black. Body subovate; in dorsal aspect with marked constriction between pronotum and elytron; with distinct constriction in profile. Rostrum with median and pair of submedian ridges, most prominent at level of antennal insertion, anteriorly rather indistinct; with sparse rows of suberect scales; epistome at middle with dorsoposteriad directed acute denticle. Pronotum coarsely punctate-reticulate, each puncture with one clavate suberect scale; with indistinct subapical constriction. Elytra with striae deeply impressed, each with row of suberect clavate scales; intervals subcarinate, microreticulate. Metafemur subapically with stridulatory patch. Abdominal ventrite 5 flat, sparsely covered with small clavate scales. Penis (Fig. 52b) with sides of body in basal half subparallel, in apical half converging to subtruncate apex; ostium with large trapeziform sclerite; apodemes 1.8 × as long as body; transfer apparatus with subovate central fig and coiled spiniform transfer process; ductus ejaculatorius with distinct bulbus. **Intraspecific variation.** Length 2.13–2.21 mm. Female rostrum in basal half with median and pair of submedian ridges less prominent, but distinct; in apical half rugose-punctate; sparse scales recumbent; epistome simple.

##### Material examined.

Holotype (SMNK): ARC1440 (EMBL # LM655554), PHILIPPINES, Palawan, Puerto Princessa region, Sabang, Mt. Bloomfield, N10°11.62', E118°52.35', 500-700 m, 10-XII-2009. Paratypes (SMNK): 2 exx, ARC1441 (EMBL # LM655555), ARC1442 (PCR failed), same data as holotype.

##### Distribution.

Palawan (Mt. Bloomfield). Elevation: ca. 500–700 m.

##### Etymology.

This epithet is based on the type locality, the island of Palawan.

##### Notes.

*Trigonopterus palawanensis* Riedel, sp. n. was coded as “*Trigonopterus* sp. 344”.

#### 53. Trigonopterus pangandaranensis

Taxon classificationAnimaliaColeopteraCurculionidae

Riedel sp. n.

http://zoobank.org/5E7CA176-4FF1-470A-81A5-048027269E7A

##### Diagnostic description.

**Figure 53.**
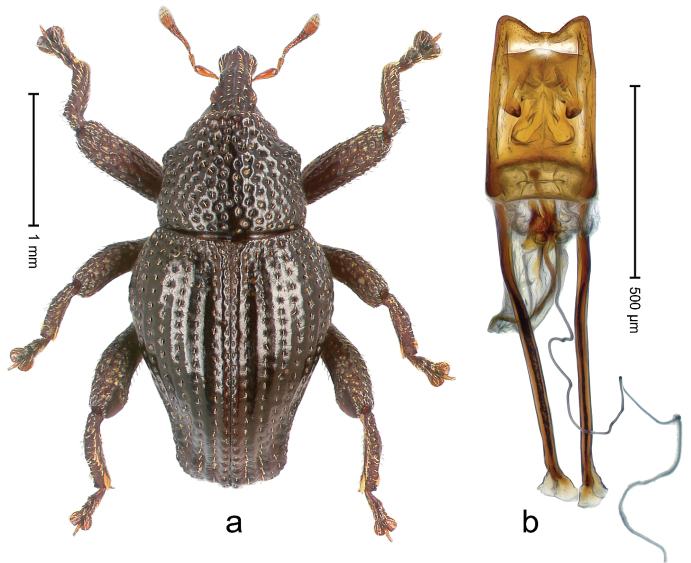
*Trigonopterus pangandaranensis* Riedel, sp. n., holotype; **a** Habitus **b** Penis.

Holotype, male (Fig. 53a). Length 2.95 mm. Color of legs and head ferruginous, remainder black. Body elongate; in dorsal aspect with marked constriction between pronotum and elytron; in profile dorsally without constriction. Rostrum dorsally microreticulate; with median carina terminating on forehead; with pair of submedian ridges; intervening furrows each with sparse row of erect scales; epistome with transverse, angulate ridge forming distinct median denticle. Pronotum anterolaterally subangularly projecting; without distinct subapical constriction; disk coarsely punctate; each punctures containing recumbent scale, subapically with suberect scale; without median ridge. Elytra with striae deeply impressed; each with row of small recumbent scales; intervals almost flat; interval 7 swollen subapically, laterally weakly projecting; apex extended ventrad, beak-shaped. Meso- and metafemur with crenulate anteroventral ridge terminating with blunt tooth. Metafemur subapically with stridulatory patch and transverse row of denticles. Abdominal ventrite 5 subapically with shallow pit. Penis (Fig. 53b) with body in profile weakly curved ventrad; in dorsal aspect sides subparallel; apex with subangulate notch; transfer apparatus thick flagelliform, ca. as long as body; apodemes 1.9 × as long as body; ductus ejaculatorius without bulbus. **Intraspecific variation.** Length 2.65–2.98 mm. Body of females slender; males slightly wider, especially between humeri. Female rostrum dorsally with pair of lateral furrows, pair of submedian furrows continued apicad by coarse punctures; epistome simple. Female abdominal ventrite 5 flat.

##### Material examined.

Holotype (MZB): ARC2682 (EMBL # LM655940), West Java Prov., Ciamis, Pangandaran, sample 2, S07°42.836', E108°39.478', 157 m, 22-IV-2012. Paratypes (SMNK, ZSM): W-Java Prov., Ciamis, Pangandaran: 3 exx, ARC2683 (EMBL # LM655941), same data as holotype; 3 exx, ARC2686 (EMBL # LM655944), ARC2687 (EMBL # LM655945), ARC3637 (EMBL # LM656059), sample 3, S07°43.070', E108°39.634', 156 m, 22-IV-2012.

##### Distribution.

W-Java Prov. (Pangandaran). Elevation: 156–157 m.

##### Etymology.

This epithet is based on the type locality, Pangandaran.

##### Notes.

*Trigonopterus pangandaranensis* Riedel, sp. n. was coded as “*Trigonopterus* sp. 372”.

#### 54. Trigonopterus paraflorensis

Taxon classificationAnimaliaColeopteraCurculionidae

Riedel sp. n.

http://zoobank.org/6821B9F3-461A-48F2-A1E2-EAEE65AF0BAB

##### Diagnostic description.

**Figure 54.**
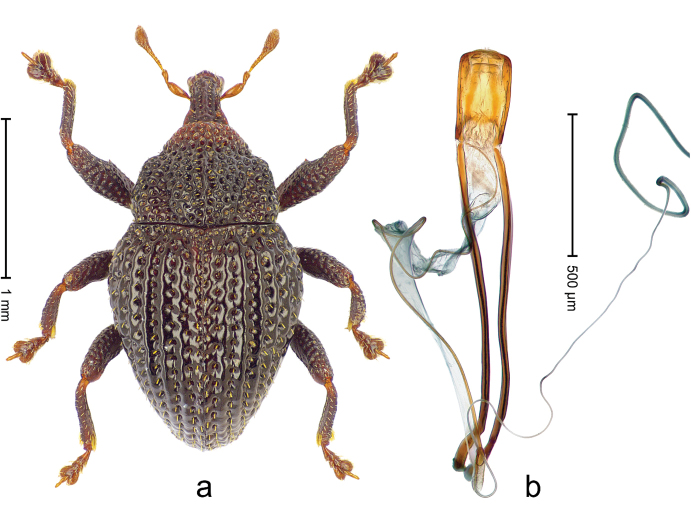
*Trigonopterus paraflorensis* Riedel, sp. n., holotype; **a** Habitus **b** Penis.

Holotype, male (Fig. 54a). Length 2.06 mm. Color of head and legs ferruginous, remainder black. Body in dorsal aspect with marked constriction between pronotum and elytron; with indistinct constriction in profile. Rostrum with median ridge and pair of submedian ridges, anteriorly coarsely punctate-scabrous; epistome with transverse, subangulate ridge. Pronotum with subapical constriction; disk coarsely punctate-reticulate, sparsely setose; with pair of curved sublateral impressions; medially swollen. Elytra relatively slender; with striae deeply impressed, each with row of short suberect bristles; intervals costate, subglabrous; apex narrow, rounded. Femora each with small tooth. Metafemur subapically with stridulatory patch. Abdominal ventrite 5 coarsely punctate, with median impression bordered by pair of weak longitudinal costae. Penis (Fig. 54b) with sides subparallel, in apical third markedly converging to narrow subglabrous apex; transfer apparatus flagelliform, 6.6 × as long as body; apodemes 4.0 × as long as body; ductus ejaculatorius without bulbus. **Intraspecific variation.** Length 1.70–2.20 mm. Penis with flagelliform transfer apparatus 6.0–6.9 × as long as body; apodemes 3.4–4.1 × as long as body. No females available.

##### Material examined.

Holotype (MZB): ARC3622 (EMBL # LM656052), East Nusa Tenggara Prov., Flores, Ruteng, Mt. Ranaka, sample 7, S08°37.781', E120°31.184', 1730 m, 10-III-2011. Paratypes (MZB, SMNK): Flores: 4 exx, ARC2205 (EMBL # LM655661), ARC2206 (EMBL # LM655662), ARC3623 (EMBL # LM656053), ARC3624 (EMBL # LM656054), same data as holotype.

##### Distribution.

East Nusa Tenggara Prov., Flores (Mt. Ranaka). Elevation: 1730 m.

##### Etymology.

This epithet is based on a combination of the Greek prefix *para*- (next to; near by) and the specific epithet of *Trigonopterus florensis* Riedel, sp. n., a sibling species.

##### Notes.

*Trigonopterus paraflorensis* Riedel, sp. n. was coded as “*Trigonopterus* sp. 441”. Morphologically it is very similar to *Trigonopterus florensis* Riedel, sp. n. and *Trigonopterus pseudoflorensis* Riedel, sp. n. but its cox1 sequences differ 7.75%, respectively 7.14% smallest interspecific *p*-distance.

#### 55. Trigonopterus pararugosus

Taxon classificationAnimaliaColeopteraCurculionidae

Riedel sp. n.

http://zoobank.org/CEE5CBA1-54A0-4363-9D9D-CC54034D59E9

##### Diagnostic description.

**Figure 55.**
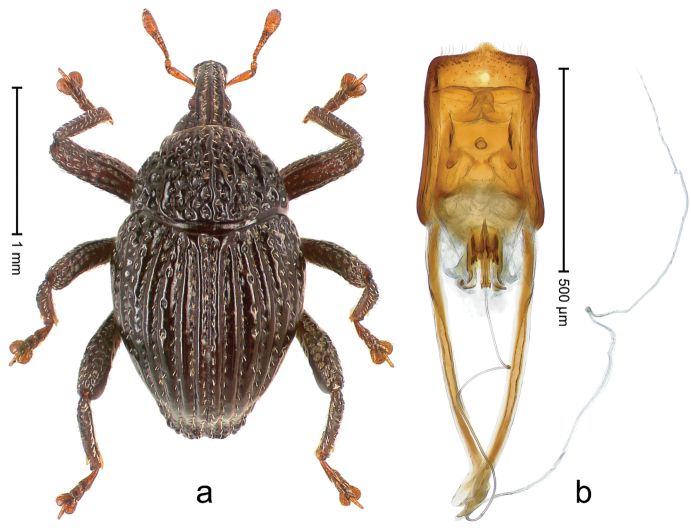
*Trigonopterus pararugosus* Riedel, sp. n., holotype; **a** Habitus **b** Penis.

Holotype, male (Fig. 55a). Length 2.16 mm. Color of antennae and legs ferruginous, remainder black. Body subovate, in dorsal aspect with weak constriction between pronotum and elytron; with distinct constriction in profile. Rostrum with median ridge and pair of submedian ridges, intervening furrows each with sparse row of mesad directed scales; epistome with irregular transverse ridge. Pronotum without subapical constriction; disk with coarse irregular ridges and tubercles, with sparse, suberect scales. Elytra with striae deeply impressed, each with sparse row of slender, suberect scales; intervals costate, intervals 3 and 5 with row of punctures, others subglabrous; sutural interval basally swollen and widened laterad; suture incised, bordered by rows of suberect scales; elytral apex scabrous, margin rounded, without apical denticle. Femora with simple anteroventral ridge, in meso- and metafemur crenate. Metafemur subapically with stridulatory patch. Abdominal ventrite 5 dull, with T-shape ridge along base and middle, coarsely punctate, with sparse erect scales. Penis (Fig. 55b) with sides of body weakly concave; apical edge subangulate, with sparse fringe of setae; apex with rounded median extension; transfer apparatus compact; apodemes 2.0 × as long as body; ductus ejaculatorius without bulbus. **Intraspecific variation.** Length 2.16–2.38 mm. Color of elytra completely black, with ferruginous sutural interval, or entirely ferruginous. Female rostrum in apical third dorsally subglabrous, sparsely punctate; epistome simple. Female elytra ventroapically with pair of laterally flattened denticles, male elytra ventroapically simple; elytral apex in Mesehe-specimen with median suture incised. Female abdominal ventrite 5 medially with weak ridge.

##### Material examined.

Holotype (MZB): ARC0585 (EMBL # LM655521), Bali, Bedugul, Tamblingan, above the village along water pipe, sample 4, S08°15.854', E115°05.312', 1310 m, 06-XI-2007. Paratypes (MZB, SMNK, ZSM): Bali: 2 exx, ARC0584 (EMBL # LM655520), same data as holotype; 2 exx, ARC3586 (EMBL # LM656019), ARC3587, Tamblingan, sample 2, S08°15.015', E115°06.202', 1255 m, 06-XI-2007; 2 exx, ARC2315 (EMBL # LM655768), ARC2316 (EMBL # LM655769), Tamblingan, sample 5, S08°15.108', E115°05.573', 1350 m, 03-IV-2011; 1 ex, ARC2306 (EMBL # LM655759), Mt. Batukaru, Wangayagede, sample 3, S08°21.431', E115°05.699', 1170 m, 31-III-2011; 2 exx, ARC2310 (EMBL # LM655763), ARC2311 (EMBL # LM655764), Mt. Batukaru, Wangayagede, sample 5, S08°21.130', E115°05.321', 1520 m, 31-III-2011; 1 ex, ARC2255 (EMBL # LM655709), Negara, Kampung Pasatan, Desa Pohsanten, Mt. Mesehe, sample 8, S08°16.454', E114°41.725', 825 m, 18-III-2011, sifted.

##### Distribution.

Bali (Mt. Batukaru, Mt. Mesehe, Tamblingan). Elevation: 825–1520 m.

##### Etymology.

This epithet is based on a combination of the Greek prefix *para*- (next to; near by) and the specific epithet of *Trigonopterus rugosus* Riedel, sp. n., a sibling species.

##### Notes.

*Trigonopterus pararugosus* Riedel, sp. n. was coded as “*Trigonopterus* sp. 327” by Tänzler et al. (2014). The interspecific *p*-distance of cox1 to *Trigonopterus rugosus* Riedel, sp. n. is 5.6–7.2%. Morphologically no clear differences could be detected and it is assumed that both represent a pair of cryptic species.

#### 56. Trigonopterus parasumbawensis

Taxon classificationAnimaliaColeopteraCurculionidae

Riedel sp. n.

http://zoobank.org/78A3D61F-7402-40EE-B6F7-3AB5062CF801

##### Diagnostic description.

**Figure 56.**
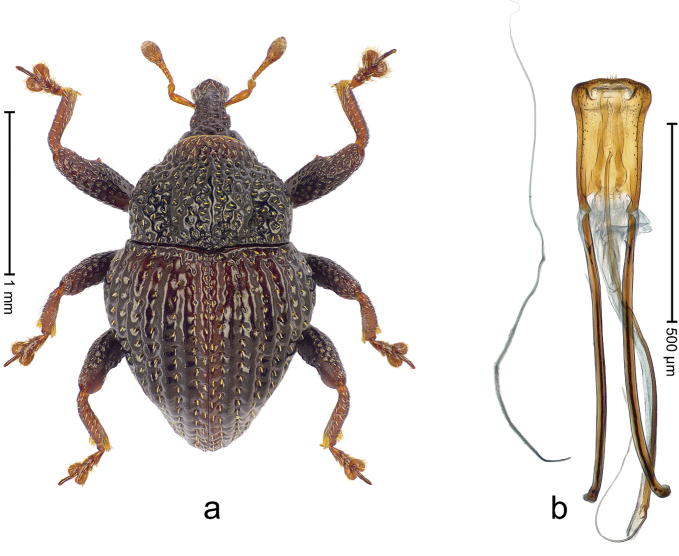
*Trigonopterus parasumbawensis* Riedel, sp. n., holotype; **a** Habitus **b** Penis.

Holotype, male (Fig. 56a). Length 1.85 mm. Color of antennae, tibiae and tarsi ferruginous, elytra dark ferruginous, remainder black. Body in dorsal aspect with marked constriction between pronotum and elytron; with distinct constriction in profile. Rostrum with median ridge and pair of submedian ridges, median ridge terminating before apex; epistome with transverse, subangulate ridge. Pronotum with indistinct subapical constriction; disk coarsely punctate-rugose, sparsely setose; in basal half with pair of sublateral, kidney-shaped impressions; with indistinct median ridge. Elytra with striae deeply impressed, each with row of short, suberect scales; intervals costate, subglabrous; apex rounded. Femora with small tooth; metafemur subapically with stridulatory patch. Abdominal ventrite 5 flat, coarsely punctate. Penis (Fig. 56b) with sides of body subparallel; in apical third widening and rounded to apex, medially with sparse setae; transfer apparatus flagelliform, 3.3 × longer than body; apodemes 2.5 × as long as body. **Intraspecific variation.** Length 1.56–1.90 mm. Female rostrum dorsally subglabrous, sparsely punctate, with dorsolateral pair of furrows. Contour of elytral humeri subangulate in males, rounded in females.

##### Material examined.

Holotype (MZB): ARC3603 (EMBL # LM656033), West Nusa Tenggara Prov., Sumbawa, Batu Dulang, Gn. Batu Pasak, sample 3, S08°37.524', E117°15.423', 1385 m, 12-IV-2010. Paratypes (MZB, SMNK, ZSM): West Nusa Tenggara Prov., Sumbawa: 5 exx, ARC3641 (EMBL # LM656062), ARC3642 (EMBL # LM656063), ARC3644 (EMBL # LM656065), ARC3645 (EMBL # LM656066), same data as holotype; 1 ex, ARC3649 (EMBL # LM656068), Batu Dulang, Gn. Batu Pasak, sample 3, S08°37.524', E117°15.423', 1385 m, 18-IV-2010; 1 ex, ARC3656 (EMBL # LM656074), Batu Dulang, Gn. Batu Pasak, sample 5, S08°37.005', E117°15.790', 1320 m, 18-IV-2010.

##### Distribution.

West Nusa Tenggara Prov., Sumbawa (Batu Dulang). Elevation: 1385 m.

##### Etymology.

This epithet is based on the Greek prefix *para*- (next to; near by) and the name of *Trigonopterus sumbawensis* Riedel, sp. n., a sibling species.

##### Notes.

*Trigonopterus parasumbawensis* Riedel, sp. n. was coded as “*Trigonopterus* sp. 443”.

#### 57. Trigonopterus pauxillus

Taxon classificationAnimaliaColeopteraCurculionidae

Riedel sp. n.

http://zoobank.org/23CE266B-C145-4887-9B05-2F63308A4BA2

##### Diagnostic description.

**Figure 57.**
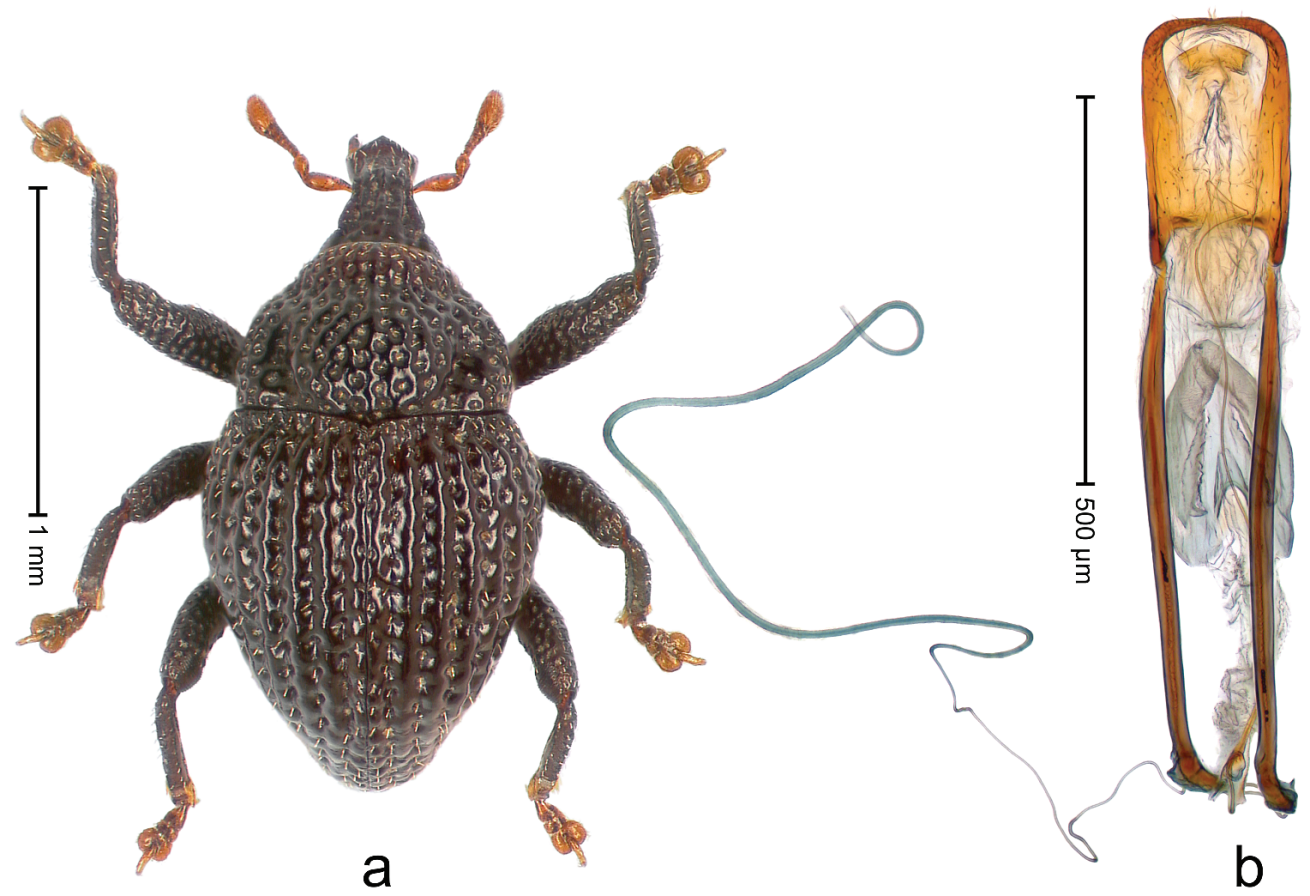
*Trigonopterus pauxillus* Riedel, sp. n., holotype; **a** Habitus **b** Penis.

Holotype, male (Fig. 57a). Length 1.65 mm. Color of antennae and tarsi ferruginous, remainder black. Body in dorsal aspect with marked constriction between pronotum and elytron; with distinct constriction in profile. Rostrum in basal half with median ridge and pair of submedian ridges, in apical half flat; epistome with transverse, subangulate ridge. Pronotum with indistinct subapical constriction; disk coarsely punctate-rugose, sparsely setose; in basal half with pair of sublateral, curved impressions. Elytra with striae deeply impressed, each with row of short, suberect setae; intervals costate, subglabrous; apex rounded. Meso- and metafemur with small tooth; profemur, simple. Metafemur subapically with stridulatory patch. Abdominal ventrite 5 flat, coarsely punctate. Penis (Fig. 57b) with sides of body subparallel, in apical third rounded to apex, subglabrous; transfer apparatus flagelliform, thin, 3.1 × as long as body; apodemes 2.4 × as long as body; ductus ejaculatorius with indistinct bulbus. **Intraspecific variation.** Length 1.65–2.01 mm. Coloration dark as in holotype, or partly deep ferruginous, especially along elytral suture. Female rostrum dorsally subglabrous, sparsely punctate, with dorsolateral pair of furrows. Pronotum with impressions in basal half simple or kidney-shaped; disk with or without median ridge. Elytral intervals more or less distinctly costate-carinate. Profemur with or without tooth.

##### Material examined.

Holotype (MZB): ARC2340 (EMBL # LM655790), West Nusa Tenggara Prov., Sumbawa, Tepal, Pc. Nengas, sample 7, S08°35.176', E117°08.295', 1490 m, 16-IV-2010. Paratypes (MZB, SMNK, ZSM): West Nusa Tenggara Prov., Sumbawa: 3 exx, ARC1534 (EMBL # LM655632), ARC2342 (EMBL # LM655792), ARC2344 (EMBL # LM655794), same data as holotype; 2 exx, ARC1514 (EMBL # LM655612), ARC1515 (EMBL # LM655613), Batu Dulang, Mt. Batu Pasak, sample 2, S08°37.028', E117°15.783', 1305 m, 12-IV-2010; 1 ex, ARC3652 (EMBL # LM656070), Batu Dulang, Mt. Batu Pasak, sample 4, S08°37.318', E117°15.339', 1280 m, 18-IV-2010; 1 ex, ARC3612 (EMBL # LM656042), Tepal, Pc. Nengas, sample 3, S08°35.386', E117°08.251', 1415 m, 15-IV-2010; 2 exx, ARC3605 (EMBL # LM656035), ARC3607 (EMBL # LM656037), Tepal, Pc. Nengas, sample 2, S08°35.884', E117°08.384', 1310 m, 15-IV-2010.

##### Distribution.

West Nusa Tenggara Prov., Sumbawa (Batu Dulang, Tepal). Elevation: 1280–1490 m.

##### Etymology.

This epithet is based on the Latin adjective *pauxillus* (small) and refers to its body size.

##### Notes.

*Trigonopterus pauxillus* Riedel, sp. n. was coded as “*Trigonopterus* sp. 324” by Tänzler et al. (2014).

#### 58. Trigonopterus payungensis

Taxon classificationAnimaliaColeopteraCurculionidae

Riedel sp. n.

http://zoobank.org/46295DD6-BC87-4A60-98D7-F6C2FF4B847E

##### Diagnostic description.

**Figure 58.**
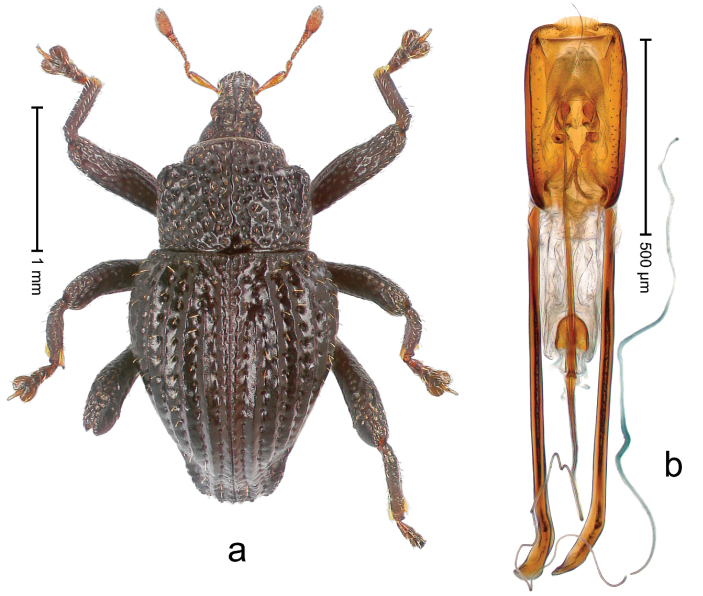
*Trigonopterus payungensis* Riedel, sp. n., holotype; **a** Habitus **b** Penis.

Holotype, male (Fig. 58). Length 2.58 mm. Color of tibiae, tarsi and antennae ferruginous, remainder black. Body subhexagonal; in dorsal aspect with marked constriction between pronotum and elytron; with distinct constriction in profile. Rostrum coarsely rugose-punctate, with median ridge and pair of irregular submedian ridges; epistome with indistinct, transverse, subangulate ridge. Pronotum anterolaterally angularly projecting; with distinct subapical constriction; disk scabrous, punctures each with one erect seta; interspaces weakly microreticulate; with median ridge. Elytra with striae deeply impressed, each with row of suberect slender scales partly abraded; intervals carinate, nude, weakly microreticulate; interval 7 swollen subapically, laterally weakly projecting. Femora edentate. Metafemur subapically with stridulatory patch and transverse row of denticles. Abdominal ventrite 5 flat, densely punctate, setose. Penis (Fig. 58b) in profile moderately curved; in dorsal aspect body slender, sides subparallel; apex with short, rounded extension; transfer apparatus flagelliform, 2 × longer than body; apodemes 2.2 × as long as body; ductus ejaculatorius without bulbus. **Intraspecific variation.** Length 2.40–2.58 mm. Female rostrum dorsally with longitudinal furrows; epistome simple. Pronotum with anterolateral knobs blunt, somewhat rounded in male, or more acute, angularly projecting in females. Female abdominal ventrite 5 flat.

##### Material examined.

Holotype (MZB): ARC2710 (EMBL # LM655966), West Java Prov., Garut, Cikajang, Mt. Payung, sample 2, S07°25.268', E107°48.492', 1250 m, 26-IV-2012. Paratypes (MZB, SMNK, ZSM): 7 exx, ARC3598 (EMBL # LM656028), ARC3599 (EMBL # LM656029), ARC3600 (EMBL # LM656030), ARC3601 (EMBL # LM656031), same data as holotype; 3 exx, ARC2702 (EMBL # LM655958), ARC2703 (EMBL # LM655959), ARC3590 (EMBL # LM656021), W-Java Prov., Garut, Cikajang, Mt. Payung, sample 1, S07°25.345', E107°48.825', 1085 m, 26-IV-2012.

##### Distribution.

W-Java Prov. (Mt. Payung). Elevation: 1085–1250 m.

##### Etymology.

This epithet is based on the type locality.

##### Notes.

*Trigonopterus payungensis* Riedel, sp. n. was coded as “*Trigonopterus* sp. 312”.

#### 59. Trigonopterus porcatus

Taxon classificationAnimaliaColeopteraCurculionidae

Riedel sp. n.

http://zoobank.org/85B04D6A-D2C7-4A6B-BBC8-96A794A722A4

##### Diagnostic description.

**Figure 59.**
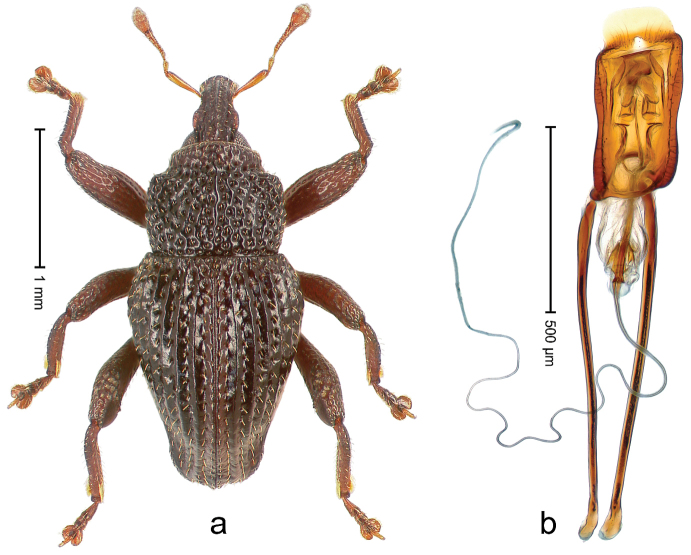
*Trigonopterus porcatus* Riedel, sp. n., holotype; **a** Habitus **b** Penis.

Holotype, male (Fig. 59a). Length 2.53 mm. Color of legs and head ferruginous, remainder black. Body elongate; in dorsal aspect with marked constriction between pronotum and elytron; with distinct constriction in profile. Rostrum coarsely rugose-punctate, with median ridge and pair of somewhat irregular submedian ridges; epistome with indistinct, transverse, subangulate ridge. Pronotum anterolaterally angularly projecting; with distinct subapical constriction; disk coarsely rugose-punctate, punctures each with one suberect seta; interspaces microreticulate; with median ridge. Elytra with striae deeply impressed, each with row of suberect bristles; intervals carinate, nude; interval 7 swollen subapically, laterally weakly projecting; sutural interval at apex slightly protruding. Femora edentate. Metafemur subapically with stridulatory patch and transverse row of denticles. Abdominal ventrite 5 flat, sparsely punctate, apically sparsely setose. Penis (Fig. 59b) asymmetrical, appearing twisted; in profile moderately curved; apex with broad, spatulate extension; transfer apparatus flagelliform, ca. as long as body; apodemes 2.0 × as long as body; ductus ejaculatorius without bulbus. **Intraspecific variation.** Length 2.15–2.63 mm. Female rostrum dorsally with pairs of lateral and submedian furrows; epistome simple.

##### Material examined.

Holotype (MZB): ARC2699 (EMBL # LM655955), West Java, Garut, Cikajang, Mt. Payung, sample 1, S07°25.345', E107°48.825', 1085 m, 26-IV-2012. Paratypes (MZB, SMNK, ZSM): W-Java Prov., Garut, Cikajang, Mt. Payung: 10 exx, ARC2700 (EMBL # LM655956), ARC2701 (EMBL # LM655957), ARC3638 (EMBL # LM656060), same data as holotype; 7 exx, ARC2711 (EMBL # LM655967), sample 2, S07°25.268', E107°48.492', 1250 m, 26-IV-2012; 8 exx, ARC2714 (EMBL # LM655970), sample 3, S07°25.320', E107°48.259', 1560 m, 26-IV-2012; 1 ex, ARC3594 (EMBL # LM656025), Sumedang, Sukajadi, Mt. Cakrabuana, sample 5, S07°01.804', E108°08.044', 1563 m, 20-IV-2012.

##### Distribution.

W-Java Prov. (Mt. Payung, Mt. Cakrabuana). Elevation: 1085–1563 m.

##### Etymology.

This epithet is based on the Latin *porca* (ridge between two furrows) and to be treated as an adjective. It refers to the elytral sculpture.

##### Notes.

*Trigonopterus porcatus* Riedel, sp. n. was coded as “*Trigonopterus* sp. 370”.

#### 60. Trigonopterus pseudoflorensis

Taxon classificationAnimaliaColeopteraCurculionidae

Riedel sp. n.

http://zoobank.org/C8F1FF61-9818-478E-ACF1-8E40A5A37698

##### Diagnostic description.

**Figure 60.**
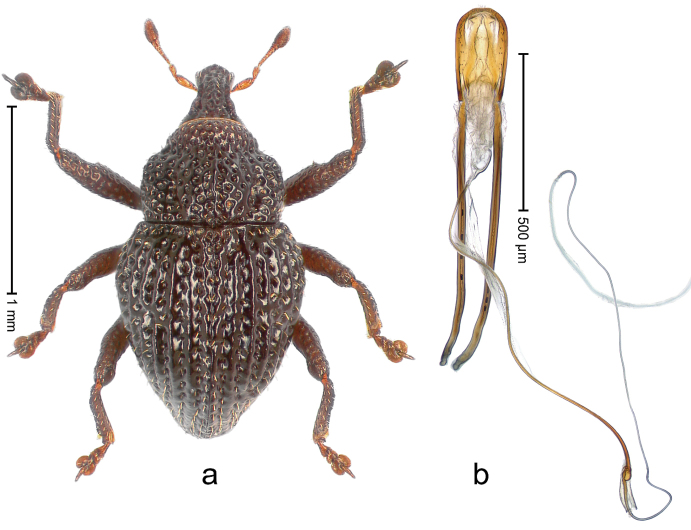
*Trigonopterus pseudoflorensis* Riedel, sp. n., holotype; **a** Habitus **b** Penis.

Holotype, male (Fig. 60a). Length 1.72 mm. Color of head and legs ferruginous, remainder black. Body in dorsal aspect with marked constriction between pronotum and elytron; with indistinct constriction in profile. Rostrum dorsally coarsely punctate-scabrous; median ridge and pair of submedian ridges indistinct; epistome with transverse, subangulate ridge indistinct. Pronotum with subapical constriction; disk coarsely punctate, sparsely setose; with pair of curved sublateral impressions; medially swollen. Elytra relatively slender; with striae deeply impressed, each with row of suberect bristles; intervals costate, subglabrous; apex narrow, rounded. Femora each with small tooth. Metafemur subapically with stridulatory patch. Abdominal ventrite 5 coarsely punctate, with median impression bordered by pair of longitudinal costae. Penis (Fig. 60b) with sides subparallel, in apical third markedly converging to narrow apex, medially with sparse setae; transfer apparatus flagelliform, 4.1 × as long as body; apodemes 3.7 × as long as body; ductus ejaculatorius without bulbus. **Intraspecific variation.** Length 1.70–2.14 mm. Penis with flagelliform transfer apparatus 4.1–4.6 × as long as body; apodemes 3.7–4.0 × as long as body. No females available.

##### Material examined.

Holotype (MZB): ARC2209 (EMBL # LM655665), East Nusa Tenggara Prov., Flores, Ruteng, Mt. Ranaka, sample 6, S08°38.243', E120°31.569', 1995 m, 09-III-2011. Paratypes (SMNK, ZSM): Flores: 3 exx, ARC3613 (EMBL # LM656043), ARC3614 (EMBL # LM656044), ARC3615 (EMBL # LM656045), Ruteng, Mt. Ranaka, sample 5, S08°38.277', E120°31.616', 2010 m, 09-III-2011.

##### Distribution.

East Nusa Tenggara Prov., Flores (Mt. Ranaka). Elevation: 1995–2010 m.

##### Etymology.

This epithet is based on the Greek prefix *pseudo* (false) and the name of *Trigonopterus florensis* Riedel, sp. n., a sibling species.

##### Notes.

*Trigonopterus pseudoflorensis* Riedel, sp. n. was coded as “*Trigonopterus* sp. 352” by Tänzler et al. (2014). Morphologically it is very similar to *Trigonopterus florensis* Riedel, sp. n. and *Trigonopterus paraflorensis* Riedel, sp. n. but its cox1 sequences differ 6.69%, respectively 7.14% smallest interspecific *p*-distance.

#### 61. Trigonopterus pseudosumbawensis

Taxon classificationAnimaliaColeopteraCurculionidae

Riedel sp. n.

http://zoobank.org/C176D270-253E-4B06-9AA5-A3B969A5E332

##### Diagnostic description.

**Figure 61.**
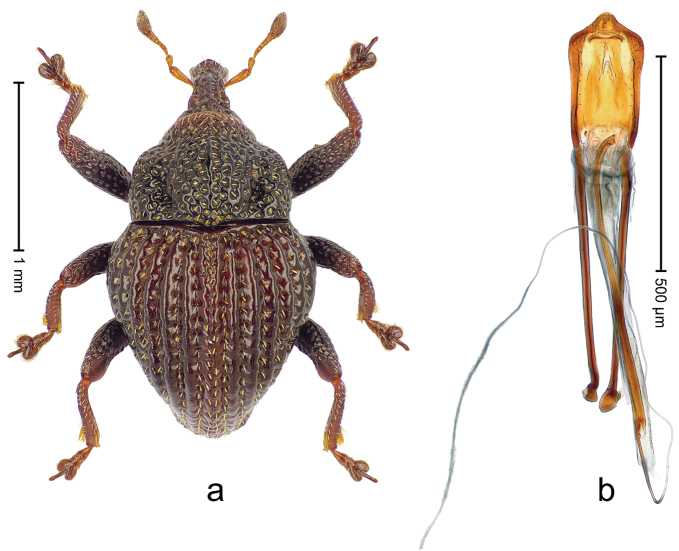
*Trigonopterus pseudosumbawensis* Riedel, sp. n., holotype; **a** Habitus **b** Penis.

Holotype, male (Fig. 61a). Length 1.94 mm. Color of antennae, legs and elytron ferruginous, remainder black. Body in dorsal aspect with marked constriction between pronotum and elytron; with distinct constriction in profile. Rostrum in basal half with median ridge and pair of submedian ridges, in apical half rugose-punctate; epistome with transverse, subangulate ridge. Pronotum with indistinct subapical constriction; disk coarsely punctate-rugose, sparsely setose; in basal half with pair of sublateral, kidney-shaped impressions. Elytra with striae deeply impressed, each with row of short, suberect scales; intervals costate, subglabrous; apex narrow. Femora with small tooth; metafemur subapically with stridulatory patch. Abdominal ventrite 5 flat, coarsely punctate. Penis (Fig. 61b) with sides of body subparallel; in apical quarter with sides angularly projecting, abruptly converging to slightly extended apex, medially with sparse setae; transfer apparatus flagelliform, 2.3 × longer than body; apodemes 2.3 × as long as body. **Intraspecific variation.** Length 1.52–1.94 mm. Color of elytra ferruginous or almost black. Body relatively slender or rather roundish. Penis with flagelliform transfer apparatus 2.3–2.4 × longer than body; apodemes 2.3–2.4 × as long as body.

##### Material examined.

Holotype (MZB): ARC3602 (EMBL # LM656032), West Nusa Tenggara Prov., Sumbawa, Batu Dulang, Gn. Batu Pasak, sample 3, S08°37.524', E117°15.423', 1385 m, 12-IV-2010. Paratypes (SMNK): Sumbawa, Batu Dulang, Gn. Batu Pasak: 2 exx, ARC3604 (EMBL # LM656034), ARC3643 (EMBL # LM656064), ARC3646 (EMBL # LM656067), ARC3651 (EMBL # LM656069), same data as holotype; 1 ex, ARC3657 (EMBL # LM656075), sample 5, S08°37.005', E117°15.790', 1320 m, 18-IV-2010.

##### Distribution.

West Nusa Tenggara Prov., Sumbawa (Batu Dulang). Elevation: 1385 m.

##### Etymology.

This epithet is based on the Greek prefix *pseudo* (false) and the name of *Trigonopterus sumbawensis* Riedel, sp. n., a sibling species.

##### Notes.

*Trigonopterus pseudosumbawensis* Riedel, sp. n. was coded as “*Trigonopterus* sp. 442”.

#### 62. Trigonopterus punctatoseriatus

Taxon classificationAnimaliaColeopteraCurculionidae

Riedel sp. n.

http://zoobank.org/C1FC2C71-5BCA-476B-B897-8DE53C786BBF

##### Diagnostic description.

**Figure 62.**
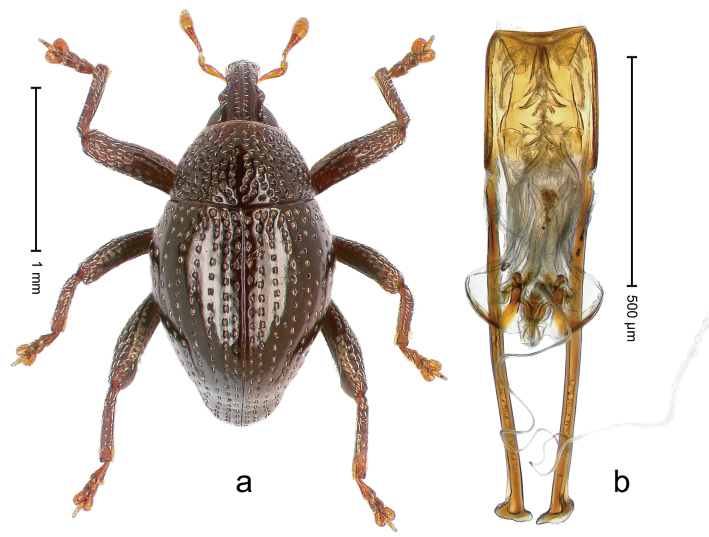
*Trigonopterus punctatoseriatus* Riedel, sp. n., holotype; **a** Habitus **b** Penis.

Holotype, male (Fig. 62a). Length 1.88 mm. Color of antennae and legs ferruginous; remainder black. Body subovate, in dorsal aspect with weak constriction between pronotum and elytron; in profile dorsally convex. Rostrum with median ridge and pair of submedian ridges, intervening furrows each with sparse row of mesad directed setae; epistome simple. Pronotum with coarse punctures partly forming longitudinal rows; each puncture containing long recumbent seta; interspaces subglabrous, especially midline. Elytra with striae distinct; each puncture containing one recumbent seta; intervals flat, subglabrous. Femora with simple, crenate anteroventral ridge. Metafemur subapically with stridulatory patch. Metatibia apically with uncus, without premucro. Abdominal ventrite 5 coarsely punctate, sparsely setose, flat. Penis (Fig. 62b) with sides of body subparallel; apex sparsely setose, converging to median triangular extension, curved ventrad; transfer apparatus compact, symmetrical, with two pair of tendons attached; laterally with pair of sickle-shaped sclerites; apodemes 2.8 × as long as body; ductus ejaculatorius without bulbus. **Intraspecific variation.** Length 1.84–2.00 mm. Female rostrum in apical half dorsally subglabrous, punctate.

##### Material examined.

Holotype (MZB): ARC1497 (EMBL # LM655595), West Nusa Tenggara Prov., Sumbawa, Tepal, Pc. Nengas, sample 2, S08°35.884', E117°08.384', 1310 m, 15-IV-2010. Paratypes (MZB, SMNK, ZSM): West Nusa Tenggara Prov., Sumbawa: 1 ex, ARC1498 (EMBL # LM655596), same data as holotype; 1 ex, ARC1535 (EMBL # LM655633), Tepal, Pc. Nengas, sample 5, S08°35.740', E117°08.721',1330 m, 16-IV-2010; 2 exx, Tepal, Pc. Nengas, sample 6, S08°35.533', E117°08.605',1350 m, 16-IV-2010; 1 ex, ARC1518 (EMBL # LM655616), Batu Dulang, Mt. Batu Pasak, sample 2, S08°37.028', E117°15.783', 1305 m, 12-IV-2010; 1 ex, ARC1528 (EMBL # LM655626), Batu Dulang, Mt. Batu Pasak, sample 3, S08°37.524', E117°15.423', 1385 m, 18-IV-2010.

##### Distribution.

West Nusa Tenggara Prov., Sumbawa (Batu Dulang, Tepal). Elevation: 1305–1385 m.

##### Etymology.

This epithet is based on a combination of the Latin participles *punctatus* (punctate) and *seriatus* (in rows) and refers to the elytral sculpture.

##### Notes.

*Trigonopterus punctatoseriatus* Riedel, sp. n. was coded as “*Trigonopterus* sp. 287” by Tänzler et al. (2014).

#### 63. Trigonopterus ranakensis

Taxon classificationAnimaliaColeopteraCurculionidae

Riedel sp. n.

http://zoobank.org/D9DF6F45-A030-4705-95C6-C52EF6564741

##### Diagnostic description.

**Figure 63.**
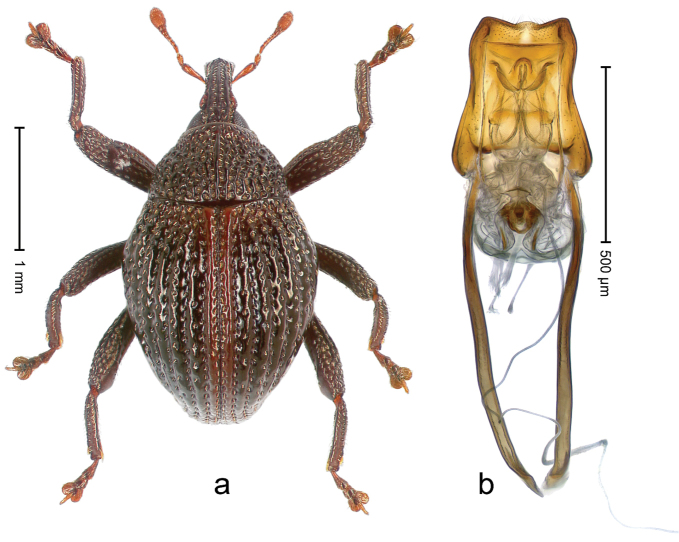
*Trigonopterus ranakensis* Riedel, sp. n., holotype; **a** Habitus **b** Penis.

Holotype, male (Fig. 63a). Length 2.53 mm. Color of antennae, legs and sutural interval ferruginous; remainder black. Body subovate, in dorsal aspect and in profile with weak constriction between pronotum and elytron. Rostrum with median ridge and pair of submedian ridges, intervening furrows each with sparse row of mesad directed setae; epistome with weak transverse ridge. Pronotum coarsely punctate, laterally reticulate, submedially interspaces longitudinally rugose, with median costa; with sparse, suberect, slender scales. Elytra with striae deeply impressed, each punctures containing slender suberect scale; intervals costate, subglabrous, in basal half partly with row of punctures, along basal margin corrugate; suture incised, bordered by dense rows of punctures. Femora with simple, crenate anteroventral ridge. Metafemur subapically with stridulatory patch. Metatibia apically with uncus, without premucro. Abdominal ventrite 5 coarsely punctate, with median carina. Penis (Fig. 63b) with sides of body in basal half converging, in apical half subparallel; apex angulate, sparsely setose; transfer apparatus compact, symmetrical, with two pairs of tendons attached; apodemes 2.6 × as long as body; ductus ejaculatorius without bulbus. **Intraspecific variation.** Length 2.16–2.73 mm. Female rostrum in apical half dorsally subglabrous, punctate; epistome simple. Color of sutural interval ferruginous or darker, almost black. Female abdominal ventrite flat, with very low median ridge.

##### Material examined.

Holotype (MZB): ARC2196 (EMBL # LM655652), East Nusa Tenggara Prov., Flores Isl., Ruteng, Mt. Ranaka, sample 1, S08°37.321', E120°31.463', 1535 m, 08-III-2011 (MZB). Paratypes (MZB, SMNK): Flores Isl., Ruteng, Mt. Ranaka: 1 ex, ARC2197 (EMBL # LM655653), same data as holotype; 1 ex, ARC2198 (EMBL # LM655654), sample 3, S08°38.099', E120°31.745', summit area, 2205 m, 09-III-2011; 1 ex, ARC2582 (EMBL # LM655891), sample 7, S08°37.714', E120°31.509', 1850m, 09-III-2011.

##### Distribution.

East Nusa Tenggara Prov., Flores (Ruteng). Elevation: 1535–2205 m.

##### Etymology.

This epithet is based on the type locality.

##### Notes.

*Trigonopterus ranakensis* Riedel, sp. n. was coded as “*Trigonopterus* sp. 342” by Tänzler et al. (2014).

#### 64. Trigonopterus relictus

Taxon classificationAnimaliaColeopteraCurculionidae

Riedel sp. n.

http://zoobank.org/E4F269E2-ABDF-4E15-8518-412280ACCA4F

##### Diagnostic description.

**Figure 64.**
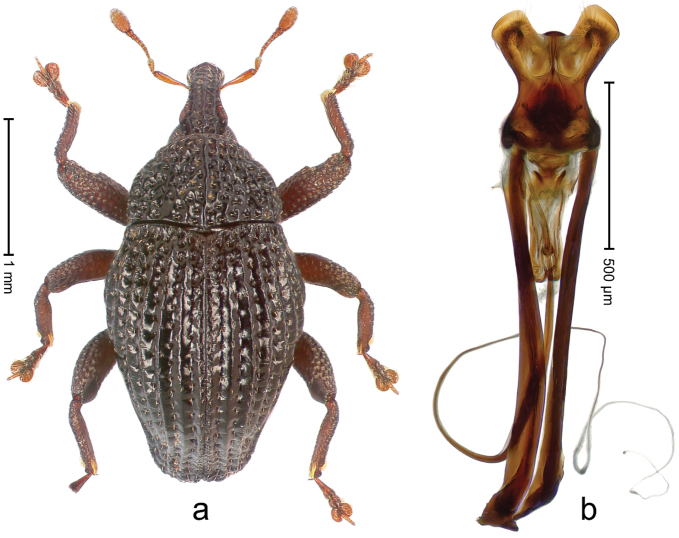
*Trigonopterus relictus* Riedel, sp. n., holotype; **a** Habitus **b** Penis.

Holotype, male (Fig. 64a). Length 2.60 mm. Color of head and legs ferruginous, remainder black. Body in dorsal aspect with marked constriction between pronotum and elytron; with shallow constriction in profile. Rostrum with median ridge and pair of submedian ridges, intervening furrows punctate, sparsely setose; epistome with indistinct, transverse, subangulate ridge. Pronotum with distinct subapical constriction; disk coarsely punctate-rugose, sparsely setose; with pair of indistinct sublateral impressions; with distinct median ridge. Elytra with striae deeply impressed; intervals costate to carinate, ridges with transverse wrinkles; sparsely setose with small suberect setae; apex with sublateral pair of blunt teeth, with suture incised. Femora each with small tooth. Metafemur subapically with stridulatory patch. Abdominal ventrite 5 concave, at middle subglabrous, laterally punctate, sparsely setose. Penis (Fig. 64b) with sides of body constricted at middle; apex with marked median notch; subapically markedly widened, forming blade-like processes bearing setose fringes on their dorsal surface; left blade-like process slightly larger; dorsally with median sickle-shaped process protruding apicad; transfer apparatus flagelliform, slightly longer than body; apodemes 3.2 × as long as body; ductus ejaculatorius without bulbus. **Intraspecific variation.** Length 2.08–2.60 mm. Color of body dark ferruginous to black. Body of females more compact, elytra shorter. Female rostrum dorsally in apical half with subglabrous costa, coarsely punctate; epistome simple. Female elytral apex subangulate, without pair of sublateral teeth. Female abdominal ventrite 5 flat, punctate.

##### Material examined.

Holotype (MZB): ARC0308 (EMBL # LM655498), E-Java Prov., Banyuwangi, Mt. Ijen, Licin, sample 4, S08°06.673', E114°14.488', 1255 m, 31-VIII-2006. Paratypes (MZB, SMNK, ZSM): E-Java Prov.: 19 exx, ARC0309 (EMBL # LM655499), same data as holotype; 33 exx, ARC0301 (EMBL # LM655491), ARC0302 (EMBL # LM655492), Banyuwangi, Mt. Ijen, Licin, sample 2, S08°05.603', E114°14.366', 1575 m, 28-VIII-2006; 12 exx, ARC0310 (EMBL # LM655500), ARC0311 (EMBL # LM655501), Banyuwangi, Mt. Ijen, Licin, sample 3, S08°06.056', E114°14.621', 1400 m, 28-VIII-2006; 2 exx, Banyuwangi, Mt. Ijen, Licin, sample 4, S08°06.673', E114°14.488', 1255 m, 28-VIII-2006; 2 exx, Banyuwangi, Mt. Ijen, Licin, sample 6, S08°07.115', E114°14.650', 1100 m, 31-VIII-2006; 9 exx, ARC2460 (EMBL # LM655798), ARC2461 (EMBL # LM655799), Krucil, Bremi, Mt. Argopuro (trail to Taman Hidup), sample 2, S07°58.478', E113°31.069', 1457 m, 10-XI-2011; 4 exx, ARC2462 (EMBL # LM655800), ARC2463 (EMBL # LM655801), Krucil, Bremi, Mt. Argopuro (trail to Taman Hidup), sample 3, S07°58.586', E113°31.345', 1628 m, 10-XI-2011; 2 exx, Krucil, Bremi, Mt. Argopuro (trail to Taman Hidup), sample 7, S07°58.449', E113°31.002', 1457 m, 11-XI-2011.

##### Distribution.

E-Java Prov. (Mt. Argopuro, Mt. Ijen). Elevation: 1100–1628 m.

##### Etymology.

This epithet is based on the Latin participle *relictus* (left behind), referring to the distribution of the species which inhabits an isolated patch of wet forest.

##### Notes.

*Trigonopterus relictus* Riedel, sp. n. was coded as “*Trigonopterus* sp. 317” by Tänzler et al. (2014).

#### 65. Trigonopterus rinjaniensis

Taxon classificationAnimaliaColeopteraCurculionidae

Riedel sp. n.

http://zoobank.org/CF981303-9B4C-4B44-AC0E-8DE024A6F950

##### Diagnostic description.

**Figure 65.**
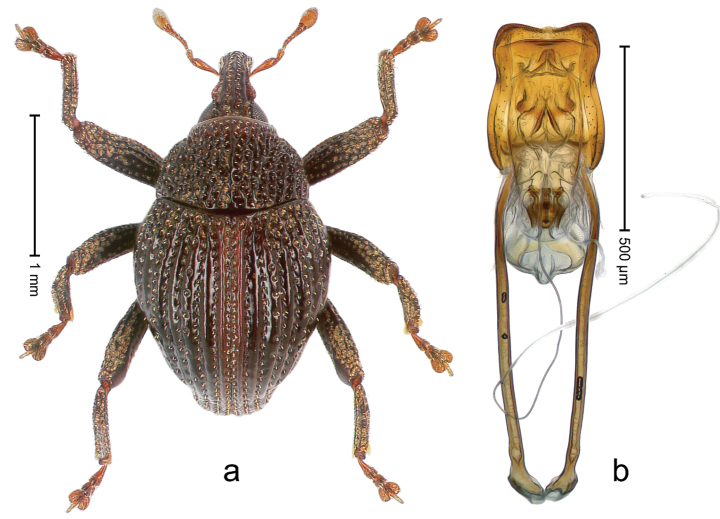
*Trigonopterus rinjaniensis* Riedel, sp. n., holotype; **a** Habitus **b** Penis.

Holotype, male (Fig. 65a). Length 2.18 mm. Color of antennae, tarsi and sutural interval ferruginous; remainder black. Body subovate, in dorsal aspect and in profile with weak constriction between pronotum and elytron. Rostrum with median ridge and pair of indistinct submedian ridges, intervening furrows each with sparse row of mesad directed setae; anterior third scabrous; epistome with transverse ridge. Pronotum coarsely punctate, laterally reticulate, submedially interspaces longitudinally rugose, with median costa; with sparse, suberect, slender scales. Elytra with striae deeply impressed; each with row of small suberect scales; intervals costate, subglabrous; intervals 1, 3, 5 more distinctly swollen, each with row of punctures; suture incised, bordered by dense rows of punctures. Femora with simple, crenate anteroventral ridge. Metafemur subapically with stridulatory patch. Metatibia apically with uncus, without premucro. Abdominal ventrite 5 coarsely punctate, with sparse suberect scales; with median carina. Penis (Fig. 65b) at middle with distinct constriction; containing several sclerites; apex subangulate, with sparse short setae, median extension minute; transfer apparatus compact, symmetrical, with two pairs of tendons attached; transfer process spiniform, pointing ventrad; apodemes 2.3 × as long as body; ductus ejaculatorius without bulbus. **Intraspecific variation.** Length 2.18–2.41 mm. Color of sutural interval ferruginous or darker, almost black. Female rostrum in apical half dorsally subglabrous, punctate; epistome simple. Female abdominal ventrite 5 flat.

##### Material examined.

Holotype (MZB): ARC2263 (EMBL # LM655717), West Nusa Tenggara Prov., Lombok, Gn. Rinjani, Senaru, Rinjani-track, sample 3, S08°20.780', E116°23.790', 1465 m, 21-III-2011. Paratypes (MZB, SMNK, ZSM): West Nusa Tenggara Prov., Lombok, Gn. Rinjani: 2 exx, ARC2264 (EMBL # LM655718), same data as holotype; 2 exx, ARC1464 (EMBL # LM655562), ARC1465 (EMBL # LM655563), Tetebatu, Rinjani-trail from Orong Gerisak, sample 8, S08°29.173', E116°24.517', 1345 m, 04-IV-2010; 1 ex, ARC1469 (EMBL # LM655567), Tetebatu, Rinjani-trail from Orong Gerisak, sample 7, S08°29.433', E116°24.746', 1240 m, 04-IV-2010; 1 ex, ARC1470 (EMBL # LM655568), Tetebatu, Rinjani-trail from Kokok Belimbing, sample 3, S08°29.433', E116°24.746', 1245 m, 02-IV-2010; 1 ex, ARC1473 (EMBL # LM655571), Tetebatu, Rinjani-trail from Kokok Belimbing, sample 4, S08°28.320', E116°26.459', 1420 m, 02-IV-2010; 5 exx, ARC0167, Tetebatu, Rinjani-trail, 1200-1450 m, 07-XII-2004; ARC1487 (EMBL # LM655585), road between Sembalun and Sapit, sample 1, S08°25.110', E116°31.947', 1450 m, 30-III-2010; 1 ex, ARC1489 (EMBL # LM655587), road between Sembalun and Sapit, sample 4, S08°25.331', E116°31.841', 1405 m, 31-III-2010; 1 ex, ARC1492 (EMBL # LM655590), road between Sembalun and Sapit, sample 5, S08°26.453', E116°31.859', 1195 m, 31-III-2010.

##### Distribution.

West Nusa Tenggara Prov., Lombok (Senaru, Sembalun, Tetebatu). Elevation: 1195–1465 m.

##### Etymology.

This epithet is based on the type locality, Mt. Rinjani.

##### Notes.

*Trigonopterus rinjaniensis* Riedel, sp. n. was coded as “*Trigonopterus* sp. 282” by Tänzler et al. (2014).

#### 66. Trigonopterus roensis

Taxon classificationAnimaliaColeopteraCurculionidae

Riedel sp. n.

http://zoobank.org/823C11A3-19A5-4044-B0A2-F1E541083655

##### Diagnostic description.

**Figure 66.**
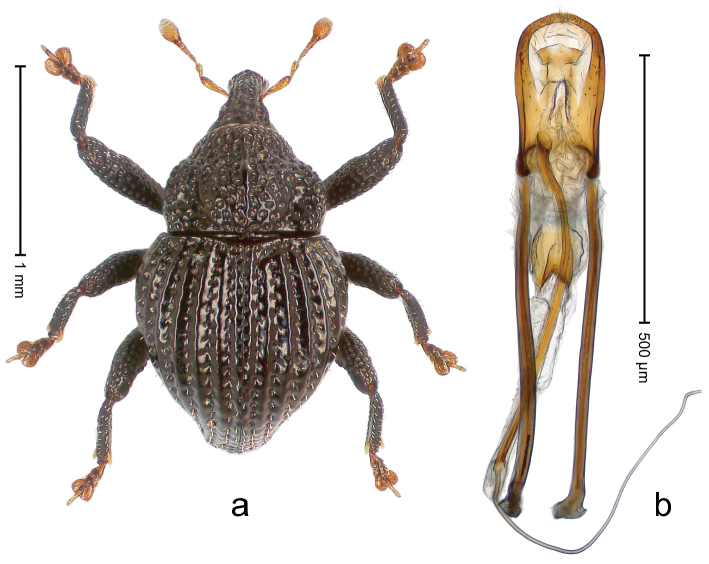
*Trigonopterus roensis* Riedel, sp. n., holotype; **a** Habitus **b** Penis.

Holotype, male (Fig. 66a). Length 1.75 mm. Color of antennae and tarsi ferruginous, remainder black. Body in dorsal aspect with marked constriction between pronotum and elytron; with distinct constriction in profile. Rostrum with median ridge and pair of submedian ridges, intervening furrows with sparse rows of setae; epistome with transverse, subangulate ridge. Pronotum with subapical constriction; disk coarsely punctate-rugose, sparsely setose; in basal half with pair of sublateral, kidney-shaped impressions; with median ridge. Elytra with striae deeply impressed, each with row of short, suberect setae; intervals costate-carinate, subglabrous; apex subtruncate, weakly rounded. Femora each with small tooth; profemur relatively long. Metafemur subapically with stridulatory patch. Abdominal ventrite 5 flat, coarsely punctate. Penis (Fig. 66b) with sides of body subparallel, in apical third converging; apex subangulate, medially with sparse setae; transfer apparatus flagelliform, relatively thick, 2.6 × as long as body; apodemes 2.5 × as long as body. **Intraspecific variation.** Length 1.71–1.86 mm. Coloration dark as in holotype, or largely deep ferruginous. Female rostrum dorsally subglabrous, sparsely punctate, with dorsolateral pair of furrows.

##### Material examined.

Holotype (MZB): ARC2219 (EMBL # LM655675), East Nusa Tenggara Prov., Flores, Labuhan Bajo, Roe, sample 1, S08°36.540', E120°01.871', 955 m, 13-III-2011. Paratypes (MZB, SMNK): Flores: 1 ex, ARC2218 (EMBL # LM655674), same data as holotype; 2 exx, ARC2227 (EMBL # LM655683), ARC2228 (EMBL # LM655684), Labuhan Bajo, Roe, sample 5, S08°35.395', E120°00.383', 790 m, 13-III-2011; 2 exx, ARC2245 (EMBL # LM655700), ARC2246 (EMBL # LM655701), Labuhan Bajo, Tebedo, lowland forest, sample 1, S08°29.848', E119°59.633', 525 m, 14-III-2011.

##### Distribution.

East Nusa Tenggara Prov., Flores (Labuhan Bajo). Elevation: 525–955 m.

##### Etymology.

This epithet is based on the type locality.

##### Notes.

*Trigonopterus roensis* Riedel, sp. n. was coded as “*Trigonopterus* sp. 353” by Tänzler et al. (2014).

#### 67. Trigonopterus rugosostriatus

Taxon classificationAnimaliaColeopteraCurculionidae

Riedel sp. n.

http://zoobank.org/CAC7CFEB-2337-4B64-87E0-1E3419BBB6F5

##### Diagnostic description.

**Figure 67.**
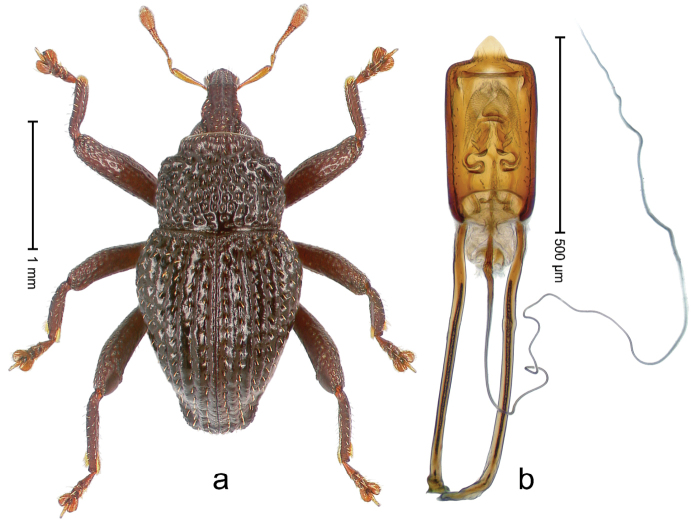
*Trigonopterus rugosostriatus* Riedel, sp. n., holotype; **a** Habitus **b** Penis.

Holotype, male (Fig. 67a). Length 2.40 mm. Color of legs and head ferruginous, remainder black. Body in dorsal aspect with marked constriction between pronotum and elytron; with distinct constriction in profile. Rostrum coarsely rugose-punctate, with median ridge and pair of indistinct submedian ridges; epistome with indistinct, transverse, subangulate ridge. Pronotum anterolaterally angularly projecting; with distinct subapical constriction; disk rugose-punctate, interspaces weakly microreticulate; punctures each with one suberect to recumbent scale becoming shorter towards base; with median ridge. Elytra with striae deeply impressed, each with row of slender suberect scales; intervals costate to carinate, almost nude, weakly microreticulate; interval 7 swollen subapically, laterally weakly projecting; sutural interval subapically simple. Femora edentate. Metafemur subapically with stridulatory patch and transverse ridge. Abdominal ventrite 5 in apical half slightly swollen, with median impression, punctate, sparsely setose. Penis (Fig. 67b) with body in profile moderately curved; in dorsal aspect sides subparallel; apex with median, acute triangular extension; transfer apparatus flagelliform, slightly shorter than body; apodemes 2.0 × as long as body; ductus ejaculatorius without bulbus. **Intraspecific variation.** Length 2.28–2.65 mm. Color of body black to dark ferruginous. Female rostrum dorsally with longitudinal furrows; epistome simple. Pronotum with anterolateral knobs rounded, or more distinct and angularly projecting, especially in females. Female abdominal ventrite 5 flat.

##### Material examined.

Holotype (MZB): ARC2672 (EMBL # LM655930), West Java Prov., Sumedang, Sukajadi, Mt. Cakrabuana, sample 4, S07°01.734', E108°08.059', 1530 m, 19-IV-2012. Paratypes (MZB, SMNK, ZSM): W-Java Prov.: 3 exx, ARC2673 (EMBL # LM655931), ARC2674 (EMBL # LM655932), ARC3591 (EMBL # LM656022), same data as holotype; 4 exx, ARC2675 (EMBL # LM655933), ARC2676 (EMBL # LM655934), ARC2677 (EMBL # LM655935), ARC3592 (EMBL # LM656023), Sumedang, Sukajadi, Mt. Cakrabuana, sample 5, S07°01.804', E108°08.044', 1563 m, 20-IV-2012; 1 ex, ARC2681 (EMBL # LM655939), Sumedang, Sukajadi, Mt. Cakrabuana, sample 6, S07°01.943', E108°08.029', 1593 m, 20-IV-2012; 1 ex, ARC2671 (EMBL # LM655929), Sumedang, Sukajadi, Mt. Cakrabuana, sample 3, S07°01.493', E108°08.065', 1394 m, 19-IV-2012; 5 exx, ARC0157, ARC0408, Ciamis, Mt. Sawal, Blok Cireong, sample 2, S07°14.127', E108°15.568', 1120 m, 01-X-2005; 3 exx, Batu Cakra, sample 1, S07°14.920', E108°15.762', 990 m, 01-X-2005; 3 exx, ARC2505 (EMBL # LM655842), ARC2506 (EMBL # LM655843), ARC2507 (EMBL # LM655844), Panjalu, Tembong, sample 3, S07°11.102', E108°16.310', 1569 m, 29-XI-2011.

##### Distribution.

W-Java Prov. (Mt. Cakrabuana, Mt. Sawal). Elevation: 990–1569 m.

##### Etymology.

This epithet is a combination of the Latin adjective *rugosus* (wrinkled) and the participle *striatus* (provided with furrows) and refers to the species´ body-sculpture.

##### Notes.

*Trigonopterus rugosostriatus* Riedel, sp. n. was coded as “*Trigonopterus* sp. 354”.

#### 68. Trigonopterus rugosus

Taxon classificationAnimaliaColeopteraCurculionidae

Riedel sp. n.

http://zoobank.org/A7757AE1-955B-49A6-8238-29D7153E5195

##### Diagnostic description.

**Figure 68.**
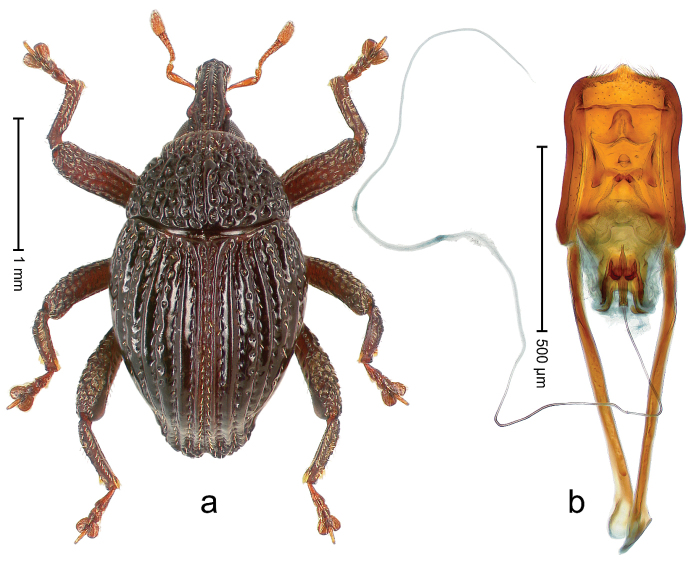
*Trigonopterus rugosus* Riedel, sp. n., holotype; **a** Habitus **b** Penis.

Holotype, male (Fig. 68a). Length 2.53 mm. Color of antennae and legs ferruginous; head and elytral sutural interval dark ferruginous, remainder black. Body subovate, in dorsal aspect with weak constriction between pronotum and elytron; with distinct constriction in profile. Rostrum with median ridge and pair of submedian ridges, intervening furrows each with sparse row of mesad directed scales; epistome with sinuate transverse ridge. Pronotum without subapical constriction; disk with coarse irregular ridges and tubercles, with sparse, suberect scales. Elytra with striae deeply impressed, each with sparse row of slender, suberect scales; intervals costate, intervals 3 and 5 with row of punctures, others subglabrous; sutural interval basally swollen and widened laterad; suture incised, bordered by rows of suberect scales; elytral apex scabrous, margin subtruncate, with slightly incised suture, with distinct apical denticle. Femora with simple anteroventral ridge, in meso- and metafemur crenate. Metafemur subapically with stridulatory patch. Abdominal ventrite 5 dull, with T-shape ridge along base and middle, coarsely punctate, with sparse erect scales. Penis (Fig. 68b) with sides of body weakly concave; apical edge subangulate, with sparse fringe of setae; apex with median angulate extension; transfer apparatus compact; apodemes 2.0 × as long as body; ductus ejaculatorius without bulbus. **Intraspecific variation.** Length 2.36–2.53 mm. Color of elytra completely black, with ferruginous sutural interval, or entirely ferruginous. Female rostrum in apical third dorsally subglabrous, sparsely punctate; epistome simple. Female elytra ventroapically with pair of laterally flattened denticles, male elytra ventroapically simple. Female abdominal ventrite 5 medially with weak ridge.

##### Material examined.

Holotype (MZB): ARC0586 (EMBL # LM655522), Bali, Bedugul, Mt. Catur, sample 3, S08°15.626', E115°11.354', 1690 m, 07-XI-2007. Paratypes (MZB, SMNK, ZSM): Bali: 3 exx, ARC0587 (EMBL # LM655523), same data as holotype; 3 exx, Bedugul, Mt. Catur, sample 2, S08°15.147', E115°11.323', 1930 m, 07-XI-2007; 3 exx, ARC0571 (EMBL # LM655507), ARC0572 (EMBL # LM655508), Bedugul, Mt. Pohen (= Mt. Tapak = Mt.Keramat), sample 2, S08°16.328', E115°08.360', 1785 m, 01-XI-2007; 2 exx, ARC0573 (EMBL # LM655509), Bedugul, Mt. Pohen (= Mt. Tapak = Mt.Keramat), sample 3, S08°16.386', E115°08.402', 1735 m, 01-XI-2007; 1 ex, ARC0574 (EMBL # LM655510), Bedugul, Mt. Pohen (= Mt. Tapak = Mt.Keramat), sample 5, S08°16.526', E115°08.634', 1580 m, 01-XI-2007; 1 ex Bedugul, Mt. Pohen (= Mt. Tapak = Mt.Keramat), sample 6, S08°16.607', E115°08.773', 1460 m, 01-XI-07; 4 exx, ARC0591 (EMBL # LM655527), ARC0592 (EMBL # LM655528), ARC0593 (EMBL # LM655529), ARC0594 (EMBL # LM655530), Kintamani, Mt. Abang, sample 1, S08°17.118', E115°24.868', 1440 m, 09-XI-2007, 01-XI-2007; 6 exx, ARC2317 (EMBL # LM655770), ARC2318 (EMBL # LM655771), Angseri, Mt. Adeng, sample 3, S08°19.626', E115°08.554', 1385 m, 06-IV-2011; 5 exx, Angseri, Mt. Adeng, sample 4, S08°19.463', E115°08.537', 1535 m, 06-IV-2011; 9 exx, Angseri, Mt. Adeng, sample 5, S08°19.402', E115°08.545', 1590 m, 06-IV-2011; 5 exx, ARC2321 (EMBL # LM655774), ARC2322 (EMBL # LM655775), Angseri, Mt. Adeng, sample 6, S08°19.138', E115°08.416', 1760 m, 06-IV-2011; 1 female, Bedugul, mountain NW of botanical garden, 1400 m, 04-XII-2004 (ARC).

##### Distribution.

Bali (Mt. Andeng, Bedugul, Kintamani). Elevation: 1385–1785 m.

##### Etymology.

This epithet is based on the Latin adjective *rugosus* and refers to the wrinkled integument.

##### Notes.

*Trigonopterus rugosus* Riedel, sp. n. was coded as “*Trigonopterus* sp. 280” by Tänzler et al. (2014). The minimal p-distances of cox1 to *Trigonopterus pararugosus* Riedel, sp. n. is 5.6–7.2%. Morphologically no clear differences could be detected and it is assumed that both represent a pair of cryptic species.

#### 69. Trigonopterus rutengensis

Taxon classificationAnimaliaColeopteraCurculionidae

Riedel sp. n.

http://zoobank.org/F94D327D-D925-4BBA-A6C8-3DB9C9D51A7D

##### Diagnostic description.

**Figure 69.**
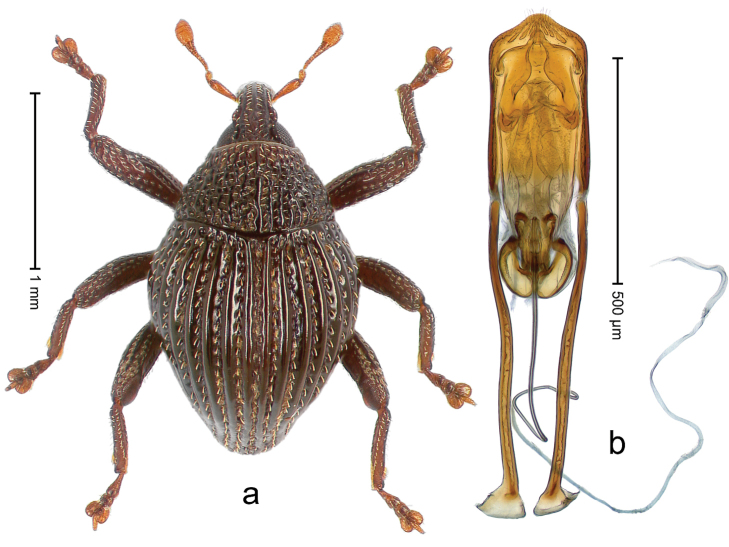
*Trigonopterus rutengensis* Riedel, sp. n., holotype; **a** Habitus **b** Penis.

Holotype, male (Fig. 69a). Length 1.78 mm. Color of antennae and tarsi ferruginous; remainder dark ferruginous to black, with slight bronze lustre. Body subovate, in dorsal aspect and in profile with weak constriction between pronotum and elytron. Rostrum with median ridge and pair of submedian ridges, intervening furrows each with sparse row of mesad directed scales; epistome simple. Pronotum coarsely punctate, interspaces longitudinally rugose; with median costa; with sparse, subrecumbent scales. Elytra with striae deeply impressed, each with row of slender suberect scales; intervals costate, subglabrous; incised suture bordered by row of squamiferous punctures, partly overgrown by costate sutural interval. Femora with simple, crenate anteroventral ridge. Metafemur subapically with stridulatory patch. Metatibia apically with uncus and small premucro. Abdominal ventrite 5 coarsely punctate, except apical third subglabrous. Penis (Fig. 69b) with sides of body subparallel; apex broadly angulate, medially rounded and sparsely setose; transfer apparatus compact, symmetrical, supported by large crescent-shaped sclerite; apodemes 2.0 × as long as body; ductus ejaculatorius without bulbus. **Intraspecific variation.** Length 1.78–2.02 mm. Color of elytra ferruginous to black, greenish-bronze lustre more or less distinct. Female rostrum in apical half dorsally subglabrous, longitudinally rugose-punctate; epistome simple.

##### Material examined.

Holotype (MZB): ARC2193 (EMBL # LM655649), East Nusa Tenggara Prov., Flores Isl., Danau Ranamese, sample 2, S08°37.235', E120°33.554', 1295 m, 08-III-2011. Paratypes (MZB, SMNK, ZSM): Flores Isl., Ruteng: 1 ex, ARC2207 (EMBL # LM655663), Mt. Ranaka, sample 2, S08°37.164', E120°31.106', 1450 m, 08-III-2011; 1 ex, ARC3627 (EMBL # LM656057), Mt. Ranaka, sample 11, S08°37.386', E120°31.156', 1575 m, 08-III-2011; 4 exx, ARC2584 (EMBL # LM655893), ARC2585 (EMBL # LM655894), Mt. Ranaka, sample 10, S08°37.481', E120°31.410', 1610 m, 10-III-2011; 3 exx, ARC2208 (EMBL # LM655664), ARC2577 (EMBL # LM655886), ARC2578 (EMBL # LM655887), Danau Ranamese, sample 4, S08°38.385', E120°33.568', 1215 m, 08-III-2011; 2 exx, ARC2586 (EMBL # LM655895), Danau Ranamese, sample 6, S08°38.370', E120°33.798', 1215 m, 11-III-2011.

##### Distribution.

East Nusa Tenggara Prov., Flores (Ruteng). Elevation: 1215–1610 m.

##### Etymology.

This epithet is based on the type locality.

##### Notes.

*Trigonopterus rutengensis* Riedel, sp. n. was coded as “*Trigonopterus* sp. 306”.

#### 70. Trigonopterus saltator

Taxon classificationAnimaliaColeopteraCurculionidae

Riedel sp. n.

http://zoobank.org/3E33D014-4206-40E2-8218-412EC4AA98E5

##### Diagnostic description.

**Figure 70.**
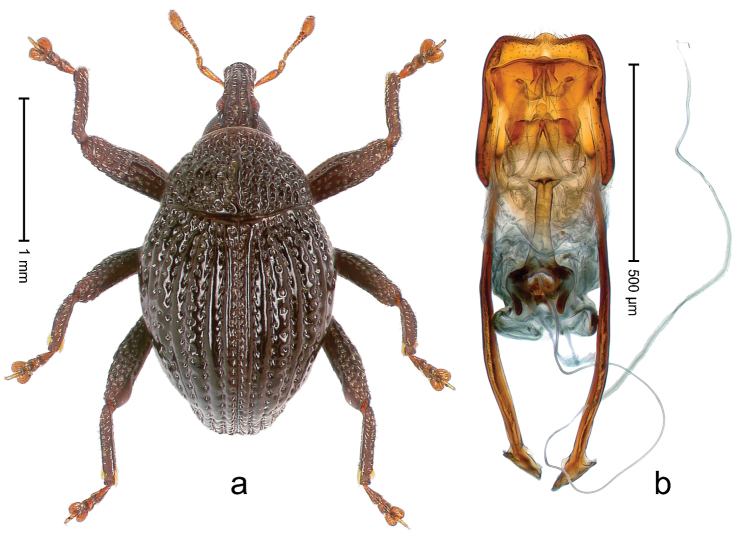
*Trigonopterus saltator* Riedel, sp. n., holotype; **a** Habitus **b** Penis.

Holotype, male (Fig. 70a). Length 2.20 mm. Color of antennae and legs ferruginous; remainder black. Body subovate, in dorsal aspect and in profile with weak constriction between pronotum and elytron. Rostrum with median ridge and pair of indistinct submedian ridges, intervening furrows each with sparse row of mesad directed setae; anterior third scabrous; epistome with transverse ridge. Pronotum coarsely punctate, laterally reticulate, submedially interspaces longitudinally rugose, with median costa; with sparse, suberect, slender scales. Elytra with striae deeply impressed; each with row of minute suberect setae; intervals costate, subglabrous; intervals 1, 3, 5 more distinctly swollen, each with row of punctures; row of punctures on sutural interval dense, facing mesad towards incised suture. Femora with simple, crenate anteroventral ridge. Metafemur subapically with stridulatory patch. Protibia with uncus relatively long, stout. Metatibia apically with uncus, without premucro. Abdominal ventrite 5 coarsely punctate, with sparse suberect setae; with median carina. Penis (Fig. 70b) with sides of body converging, at middle with shallow constriction; containing several sclerites; apex sparsely setose, with median, subtriangular extension; transfer apparatus small, symmetrical, with pair of tendons attached, with median narrow apically biramose sclerite; apodemes 2.7 × as long as body; ductus ejaculatorius without bulbus. **Intraspecific variation.** Length 2.20–2.31 mm. Color of sutural interval and elytral apex ferruginous or darker, almost black. Female unknown.

##### Material examined.

Holotype (MZB): ARC1530 (EMBL # LM655628), West Nusa Tenggara Prov., Sumbawa, Batu Dulang, Gn. Batu Pasak, sample 3, S08°37.524', E117°15.423', 1385 m, 18-IV-2010. Paratypes (SMNK): 2 exx, ARC1529 (EMBL # LM655627), ARC1531 (EMBL # LM655629), same data as holotype.

##### Distribution.

West Nusa Tenggara Prov., Sumbawa (Batu Dulang). Elevation: 1385 m.

##### Etymology.

This epithet is based on the Latin noun *saltator* (leaper) and refers to the biogeographic pattern where close relatives of this species are found on islands to the West.

##### Notes.

*Trigonopterus saltator* Riedel, sp. n. was coded as “*Trigonopterus* sp. 281” by Tänzler et al. (2014).

#### 71. Trigonopterus santubongensis

Taxon classificationAnimaliaColeopteraCurculionidae

Riedel sp. n.

http://zoobank.org/D50D6C5A-110B-4DD6-98BD-8447785B4D85

##### Diagnostic description.

**Figure 71.**
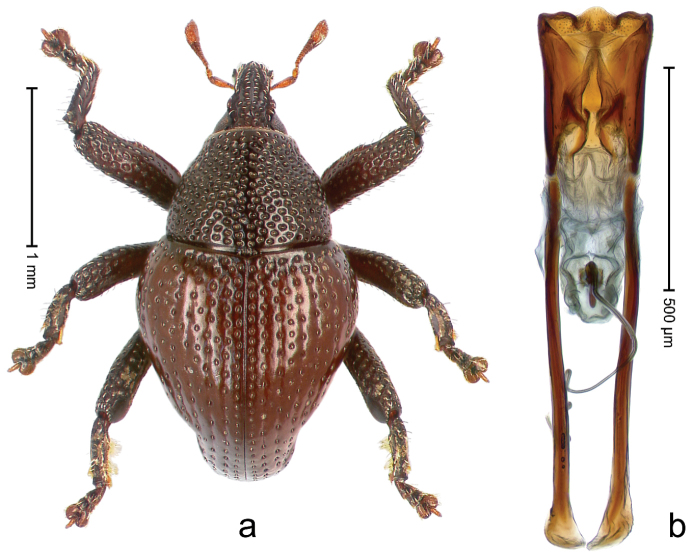
*Trigonopterus santubongensis* Riedel, sp. n., holotype; **a** Habitus **b** Penis.

Holotype, male (Fig. 71a). Length 2.23 mm. Color of antennae, legs and elytra ferruginous; remainder black. Body in dorsal aspect subrhomboid, with weak constriction between pronotum and elytron; in profile dorsally convex. Rostrum with indistinct median and pair of submedian ridges uniting in apical third; coarsely punctate-reticulate, with sparse suberect scales; epistome with irregular, subangulate ridge. Pronotum with disk densely punctate, reticulate; each puncture containing small recumbent seta. Elytra with striae distinct, marked by coarse punctures; intervals flat, microreticulate; sutural intervals with dense row of smaller punctures, remaining intervals with few interspersed coarse punctures; each puncture containing small seta; elytral apex subtruncate. Anteroventral ridge of femora terminating in apical third forming indistinct tooth. Profemur in basal half posteroventrally with tooth. Metafemur subapically with stridulatory patch. Dorsal edge of tibiae with subbasal angulation, dentate in protibia. Metatibia ventrally with fringe of long, stiff setae. Thoracic venter deeply concave. Abdominal ventrites 1–2 concave, subglabrous, near metacoxae with small cluster of peg-shaped setae; abdominal ventrite 5 concave, at base laterally with protrusion, at middle glabrous, apically densely setose. Penis (Fig. 71b) with sides of body diverging, containing two pairs of distinct sclerites; apex with median rounded extension bordered by pair of swellings; transfer apparatus spiniform; apodemes 2.5 × as long as body; ductus ejaculatorius apically torn off, in other males without distinct bulbus. **Intraspecific variation.** Length 2.23–2.53 mm. Female rostrum dorsally subglabrous, with submedian row of coarse punctures; epistome simple, subapically with 4 shallow longitudinal impressions. Female metatibia ventrally simple, with fringe of sparse thin setae. Female abdominal ventrite 1 without cluster of peg-shaped setae; female abdominal ventrite 5 subbasally with marked transverse swelling.

##### Material examined.

Holotype (SMNK): ARC2523 (EMBL # LM655860), MALAYSIA, Sarawak, Kuching, Mt. Santubong, sample 3, N01°43.884', E110°19.727', 496 m, 06-XII-2011. Paratypes (SMNK, ZSM): MALAYSIA, Sarawak, Kuching, Mt. Santubong: 1 ex, ARC2524 (EMBL # LM655861), same data as holotype; 13 exx, ARC2527 (EMBL # LM655864), ARC2528 (EMBL # LM655865), sample 1, N01°43.760', E110°19.113', 138 m, 05-XII-2011; 6 exx, sample 2, N01°43.779', E110°19.183', 173 m, 05-XII-2011; 1 ex, sample 3, N01°43.884', E110°19.727', 496 m, 06-XII-2011; 10 exx, sample 4, N01°43.884', E110°19.715', 487 m, 06-XII-2011; 8 exx, sample 5, N01°43.843', E110°19.672', 478 m, 06-XII-2011; 10 exx, sample 6, N01°43.830', E110°19.601', 375 m, 06-XII-2011.

##### Distribution.

Sarawak (Santubong). Elevation: 138–496 m.

##### Etymology.

This epithet is based on the type locality.

##### Notes.

*Trigonopterus santubongensis* Riedel, sp. n. was coded as “*Trigonopterus* sp. 361”.

#### 72. Trigonopterus sasak

Taxon classificationAnimaliaColeopteraCurculionidae

Riedel sp. n.

http://zoobank.org/0AA22178-609E-480D-9ACB-4631E165A7C0

##### Diagnostic description.

**Figure 72.**
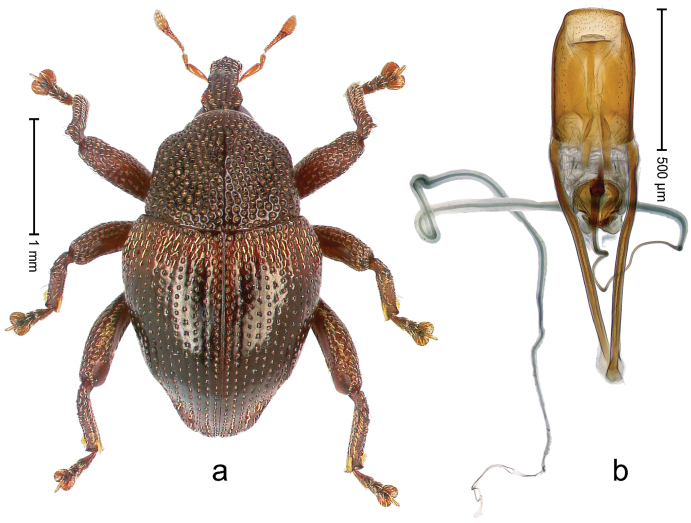
*Trigonopterus sasak* Riedel, sp. n., holotype; **a** Habitus **b** Penis.

Holotype, male (Fig. 72a). Length 2.88 mm. Color ferruginous, pronotum and center of elytron black. Body subovate; in dorsal aspect with marked constriction between pronotum and elytron; in profile dorsally convex. Rostrum in apical half scabrous, coarsely punctate; in basal half with median and pair of submedian ridges; intervening furrows punctate; with sparse subrecumbent, piliform scales; epistome with transverse, angulate ridge forming indistinct median denticle. Pronotum with sides converging in straight line, anterolaterally rounded to subapical constriction; disk with pair of submedian depressions, coarsely punctate, reticulate; each puncture containing inconspicuous seta; with distinct median ridge. Elytra with striae indistinct; intervals flat, punctate; at base, laterally and subapically ferruginous, densely coarsely punctate, with scattered recumbent lanceolate scales; remainder less densely punctate, punctures in rows, black. Anteroventral ridge of femora distinct, in meso- and metafemur forming large tooth. Metafemur subapically with stridulatory patch. Dorsal edge of tibiae subbasally with angulation, in pro- and mesotibia with small tooth. Abdominal ventrite 5 flat, coarsely punctate. Penis (Fig. 72b) with sides of body subparallel; in apical 1/3 tapering to rounded apex; transfer apparatus compact; apodemes 2.3 × as long as body; ductus ejaculatorius with indistinct bulbus. **Intraspecific variation.** Length 2.38–2.98 mm. Color of specimens from Sesaot as in holotype; from other localities darker, body black, legs dark ferruginous, only antennae light ferruginous. Female body more slender. Female rostrum dorsally punctate-rugose, with median and pair of submedian subglabrous costae; epistome simple. Elytra with scales near base more or less distinct.

##### Material examined.

Holotype (MZB): ARC1482 (EMBL # LM655580), West Nusa Tenggara Prov., Lombok, Sesaot, Rinjani-trail, sample 4, S08°30.047', E116°13.826', 615 m, 28-III-2010. Paratypes (MZB, SMNK, ZSM): Lombok: 2 exx, ARC1481 (EMBL # LM655579), same data as holotype; 1 ex, ARC1484 (EMBL # LM655582), Sesaot, Rinjani-trail, sample 3, S08°29.841', E116°13.679', 625 m, 28-III-2010; 1 ex, ARC2301 (EMBL # LM655755), W Sengigi, Mt. Pusuk, sample 3, S08°27.723', E116°04.160', 585 m, 29-III-2011; 1 ex, ARC2285 (EMBL # LM655739), Sajang, sample 1, S08°19.370', E116°29.570', 845 m, 26-III-2011; 10 exx, ARC2295 (EMBL # LM655749), ARC2296 (EMBL # LM655750), ARC2297 (EMBL # LM655751), ARC2298 (EMBL # LM655752), Mt. Pengasingan, SE Sembalun, sample 2, S08°19.354', E116°30.530', 945 m, 27-III-2011.

##### Distribution.

West Nusa Tenggara Prov., Lombok (Mt. Pusuk, Sajang, Sembalun, Sesaot). Elevation: 615–945 m.

##### Etymology.

This epithet is based on the Sasak tribe traditionally inhabiting the island of Lombok. It is a noun in apposition.

##### Notes.

*Trigonopterus sasak* Riedel, sp. n. was coded as “*Trigonopterus* sp. 330” by Tänzler et al. (2014). Cox1 sequences of the populations from West and East Lombok differ 9.7–10.6% *p*-distance, but morphologically no differences could be found.

#### 73. Trigonopterus satu

Taxon classificationAnimaliaColeopteraCurculionidae

Riedel sp. n.

http://zoobank.org/8CA7D9CD-B105-4E0F-B5EC-06C65838923E

##### Diagnostic description.

**Figure 73.**
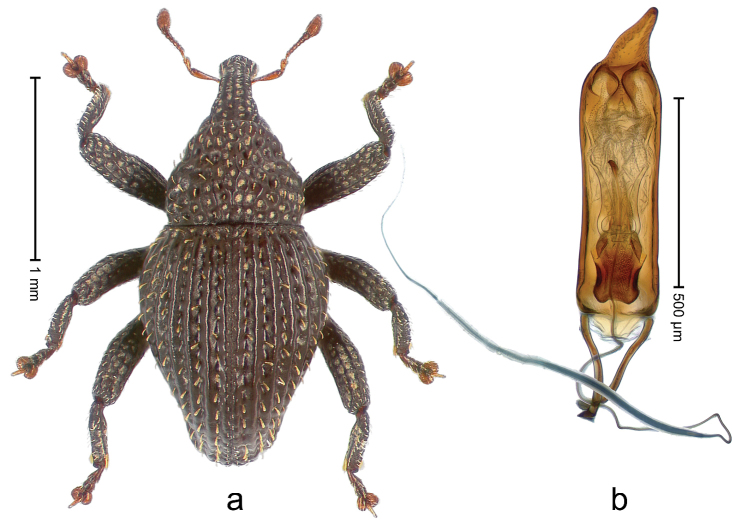
*Trigonopterus satu* Riedel, sp. n., holotype; **a** Habitus **b** Penis.

Holotype, male (Fig. 73a). Length 1.90 mm. Color of tarsi and antennae ferruginous; remainder black. Body subovate; in dorsal aspect and in profile with distinct constriction between pronotum and elytron. Rostrum with median ridge and pair of submedian ridges; with sparse rows of suberect setae; epistome with transverse, angulate ridge. Pronotum with shallow subapical constriction; disk coarsely punctate, scabrous; each puncture containing erect clavate scale inserting at posterior rim; with median ridge. Elytra with striae marked by deep punctures; in front of each puncture with elongate-claviform, erect scale; intervals costate, subglabrous, weakly microreticulate; interval 7 subapically costate; sutural interval simple. Femora edentate. Metafemur subapically without stridulatory patch. Abdominal ventrite 5 flat, microreticulate, punctate, with sparse erect clavate scales. Penis (Fig. 73b) with apex asymmetrically extended to the left; apical extension of medium-size; basal orifice ventrally with rim; apodemes short, 0.5 × as long as body; ductus ejaculatorius with bulbus. **Intraspecific variation.** Length 1.90–2.11 mm. Color of body bright ferruginous or darker, almost black. Female rostrum dorsally in apical half subglabrous, densely punctate; epistome simple. Profile of elytral apex in females more slender than in males, more distinctly curved ventrad.

##### Material examined.

Holotype (MZB): ARC2331 (EMBL # LM655784), E-Java Prov., Banyuwangi Reg., Meru Betiri N.P., Sukamade, sample 2, S08°32.221', E113°51.985', 260 m, 12/13-IV-2011. Paratypes (MZB, SMNK, ZSM): E-Java Prov., Banyuwangi Reg., Meru Betiri N.P., Sukamade: 17 exx, ARC2328 (EMBL # LM655781), ARC2329 (EMBL # LM655782), ARC2330 (EMBL # LM655783), same data as holotype; 1 ex, sample 4, S08°32.163', E113°51.904', 340 m, 13-IV-2011.

##### Distribution.

E-Java Prov. (Meru Betiri N.P.). Elevation: 260–340 m.

##### Etymology.

This epithet is based on the Indonesian word for “one” and is treated as a noun in apposition.

##### Notes.

*Trigonopterus satu* Riedel, sp. n. was coded as “*Trigonopterus* sp. 341”.

#### 74. Trigonopterus schulzi

Taxon classificationAnimaliaColeopteraCurculionidae

Riedel sp. n.

http://zoobank.org/3FB9B2F0-8C3F-4567-911B-D8E193E93336

##### Diagnostic description.

**Figure 74.**
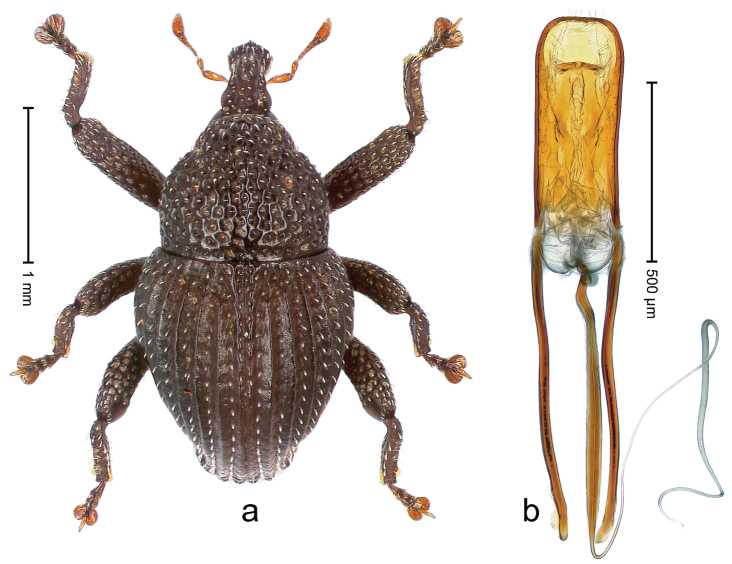
*Trigonopterus schulzi* Riedel, sp. n., holotype; **a** Habitus **b** Penis.

Holotype, male (Fig. 74a). Length 2.45 mm. Color of antennae light ferruginous; remainder dark ferruginous. Body subovate; in dorsal aspect with marked constriction between pronotum and elytron; with distinct constriction in profile. Rostrum basally with pair of indistinct submedian ridges; at middle with marked, anteriorly hollow protrusion, posteriorly with median tooth and pair of lateral teeth; between protuberance and epistome relatively flat, subglabrous, microreticulate; epistome at middle with dorsoposteriad directed horn, laterally with pair of denticles. Pronotum coarsely punctate-reticulate, each puncture with one clavate suberect scale; with indistinct subapical constriction. Elytra with striae deeply impressed, each with row of suberect clavate scales; intervals subcarinate, microreticulate. Metafemur subapically with stridulatory patch. Abdominal ventrite 5 transversely impressed, with sparse coarse punctures. Penis (Fig. 74b) with sides of body subparallel; apex rounded; apodemes 1.8 × as long as body; transfer apparatus digitiform, confluent with basally sclerotized and swollen ductus ejaculatorius; subapically ductus ejaculatorius with indistinct bulbus. **Intraspecific variation.** Length 2.32–2.45 mm. Horn of rostral epistome slightly shorter or longer. Female unknown.

##### Material examined.

Holotype (SMNK): ARC1438 (EMBL # LM655552), PHILIPPINES, Palawan, Puerto Princessa region, Sabang, Mt. Bloomfield, N10°11.62', E118°52.35', 500–700 m, 10-XII-2009. Paratypes (SMNK): 2 exx, ARC1437 (EMBL # LM655551), ARC1439 (EMBL # LM655553), same data as holotype.

##### Distribution.

Palawan (Mt. Bloomfield). Elevation: ca. 500–700 m.

##### Etymology.

This species is named in honour of Andreas Schulz (Leverkusen, Germany), a successful collector of edaphic arthropods.

##### Notes.

*Trigonopterus schulzi* Riedel, sp. n. was coded as “*Trigonopterus* sp. 343” by Tänzler et al. (2014).

#### 75. Trigonopterus sebelas

Taxon classificationAnimaliaColeopteraCurculionidae

Riedel sp. n.

http://zoobank.org/82A23849-CAED-4548-AC75-991D4B7F6F3A

##### Diagnostic description.

**Figure 75.**
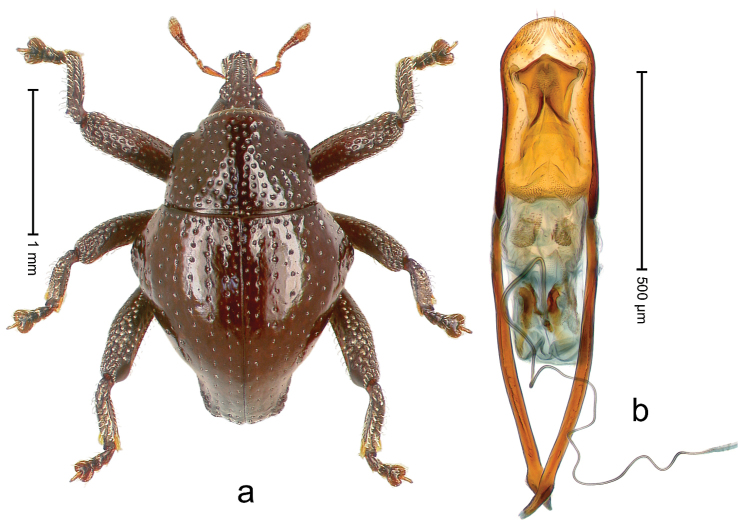
*Trigonopterus sebelas* Riedel, sp. n., holotype; **a** Habitus **b** Penis.

Holotype, male (Fig. 75a). Length 2.24 mm. Color of antennae light ferruginous, pronotum black, remainder dark ferruginous. Body in dorsal aspect subrhomboid, with marked constriction between pronotum and elytron; in profile dorsally convex. Rostrum with median ridge bordered by rows of coarse punctures; with sparse suberect scales; epistome with transverse, subangulate ridge bearing 5 denticles. Pronotum with indistinct subapical constriction; anteriorly with flanges projecting laterally; sides subparallel; disk densely punctate; interspaces subglabrous; each puncture containing small recumbent seta. Elytra with humeri swollen, laterally projecting subangularly, with coarse punctures; striae marked by small punctures, each containing minute seta; intervals flat, subglabrous; elytral apex subtruncate, in apical aspect ventral outline concave. Femora with anteroventral ridge, in meso- and metafemur with denticle. Metafemur subapically with stridulatory patch. Dorsal edge of tibiae subbasally angulate. Abdominal ventrites 1–2 forming common cavity, at middle flat, subglabrous, with sparse erect scales; laterally with distinct rim; abdominal ventrite 5 weakly concave, coarsely punctate, with sparse erect scales. Penis (Fig. 75b) with sides of body subparallel, in apical half with shallow constriction; apex rounded; behind ostium with pair of large sclerites; transfer apparatus digiform, held by frame of two parallel sclerites; apodemes 1.9 × as long as body; ductus ejaculatorius without bulbus. **Intraspecific variation.** Length 1.78–2.36 mm. Body of small specimens more slender, with elytral humeri less distinctly projecting, lateral flanges of pronotum less distinct. Female rostrum slender, dorsally subglabrous, with submedian rows of coarse punctures, sublaterally punctate-rugose; epistome simple. Female elytral humeri less prominent, evenly convex.

##### Material examined.

Holotype (MZB): ARC0715 (EMBL # LM655540), E-Kalimantan Prov., Berau Dist., Hutan Wisata Sei Tangap, ca. 8 km W of Tanjungredeb, N02°08.07', E117°24.65', 30 m, 02-X-2008. Paratypes (MZB, SMNK, ZSM): E-KALIMANTAN, Berau Dist.: 26 exx, ARC0714 (EMBL # LM655539), ARC0716 (EMBL # LM655541), ARC0717 (EMBL # LM655542), ARC0718 (EMBL # LM655543), ARC0719 (EMBL # LM655544), ARC0720 (EMBL # LM655545), ARC0721 (EMBL # LM655546), same data as holotype; 3 exx, Hutan Mayang Mangurai, ca. 15 km SW of Tanjungredeb, N02°06.217', E117°24.003‘, 20 m, 30-XI-2008; 15 exx, ARC1418, ARC1419, ARC1420, ARC1426 (PCR failed), ca. 45 km N of Tanjungredeb, 1 km off road Tanjungredeb - Tanjungselor, N02°29.55', E117°28.77', 190 m, 29.IX./3.X.2008; 2 exx, near Kampung Suaran, ca 40 km S of Tanjungredeb, primary forest on limestone, N01°59.70', E117°36.05', 50 m, 01-X-2008.

##### Distribution.

E-Kalimantan Prov. (Tanjungredeb). Elevation: 20–190 m.

##### Etymology.

This epithet is based on the Indonesian word for “eleven” and is treated as a noun in apposition.

##### Notes.

*Trigonopterus sebelas* Riedel, sp. n. was coded as “*Trigonopterus* sp. 308” by Tänzler et al. (2014).

#### 76. Trigonopterus sembilan

Taxon classificationAnimaliaColeopteraCurculionidae

Riedel sp. n.

http://zoobank.org/D8E88636-C39C-43AF-BF08-3A89FF82E118

##### Diagnostic description.

**Figure 76.**
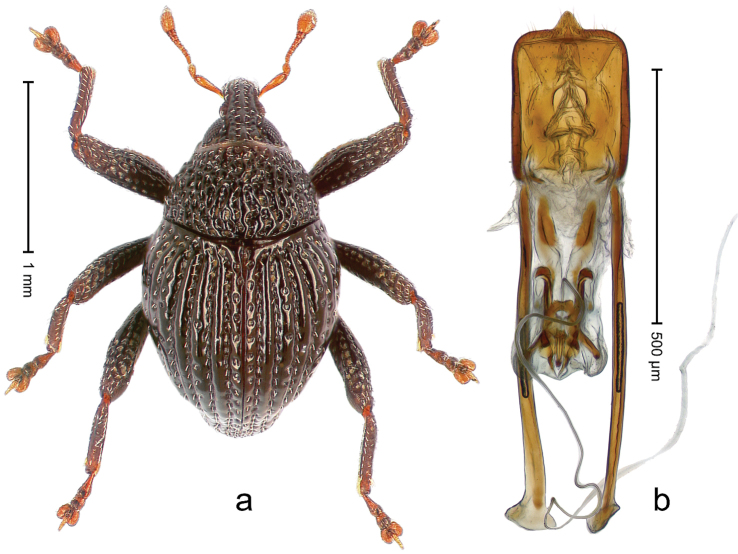
*Trigonopterus sembilan* Riedel, sp. n., holotype; **a** Habitus **b** Penis.

Holotype, male (Fig. 76a). Length 1.84 mm. Color of antennae, tarsi and tibiae ferruginous; remainder black. Body subovate, in dorsal aspect and in profile with weak constriction between pronotum and elytron. Rostrum with median ridge and pair of indistinct submedian ridges, intervening furrows each with sparse row of mesad directed setae; anterior third scabrous; epistome weakly swollen, simple. Pronotum coarsely punctate, laterally reticulate, submedially interspaces longitudinally rugose, with median costa; with sparse, subrecumbent setae. Elytra with striae deeply impressed; each with row of suberect setae; intervals costate, subglabrous; intervals 1, 3, 5 more distinctly swollen; sutural interval with row of coarse punctures, intervals 3 and 5 with few scattered punctures. Femora with simple, crenate anteroventral ridge. Metafemur subapically with stridulatory patch. Metatibia apically with uncus, without premucro. Abdominal ventrite 5 coarsely punctate, with sparse recumbent setae, at middle with shallow impression. Penis (Fig. 76b) with sides of body subparallel; apex sparsely setose, with median triangular extension; endophallus with pair of elongate sclerites; transfer apparatus compact, symmetrical, with two pairs of tendons attached; laterally with pair of subparallel sclerites, markedly bent at middle, diverging to apex of endophallus; apodemes 2.1 × as long as body; ductus ejaculatorius without bulbus. **Intraspecific variation.** Length 1.84–2.00 mm. Female rostrum in apical half dorsally subglabrous, with submedian rows of small punctures; epistome simple. Female abdominal ventrite 5 flat.

##### Material examined.

Holotype (MZB): ARC1499 (EMBL # LM655597), West Nusa Tenggara Prov., Sumbawa, Tepal, Pc. Nengas, sample 2, S08°35.884', E117°08.384', 1310 m, 15-IV-2010 (MZB). Paratypes (MZB, SMNK, ZSM): West Nusa Tenggara Prov., Sumbawa: 2 exx, ARC1500 (EMBL # LM655598), ARC1501 (EMBL # LM655599), same data as holotype; 2 exx, Tepal, Pc. Nengas, sample 7, S08°35.176', E117°08.295', 1490 m, 16-IV-2010; 3 exx, ARC1519 (EMBL # LM655617), ARC1520 (EMBL # LM655618), ARC1521 (EMBL # LM655619), Batu Dulang, Mt. Batu Pasak, sample 2, S08°37.028', E117°15.783', 1305 m, 12-IV-2010.

##### Distribution.

West Nusa Tenggara Prov., Sumbawa (Batu Dulang, Tepal). Elevation: 1305–1490 m.

##### Etymology.

This epithet is based on the Indonesian word for “nine” and is treated as a noun in apposition.

##### Notes.

*Trigonopterus sembilan* Riedel, sp. n. was coded as “*Trigonopterus* sp. 283”.

#### 77. Trigonopterus sepuluh

Taxon classificationAnimaliaColeopteraCurculionidae

Riedel sp. n.

http://zoobank.org/40D3334D-CBA5-4B5E-88CC-ED054A39BD6C

##### Diagnostic description.

**Figure 77.**
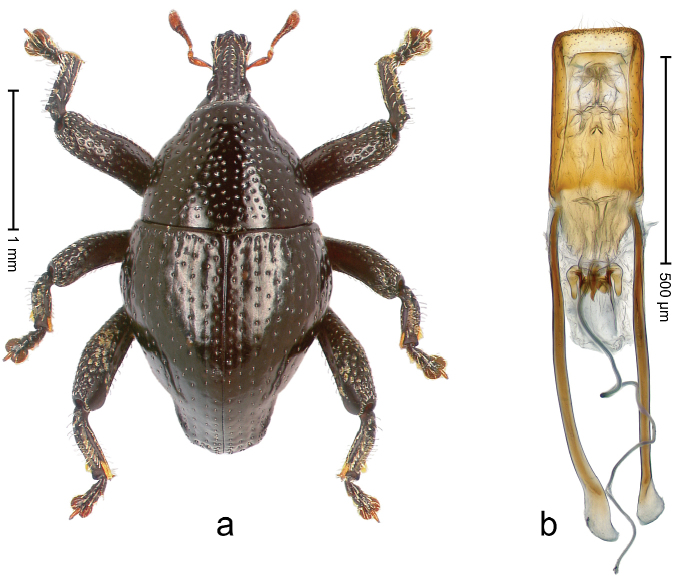
*Trigonopterus sepuluh* Riedel, sp. n., holotype; **a** Habitus **b** Penis.

Holotype, male (Fig. 77a). Length 2.53 mm. Color black; antennae and tarsi ferruginous. Body in dorsal aspect subovate, with weak constriction between pronotum and elytron; in profile dorsally convex. Rostrum with median and pair of indistinct submedian ridges; intervening furrows containing row of coarse punctures, with rows of erect scales; epistome with subangulate ridge bearing five denticles. Head with front margin of eye bordered by distinct furrow. Pronotum with disk densely punctate with small punctures; interspaces subglabrous; each puncture containing small recumbent seta. Elytra with striae marked by small punctures; intervals flat, weakly microreticulate, with small interspersed punctures, especially on sutural interval; elytral apex subtruncate, in apical aspect ventral outline weakly bisinuate. Metafemur subapically with stridulatory patch. Dorsal edge of tibiae with subbasal angulation, dentate in pro- and mesotibia. Abdominal ventrites 1–2 subglabrous, with few long erect narrow scales, laterally with swollen rim; abdominal ventrite 2 in profile projecting dentiform; abdominal ventrite 5 flat, basally with indistinct transverse costa, with sparse coarse punctures and suberect scales. Penis (Fig. 77b) with sides of body subparallel; apex rounded, with sparse setae; transfer apparatus small, complex, wider than long; apodemes 1.9 × as long as body; ductus ejaculatorius without bulbus. **Intraspecific variation.** Length 2.16–2.55 mm. Female body between humeri slightly more slender. Female rostrum dorsally subglabrous, coarsely punctate, interspaces microreticulate; epistome simple.

##### Material examined.

Holotype (MZB): ARC2538 (EMBL # LM655875), W-Kalimantan Prov., Bengkayan, Suka-Bangun, Mt. Bawang, sample 5, N00°54.103', E109°22.515', 652 m, 11-XII-2011 (MZB). Paratypes (MZB, SMNK, ZSM): 8 exx, ARC2539 (EMBL # LM655876), ARC2540 (EMBL # LM655877), ARC2541 (EMBL # LM655878), same data as holotype.

##### Distribution.

W-Kalimantan Prov. (Mt. Bawang). Elevation: 652 m.

##### Etymology.

This epithet is based on the Indonesian word for “ten” and is treated as a noun in apposition.

##### Notes.

*Trigonopterus sepuluh* Riedel, sp. n. was coded as “*Trigonopterus* sp. 365” by Tänzler et al. (2014).

#### 78. Trigonopterus seriatus

Taxon classificationAnimaliaColeopteraCurculionidae

Riedel sp. n.

http://zoobank.org/FDA4C65D-9753-4F85-8A1A-10B82B67ACE3

##### Diagnostic description.

**Figure 78.**
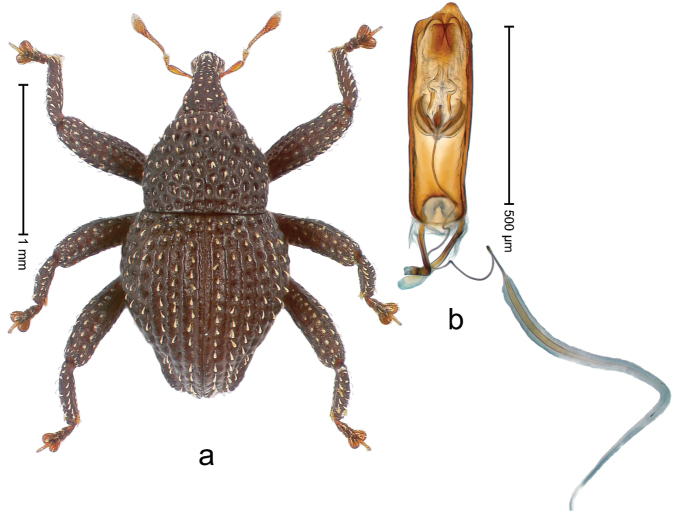
*Trigonopterus seriatus* Riedel, sp. n., holotype; **a** Habitus **b** Penis.

Holotype, male (Fig. 78a). Length 2.00 mm. Color of legs and head ferruginous; remainder black. Body subovate; in dorsal aspect and in profile with distinct constriction between pronotum and elytron. Rostrum irregularly rugose-punctate; with short, clavate, suberect scales; epistome at middle with indistinct, curved ridge. Pronotum with shallow subapical constriction; disk coarsely punctate; each puncture containing erect clavate scale inserting at posterior rim; surface dull, microreticulate. Elytra with striae deeply impressed, in front of each puncture with claviform, suberect scale; intervals costate; surface dull, coriaceous, microreticulate; interval 7 subapically forming distinct ridge. Femora edentate. Metafemur subapically without stridulatory patch. Abdominal ventrite 5 flat, microreticulate, with sparse scales. Penis (Fig. 78b) with apex asymmetrically extended on the left; apical extension short; basal orifice ventrally without rim; apodemes short, 0.5 × as long as body; ductus ejaculatorius with bulbus. **Intraspecific variation.** Length 1.80–2.14 mm. Female rostrum dorsally in apical half subglabrous, with small punctures; epistome simple.

##### Material examined.

Holotype (MZB): ARC2730 (EMBL # LM655986), E-Sumatra,, Lampung Prov., Bukit Barisan Selatan N.P., Kota Agung, Sukaraja, S05°31.678', E104°25.508', 608 m, 02-V-2012. Paratypes (MZB, SMNK, ZSM): E-Sumatra, Lampung Prov.: 5 exx, ARC0271 (EMBL # LM655462), Bawang, Pedada Bay, Mt. Tanggang, sample 1, S05°43.933', E105°06.598', 579 m, 09-VIII-2006; 5 exx, ARC0268 (EMBL # LM655459), ARC0269 (EMBL # LM655460), ARC0270 (EMBL # LM655461), Bawang, Pedada Bay, Mt. Tanggang, sample 2, S05°43.947', E105°06.480', 659 m, 09-VIII-2006; 1 ex, Bawang, Pedada Bay, Mt. Tanggang, sample 3, S05°43.938', E105°06.440', 673 m, 09-VIII-2006; 4 exx, ARC0272 (EMBL # LM655463), Bawang, Pedada Bay, Mt. Tanggang, sample 4, S05°43.871', E105°06.393', 744 m, 09-VIII-2006; 4 exx, ARC2727 (EMBL # LM655983), ARC2728 (EMBL # LM655984), Bukit Barisan Selatan N.P., Kota Agung, Sukaraja, sample 2, S05°30.330', E104°25.838', 660 m, 01-V-2012; 2 exx, Bukit Barisan Selatan N.P., Kota Agung, Sukaraja, sample 3, S05°32.289', E104°26.295', 495 m, 01-V-2012; 11 exx, Bukit Barisan Selatan N.P., Kota Agung, Sukaraja, sample 4, S05°33.406', E104°25.680', 405 m, 01-V-2012; 2 exx, Bukit Barisan Selatan N.P., Kota Agung, Sukaraja, sample 5, S05°31.044', E104°25.664', 562 m, 02-V-2012; 1 ex, Bukit Barisan Selatan N.P., Kota Agung, Sukaraja, sample 6, S05°31.678', E104°25.508', 608 m, 02-V-2012; 2 exx, Bukit Barisan Selatan N.P., Kota Agung, Sukaraja, sample 7, S05°33.768', E104°25.361', 371 m, 02-V-2012; 1 ex, Bukit Barisan Selatan N.P., Kota Agung, Sukaraja, sample 8, S05°33.032', E104°25.985', 421 m, 02-V-2012; 1 ex, Bukit Barisan Selatan N.P., Kota Agung, Sukaraja, sample 9, S05°32.611', E104°26.353', 455 m, 02-V-2012.

##### Distribution.

Lampung Prov. (Bukit Barisan Selatan N.P., Pedada Bay). Elevation: 371–744 m.

##### Etymology.

This epithet is based on the Latin adjective *seriatus* (arranged in rows) and refers to the elytral scales.

##### Notes.

*Trigonopterus seriatus* Riedel, sp. n. was coded as “*Trigonopterus* sp. 339”.

#### 79. Trigonopterus serratifemur

Taxon classificationAnimaliaColeopteraCurculionidae

Riedel sp. n.

http://zoobank.org/05A5185A-017E-44A1-952F-A6C2A7D10865

##### Diagnostic description.

**Figure 79.**
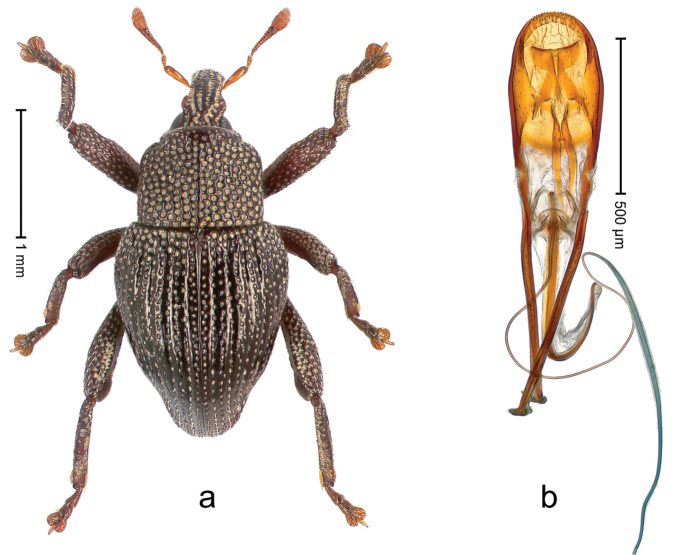
*Trigonopterus serratifemur* Riedel, sp. n., holotype; **a** Habitus **b** Penis.

Holotype, male (Fig. 79a). Length 2.46 mm. Color of antennae and legs ferruginous; remainder black. Body in dorsal aspect subovate, with marked constriction between pronotum and elytron; in profile dorsally flattened, apex convex. Rostrum in basal half with median and pair of submedian ridges becoming indistinct apically; intervening furrows each with row of coarse punctures and suberect piliform scales; epistome with indistinct, transverse ridge. Pronotum with sides subparallel, anteriorly with distinct subapical constriction; disk coarsely punctate, reticulate; with indistinct median ridge; sparsely setose with minute setae. Elytra with striae distinct; intervals flat, each with row of small punctures; subbasally punctures more dense and confused; each puncture containing small recumbent scale. Profemur with anteroventral ridge indistinct; meso- and metafemur anteroventrally serrate, bearing row of large sharp teeth, in apical 1/3 simple. Metafemur subapically with stridulatory patch. Dorsal edge of tibia subbasally with angulation, in mesotibia with distinct tooth. Abdominal ventrite 5 flat, coarsely punctate. Penis (Fig. 79b) with sides converging to subangulate, denticulate apex; in profile body bent ventrad at right angle; transfer apparatus flagelliform, 2.0 × longer than body; apodemes 2.0 × as long as body; ductus ejaculatorius with bulbus. **Intraspecific variation.** Length 2.20–2.70 mm. Female rostrum dorsally punctate-rugose, with median and pair of submedian subglabrous costae; epistome simple.

##### Material examined.

Holotype (MZB): ARC2236 (EMBL # LM655692), East Nusa Tenggara Prov., Flores, Labuhan Bajo, Tebedo, sample 1: S08°29.848', E119°59.633', 525 m, 14-III-2011. Paratypes (MZB, SMNK, ZSM): 12 exx, ARC2237 (EMBL # LM655693), ARC2238 (EMBL # LM655694), ARC2239 (EMBL # LM655695), ARC2240 (EMBL # LM655696), same data as holotype; 1 ex, ARC2223 (EMBL # LM655679), Flores, Labuhan Bajo, Roe, sample 5, S08°35.395', E120°00.383', 790 m, 13-III-2011; 1 ex, ARC2229 (EMBL # LM655685), Flores, Labuhan Bajo, Roe, sample 3, S08°36.259', E120°01.539', 975 m, 13-III-2011.

##### Distribution.

East Nusa Tenggara Prov., Flores (Roe, Tebedo). Elevation: 525–975 m.

##### Etymology.

This epithet is a combination of the Latin nouns *serra* (saw) and *femur* (thigh) and is to be treated as noun in apposition.

##### Notes.

*Trigonopterus serratifemur* Riedel, sp. n. was coded as “*Trigonopterus* sp. 351”.

#### 80. Trigonopterus setifer

Taxon classificationAnimaliaColeopteraCurculionidae

Riedel sp. n.

http://zoobank.org/438D15CE-1842-4ADD-BF5F-307D8FD5FD64

##### Diagnostic description.

**Figure 80.**
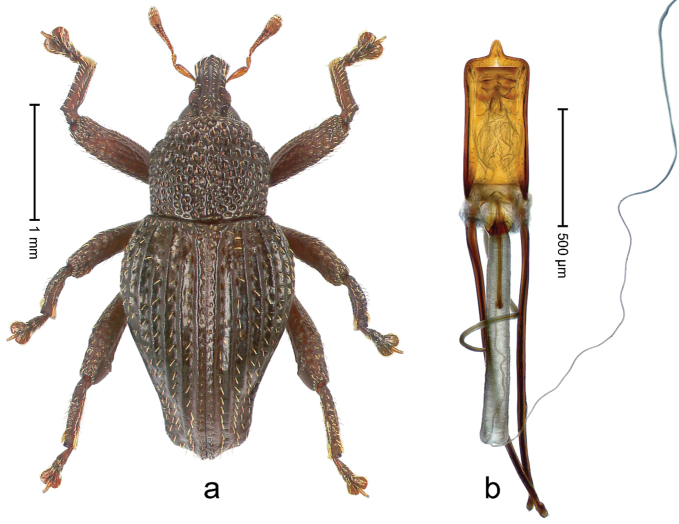
*Trigonopterus setifer* Riedel, sp. n., holotype; **a** Habitus **b** Penis.

Holotype, male (Fig. 80a). Length 3.06 mm. Color ferruginous, sides of elytra black. Body elongate; in dorsal aspect with marked constriction between pronotum and elytron; with distinct constriction in profile. Rostrum with median carina terminating abruptly on forehead; with pair of submedian ridges; intervening furrows each with row of erect, piliform scales; epistome at middle with minute denticle. Pronotum with distinct subapical constriction; disk densely punctate, interspaces partly rugose; punctures each with one piliform scale, in apical half these scales long, suberect. Elytra with striae deeply impressed, each with row of suberect piliform scales; intervals carinate, nude; intervals 2 and 4 basally flat; interval 7 swollen subapically, laterally weakly projecting; sutural interval at apex slightly protruding. Meso- and metafemur with crenulate anteroventral ridge terminating with blunt tooth. Metafemur subapically with stridulatory patch and transverse row of denticles. Abdominal ventrite 5 basally swollen, subapically with pit, sparsely setose. Penis (Fig. 80b); apex with narrow median extension; endophallus in repose basally passing middle of apodemes; transfer apparatus flagelliform, 2 × as long as body, outside of body, from ventrally coiled dorsad over apodeme and endophallic tube; apodemes 2.3 × as long as body; ductus ejaculatorius without bulbus. **Intraspecific variation.** Length 2.56–3.22 mm. Female rostrum dorsally with pairs of lateral and submedian furrows; epistome simple. Female abdominal ventrite 5 flat.

##### Material examined.

Holotype (MZB): ARC2713 (EMBL # LM655969), West Java, Garut, Cikajang, Mt. Payung, sample 3, S07°25.320', E107°48.259', 1560 m, 26-IV-2012. Paratypes (MZB, SMNK): W-Java Prov., Garut, Cikajang, Mt. Payung: 4 exx, ARC2704 (EMBL # LM655960), ARC2705 (EMBL # LM655961), ARC2709 (EMBL # LM655965), sample 2, S07°25.268', E107°48.492', 1250 m, 26-IV-2012; 1 ex, same data as holotype.

##### Distribution.

W-Java Prov. (Mt. Payung). Elevation: 1250–1560 m.

##### Etymology.

This epithet is a combination of the Latin noun *seta* (bristle) and the suffix –*fer* (bear, carry) and refers to the elytral vestiture.

##### Notes.

*Trigonopterus setifer* Riedel, sp. n. was coded as “*Trigonopterus* sp. 374”.

#### 81. Trigonopterus silvestris

Taxon classificationAnimaliaColeopteraCurculionidae

Riedel sp. n.

http://zoobank.org/4D345252-97B1-453F-ADE6-1F77BE5926A4

##### Diagnostic description.

**Figure 81.**
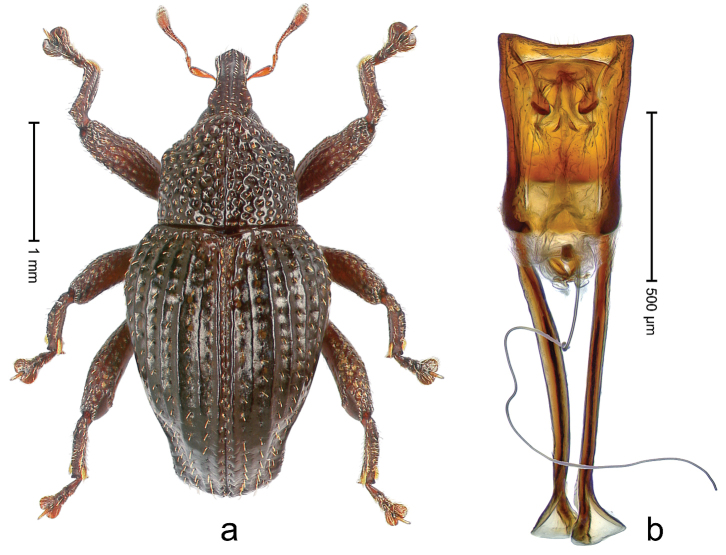
*Trigonopterus silvestris* Riedel, sp. n., holotype; **a** Habitus **b** Penis.

Holotype, male (Fig. 81a). Length 3.25 mm. Color of legs and head ferruginous, remainder black. Body elongate; in dorsal aspect with marked constriction between pronotum and elytron; in profile dorsally with indistinct constriction. Rostrum with median carina terminating on forehead; with pair of submedian ridges; intervening furrows each with sparse row of erect, piliform scales; epistome medially with transverse ridge, without denticle. Pronotum anterolaterally subangularly projecting; without distinct subapical constriction; disk coarsely rugose-punctate; each puncture containing suberect piliform scale; with median ridge. Elytra with striae deeply impressed; each with row of suberect piliform scales; intervals weakly carinate; costa of interval 2 shortened at base; interval 7 swollen subapically, laterally weakly projecting; apex extended ventrad, beak-shaped. Meso- and metafemur with crenulate anteroventral ridge terminating with blunt tooth. Metafemur subapically with stridulatory patch and transverse row of denticles. Abdominal ventrite 5 almost flat, subapically with shallow pit. Penis (Fig. 81b) with body in profile moderately curved ventrad; in dorsal aspect sides subparallel in basal half, anteriorly gently diverging; apex subangulate, without median extension; apodemes 2.0 × as long as body; ductus ejaculatorius without bulbus. **Intraspecific variation.** Length 2.79–3.34 mm. Female rostrum dorsally with pairs of lateral and submedian furrows; epistome simple. Elytral apex in males rounded; in females narrower, with distinct median notch. Female abdominal ventrite 5 flat.

##### Material examined.

Holotype (MZB): ARC2668 (EMBL # LM655926), West Java Prov., Sumedang, Sukajadi, Mt. Cakrabuana, sample 3, S07°01.493', E108°08.065', 1394 m, 19-IV-2012. Paratypes (MZB, SMNK, ZSM): W-Java Prov., Sumedang, Sukajadi, Mt. Cakrabuana: 7 exx, ARC2669 (EMBL # LM655927), ARC2670 (EMBL # LM655928), same data as holotype; 1 ex, sample 2, S07°01.243', E108°08.056', 1292 m, 19-IV-2012; 2 exx, sample 4, S07°01.734', E108°08.059', 1530 m, 19-IV-2012; 4 exx, sample 5, S07°01.804', E108°08.044', 1563 m, 20-IV-2012.

##### Distribution.

W-Java Prov. (Mt. Cakrabuana). Elevation: 1292–1563 m.

##### Etymology.

This epithet is based on the Latin adjective *silvestris* (living in the forest) and refers to the species habitat.

##### Notes.

*Trigonopterus silvestris* Riedel, sp. n. was coded as “*Trigonopterus* sp. 373”.

#### 82. Trigonopterus singkawangensis

Taxon classificationAnimaliaColeopteraCurculionidae

Riedel sp. n.

http://zoobank.org/E70E6F90-B224-4219-A0CA-F8A21F45CA68

##### Diagnostic description.

**Figure 82.**
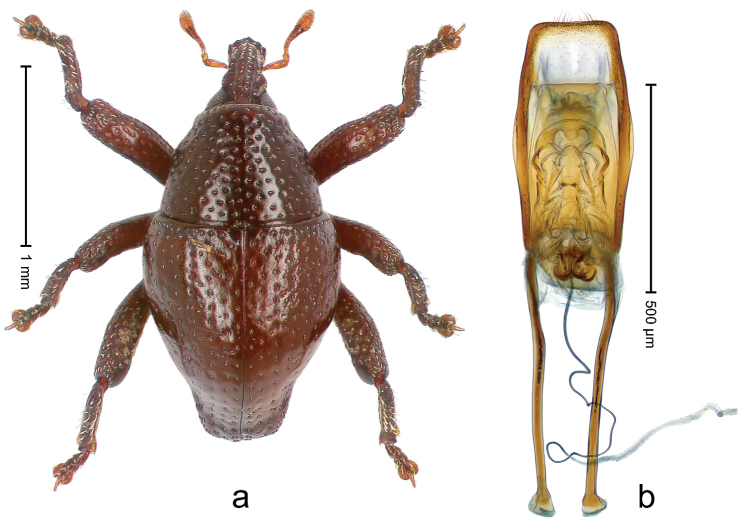
*Trigonopterus singkawangensis* Riedel, sp. n., holotype; **a** Habitus **b** Penis.

Holotype, male (Fig. 82a). Length 1.90 mm. Color ferruginous, pronotum almost black. Body in dorsal aspect subovate, with weak constriction between pronotum and elytron; in profile dorsally convex. Rostrum with median and pair of indistinct submedian ridges; chagreened, with sparse rows of subrecumbent scales; in front of forehead median ridge terminating, projecting from profile; epistome with subangulate ridge. Pronotum with disk densely punctate; interspaces subglabrous, microreticulate; each puncture containing small recumbent seta. Elytra with striae indistinct; punctuation almost confused, punctures small; intervals flat, microreticulate; elytral apex subtruncate. Anteroventral ridge of femora terminating in apical third. Metafemur subapically with stridulatory patch. Dorsal edge of tibiae with subbasal angulation, dentate in pro- and mesotibia. Abdominal ventrites 1–2 at middle concave, subglabrous, anteriorly with few long erect narrow scales, laterally with swollen rim; abdominal ventrite 2 posteriorly with transverse ridge, in profile projecting dentiform; abdominal ventrite 5 basally with transverse costa, at middle weakly concave, subglabrous, laterally punctate and with sparse suberect scales. Penis (Fig. 82b) with sides of body subparallel, near middle widened; apex subtruncate, sparsely setose; transfer apparatus complex, small; apodemes 1.3 × as long as body; ductus ejaculatorius without distinct bulbus. **Intraspecific variation.** Length 1.90–2.32 mm. Female rostrum dorsally subglabrous, with submedian rows of coarse punctures; epistome simple. Elytral punctuation almost confused or with distinct striae.

##### Material examined.

Holotype (MZB): ARC2547 (EMBL # LM655884), W-Kalimantan Prov., Singkawang, Mt. Poteng, sample 3, N00°51.211', E109°03.418', 651 m, 13-XII-2011. Paratypes (SMNK): 1 ex, ARC2548 (EMBL # LM655885), same data as holotype.

##### Distribution.

W-Kalimantan Prov. (Singkawang). Elevation: 651 m.

##### Etymology.

This epithet is based on the type locality.

##### Notes.

*Trigonopterus singkawangensis* Riedel, sp. n. was coded as “*Trigonopterus* sp. 367”.

#### 83. Trigonopterus singularis

Taxon classificationAnimaliaColeopteraCurculionidae

Riedel sp. n.

http://zoobank.org/6D189A5B-570D-42E8-9005-42F6D5C5C199

##### Diagnostic description.

**Figure 83.**
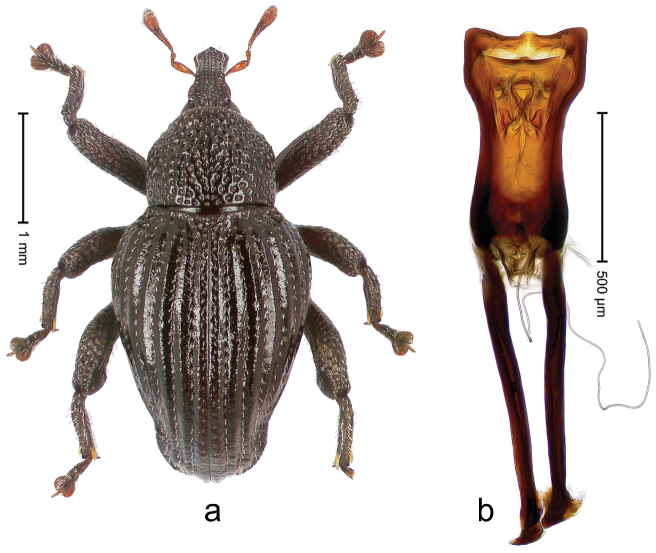
*Trigonopterus singularis* Riedel, sp. n., holotype; **a** Habitus **b** Penis.

Holotype, male (Fig. 83a). Length 3.44 mm. Color of tarsi and antennae ferruginous, remainder black. Body elongate; in dorsal aspect with marked constriction between pronotum and elytron; in profile dorsally with indistinct constriction. Rostrum with median carina terminating on forehead; with pair of submedian ridges; intervening furrows each with row of erect, piliform scales; epistome with transverse, angulate ridge forming minute median denticle. Pronotum without distinct subapical constriction; disk densely punctate, interspaces glabrous; each puncture containing one small scale. Elytra with striae deeply impressed; each with row of narrow, suberect scales; intervals weakly carinate, nude, coriaceous; interval 7 swollen subapically, laterally weakly projecting; apex extended ventrad, beak-shaped. Metafemur with crenulate anteroventral ridge terminating with small tooth; subapically with stridulatory patch and transverse row of denticles. Metatibia in basal half with dorsal edge denticulate. Abdominal ventrite 5 flat, subapically with shallow pit. Penis (Fig. 83b) with body in profile markedly curved ventrad; in dorsal aspect sides basally concave, before apex subparallel; apex medially with small rounded extension; apodemes 1.6 × as long as body; ductus ejaculatorius without bulbus. **Intraspecific variation.** Length 3.06–3.44 mm. Female rostrum dorsally with pair of lateral furrows, and pair of more shallow submedian furrows; epistome simple. Elytral apex in males rounded, in females narrower, subangulate. Female abdominal ventrite 5 flat. Penis of specimen ARC2747 narrow, sides subparallel from base to shortly before apex.

##### Material examined.

Holotype (MZB): ARC0281 (EMBL # LM655471), East Sumatra, Lampung Prov., Kalianda, Mt. Rajabasa, sample 1, S05°46.896', E105°37.687', 1281 m, 15-VIII-2006. Paratypes (MZB, SMNK): E-Sumatra, Lampung Prov., Kalianda, Mt. Rajabasa: 1 ex, ARC0282 (EMBL # LM655472), same data as holotype; 2 exx, ARC2746 (EMBL # LM656002), ARC2747 (EMBL # LM656003), sample 1, S05°46.896', E105°37.687', 1281 m, 07-V-2012; 1 ex, sample 6, S05°46.651', E105°37.574', 1257 m, 07-V-2012.

##### Distribution.

Lampung Prov. (Mt. Rajabasa). Elevation: 1257–1281 m.

##### Etymology.

This epithet is based on the Latin adjective *singularis* (alone) and refers to the fact that no other *Trigonopterus* species could be found on the type locality.

##### Notes.

*Trigonopterus singularis* Riedel, sp. n. was coded as “*Trigonopterus* sp. 321”.

#### 84. Trigonopterus sinuatus

Taxon classificationAnimaliaColeopteraCurculionidae

Riedel sp. n.

http://zoobank.org/B4422C42-BCE5-4DC5-BC23-2356A241CF00

##### Diagnostic description.

**Figure 84.**
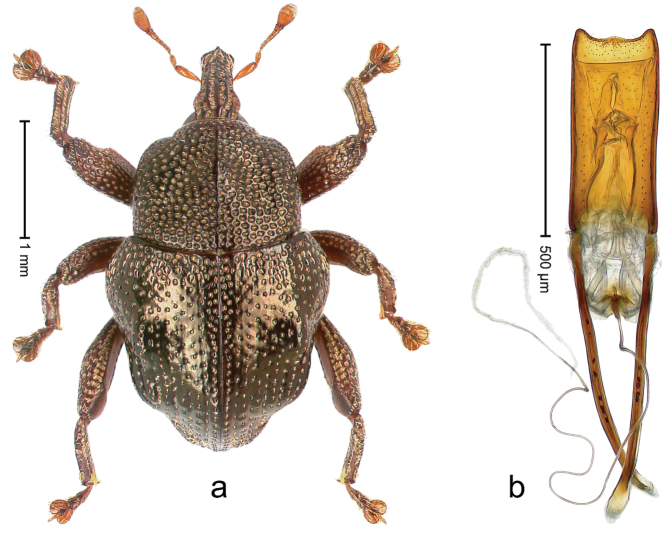
*Trigonopterus sinuatus* Riedel, sp. n., holotype; **a** Habitus **b** Penis.

Holotype, male (Fig. 84a). Length 2.95 mm. Color of legs and head ferruginous; pronotum and elytron black with bronze lustre. Body in dorsal aspect with marked constriction between pronotum and elytron; in profile dorsally convex. Rostrum in apical half scabrous, coarsely punctate; in basal half with median and pair of submedian ridges; intervening furrows punctate, each with sparse row of erect setae; epistome with transverse, angulate ridge forming distinct median denticle. Pronotum with sides subparallel, anterolaterally rounded to subapical constriction; disk with pair of submedian impressions, coarsely punctate, reticulate; each puncture containing inconspicuous seta; with distinct median ridge. Elytra with humeri swollen, laterally projecting; interval 5 near middle with distinct tubercle dorsally projecting; interval 6 behind middle swollen, gently projecting from lateral outline; striae indistinct; intervals flat, punctate; at base and laterally densely coarsely punctate, remainder less densely punctate. Anteroventral ridge of femora distinct, in meso- and metafemur forming large tooth. Metafemur subapically with stridulatory patch. Dorsal edge of tibiae subbasally with angulation, in mesotibia with blunt tooth. Abdominal ventrite 5 flat, coarsely punctate. Penis (Fig. 84b) with sides of body subparallel; apex subtruncate, weakly angulate; transfer apparatus compact; apodemes 1.6 × as long as body; ductus ejaculatorius without bulbus. **Intraspecific variation.** Length 2.23–3.06 mm. Female body more slender. Female rostrum dorsally subglabrous, with submedian rows of punctures and sublateral pair of furrows; epistome simple. Female elytra subovate, without tubercles, lateral contour convex.

##### Material examined.

Holotype (MZB): ARC1510 (EMBL # LM655608), West Nusa Tenggara Prov., Sumbawa, Batu Dulang, Mt. Batu Pasak, sample 2, S08°37.028', E117°15.783', 1305 m, 12-IV-2010. Paratypes (MZB, SMNK, ZSM): West Nusa Tenggara Prov., Sumbawa: 13 exx, ARC1509 (EMBL # LM655607), ARC1511 (EMBL # LM655609), ARC1512 (EMBL # LM655610), same data as holotype; 1 ex, Batu Dulang, Mt. Batu Pasak, sample 3, S08°37.524', E117°15.423', 1385 m, 12-IV-2010; 1 ex, Batu Dulang, Mt. Batu Pasak, sample 3, S08°37.524', E117°15.423', 1385 m, 18-IV-2010; 5 exx, ARC1493 (EMBL # LM655591), ARC1494 (EMBL # LM655592), ARC1495 (EMBL # LM655593), ARC1496 (EMBL # LM655594), Tepal, Pc. Nengas, sample 2, S08°35.884', E117°08.384', 1310 m, 15-IV-2010; 2 exx, Tepal, Pc. Nengas, sample 3, S08°35.386', E117°08.251', 1415 m, 15-IV-2010; 7 exx, Tepal, Pc. Nengas, sample 5, S08°35.740', E117°08.721', 1330 m, 16-IV-2010; 1 ex, Tepal, Pc. Nengas, sample 6, S08°35.533', E117°08.605', 1350 m, 16-IV-2010; 2 exx, Tepal, Pc. Nengas, sample 7, S08°35.176', E117°08.295', 1490 m, 16-IV-2010.

##### Distribution.

West Nusa Tenggara Prov., Sumbawa (Batu Dulang, Tepal). Elevation: 1305–1490 m.

##### Etymology.

This epithet is based on the Latin adjective *sinuatus* (bent) and refers to the elytral outline.

##### Notes.

*Trigonopterus sinuatus* Riedel, sp. n. was coded as “*Trigonopterus* sp. 333” by Tänzler et al. (2014). Cox1 sequences of the populations from Tepal and Batu Dulang differ 15.7–16.0% *p*-distance, but morphologically no differences could be found.

#### 85. Trigonopterus squalidus

Taxon classificationAnimaliaColeopteraCurculionidae

Riedel sp. n.

http://zoobank.org/6B86EF6F-A4DE-453D-8CA7-E45A6890E327

##### Diagnostic description.

**Figure 85.**
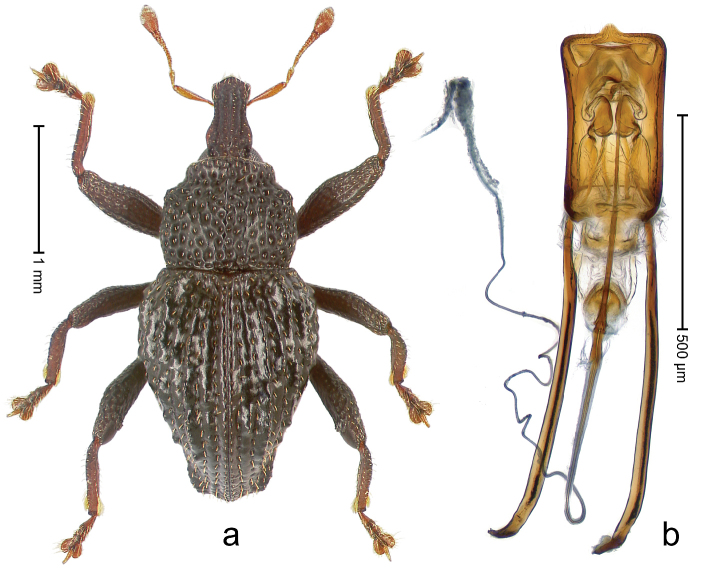
*Trigonopterus squalidus* Riedel, sp. n., holotype; **a** Habitus **b** Penis.

Holotype, male (Fig. 85a). Length 2.78 mm. Color of legs and head ferruginous, remainder black. Body elongate; in dorsal aspect with marked constriction between pronotum and elytron; with distinct constriction in profile. Rostrum with median ridge and pair of submedian ridges, intervening furrows punctate; punctures each with erect narrow scale; epistome with transverse, subangulate ridge. Pronotum anterolaterally angularly projecting; with distinct subapical constriction; disk scabrous, punctures each with one small erect scale; interspaces dull, greyish, markedly microreticulate; with irregular median ridge. Elytra with striae deeply impressed, each with row of narrow suberect scales; intervals irregularly costate-tuberculate, with minute recumbent scales; interval 5 subbasally swollen; interval 7 swollen subapically, laterally weakly projecting. Femora edentate, with crenulate anteroventral ridge. Metafemur subapically with stridulatory patch and transverse row of denticles. Abdominal ventrite 5 flat, densely punctate, setose. Penis (Fig. 85b) with body in profile moderately curved; in dorsal aspect sides subparallel, subapically slightly widened; apex with median, acute extension; transfer apparatus flagelliform, 1.5 × longer than body; apodemes 2.4 × as long as body; ductus ejaculatorius without bulbus. **Intraspecific variation.** Length 2.59–2.84 mm. Female rostrum dorsally subglabrous, with pair of lateral furrows, coarsely punctate; epistome simple.

##### Material examined.

Holotype (MZB): ARC2737 (EMBL # LM655993), East Sumatra, Lampung Prov., Gisting, Sidokaton, Mt. Tanggamus, sample 1, S05°25.475', E104°41.063', 1539 m, 03-V-2012. Paratypes (MZB, SMNK, ZSM): E-Sumatra, Lampung Prov., Gisting, Sidokaton, Mt. Tanggamus: 2 exx, ARC2741 (EMBL # LM655997), ARC2742 (EMBL # LM655998), sample 2, S05°25.491', E104°41.006', 1636 m, 03-V-2012; 4 exx, ARC2744 (EMBL # LM656000), sample 3, S05°25.508', E104°40.882', 1759 m, 03-V-2012; 3 exx, sample 4, S05°25.484', E104°41.027', 1623 m, sifted.

##### Distribution.

Lampung Prov. (Mt. Tanggamus). Elevation: 1539–1759 m.

##### Etymology.

This epithet is based on the Latin adjective *squalidus* (rough, dirty) and refers to the rough integument often covered with dirt incrustations.

##### Notes.

*Trigonopterus squalidus* Riedel, sp. n. was coded as “*Trigonopterus* sp. 371”.

#### 86. Trigonopterus sumatrensis

Taxon classificationAnimaliaColeopteraCurculionidae

Riedel sp. n.

http://zoobank.org/1E64DB33-127E-440E-B25C-6BC14E8BCCCF

##### Diagnostic description.

**Figure 86.**
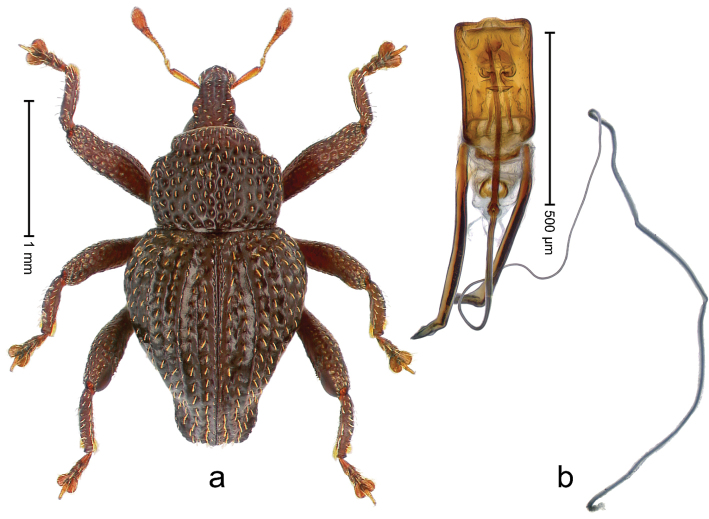
*Trigonopterus sumatrensis* Riedel, sp. n., holotype; **a** Habitus **b** Penis.

Holotype, male (Fig. 86a). Length 2.38 mm. Color of legs and antennae ferruginous, remainder dark ferruginous to black. Body subhexagonal; in dorsal aspect with marked constriction between pronotum and elytron; with distinct constriction in profile. Rostrum with median ridge and pair of submedian ridges, intervening furrows punctate; punctures each with erect narrow scale; epistome with transverse ridge. Pronotum anterolaterally angularly projecting; with distinct subapical constriction; disk coarsely punctate, each puncture containing one clavate scale; interspaces dull, microreticulate; indistinct median ridge subglabrous. Elytra with striae deeply impressed, each with row of suberect slender yellowish scales; intervals costate to carinate; intervals 1, 3, 5 more distinctly raised; interval 7 swollen subapically, laterally projecting. Profemur edentate, meso- and metafemur ventrally with little tooth. Metafemur subapically with stridulatory patch and transverse rows of denticles. Abdominal ventrite 5 flat. Penis (Fig. 86b) with body in profile markedly curved ventrad; in dorsal aspect sides subparallel; apex with short median extension rounded; transfer apparatus flagelliform, ca. 2 × as long as body; apodemes 2.2 × as long as body; ductus ejaculatorius without bulbus. **Intraspecific variation.** Length 2.28–2.55 mm. Color of body ferruginous or black. Female rostrum dorsally with pairs of lateral and submedian furrows; epistome simple. Pronotum with anterolateral knobs projecting subrectangularly in males, more acute in females.

##### Material examined.

Holotype (MZB): ARC2743 (EMBL # LM655999), East Sumatra, Lampung Prov., Gisting, Sidokaton, Mt. Tanggamus, sample 2, S05°25.491', E104°41.006', 1636 m, 03-V-2012. Paratypes (MZB, SMNK, ZSM): E-Sumatra, Lampung Prov.: 23 exx, ARC0275 (EMBL # LM655466), ARC0276 (EMBL # LM655467), ARC0277 (EMBL # LM655468), ARC0278 (EMBL # LM655469), ARC0279 (EMBL # LM655470), ARC0393, Sumberjaya, Bodongjaya, Mt. Rigis, sample 3, S05°03.140', E104°26.912', 1351 m, 12-VIII-2006; 27 exx, ARC2738 (EMBL # LM655994), ARC2739 (EMBL # LM655995), ARC2740 (EMBL # LM655996), Gisting, Sidokaton, Mt. Tanggamus, sample 1, S05°25.475', E104°41.063', 1539 m, 03-V-2012; 5 exx, Gisting, Sidokaton, Mt. Tanggamus, sample 2, S05°25.491', E104°41.006', 1636 m, 03-V-2012; 8 exx, ARC2745 (EMBL # LM656001), Gisting, Sidokaton, Mt. Tanggamus, sample 4, S05°25.484', E104°41.027', 1632 m, 03-V-2012.

##### Distribution.

Lampung Prov. (Sumberjaya, Mt. Tanggamus). Elevation: 1351–1636 m.

##### Etymology.

This epithet is based on the name of Sumatra Island.

##### Notes.

*Trigonopterus sumatrensis* Riedel, sp. n. was coded as “*Trigonopterus* sp. 300”.

#### 87. Trigonopterus sumbawensis

Taxon classificationAnimaliaColeopteraCurculionidae

Riedel sp. n.

http://zoobank.org/BF4BCE1E-0367-4FBC-8BE4-A8A681A56FCA

##### Diagnostic description.

**Figure 87.**
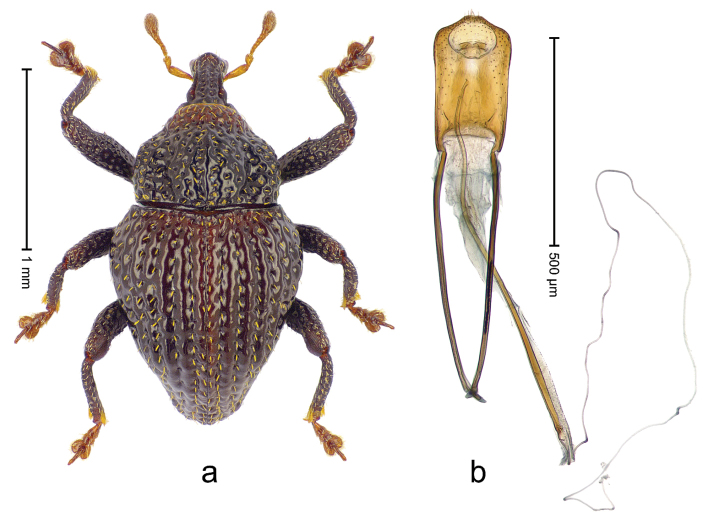
*Trigonopterus sumbawensis* Riedel, sp. n., holotype; **a** Habitus **b** Penis.

Holotype, male (Fig. 87a). Length 1.76 mm. Color of antennae and tarsi ferruginous, elytra dark ferruginous, remainder black. Body in dorsal aspect with marked constriction between pronotum and elytron; with distinct constriction in profile. Rostrum with median ridge terminating at level of antennal insertion and pair of submedian ridges, coarsely punctate; epistome with transverse, subangulate ridge. Pronotum with indistinct subapical constriction; disk coarsely punctate-rugose, sparsely setose; in basal half with pair of sublateral, kidney-shaped impressions; with median ridge. Elytra with striae deeply impressed, each with row of short, suberect scales; intervals costate, subglabrous; apex rounded. Femora with small tooth; metafemur subapically with stridulatory patch. Abdominal ventrite 5 flat, coarsely punctate. Penis (Fig. 87b) with sides of body subparallel; in apical quarter with sides angularly projecting, abruptly converging to slightly extended apex, medially with sparse setae; transfer apparatus flagelliform, 2.9 × longer than body; apodemes 2.0 × as long as body. **Intraspecific variation.** Length 1.44–1.98 mm. Body relatively slender or rather roundish (in female ARC2343). Female rostrum dorsally subglabrous, sparsely punctate, with dorsolateral pair of furrows. Elytral intervals subglabrous or with transverse wrinkles. Elytral striae with rows of suberect scales or with rows of subrecumbent setae. Femora with tooth small or minute; profemur with or without tooth.

##### Material examined.

Holotype (MZB): ARC3609 (EMBL # LM656039), West Nusa Tenggara Prov., Sumbawa, Tepal, Pc. Nengas, sample 3, S08°35.386', E117°08.251', 1415 m, 15-IV-2010. Paratypes (MZB, SMNK, ZSM): Sumbawa: 2 exx, ARC3610 (EMBL # LM656040), ARC3611 (EMBL # LM656041), same data as holotype; 4 exx, ARC1533 (EMBL # LM655631), ARC2339 (EMBL # LM655789), ARC2341 (EMBL # LM655791), ARC2343 (EMBL # LM655793), Tepal, Pc. Nengas, sample 7, S08°35.176', E117°08.295', 1490 m, 16-IV-2010; 2 exx, ARC3606 (EMBL # LM656036), ARC3608 (EMBL # LM656038), Tepal, Pc. Nengas, sample 2, S08°35.884', E117°08.384', 1310 m, 15-IV-2010; 2 exx, ARC1516 (EMBL # LM655614), ARC1517(EMBL # LM655615), Batu Dulang, Mt. Batu Pasak, sample 2, S08°37.028', E117°15.783', 1305 m, 12-IV-2010; 3 exx, ARC3653 (EMBL # LM656071), ARC3654 (EMBL # LM656072), ARC3655 (EMBL # LM656073), Batu Dulang, Mt. Batu Pasak, sample 4, S08°37.318', E117°15.339', 1280 m, 18-IV-2010.

##### Distribution.

West Nusa Tenggara Prov., Sumbawa (Batu Dulang, Tepal). Elevation: 1280–1490 m.

##### Etymology.

This epithet is based on the island of Sumbawa.

##### Notes.

*Trigonopterus sumbawensis* Riedel, sp. n. was coded as “*Trigonopterus* sp. 326” by Tänzler et al. (2014).

#### 88. Trigonopterus sundaicus

Taxon classificationAnimaliaColeopteraCurculionidae

Riedel sp. n.

http://zoobank.org/25CC7286-151B-49E4-89BB-3DB48B840452

##### Diagnostic description.

**Figure 88.**
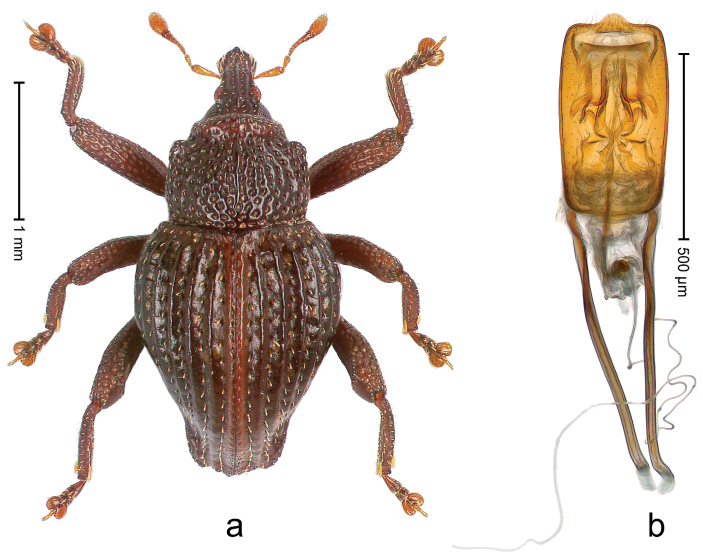
*Trigonopterus sundaicus* Riedel, sp. n., holotype; **a** Habitus **b** Penis.

Holotype, male (Fig. 88a). Length 2.70 mm. Color of head, legs, and sutural interval of elytra ferruginous, remainder black. Body subhexagonal; in dorsal aspect with marked constriction between pronotum and elytron; with distinct constriction in profile. Rostrum with median ridge and pair of submedian ridges, intervening furrows each with row of erect scales; epistome with transverse, angulate ridge forming median denticle. Pronotum anterolaterally angularly projecting; with distinct subapical constriction; disk rugose-punctate, interspaces microreticulate; punctures each with small suberect scale; with glabrous median ridge. Elytra with striae deeply impressed, each with row elongate suberect scales; intervals costate to carinate, with weak transverse wrinkles, microreticulate; interval 7 swollen subapically, laterally projecting. Femora edentate, with crenulate anteroventral ridge. Metafemur subapically with stridulatory patch and transverse rows of denticles. Abdominal ventrites 1–2 with common concavity, each sublaterally with pair of knobs; in profile ventrite 2 projecting angularly; ventrite 5 basally swollen, medially with trapezoid, shallow, polished impression. Penis (Fig. 88b) with sides of body subparallel, apex medially with rounded extension; with ca. 4 symmetrical pairs of elongate endophallic sclerites; transfer apparatus flagelliform, ca. 1.5 × as long as body; apodemes 2.0 × as long as body; ductus ejaculatorius without bulbus. **Intraspecific variation.** Length 2.12–2.83 mm. Color of body black, partly or fully ferruginous. Female rostrum dorsally with pairs of lateral and submedian furrows; epistome simple. Female abdominal ventrites 1–2 with concavity shallower, sublaterally without knobs; female abdominal ventrite 5 weakly convex, almost flat, with coarse punctures.

##### Material examined.

Holotype (MZB): ARC1536 (EMBL # LM655634), Banten Prov., Pandegelang, Mt. Karang, sample 3, S06°17.110', E106°03.263', 1145 m, 20-III-2010. Paratypes (ARC, MZB, SMNK, ZSM): 5 exx, ARC0102, W-Java Prov., Mt. Halimun N.P., Citalahab, S06°44.503', E106°31.550', 1150 m, 20-21-IX-2004; 9 exx, W-Java Prov., Mt. Halimun N.P., Citalahab, sample 1A, S06°44.503', E106°31.55', 1200 m, 11-IX-2005; 5 exx, W-Java Prov., Mt. Halimun N.P., Citalahab, sample 1, S06°44.423', E106°31.738', 1100 m, 12-IX-2005; 29 exx, ARC0208 (EMBL # LM655430), ARC0209 (EMBL # LM655431), ARC0210 (EMBL # LM655432), ARC0392, W-Java Prov., Mt. Halimun N.P., Citalahab, sample 2, S06°44.503', E106°31.550', 1200 m, 12-IX-2005; 9 exx, W-Java Prov., N Ciptarasa, sample 2, S06°50.045', E106°30.218', 1050 m, 19-IX-2005; 6 exx, ARC0189 (EMBL # LM655413), W-Java Prov., N Ciptarasa, sample 3, S06°49.867', E106°30.085', 1100 m, 19-IX-2005; 3 exx, W-Java Prov., N Ciptarasa, sample 4, S06°49.802', E106°30.020', 1150 m, 19-IX-2005; 3 exx, ARC1537 (EMBL # LM655635), ARC1539 (EMBL # LM655637), same data as holotype; 1 ex, Banten Prov., Pandegelang, Mt. Karang, sample 2, S06°17.266', E106°03.262', 1025 m, 20-III-2010; 1 ex, Banten Prov., Pandegelang, Mt. Karang, sample 3, S06°17.110', E106°03.263', 1145 m, 26-IV-2010; 1 ex, ARC1538 (EMBL # LM655636), Banten Prov., Pandegelang, Mt. Karang, sample 8, 1100 m, 26-IV-2010.

##### Distribution.

Banten Prov. (Mt. Karang), W-Java Prov. (Mt. Halimun N.P.). Elevation: 1100–1200 m.

##### Etymology.

This epithet is based on the type locality which is situated just east of the Sunda Strait; it is also part of the Sunda region of Java.

##### Notes.

*Trigonopterus sundaicus* Riedel, sp. n. was coded as “*Trigonopterus* sp. 296”.

#### 89. Trigonopterus suturalis

Taxon classificationAnimaliaColeopteraCurculionidae

Riedel sp. n.

http://zoobank.org/FA64A747-1C40-4A77-9D1E-4AA7BEF1274F

##### Diagnostic description.

**Figure 89.**
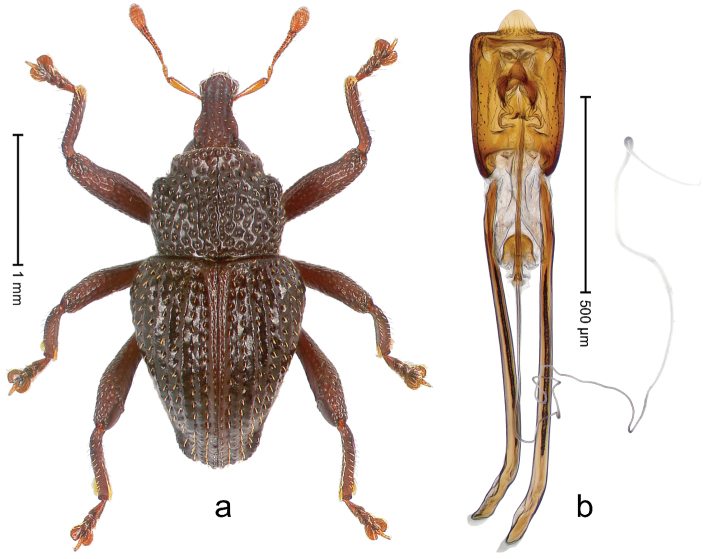
*Trigonopterus suturalis* Riedel, sp. n., holotype; **a** Habitus **b** Penis.

Holotype, male (Fig. 89a). Length 2.53 mm. Color of head, legs, and sutural interval of elytra ferruginous, remainder black. Body in dorsal aspect with marked constriction between pronotum and elytron; with distinct constriction in profile. Rostrum with median ridge and pair of submedian ridges, intervening furrows punctate; epistome with indistinct, transverse, subangulate ridge. Pronotum anterolaterally angularly projecting; with distinct subapical constriction; disk punctate, interspaces microreticulate; punctures each with one slender suberect scale; with median ridge. Elytra with striae deeply impressed, each with row of slender, suberect scales; intervals carinate, almost nude, microreticulate; sutural interval basally swollen; interval 7 swollen subapically, laterally weakly projecting. Femora edentate. Metafemur subapically with stridulatory patch and transverse ridge. Abdominal ventrite 5 flat, densely punctate, sparsely setose. Penis (Fig. 89b) with body flattened, in profile markedly curved; in dorsal aspect sides subparallel; apex with median, triangular extension; transfer apparatus flagelliform, slightly longer than body; apodemes 2.4 × as long as body; ductus ejaculatorius without bulbus. **Intraspecific variation.** Length 2.10–2.58 mm. Color of body with sutural interval ferruginous or completely black. Body shape slightly more elongate or compact. Female rostrum dorsally with pairs of lateral and submedian furrows; epistome simple.

##### Material examined.

Holotype (MZB): ARC2652 (EMBL # LM655910), West Java Prov., Bogor, Cidahu, Mt. Salak (near Javana Spa), sample 3, S06°43.425', E106°43.227', 1756 m, 13-IV-2012. Paratypes (ARC, MZB, SMNK, ZSM): W-Java Prov.: 6 exx, ARC0104, ARC0184 (EMBL # LM655409), Mt. Halimun N.P., Pasir Banteng, Mt. Botol, 1550 m, 17-XI-2004; 3 exx, Mt. Halimun N.P., Citalahab, Mt. Kendeng, 1550 m, 19-XI-2004; 3 exx, ARC2648 (EMBL # LM655906), ARC2649 (EMBL # LM655907), Bogor, Cidahu, Mt. Salak (near Javana Spa), sample 1, S06°43.733', E106°42.711', 1429 m, 13-IV-2012; 5 exx, same data as holotype; 1 ex, ARC2653 (EMBL # LM655911), Sukabumi, Mt. Gede, Cisaat, Situ Gunung, sample 1, S06°49.377', E106°55.729', 1281 m, 15-IV-2012; 1 ex, ARC2663 (EMBL # LM655921), Sukabumi, Mt. Gede, Cisaat, Situ Gunung, sample 5, S06°47.912', E106°56.457', 1940 m, 16-IV-2012; 1 ex, Sukabumi, Mt. Gede, Cisaat, Situ Gunung, sample 4, S06°48.250', E106°56.271', 1720 m, 16-IV-2012; 4 exx, ARC2666 (EMBL # LM655924), Sukabumi, Mt. Gede, Cisaat, Situ Gunung, sample 7, S06°48.377', E106°56.183', 1637 m, 16-IV-2012, under *Lithocarpus* sp.; 1 exx, ARC0159, Garut, Kawah Kamojang, sample 2, S07°09.578', E107°47.802', 1400 m, 26-IX-2005; 2 exx, ARC0182 (EMBL # LM655407), Garut, Kawah Kamojang, sample 3, S07°09.497', E107°47.828', 1430 m, 26-IX-2005; 4 exx, ARC0256 (EMBL # LM655448), ARC0257 (EMBL # LM655449), ARC0258 (EMBL # LM655450), Telaga Warna, between Puncak and Cipanas, sample 1, S06°42.253', E106°59.755', 1585 m, 06-VIII-2006; 11 exx Telaga Warna, between Puncak and Cipanas, sample 2, S06°42.097', E106°59.833', 1477 m, 06-VIII-2006.

##### Distribution.

W-Java Prov., (Mt. Gede, Mt. Halimun N.P., Kawah Kamojang, Mt. Salak). Elevation: 1400–1940 m.

##### Etymology.

This epithet is based on the Latin adjective *suturalis* and refers to the basally swollen elytral suture.

##### Notes.

*Trigonopterus suturalis* Riedel, sp. n. was coded as “*Trigonopterus* sp. 304”.

#### 90. Trigonopterus syarbis

Taxon classificationAnimaliaColeopteraCurculionidae

Riedel sp. n.

http://zoobank.org/D126AED6-7550-4C91-9CA2-01D21F05243A

##### Diagnostic description.

**Figure 90.**
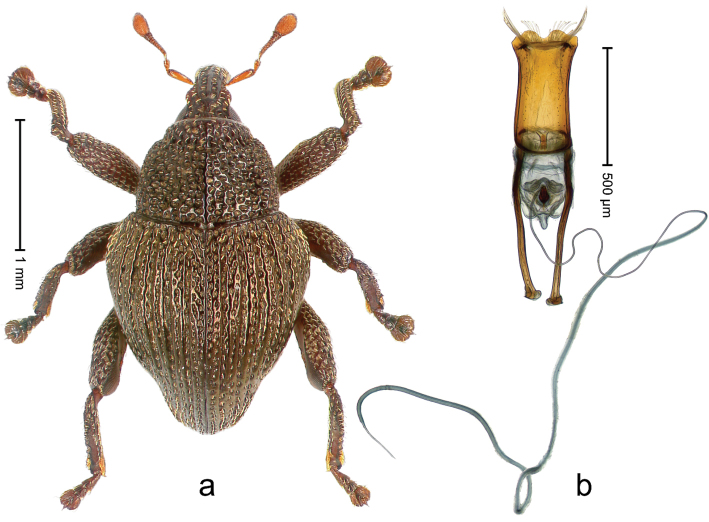
*Trigonopterus syarbis* Riedel, sp. n., holotype; **a** Habitus **b** Penis.

Holotype, male (Fig. 90a). Length 2.45 mm. Color of legs and antennae ferruginous; remainder black; elytron with reddish coppery lustre. Body in dorsal aspect with marked constriction between pronotum and elytron; in profile dorsally convex. Rostrum with median and pair of submedian ridges becoming indistinct apically; intervening furrows each with row of coarse punctures and sparse, suberect, piliform scales; epistome with indistinct, transverse ridge. Pronotum with sides weakly converging to subapical constriction; disk with pair of shallow submedian impressions, coarsely punctate, reticulate; each puncture containing inconspicuous seta; medially with indistinct ridge. Elytra with striae 1–7 deeply incised, with sparse rows of suberect, yellowish, piliform scales; intervals flat, punctate; near base and apex scales slightly more dense. Anteroventral ridge of femora distinct, in meso- and metafemur forming large tooth. Metafemur subapically with stridulatory patch. Dorsal tibial edge subbasally with angulation; mesotibia in apical 1/3 with tooth on dorsal edge. Onychium minute, drop-shaped, with median spine instead of pair of claws. Abdominal ventrite 5 flat, coarsely punctate. Penis (Fig. 90b) with sides of body subparallel, diverging to bisinuate apex; apical orifice with fringe of long setae; transfer apparatus small, compact; apodemes 1.7 × as long as body; ductus ejaculatorius without indistinct bulbus.

##### Material examined.

Holotype (MZB): ARC2195 (EMBL # LM655651), East Nusa Tenggara Prov., Flores, Ruteng, Mt. Ranaka, sample 1, S08°37.321', E120°31.463', 1535 m, 08-III-2011.

##### Distribution.

East Nusa Tenggara Prov., Flores (Ruteng). Elevation: 1535 m.

##### Etymology.

This epithet is based on the weevil genus *Syarbis* to which it bears some superficial resemblance e.g. in the reduction of the onychium. It is to be treated as a noun in apposition.

##### Notes.

*Trigonopterus syarbis* Riedel, sp. n. was coded as “*Trigonopterus* sp. 350”.

#### 91. Trigonopterus telagensis

Taxon classificationAnimaliaColeopteraCurculionidae

Riedel sp. n.

http://zoobank.org/160BF790-2480-4C82-B287-4AB20BB473F4

##### Diagnostic description.

**Figure 91.**
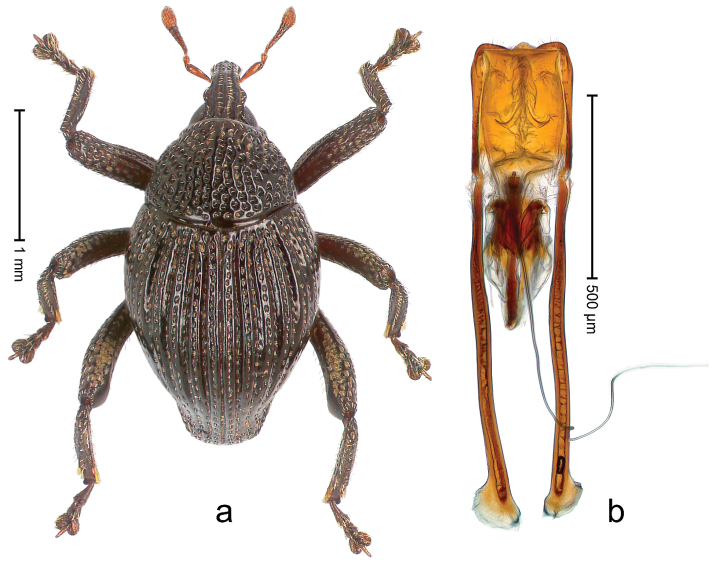
*Trigonopterus telagensis* Riedel, sp. n., holotype; **a** Habitus **b** Penis.

Holotype, male (Fig. 91a). Length 2.55 mm. Color of antennae and legs ferruginous; remainder black. Body subovate, in dorsal aspect with weak constriction between pronotum and elytron; in profile dorsally convex. Rostrum with median ridge and pair of submedian ridges, intervening furrows each with sparse row of mesad directed scales; epistome with irregular transverse ridge. Pronotum coarsely punctate, reticulate-costate, with median costa; with sparse, recumbent scales. Elytra with striae dorsally deeply incised, laterally marked by rows of coarse punctures; intervals markedly costate, dorsally flattened, with rows of punctures; sutural interval basally slightly widened laterad; each puncture containing small recumbent scale; elytral apex subtruncate. Femora with simple anteroventral ridge. Metafemur subapically with stridulatory patch. Abdominal ventrite 5 coarsely punctate, with sparse subrecumbent scales. Penis (Fig. 91b) with sides of body subparallel, with shallow constriction at middle, before apex sides slightly swollen; apex with median angulate extension; transfer apparatus with thick median rod 1.7 × as long as body; median rod projecting basad further than apicad; apodemes 2.8 × as long as body; ductus ejaculatorius without bulbus. **Intraspecific variation.** Length 2.35–2.75 mm. Color of elytra dark ferruginous to black. Female rostrum in apical half slender, dorsally subglabrous, with submedian rows of punctures, laterally punctate-rugose; epistome simple.

##### Material examined.

Holotype (MZB): ARC2312 (EMBL # LM655765), Bali, Busungbiu – Pupuan, Telaga, Primatani reserve, sample 2, S08°17.304', E114°56.635', 625 m, 02-IV-2011. Paratypes (MZB, SMNK, ZSM): Bali: 6 exx, ARC2313 (EMBL # LM655766), ARC2314 (EMBL # LM655767), ARC2956 (EMBL # LM656009), ARC2957 (EMBL # LM656010), ARC2958 (EMBL # LM656011), same data as holotype; 21 exx, ARC0723, ARC0724, ARC0725, ARC0726 (PCR failed), Busungbiu – Pupuan, S08°17.64', E114°56.72', 600–700 m, sifted.

##### Distribution.

Bali (Telaga). Elevation: 625 m.

##### Etymology.

This epithet is based on the type locality, Telaga.

##### Notes.

*Trigonopterus telagensis* Riedel, sp. n. was coded as “*Trigonopterus* sp. 289” by Tänzler et al. (2014).

#### 92. Trigonopterus tepalensis

Taxon classificationAnimaliaColeopteraCurculionidae

Riedel sp. n.

http://zoobank.org/FE88CB46-FFD3-4E18-99AF-EDA462185900

##### Diagnostic description.

**Figure 92.**
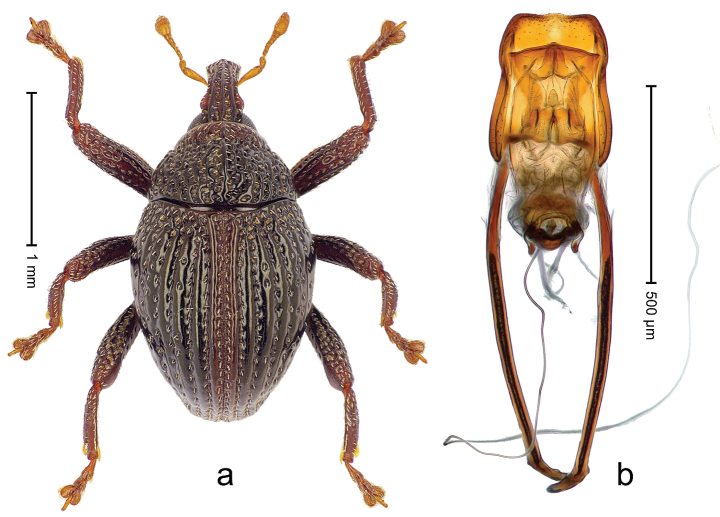
*Trigonopterus tepalensis* Riedel, sp. n., holotype; **a** Habitus **b** Penis.

Holotype, male (Fig. 92a). Length 1.98 mm. Color of antennae and legs ferruginous, sutural interval dark ferruginous; remainder black. Body subovate, in dorsal aspect and in profile with weak constriction between pronotum and elytron. Rostrum with median ridge and pair of submedian ridges, intervening furrows each with sparse row of mesad directed setae; epistome simple. Pronotum coarsely punctate, submedially interspaces longitudinally rugose, with median costa; with sparse, suberect, slender scales. Elytra with striae deeply impressed; each with row of slender suberect setae; intervals costate, subglabrous, some with scattered punctures; with dense row of punctures on sutural interval, facing mesad towards incised suture. Femora with simple, crenate anteroventral ridge. Metafemur subapically with stridulatory patch. Metatibia apically with uncus, without premucro. Abdominal ventrite 5 coarsely punctate, with sparse suberect setae; with median carina. Penis (Fig. 92b) with sides of body converging, at middle with shallow constriction; containing pairs of sclerites; apex sparsely setose, with median extension indistinct, rounded; transfer apparatus symmetrical, with two pairs of tendons attached; apodemes 2.5 × as long as body; ductus ejaculatorius without bulbus.

##### Material examined.

Holotype (MZB): ARC3588 (EMBL # LM656020), West Nusa Tenggara Prov., W-Sumbawa, Tepal, Pc. Nengas, sample 5, S08°35.740', E117°08.721', 1330 m, 16-IV-2010.

##### Distribution.

West Nusa Tenggara Prov., Sumbawa (Tepal). Elevation: 1330 m.

##### Etymology.

This epithet is based on the type locality.

##### Notes.

*Trigonopterus tepalensis* Riedel, sp. n. was coded as “*Trigonopterus* sp. 439”.

#### 93. Trigonopterus tiga

Taxon classificationAnimaliaColeopteraCurculionidae

Riedel sp. n.

http://zoobank.org/99179946-5A7F-4785-A26D-F1B11E2C72BB

##### Diagnostic description.

**Figure 93.**
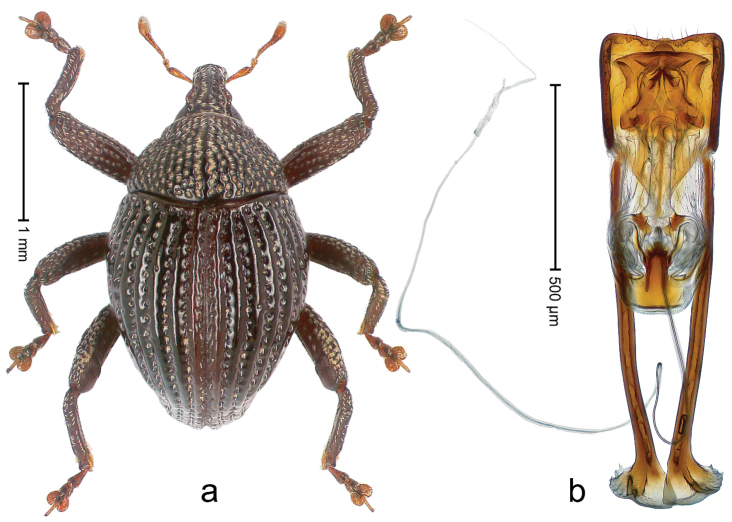
*Trigonopterus tiga* Riedel, sp. n., holotype; **a** Habitus **b** Penis.

Holotype, male (Fig. 93a). Length 2.34 mm. Color of antennae light ferruginous, legs and sutural interval dark ferruginous; remainder black. Body subovate, in dorsal aspect with weak constriction between pronotum and elytron; with distinct constriction in profile. Rostrum with median ridge and pair of irregular submedian ridges, intervening furrows each with sparse row of mesad directed setae; epistome with transverse ridge. Pronotum coarsely punctate, laterally reticulate, submedially interspaces longitudinally rugose, with median costa; with sparse, slender, subrecumbent scales. Elytra with striae deeply impressed, with row of coarse punctures, each puncture containing one suberect bristle; intervals costate, subglabrous; suture incised, dull microreticulate, bordered by row of punctures. Femora with simple, crenate anteroventral ridge. Metafemur subapically with stridulatory patch. Abdominal ventrite 5 dull, coriaceous, at middle with shallow impression, with distinct median carina. Penis (Fig. 93b) with sides of body slightly diverging in straight line; apex subangulate, subglabrous, sparsely setose; orifice with complex sclerites; transfer apparatus compact, symmetrical, apically with large anchor-shaped sclerite; apodemes 3.3 × as long as body; ductus ejaculatorius without bulbus. **Intraspecific variation.** Length 1.98–2.34 mm. Female rostrum in apical half dorsally subglabrous, punctate; epistome simple. Female abdominal ventrite flat, with weak median ridge.

##### Material examined.

Holotype (MZB): ARC2243 (EMBL # LM655698), East Nusa Tenggara Prov., Flores Isl., Labuhan Bajo, Tebedo, sample 1, S08°29.848', E119°59.633', 525 m, 14-III-2011. Paratypes (SMNK): Flores Isl., Labuhan Bajo: 1 ex, ARC2225 (EMBL # LM655681), Roe, sample 5, S08°35.395', E120°00.383', 790 m, 13-III-2011.

##### Distribution.

East Nusa Tenggara Prov., Flores (Labuhan Bajo). Elevation: 525–790 m.

##### Etymology.

This epithet is based on the Indonesian word for “three” and is treated as a noun in apposition.

##### Notes.

*Trigonopterus tiga* Riedel, sp. n. was coded as “*Trigonopterus* sp. 291” by Tänzler et al. (2014).

#### 94. Trigonopterus trigonopterus

Taxon classificationAnimaliaColeopteraCurculionidae

Riedel sp. n.

http://zoobank.org/A5C12008-E81C-4EE7-BEE4-30D38D0F76E6

##### Diagnostic description.

**Figure 94.**
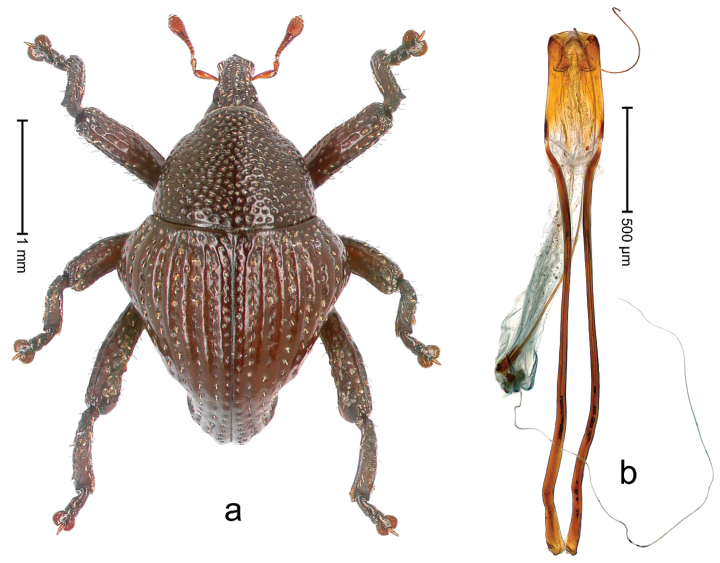
*Trigonopterus trigonopterus* Riedel, sp. n., holotype; **a** Habitus **b** Penis.

Holotype, male (Fig. 94a). Length 3.00 mm. Color of antennae light ferruginous, pronotum black, remainder dark ferruginous. Body in dorsal aspect subrhomboid, with marked constriction between pronotum and elytron; profile with blunt angulation between pronotum and elytron. Rostrum with median and pair of submedian ridges; with sparse suberect scales; epistome with transverse, subangulate ridge. Pronotum with indistinct subapical constriction; disk densely punctate, reticulate; each puncture containing small recumbent seta. Elytra with humeri markedly swollen, laterally subangularly projecting, with coarse punctures; striae distinct, deeply impressed, each with sparse row of suberect scales; intervals costate, weakly microreticulate; sutural intervals with row of smaller punctures, swollen apically; interval 8 apically forming short ridge. Metafemur subapically with stridulatory patch. Dorsal edge of tibiae subbasally dentate. Abdominal ventrites 1–2 forming common cavity, at middle flat, subglabrous, with sparse erect scales; laterally with distinct rim; abdominal ventrite 2 in profile projecting dentiform; abdominal ventrite 5 flat, coarsely punctate, with sparse erect scales. Penis (Fig. 94b) with sides of body subparallel, containing bell-shaped sclerite; apex rounded; transfer apparatus flagelliform, ca. 4 × as long as body; apodemes 3.4 × as long as body; ductus ejaculatorius without bulbus. **Intraspecific variation.** Length 2.26–3.00 mm. Color of body light or dark ferruginous. Female rostrum dorsally medially subglabrous, sublaterally punctate-rugose; epistome simple. Female elytra with humeri less prominent, convex.

##### Material examined.

Holotype (MZB): ARC2531 (EMBL # LM655868), W-Kalimantan Prov., Bengkayan, Suka-Bangun, Mt. Bawang, sample 2, N00°53.514', E109°22.301', 275 m, 10-XII-2011. Paratypes (MZB, SMNK, ZSM): W-Kalimantan Prov., Bengkayan, Suka-Bangun, Mt. Bawang: 7 exx, ARC2530 (EMBL # LM655867), ARC2532 (EMBL # LM655869), ARC2533 (EMBL # LM655870), same data as holotype; 3 exx, sample 3, N00°53.621', E109°22.475', 400 m, 10-XII-2011; 1 ex, sample 6, N00°53.992', E109°22.502', 556 m, 11-XII-2011; 6 exx, ARC2544 (EMBL # LM655881), sample 7, N00°53.921', E109°22.444', 515 m, 11-XII-2011; 1 ex, ARC2545 (EMBL # LM655882), sample 8, N00°53.736', E109°22.316', 411 m, 11-XII-2011.

##### Distribution.

W-Kalimantan Prov. (Mt. Bawang). Elevation: 275–556 m.

##### Etymology.

This epithet is based on the generic name, which is describing the triangular shape of the elytra very well. It is to be treated as a noun in apposition.

##### Notes.

*Trigonopterus trigonopterus* Riedel, sp. n. was coded as “*Trigonopterus* sp. 363” by Tänzler et al. (2014).

#### 95. Trigonopterus tujuh

Taxon classificationAnimaliaColeopteraCurculionidae

Riedel sp. n.

http://zoobank.org/2185E8B2-43AF-4265-8B4C-38A004CF3E1D

##### Diagnostic description.

**Figure 95.**
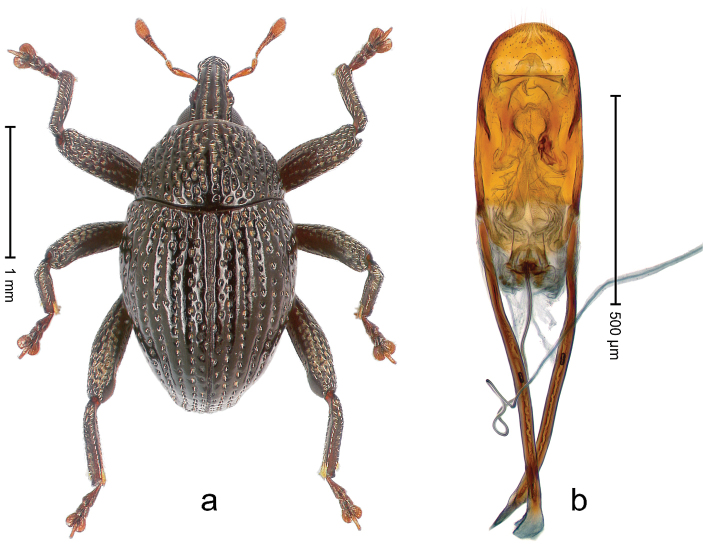
*Trigonopterus tujuh* Riedel, sp. n., holotype; **a** Habitus **b** Penis.

**Figure 96.**
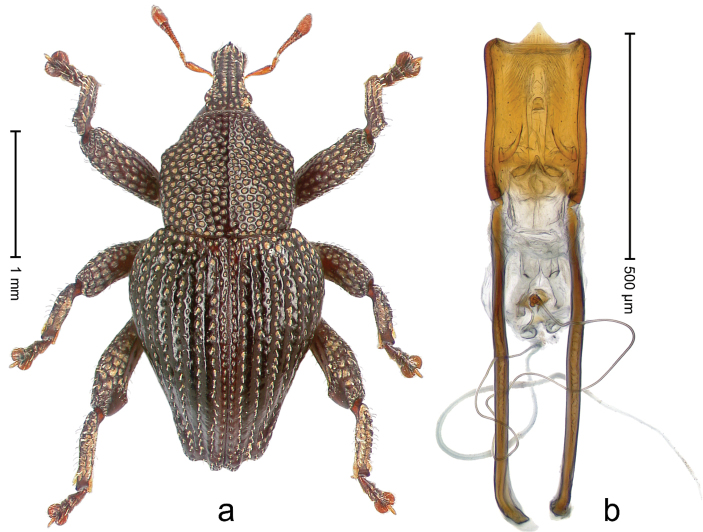
*Trigonopterus ujungkulonensis* Riedel, sp. n., holotype; **a** Habitus **b** Penis.

Holotype, male (Fig. 95a). Length 2.27 mm. Color of antennae and legs ferruginous; remainder black. Body subovate, in dorsal aspect and in profile with weak constriction between pronotum and elytron. Rostrum with median ridge and pair of indistinct submedian ridges, intervening furrows each with sparse row of mesad directed setae; anteriorly scabrous; epistome with transverse ridge. Pronotum coarsely punctate, interspaces longitudinally rugose, with median costa; with sparse, subrecumbent scales. Elytra with striae deeply impressed; each puncture containing small suberect seta; subbasally with sparse, slender, suberect scales; intervals weakly costate, almost flat, subglabrous, sutural interval with sparse coarse punctures; in basal half incised suture bordered by row of punctures, partly overgrown by sutural interval. Femora with simple, crenate anteroventral ridge. Metafemur subapically with stridulatory patch. Metatibia apically with uncus, without premucro. Abdominal ventrite 5 coarsely punctate, except apical third subglabrous. Penis (Fig. 96b) with sides of body subparallel, weakly converging; apex rounded, sparsely setose; transfer apparatus small, compact, symmetrical; apodemes 2.0 × as long as body; ductus ejaculatorius without bulbus. **Intraspecific variation.** Length 2.27–2.36 mm. Female unknown.

##### Material examined.

Holotype (MZB): ARC2231 (EMBL # LM655687), East Nusa Tenggara Prov., Flores Isl., Labuhan Bajo, Roe, sample 3, S08°36.259', E120°01.539', 975 m, 13-III-2011 (MZB). Paratypes (MZB, SMNK): 4 exx, ARC2590 (EMBL # LM655899), same data as holotype.

##### Distribution.

East Nusa Tenggara Prov., Flores (Labuhan Bajo). Elevation: 975 m.

##### Etymology.

This epithet is based on the Indonesian word for “seven” and is treated as a noun in apposition.

##### Notes.

*Trigonopterus tujuh* Riedel, sp. n. was coded as “*Trigonopterus* sp. 325” by Tänzler et al. (2014).

#### 96. Trigonopterus ujungkulonensis

Taxon classificationAnimaliaColeopteraCurculionidae

Riedel sp. n.

http://zoobank.org/3C7F602D-DEEA-4CC6-AA5B-B854D33BD761

##### Diagnostic description.

Holotype, male (Fig. 96a). Length 2.88 mm. Color of antennae light ferruginous, legs and head dark ferruginous, remainder black. Body elongate; in dorsal aspect with marked constriction between pronotum and elytron; in profile dorsally without constriction. Rostrum dorsally microreticulate; in basal half with median carina terminating on forehead; with pair of submedian ridges; intervening furrows each with row of punctures and erect scales; epistome with transverse, angulate ridge forming distinct median denticle. Pronotum anterolaterally subangularly projecting; without distinct subapical constriction; disk coarsely punctate-reticulate; each puncture containing small recumbent scale; with median glabrous wrinkle. Elytra with striae deeply impressed; each with row of narrow subrecumbent scales; intervals weakly costate, basally with row of small punctures; basally punctation confused; interval 7 swollen subapically, laterally weakly projecting; sutural interval swollen at apex, protruding. Femora edentate. Metafemur subapically with stridulatory patch and transverse row of denticles. Abdominal ventrite 5 with broadly impressed, shallow pit. Penis (Fig. 96b); sides of body subparallel; apex with median, acute triangular extension; transfer apparatus small; apodemes 2.5 × as long as body; ductus ejaculatorius without bulbus. **Intraspecific variation.** Length 2.58–3.05 mm. Color of body black, dark ferruginous, or light ferruginous. Female rostrum dorsally with median and pair of submedian costae glabrous; intervening furrows each with row of punctures bearing small scales or scales partly abraded; epistome simple. Elytra with scales long, suberect, or short, subrecumbent. Female abdominal ventrite 5 flat.

##### Material examined.

Holotype (MZB): ARC1540 (EMBL # LM655638), Java, Banten Prov., Ujung Kulon N.P., Tama Jaya, Mt. Honje, sample 5, S06°46.241', E105°31.542', 260 m, 24-IV-2010 (MZB). Paratypes (MZB, SMNK, ZSM): Java, Banten Prov., Ujung Kulon N.P., Tama Jaya, Mt. Honje: 35 exx, ARC1541 (EMBL # LM655639), same data as holotype; 23 exx, ARC1545 (EMBL # LM655643), ARC1546 (EMBL # LM655644), sample 4, S06°46.141', E105°31.649', 395 m, 23-IV-2010; 55 exx, sample 4, S06°46.141', E105°31.649', 395 m, 24-IV-2010; 24 exx, sample 6, S06°46.206', E105°31.592', 305 m, 24-IV-2010.

##### Distribution.

Banten Prov. (Ujung Kulon N.P.). Elevation: 260–395 m.

##### Etymology.

This epithet is based on the type locality.

##### Notes.

*Trigonopterus ujungkulonensis* Riedel, sp. n. was coded as “*Trigonopterus* sp. 318”.

#### 97. Trigonopterus variolosus

Taxon classificationAnimaliaColeopteraCurculionidae

Riedel sp. n.

http://zoobank.org/E9F553A1-34D2-4FCB-A9AC-3E37DA83D62F

##### Diagnostic description.

**Figure 97.**
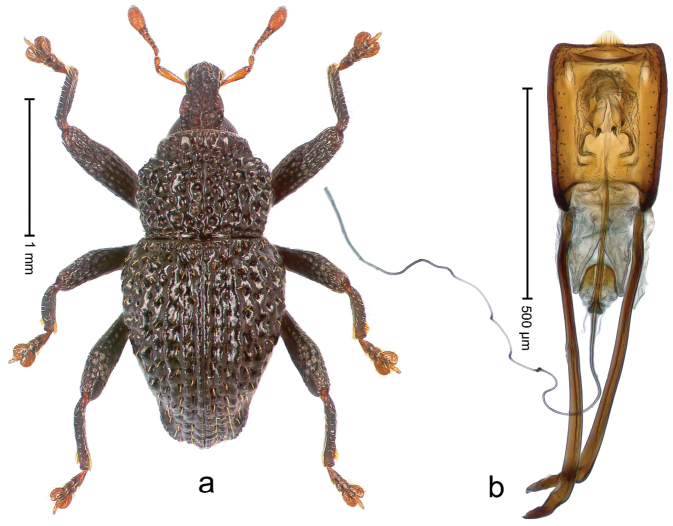
*Trigonopterus variolosus* Riedel, sp. n., holotype; **a** Habitus **b** Penis.

Holotype, male (Fig. 97a). Length 2.35 mm. Color black, tarsi and antennae ferruginous. Body elongate; in dorsal aspect with marked constriction between pronotum and elytron; with distinct constriction in profile. Rostrum coarsely rugose-punctate, with median ridge and pair of irregular submedian ridges; epistome with indistinct, transverse, subangulate ridge. Pronotum with distinct subapical constriction; disk scabrous, punctures each with one erect seta; with median ridge. Elytra with striae deeply impressed, each with row of stout suberect bristles; intervals costate-tuberculate, almost nude; interval 3 most prominent; sutural interval swollen apically; interval 7 swollen subapically, laterally weakly projecting. Femora edentate. Metafemur subapically with stridulatory patch. Abdominal ventrite 5 flat, densely punctate, setose. Penis (Fig. 97b) with body flattened, in profile markedly curved; in dorsal aspect sides weakly diverging apicad; apex with median, triangular extension; transfer apparatus flagelliform, 1.1 × longer than body; apodemes 2.6 × as long as body; ductus ejaculatorius without bulbus. **Intraspecific variation.** Length 2.13–2.50 mm. Female rostrum dorsally with median costa and pair of submedian costae separated by rows of coarse punctures; epistome simple. Elytra with sutural interval apically more or less swollen.

##### Material examined.

Holotype (MZB): ARC0314 (EMBL # LM655504), West Java Prov., SW Bandung, Ciwidey, Mt. Patuha, sample 2, S07°09.699', E107°24.377', 2100 m, 06-IX-2006. Paratypes (MZB, ARC, SMNK, ZSM): W-Java Prov.: 2 exx, ARC0312 (EMBL # LM655502), ARC0313 (EMBL # LM655503), same data as holotype; 5 exx, ARC0315 (EMBL # LM655505), ARC0316 (EMBL # LM655506), SW Bandung, Ciwidey, Mt. Patuha, sample 3, S07°09.336', E107°24.260', 2015 m, 06-IX-2006; 6 exx, ARC0004, Ciwidey, Gambung, Mt. Tilu, 1900 m, 12-VI-2002; 13 exx, ARC0169, ARC0214 (EMBL # LM655436), ARC0215 (EMBL # LM655437), Cilawu, Mt. Cikuray, sample 3, S07°19.075', E107°52.338', 2050 m, 24-IX-2005.

##### Distribution.

W-Java Prov., (Mt. Cikuray, Mt. Patuha, Mt. Tilu). Elevation: 1900–2100 m.

##### Etymology.

This epithet is based on the Latin adjective *variolosus* (scarred) and refers to the species´ rough sculpture.

##### Notes.

*Trigonopterus variolosus* Riedel, sp. n. was coded as “*Trigonopterus* sp. 302”.

#### 98. Trigonopterus vulcanicus

Taxon classificationAnimaliaColeopteraCurculionidae

Riedel sp. n.

http://zoobank.org/079AC42E-BEF1-4453-B37B-FFBC08F39D5B

##### Diagnostic description.

**Figure 98.**
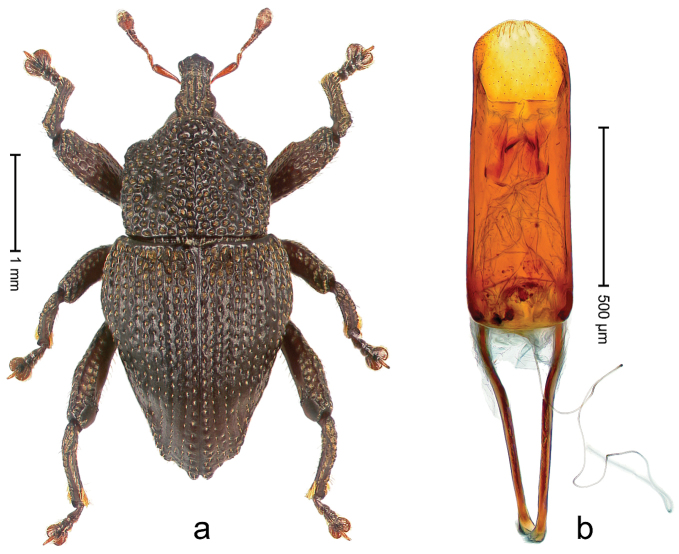
*Trigonopterus vulcanicus* Riedel, sp. n., holotype; **a** Habitus **b** Penis.

Holotype, male (Fig. 98a). Length 3.76 mm. Color of legs and antennae ferruginous, remainder black. Body elongate; in dorsal aspect with marked constriction between pronotum and elytron; in profile dorsally evenly convex. Rostrum with median carina terminating on forehead and pair of submedian ridges; intervening furrows each with row of erect piliform scales; epistome with transverse, angulate ridge forming indistinct median denticle. Pronotum anterolaterally markedly projecting, rounded; with subapical constriction; disk coarsely punctate, partly scabrous, interspaces microreticulate; each puncture containing small seta; disk with pair of submedian, shallow impressions. Elytra with striae indistinct; intervals flat; punctation dense, confused, near basal margin punctures partly confluent; each puncture containing small recumbent seta; interspaces dull, coriaceous, microreticulate; interval 7 swollen subapically, laterally weakly projecting; sutural interval at apex forming median, apically bifid protrusion. Anteroventral ridge of femora forming blunt tooth. Metafemur subapically with stridulatory patch and transverse row of denticles. Abdominal ventrite 5 flat, coarsely punctate, sparsely setose with long erect setae. Penis (Fig. 98b) with body in profile markedly curved ventrad; sides of body subparallel; apex broadly rounded; transfer apparatus minute; apodemes slightly shorter than body; ductus ejaculatorius without bulbus. **Intraspecific variation.** Length 2.68–3.88 mm. Integument of males dull, microreticulate; females with punctures sparser and smaller, interspaces polished. Female rostrum dorsally with glabrous median costa and pair of submedian costae separated by punctate furrow; epistome simple. Female elytra with lateral contour from humeri more evenly convex to apex; suture extended into acute process. Penis with body relatively short and robust in Western population; rather slender, long, basally markedly curved in Central population; intermediate between these two in Eastern population.

##### Material examined.

Holotype (MZB): ARC2514 (EMBL # LM655851), West Java Prov., Bandung, Lembang, Pangli, Mt. Bukittinggul, sample 1, S06°48.810', E107°44.216', 1753 m, 03-XII-2011. Paratypes (MZB, SMNK, ZSM): 2 exx, ARC0254 (EMBL # LM655446), ARC0255 (EMBL # LM655447), W-Java Prov., Telaga Warna, between Puncak and Cipanas, S06°42.253', E106°59.755', sample 1, 1584 m, 06-VIII-2006; 1 ex, ARC2662 (EMBL # LM655920), W-Java Prov., Sukabumi, Mt. Gede, Cisaat, Situ Gunung, sample 3, S06°48.628', E106°56.077', 1462 m, 16-IV-2012; 1 ex, ARC2667 (EMBL # LM655925), W-Java Prov., Sukabumi, Mt. Gede, Cisaat, Situ Gunung, sample 7, under *Lithocarpus* sp., S06°48.377', E106°56.183', 1637 m, 16-IV-2012; 3 exx, ARC2515 (EMBL # LM655852), same data as holotype; 4 exx, ARC2518 (EMBL # LM655855), ARC2519 (EMBL # LM655856), W-Java Prov., Bandung, Lembang, Pangli, Mt. Bukittinggul, sample 2, S06°48.845', E107°44.074', 1898 m, 03-XII-2011; 7 exx, ARC2706 (EMBL # LM655962), ARC2707 (EMBL # LM655963), ARC2708 (EMBL # LM655964), W-Java Prov., Garut, Cikajang, Mt. Payung, sample 2, S07°25.268', E107°48.492', 1250 m, 26-IV-2012; 6 exx, ARC2712 (EMBL # LM655968), W-Java Prov., Garut, Cikajang, Mt. Payung, sample 3, S07°25.320', E107°48.259', 1560 m, 26-IV-2012; 3 exx, ARC0205 (EMBL # LM655427), ARC0206 (EMBL # LM655428), ARC0207 (EMBL # LM655429), W-Java Prov., Garut, Cilawu, Mt. Cikuray, sample 1, S07°18.578', E107°52.622', 1648 m, 24-IX-2005; 3 exx, ARC0161, ARC0187 (EMBL # LM655412), W-Java Prov., Garut, Cilawu, Mt. Cikuray, sample 2, S07°18.840', E107°52.512', 1800 m, 24-IX-2005; 7 exx, ARC2720 (EMBL # LM655976), ARC2721 (EMBL # LM655977), ARC2722 (EMBL # LM655978), W-Java Prov., Garut, Wanaraja, Talagabodas, sample 2, S07°11.966', E108°03.984', 1719 m, 27-IV-2012; 1 ex, ARC2723 (EMBL # LM655979), W-Java Prov., Garut, Wanaraja, Talagabodas, sample 1, S07°12.511', E108°03.554', 1741 m, 27-IV-2012; 6 exx, ARC0162, ARC0173, ARC0199 (EMBL # LM655421), ARC0200 (EMBL # LM655422), W-Java Prov., Ciamis, Mt. Sawal, Batu Cakra, S07°14.920', E108°15.762', 990 m, 01-X-2005; 1 ex, W-Java Prov., Ciamis, Mt. Sawal, Blok Cireong, sample 2, S07°14.127', E108°15.568', 1120 m, 01-X-2005; 9 exx, ARC0293 (EMBL # LM655483), ARC0294 (EMBL # LM655484), ARC0295 (EMBL # LM655485), C-Java Prov., N slopes of Dieng figau, Petungkriyono, mountain N Tinalum, sample 1, S07°06.418', E109°44.514', 1115 m, 22-VIII-2006; 6 exx, ARC0298 (EMBL # LM655488), ARC0299 (EMBL # LM655489), ARC0300 (EMBL # LM655490), C-Java Prov., N slopes of Dieng figau, Petungkriyono, Mt. Deles, sample 3, S07°08.225', E109°43.555', 1495 m, 24-VIII-2006; 7 exx, C-Java Prov., N slopes of Dieng figau, Petungkriyono, Mt. Deles, sample 1, S07°08.221', E109°43.599', 1505 m, 24-VIII-2006; 2 exx, C-Java, Mt. Slamet, Guci, sample 5, S07°11.953', E109°10.497', 1620 m, 27-XI-2011; 6 exx, ARC2499 (EMBL # LM655836), ARC2500 (EMBL # LM655837), Central Java Prov., Mt. Slamet, Guci, sample 6, S07°11.983', E109°10.556', 1671 m, 27-XI-2011; 9 exx, ARC2501 (EMBL # LM655838), ARC2502 (EMBL # LM655839), C-Java Prov., Mt. Slamet, Guci, sample 4, S07°12.304', E109°10.450', 1406 m, 27-XI-2011.

##### Distribution.

W-Java Prov. (Mt. Gede N.P., Mt. Bukittinggul, Mt. Cikuray, Mt. Payung, Talagabodas, Mt. Sawal), C-Java Prov. (Dieng, Mt. Slamet). Elevation: 990–1898 m.

##### Etymology.

This epithet is based on the Latin adjective *vulcanicus* and refers to its presence on volcanic mountains.

##### Notes.

*Trigonopterus vulcanicus* Riedel, sp. n. was coded as “*Trigonopterus* sp. 313”. This species is divided into three populations that exhibit slightly different shapes of the penis; however, they hardly justify recognition as separate species. The western population is found at Mt. Gede; the central population at Mt. Bukittinggul, Mt. Cikuray, Mt. Payung and Talagabodas; and the Eastern population at Mt. Sawal, Dieng, and Mt. Slamet.

#### 99. Trigonopterus wallacei

Taxon classificationAnimaliaColeopteraCurculionidae

Riedel sp. n.

http://zoobank.org/7504E20B-BE06-4E9C-9E10-45C3FFF57E47

##### Diagnostic description.

**Figure 99.**
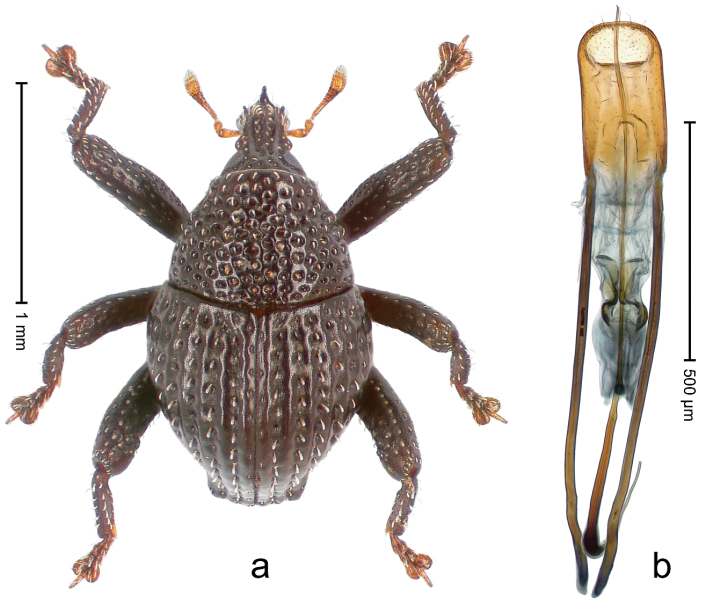
*Trigonopterus wallacei* Riedel, sp. n., holotype; **a** Habitus **b** Penis.

Holotype, male (Fig. 99a). Length 1.52 mm. Color of antennae, tarsi and tibiae ferruginous; remainder black. Body subovate, in dorsal aspect and in profile with weak constriction between pronotum and elytron. Rostrum basally with median ridge and pair of submedian ridges terminating near middle with marked protrusion, with rows of erect clavate scales; protrusion anteriorly steeply declivous, hollowed; between protuberance and epistome relatively flat, subglabrous, microreticulate; epistome at middle with dorsoposteriad directed horn, laterally with pair of denticles. Pronotum coarsely punctate-reticulate, each puncture with one clavate suberect scale. Elytra with striae deeply impressed; punctures large, each bearing one suberect scale; intervals narrow, weakly costate, subglabrous, microreticulate; apex subtruncate. Metafemur subapically with stridulatory patch. Abdominal ventrite 5 flat, coarsely punctate. Penis (Fig. 99b) with sides of body subparallel; apex rounded; transfer apparatus flagelliform; apodemes 2.9 × as long as body; ductus ejaculatorius sclerotized, swollen, apically torn off. **Intraspecific variation.** Length 1.52–1.79 mm. Female rostrum dorsally even, without teeth or cavities, punctate-rugose.

##### Material examined.

Holotype (SMNK): ARC2525 (EMBL # LM655862), MALAYSIA, Sarawak, Kuching, Mt. Santubong, sample 3, N01°43.884', E110°19.727', 496 m, 06-XII-2011. Paratypes (ARC, SMNK): MALAYSIA, Sarawak, Kuching: 2 exx, ARC2526 (EMBL # LM655863), same data as holotype; 1 ex, ARC2529 (EMBL # LM655866), Mt. Santubong, sample 4, N01°43.884', E110°19.715', 487 m, 06-XII-2011; 2 exx, sample 5, N01°43.843', E110°19.672', 478 m, 06-XII-2011; 2 exx, sample 6, N01°43.830', E110°19.601', 375 m, 06-XII-2011; 1 ex, Mt. Santubong, 700 m, 25-III-1990; 2 exx, Bako N.P., 27-III-1990.

##### Distribution.

Sarawak (Santubong). Elevation: 487–700 m.

##### Etymology.

This species is named in honour of Alfred Russel Wallace (1823–1913) for laying the foundation of research on the Natural History of the Indo-Australian Archipelago. He spent some time at this species´ type locality where he prepared the draft of his “Sarawak Law”.

##### Notes.

*Trigonopterus wallacei* Riedel, sp. n. was coded as “*Trigonopterus* sp. 362” by Tänzler et al. (2014).

### Key to species from Java and Sumatra

**Table:** 

1	Metafemur subapically simple, without stridulatory patch. Length 1.90–2.65 mm. (subgenus *Mimidotasia*)	**2**
1'	Metafemur subapically with stridulatory patch. Length 2.35–3.80 mm	**9**
2 (1)	Elytra (Figs 18a, 51a) elongate, 1.36–1.46 × longer than wide	**3**
2'	Elytra (e.g. Figs 24a, 29a, 38a) compact, 1.15–1.35 × longer than wide	**4**
3 (2)	Sutural interval of elytra swollen subapically, projecting in dorsal aspect. Penis (Fig. 18b), with long asymmetrical apical extension slightly upcurved and weakly directed to the left; E-Java	***Trigonopterus costipennis* Riedel, sp. n.**
3'	Elytral apex with sutural interval costate but not distinctly projecting in dorsal aspect. Penis (Fig. 51b), with long asymmetrical apical extension markedly directed to the left; E-Sumatra	***Trigonopterus misellus* Riedel, sp. n.**
4 (2')	Elytral striae with rows of elongate-claviform, rather slender scales	**5**
4'	Elytral striae with rows of claviform, wider scales	**6**
5 (4)	Elytral intervals costate-tuberculate. Penis (Fig. 24b) with medium-sized asymmetrical apical extension to the left	***Trigonopterus diengensis* Riedel, sp. n.**
5'	Elytral intervals costate. Penis (Fig. 29b) with long asymmetrical apical extension directed to the right	***Trigonopterus echinatus* Riedel, sp. n.**
6 (4')	Elytral intervals with relatively low costae. Penis (Figs 38b, 78b) with apical extension short, subangulate	**7**
6'	Elytral intervals with costae more distinct. Penis (Figs 34b, 73b) with apical extension acute, directed to the left	**8**
7 (6)	Color largely ferruginous. Penis (Fig. 38b). W-Java	***Trigonopterus honjensi* s Riedel, sp. n.**
7'	Color largely black. Penis (Fig. 78b). E-Sumatra	***Trigonopterus seriatus* Riedel, sp. n.**
8 (6')	Penis with endophallic structures as in Fig. 73b. E-Java	***Trigonopterus satu* Riedel, sp. n.**
8'	Penis with endophallic structures as in Fig. 34b. W-Java	***Trigonopterus foveatus* Riedel, sp. n.**
9 (1')	Elytra with striae deeply impressed; intervals costate to carinate. Femora each with small tooth. Penis (Fig. 64b) subapically forming blade-like processes. E-Java	***Trigonopterus relictus* Riedel, sp. n.**
9'	If elytral intervals costate, at least profemur edentate. Penis different, without pair of subapical blade-like processes	**10**
10	Anteroventral ridge of femora forming blunt tooth, especially in meso- and metafemur	**11**
10'	All femora without tooth, or if species from Sumatra, meso- and metafemur with small denticle	**16**
11 (10)	Sutural interval of elytra at apex markedly swollen, forming pair of distinct apical protrusions. Species from W-Java	**12**
11'	Sutural interval of elytra at apex simple or weakly swollen. Species from E-Java	**14**
12 (11)	Sutural interval of elytra at apex forming median, apically bifid protrusion. Penis (Fig. 98b) with transfer apparatus minute	***Trigonopterus vulcanicus* Riedel, sp. n.**
12'	Sutural interval of elytra at apex markedly swollen, forming pair of rounded apical protrusions. Penis (Figs 15b, 25b) basally with distinct transfer apparatus	**13**
13 (12')	Transfer apparatus of penis (Fig. 25b) compact, apically blunt	***Trigonopterus dimorphus* Riedel, sp. n.**
13'	Transfer apparatus of penis (Fig. 15b) apically with spine	***Trigonopterus binodulus* Riedel, sp. n.**
14 (11')	Disk of pronotum (Fig. 48a) densely, uniformly punctate. Protibia subbasally with distinct dorsoposterior tooth. Apex of penis (Fig. 48b) bidentate, with median notch	***Trigonopterus merubetirensis* Riedel, sp. n.**
14'	Punctation of pronotum more coarsely, forming more or less distinct pattern (Figs 8a, 39a). Protibia subbasally with simple angulation, at most with minute denticle. Apex of penis (Figs 8b, 39b) simple or with small median extension	**15**
15 (14')	Disk of pronotum (Fig. 39a) with punctures partly elongate and / or arranged in confluent rows forming rhombus-like pattern. Transfer apparatus of penis in profile ventrally with subangular extension	***Trigonopterus ijensis* Riedel, sp. n.**
15'	Disk of pronotum (Fig. 8a) with punctures less confluent; rhombus-like pattern less distinct. Transfer apparatus of penis in profile with ventral contour evenly convex	***Trigonopterus argopurensis* Riedel, sp. n.**
16 (10')	Elytral intervals each with secondary rows of punctures resembling striae; at middle with transverse subglabrous band	***Trigonopterus alaspurwensis* Riedel, sp. n.**
16'	Elytra with sculpture different; if sculpture coarse, never with transverse subglabrous band at middle	**17**
17 (16')	Elytra with striae indistinct, marked by fine lines and rows of small punctures; intervals flat, more or less coriaceous	**18**
17'	Elytra with striae deeply impressed and / or intervals elevated	**20**
18 (17)	Tarsomere 3 of protarsus 2.8–3.0 × as wide as tarsomere 1, markedly larger than of mesotarsus, (Fig. 45a)	***Trigonopterus latipes* Riedel, sp. n.**
18'	Tarsomere 3 of protarsus 2.4–2.7 × as wide as tarsomere 1, at most slightly larger than of mesotarsus (Figs 1a, 9a)	**19**
19 (18')	Body (Fig. 9a) slender; elytra 1.25–1.34 × longer than wide; sculpture more distinct. Transfer apparatus of penis (Fig. 9b) flagelliform, 4.0 × longer than body; apodemes ca. 3 × as long as body	***Trigonopterus arjunensis* Riedel, sp. n.**
19'	Body (Fig. 1a) across humeri wider; elytra 1.11–1.23 × longer than wide; sculpture rather subglabrous. Transfer apparatus of penis (Fig. 1b) thick flagelliform, 2.0 × longer than body; apodemes ca. 2 × as long as body	***Trigonopterus acuminatus* Riedel, sp. n.**
20 (17')	Pronotum laterally without distinct preapical constriction. Usually larger species, length of elytra+pronotum 2.05–3.44 mm	**21**
20'	Pronotum laterally with distinct preapical constriction. Usually smaller species, length of elytra+pronotum 1.76–3.22 mm	**26**
21 (20)	Pronotum in apical half slightly rounded apicad. Species from East Sumatra	**22**
21'	Pronotum anterolaterally subangularly projecting. Species from Java	**23**
22 (21)	Elytra 2.62–2.65 × as long as pronotum. Penis (Fig. 83b) with more distinct lateral constriction. Mt. Rajabasa	***Trigonopterus singularis* Riedel, sp. n.**
22'	Elytra 2.50–2.56 × as long as pronotum. Sides of penis (Fig. 7b) gently diverging towards apex. Pedada Bay and Bukit Barisan N.P	***Trigonopterus amphoralis* Marshall**
23 (21')	Elytral intervals carinate; striae with rows of long erect piliform scales	**24**
23'	Elytral intervals costate; striae with rows of shorter subrecumbent scales	**25**
24 (23)	Rostrum apically without distinct median denticle. Apex of penis (Fig. 81a) subangulate, without median extension	***Trigonopterus silvestris* Riedel, sp. n.**
24'	Rostrum apically with distinct median denticle. Apex of penis (Fig. 17b) medially with small rounded extension	***Trigonopterus cahyoi* Riedel, sp. n.**
25 (23')	Elytral apex (Fig. 53a) wider and with sutural interval only weakly swollen. Elytral intervals almost flat, without punctures	***Trigonopterus pangandaranensis* Riedel, sp. n.**
25'	Elytral apex (Fig. 96a) narrow and with swollen sutural interval. Irregular elytral intervals sparsely punctate	***Trigonopterus ujungkulonensis* Riedel, sp. n.**
26 (20')	Sides of pronotum behind preapical constriction rounded, without distinct angular projections	**27**
26'	Sides of pronotum behind preapical constriction angularly projecting	**31**
27 (26)	Sutural intervals of elytra extended into median apical tooth. Mt. Gede	***Trigonopterus gedensis* Riedel, sp. n.**
27'	Sutural intervals of elytra at most swollen, but without distinct tooth	**28**
28 (27')	Elytral intervals costate-tuberculate; striae each with sparse row of suberect bristles	**29**
28'	Elytral intervals carinate; striae each with row of suberect piliform scales or without conspicuous pubescence	**30**
29 (28)	Penis (Fig. 10b) with flagellum 1.5 × longer than body of penis	***Trigonopterus asper* Riedel, sp. n.**
29'	Penis (Fig. 97b) with flagellum 1.1 × longer than body of penis	***Trigonopterus variolosus* Riedel, sp. n.**
30 (28')	Elytral striae each with row of minute recumbent scales. E-Sumatra	***Trigonopterus lampungensis* Riedel, sp. n.**
30'	Elytral striae with rows of suberect piliform scales. W-Java	***Trigonopterus setifer* Riedel, sp. n.**
31 (26')	Elytral intervals irregularly costate-tuberculate; especially towards sides costae laterally corrugate	**32**
31'	Elytral intervals costate-carinate	**36**
32 (31)	Rostrum apically with denticle	**33**
32'	Rostrum apically without denticle	**34**
33 (32)	Metatibia ventrally with sparse row of long erect setae. Penis (Fig. 37b) with flagellum ca. 2.4 × longer than body of penis	***Trigonopterus halimunensis* Riedel, sp. n.**
33'	Metatibia ventrally simple, without row of long setae. Penis (Fig. 28b) with flagellum ca. 1.9 × longer than body of penis	***Trigonopterus duabelas* Riedel, sp. n.**
34 (32')	Elytral intervals partly flat, partly costate-tuberculate; interval 5 subbasally swollen. E-Sumatra	***Trigonopterus squalidus* Riedel, sp. n.**
34'	Elytral intervals irregularly costate-tuberculate; interval 5 subbasally simple. W-Java	**35**
35 (34')	Penis (Fig. 4b) in dorsal aspect with body slender, sides subparallel	***Trigonopterus allopatricus* Riedel, sp. n.**
35'	Penis (Fig. 97b) with body flattened, in dorsal aspect wider, lateral contours weakly diverging apicad	***Trigonopterus variolosus* Riedel, sp. n.**
36 (32')	Elytra subnude, with sparse small recumbent setae	**37**
36'	Elytral striae each with row of suberect piliform scales	**38**
37 (36)	Anterolateral angular projections of pronotum distinct. Elytra setae recumbent but distinct. Java	***Trigonopterus javensis* Riedel, sp. n.**
37'	Anterolateral angular projections of pronotum indistinct. Elytral setae minute, easily overlooked. E-Sumatra	***Trigonopterus lampungensis* Riedel, sp. n.**
38 (36')	Profemur edentate, meso- and metafemur ventrally with small denticle. E-Sumatra	***Trigonopterus sumatrensis* Riedel, sp. n.**
38'	All femora edentate. Java	**39**
39 (38')	Sutural interval of elytra basally swollen, widened, raised above level of other intervals	***Trigonopterus suturalis* Riedel, sp. n.**
39'	Sutural interval of elytra surmounted by other intervals	**40**
40 (39')	Rostrum subapically with median denticle. Abdominal ventrite 5 swollen, at middle with shallow impression	**41**
40'	Rostrum subapically simple. Abdominal ventrite 5 flat or slightly swollen in apical half	**42**
41 (40)	Profile of abdominal ventrite 2 projecting angularly. Penis (Fig. 88b) apically with rounded extension; body with ca. 4 symmetrical pairs of elongate endophallic sclerites	***Trigonopterus sundaicus* Riedel, sp. n.**
41'	Profile of abdominal ventrite 2 simple. Penis (Fig. 6b) apically with pointed extension; endophallic sclerites asymmetrical	***Trigonopterus angulicollis* Riedel, sp. n.**
42 (40')	Body (Fig. 59a) slightly more elongate; elytra 1.31–1.52 × longer than wide. Penis (Fig. 59b) asymmetrical, appearing twisted	***Trigonopterus porcatus* Riedel, sp. n.**
42'	Body (Fig. 58a, 67a) slightly more compact; elytra 1.18–1.29 × longer than wide. Penis (Figs 58, 67b) symmetrical	**43**
43 (42')	Penis (Fig. 58b) apically with short, rounded extension; flagellum 2 × longer than body of penis	***Trigonopterus payungensis* Riedel, sp. n.**
43'	Penis (Fig. 67b) apically with acute triangular extension; flagellum slightly shorter than body of penis	***Trigonopterus rugosostriatus* Riedel, sp. n.**

### Key to species from Bali

**Table:** 

1	Body in dorsal aspect with marked constriction between pronotum and elytron. Pronotum in basal half with pair of sublateral, kidney-shaped impressions (Fig. 12a)	***Trigonopterus baliensis* Riedel, sp. n.**
1'	Body subovate, in dorsal aspect with weak constriction between pronotum and elytron. Pronotum without pair of distinct sublateral impressions	**2**
2	Profile with distinct constriction between pronotum and elytron. Pronotum (Figs 55a, 68a) coarsely sculptured with irregular ridges and tubercles	***Trigonopterus rugosus* Riedel, sp. n.** and ***Trigonopterus pararugosus* Riedel, sp. n.**
2'	Profile dorsally convex. Pronotum coarsely punctate, reticulate	**3**
3 (2')	Elytral intervals (Figs 43a, 49a) weakly costate or flat	**4**
3'	Elytral intervals (Figs 13a, 42a, 91a) dorsally markedly costate	**5**
4 (3)	Median rod of transfer apparatus thick, straight, 2.0 × as long as body of penis (Fig. 43b)	***Trigonopterus klatakanensis* Riedel, sp. n.**
4'	Median rod of transfer apparatus flagelliform, forming almost full coil, ca 2.7 × as long as body of penis (Fig. 49b)	***Trigonopterus mesehensis* Riedel, sp. n.**
5 (3')	Median rod of male transfer apparatus thin, shorter (1.4 × as long as body of penis), projecting basad (Fig. 13b)	***Trigonopterus batukarensis* Riedel, sp. n.**
5'	Median rod of male transfer apparatus thick, longer (1.6–1.7 × as long as body of penis), projecting basad further than apicad (Figs 42b, 91b)	**6**
6 (5')	Body of penis (Fig. 42b) with sides subparallel, before apex weakly converging in straight line	***Trigonopterus kintamanensis* Riedel, sp. n.**
6'	Body of penis (Fig. 91b) with shallow constriction at middle, before apex sides slightly swollen	***Trigonopterus telagensis* Riedel, sp. n.**

### Key to species from Lombok, Sumbawa and Flores

**Table:** 

1	Body in dorsal aspect with marked constriction between pronotum and elytron (e.g. Figs 20a, 87a)	**2**
1'	Body subovate, in dorsal aspect with weak constriction between pronotum and elytron (e.g. Figs 2a, 27a)	**20**
2 (1)	Length 1.44–2.20 mm. Pronotum in basal half with pair of sublateral impressions. Coloration of body dark ferruginous to black	**3**
2'	Length 2.20–4.04 mm. Pronotum without pair of distinct impressions. Coloration of body partly ferruginous, coppery, bronze, or black	**11**
3 (2)	Flagelliform transfer apparatus (Fig. 47b) subequal to body of penis. Lombok	***Trigonopterus lombokensis* Riedel, sp. n.**
3'	Flagelliform transfer apparatus (Figs 33b, 54b, 56b, 57b, 60b, 61b, 66b, 87b) longer than body of penis	**4**
4 (3')	Penis (Figs 61b, 87b) subapically with sides angularly projecting, abruptly converging to slightly extended apex. Sumbawa	**5**
4'	Penis with weakly subangulate or rounded apex never extended medially	**6**
5 (4)	Flagelliform transfer apparatus (Fig. 87b) 2.9 × longer than body of penis	***Trigonopterus sumbawensis* Riedel, sp. n.**
5'	Flagelliform transfer apparatus (Fig. 61b) 2.3–2.4 × longer than body of penis	***Trigonopterus pseudosumbawensis* Riedel, sp. n.**
6 (4')	Flagelliform transfer apparatus (Fig. 66b) relatively thick, 2.6 × as long as body of penis; endophallus with sclerite. Flores	***Trigonopterus roensis* Riedel, sp. n.**
6'	Flagelliform transfer apparatus (Figs 33b, 54b, 56b, 57b, 60b) thin, 3–6 × as long as body of penis; endophallus without sclerite	**7**
7 (6')	Flagelliform transfer apparatus ca. 3.0–3.3 × longer than body of penis; apodemes 2.4–2.5 × as long as body of penis. Sumbawa	**8**
7'	Flagelliform transfer apparatus ca. 4.0–7.0 × longer than body of penis; apodemes 3.2–4.1 × as long as body of penis. Flores	**9**
8 (7)	Penis (Fig. 56b) in apical third distinctly widening and rounded to weakly subangulate apex	***Trigonopterus parasumbawensis* Riedel, sp. n.**
8'	Penis (Fig. 57b) with sides parallel before rounded apex	***Trigonopterus pauxillus* Riedel, sp. n.**
9 (7')	Penis (Fig. 54b); flagelliform transfer apparatus 6.0–6.9 × as long as body	***Trigonopterus paraflorensis* Riedel, sp. n.**
9'	Penis (Figs 33b, 60b); flagelliform transfer apparatus 3.9–4.6 × as long as body	**10**
10 (9')	Elytra more compact (Fig. 33a). Penis (Fig. 33b); flagelliform transfer apparatus 3.9–4.0 × as long as body. Medium elevations of the Mt. Ranaka / Lake Ranamese area	***Trigonopterus florensis* Riedel, sp. n.**
10'	Elytra more slender (Fig. 60a). Penis (Fig. 60b); flagelliform transfer apparatus 4.1–4.6 × as long as body. Summit area of Mt. Ranaka	***Trigonopterus pseudoflorensis* Riedel, sp. n.**
11 (2')	Profemur with large subacute tooth. Color of elytra black, usually basal third ferruginous	***Trigonopterus dentipes* Riedel, sp. n.**
11'	Profemur with blunt tooth or simple	**12**
12 (11')	Color of elytra with bright metallic lustre, coppery	**13**
12'	Color of elytra black, sometimes basally ferruginous; at most with bronze lustre, never bright metallic	**17**
13 (12)	Onychium minute, drop-shaped, with median spine instead of pair of claws; weakly projecting beyond margin of tarsomere 3	***Trigonopterus syarbis* Riedel, sp. n.**
13'	Onychium distinctly projecting beyond margin of tarsomere 3, apically with pair of claws	**14**
14 (13')	Elytra with humeri markedly swollen, laterally markedly projecting (Figs 35a, 50a)	**15**
14'	Elytra with humeri simple (Fig. 19a) or weakly swollen (Fig. 20a), not markedly projecting	**16**
15 (14)	Elytra (Fig. 35a) with interval 4 subbasally swollen, forming dorsal protrusion; sutural interval simple	***Trigonopterus fulgidus* Riedel, sp. n.**
15'	Elytra (Fig. 50a) with sutural interval in basal half distinctly swollen; interval 4 without dorsal protrusion	***Trigonopterus micans* Riedel, sp. n.**
16 (14')	Elytra (Fig. 20a) with interval 5 and 6 behind middle swollen, gently projecting from lateral outline. Penis (Fig. 20b) apically subtruncate, weakly rounded, subglabrous	***Trigonopterus cupreus* Riedel, sp. n.**
16'	Elytra (Fig. 19a) with interval 5 and 6 simple. Penis (Fig. 19b) apically rounded, with fringe of conglutinate flattened setae, interrupted by glabrous median notch	***Trigonopterus cuprescens* Riedel, sp. n.**
17 (12')	Body (Fig. 79a) subovate, slender. Meso- and metafemur anteroventrally with row of large sharp teeth	***Trigonopterus serratifemur* Riedel, sp. n.**
17'	Body (Figs 21a, 72a, 84a) wider. Meso- and metafemur anteroventrally with single tooth	**18**
18 (17')	Elytra (Fig. 84a) completely black with slight bronze lustre; interval 5 forming dorsal tubercle; interval 6 behind middle swollen, gently projecting from lateral outline	***Trigonopterus sinuatus* Riedel, sp. n.**
18'	Elytra basally ferruginous; intervals 5 and 6 simple	**19**
19 (18')	Elytral interval 5 in front of middle swollen, dorsally projecting. Penis (Fig. 21b)	***Trigonopterus dacrycarpi* Riedel, sp. n.**
19'	Elytral interval 5 simple. Penis (Fig. 72b)	***Trigonopterus sasak* Riedel, sp. n.**
20 (1')	species from Lombok	**21**
20'	species from Sumbawa	**23**
20'	species from Flores	**27**
21 (20)	Elytral intervals flat, subglabrous. Pronotum longitudinally rugose	***Trigonopterus disruptus* Riedel, sp. n.**
21'	Elytral intervals costate. Pronotum coarsely punctate	**22**
22 (21')	Elytra black except sutural interval ferruginous; intervals 3 and 5 more distinctly swollen, with row of punctures	***Trigonopterus rinjaniensis* Riedel, sp. n.**
22'	Elytra with bronze lustre; intervals 3 and 5 as other intervals, subglabrous	***Trigonopterus aeneomicans* Riedel, sp. n.**
23 (20')	Elytra with intervals flat, striae marked by regular rows of punctures	***Trigonopterus punctatoseriatus* Riedel, sp. n.**
23'	Elytra with striae deeply impressed, intervals costate	**24**
24 (23')	Elytra with bronze lustre	***Trigonopterus aeneomicans* Riedel, sp. n.**
24'	Elytra largely black, without distinct metallic lustre	**25**
25 (24')	Sutural intervals of elytra costate, with row of punctures facing laterad. Abdominal ventrite 5 medially with shallow impression	***Trigonopterus sembilan* Riedel, sp. n.**
25'	Sutural intervals of elytra forming pair of ridges, inner face with dense row of punctures. Abdominal ventrite 5 with median carina	**26**
26 (25')	Elytral interval 3 with dense row of punctures. Penis as in Fig. 70b, transfer apparatus with long median extension	***Trigonopterus saltator* Riedel, sp. n.**
26'	Elytral interval 3 subglabrous, with few punctures. Penis as in Fig. 92b, transfer apparatus without median extension	***Trigonopterus tepalensis* Riedel, sp. n.**
27 (20')	Tarsomere 3 of protarsus enlarged, medially deeply incised. Penis (Fig. 32b) in basal half with marked constriction; apex medially with distinct spine, with anterolateral subangular flanges	***Trigonopterus fissitarsis* Riedel, sp. n.**
27'	Tarsomere 3 of protarsus of similar shape as tarsomere 3 of mesotarsus. Penis without marked constriction	**28**
28 (27')	Elytral intervals weakly costate, almost flat; striae consisting of rows of deeply impressed punctures	***Trigonopterus tujuh* Riedel, sp. n.**
28'	Elytral intervals distinctly costate; striae incised	**29**
29 (28')	Abdominal ventrite 5 with median carina, at least in basal third	**30**
29'	Abdominal ventrite 5 without median carina	**32**
30 (29)	Elytral interval black; suture broadly sunk-in. Abdominal ventrite 5 with median carina in basal third	***Trigonopterus delapan* Riedel, sp. n.**
30'	Elytral interval ferruginous. Abdominal ventrite 5 with complete median carina	**31**
31 (30')	Elytral intervals subglabrous, forming regular costae. Penis (Fig. 93b) with large anchor-shaped endophallic sclerite	***Trigonopterus tiga* Riedel, sp. n.**
31'	Elytral intervals on dorsum punctate, along basal margin corrugate. Penis (Fig. 63b) with sides of body in basal half converging	***Trigonopterus ranakensis* Riedel, sp. n.**
32 (29')	Metatibia apically with uncus and small premucro	**33**
32'	Metatibia apically with uncus, without premucro	**34**
33 (32)	Body (Fig. 27a) more slender. Suberect slender scales of elytral striae shorter	***Trigonopterus dua* Riedel, sp. n.**
33'	Body (Fig. 69a) wider. Suberect slender scales of elytral striae longer	***Trigonopterus rutengensis* Riedel, sp. n.**
34 (32')	Body (Fig. 31a) more slender. Suberect slender scales of elytral striae shorter	***Trigonopterus enam* Riedel, sp. n.**
34'	Body wider (Figs 30a, 46a). Suberect slender scales of elytral striae longer	**35**
35 (34')	Penis (Fig. 46b) with transfer apparatus small, with median spine projecting to apex of endophallus	***Trigonopterus lima* Riedel, sp. n.**
35'	Penis (Fig. 30b) with transfer apparatus compact, without median spine	***Trigonopterus empat* Riedel, sp. n.**

### Key to species from Borneo and Palawan

**Table:** 

1	Body subovate. Elytra with striae each bearing distinct row of suberect scales, intervals costate	**2**
1'	Body subovate or subrhomboid. Elytra with striae simple, punctures containing small scale or seta; intervals flat. IF elytra with sparse rows of suberect scales and costate intervals, then body subrhomboid	**4**
2	Epistome with dorsoposteriad directed horn and rostrum at middle with dorsal protrusion	**3**
2'	Epistome with denticle; rostrum with median and pair of submedian ridges, without dorsal protrusion between antennal insertions	***Trigonopterus palawanensis* Riedel, sp. n.**
3 (2)	Length 1.52–1.79 mm. Elytral intervals narrow, weakly costate	***Trigonopterus wallacei* Riedel, sp. n.**
3'	Length 2.32–2.45 mm. Elytral intervals subcarinate	***Trigonopterus schulzi* Riedel, sp. n.**
4 (1')	Elytra with humeri swollen, laterally markedly projecting	**5**
4'	Elytra with humeri simple, not markedly projecting	**6**
5 (4)	Pronotum anteriorly simple. Elytral striae deeply impressed; intervals costate. Anteroventral ridge of meso- and metafemur simple	***Trigonopterus trigonopterus* Riedel, sp. n.**
5'	Pronotum anteriorly with flanges projecting laterally. Elytral striae marked by small punctures; intervals flat. Anteroventral ridge of meso- and metafemur denticulate	***Trigonopterus sebelas* Riedel, sp. n.**
6 (4')	Profemur in basal half posteroventrally with slightly bifid tooth. Male metatibia ventrally with dense fringe of long, stiff setae	***Trigonopterus santubongensis* Riedel, sp. n.**
6'	Profemur in basal half posteroventrally without tooth. Male metatibia with sparse rows of setae	**7**
7 (6')	Elytral apex with suture distinctly incised. Abdominal ventrite 2 in profile simple.	
7'	Elytral apex with suture simple, not incised. Abdominal ventrite 2 in profile projecting dentiform	**9**
8 (7)	Penis as in Fig. 16b; endophallus containing numerous coarse denticles, apically with pair of relatively small sclerites	***Trigonopterus bornensis* Riedel, sp. n.**
8'	Penis as in Fig. 41b; endophallus with complex structures, several sclerites, containing few denticles	***Trigonopterus kalimantanensis* Riedel, sp. n.**
9 (7')	In front of forehead median ridge of rostrum swollen, projecting subangularly from profile	**10**
9'	Base of rostrum in profile simple, evenly confluent with forehead	
10 (9)	Penis as in Fig. 14b, near middle with shallow constriction; transfer apparatus complex, wider than long	***Trigonopterus bawangensis* Riedel, sp. n.**
10'	Penis as in Fig. 82b, near middle widened; transfer apparatus complex, small	***Trigonopterus singkawangensis* Riedel, sp. n.**
11 (9')	Color largely black. Elytral intervals flat. Penis as in Fig. 77b, apex rounded; body without distinct sclerites; transfer apparatus small, complex, wider than long	***Trigonopterus sepuluh* Riedel, sp. n.**
11	Color largely ferruginous. Elytral intervals weakly costate. Penis as in Fig. 11b, with pair of large orificial sclerites, slightly more basad with pair of smaller, darker sclerites; apex with distinct median incision	***Trigonopterus attenboroughi* Riedel, sp. n.**

### Catalogue of species groups of *Trigonopterus* Fauvel in Sundaland and the Lesser Sunda Islands

**subgenus *Mimidotasia* Voss:***Trigonopterus costipennis* Riedel, sp. n., *Trigonopterus diengensis* Riedel, sp. n., *Trigonopterus echinatus* Riedel, sp. n., *Trigonopterus foveatus* Riedel, sp. n., *Trigonopterus honjensis* Riedel, sp. n., *Trigonopterus misellus* Riedel, sp. n., *Trigonopterus satu* Riedel, sp. n., *Trigonopterus seriatus* Riedel, sp. n.

***Trigonopterus attenboroughi*-group:***Trigonopterus attenboroughi* Riedel, sp. n., *Trigonopterus bawangensis* Riedel, sp. n., *Trigonopterus santubongensis* Riedel, sp. n., *Trigonopterus sebelas* Riedel, sp. n., *Trigonopterus sepuluh* Riedel, sp. n., *Trigonopterus singkawangensis* Riedel, sp. n.

***Trigonopterus bornensis*-group:***Trigonopterus bornensis* Riedel, sp. n., *Trigonopterus kalimantanensis* Riedel, sp. n.

***Trigonopterus dimorphus*-group:***Trigonopterus acuminatus* Riedel, sp. n., *Trigonopterus alaspurwensis* Riedel, sp. n., *Trigonopterus allopatricus* Riedel, sp. n., *Trigonopterus amphoralis* Marshall, *Trigonopterus angulicollis* Riedel, sp. n., *Trigonopterus argopurensis* Riedel, sp. n., *Trigonopterus arjunensis* Riedel, sp. n., *Trigonopterus asper* Riedel, sp. n., *Trigonopterus binodulus* Riedel, sp. n., *Trigonopterus cahyoi* Riedel, sp. n., *Trigonopterus cuprescens* Riedel, sp. n., *Trigonopterus cupreus* Riedel, sp. n., *Trigonopterus dacrycarpi* Riedel, sp. n., *Trigonopterus dentipes* Riedel, sp. n., *Trigonopterus dimorphus* Riedel, sp. n., *Trigonopterus duabelas* Riedel, sp. n., *Trigonopterus fulgidus* Riedel, sp. n., *Trigonopterus gedensis* Riedel, sp. n., *Trigonopterus halimunensis* Riedel, sp. n., *Trigonopterus ijensis* Riedel, sp. n., *Trigonopterus javensis* Riedel, sp. n., *Trigonopterus lampungensis* Riedel, sp. n., *Trigonopterus latipes* Riedel, sp. n., *Trigonopterus merubetirensis* Riedel, sp. n., *Trigonopterus micans* Riedel, sp. n., *Trigonopterus pangandaranensis* Riedel, sp. n., *Trigonopterus payungensis* Riedel, sp. n., *Trigonopterus porcatus* Riedel, sp. n., *Trigonopterus rugosostriatus* Riedel, sp. n., *Trigonopterus sasak* Riedel, sp. n., *Trigonopterus serratifemur* Riedel, sp. n., *Trigonopterus setifer* Riedel, sp. n., *Trigonopterus silvestris* Riedel, sp. n., *Trigonopterus singularis* Riedel, sp. n., *Trigonopterus sinuatus* Riedel, sp. n., *Trigonopterus squalidus* Riedel, sp. n., *Trigonopterus sumatrensis* Riedel, sp. n., *Trigonopterus sundaicus* Riedel, sp. n., *Trigonopterus suturalis* Riedel, sp. n., *Trigonopterus syarbis* Riedel, sp. n., *Trigonopterus ujungkulonensis* Riedel, sp. n., *Trigonopterus variolosus* Riedel, sp. n., *Trigonopterus vulcanicus* Riedel, sp. n.

***Trigonopterus politus*-group:***Trigonopterus allotopus* Riedel, sp. n.

***Trigonopterus relictus*-group:***Trigonopterus baliensis* Riedel, sp. n., *Trigonopterus florensis* Riedel, sp. n., *Trigonopterus lombokensis* Riedel, sp. n., *Trigonopterus paraflorensis* Riedel, sp. n., *Trigonopterus parasumbawensis* Riedel, sp. n., *Trigonopterus pauxillus* Riedel, sp. n., *Trigonopterus pseudoflorensis* Riedel, sp. n., *Trigonopterus pseudosumbawensis* Riedel, sp. n., *Trigonopterus relictus* Riedel, sp. n., *Trigonopterus roensis* Riedel, sp. n., *Trigonopterus sumbawensis* Riedel, sp. n.

***Trigonopterus saltator*-group:***Trigonopterus aeneomicans* Riedel, sp. n., *Trigonopterus batukarensis* Riedel, sp. n., *Trigonopterus delapan* Riedel, sp. n., *Trigonopterus disruptus* Riedel, sp. n., *Trigonopterus dua* Riedel, sp. n., *Trigonopterus empat* Riedel, sp. n., *Trigonopterus enam* Riedel, sp. n., *Trigonopterus fissitarsis* Riedel, sp. n., *Trigonopterus kintamanensis* Riedel, sp. n., *Trigonopterus klatakanensis* Riedel, sp. n., *Trigonopterus lima* Riedel, sp. n., *Trigonopterus mesehensis* Riedel, sp. n., *Trigonopterus pararugosus* Riedel, sp. n., *Trigonopterus punctatoseriatus* Riedel, sp. n., *Trigonopterus ranakensis* Riedel, sp. n., *Trigonopterus rinjaniensis* Riedel, sp. n., *Trigonopterus rugosus* Riedel, sp. n., *Trigonopterus rutengensis* Riedel, sp. n., *Trigonopterus saltator* Riedel, sp. n., *Trigonopterus sembilan* Riedel, sp. n., *Trigonopterus telagensis* Riedel, sp. n., *Trigonopterus tepalensis* Riedel, sp. n., *Trigonopterus tiga* Riedel, sp. n., *Trigonopterus tujuh* Riedel, sp. n.

***Trigonopterus trigonopterus*-group:***Trigonopterus trigonopterus* Riedel, sp. n.

***Trigonopterus wallacei*-group:***Trigonopterus palawanensis* Riedel, sp. n., *Trigonopterus schulzi* Riedel, sp. n., *Trigonopterus wallacei* Riedel, sp. n.

## Plates

## Discussion

Herein, we apply our fast track taxonomy approach (Riedel et al. 2013a) to revise the entire *Trigonopterus* fauna of a defined region. The proportion of closely related species to be separated was higher than in our earlier work covering representatives of different species groups (Riedel et al. 2013b), but this did not cause any complications. Diagnosis of species using *cox1* sequences was straightforward and the question where to draw the line between allopatric populations and putative species entities usually could be answered by the evaluation of morphological, especially genital characters. In a few cases, especially in the *Trigonopterus relictus*-group, sibling species could be revealed that occur syntopically. After recognizing a subdivision into well separated clades of relatively high divergence we reassessed genital characters. We found that the length of the flagellum was correlated with these clusters. It can only be speculated that their discovery would not have been possible with morphological taxonomy alone as the taxonomist in charge had initially relegated these differences to intraspecific variation by lumping a number of them.

At the start of this project and based on literature the high diversity of *Trigonopterus* in the Papuan region (Riedel et al. 2010; Tänzler et al. 2012) seemed to contrast with a relatively poor representation in the Philippines (three described species: Heller 1915; Hustache 1925) and Sulawesi (one described species: Riedel 2011), while its presence in Sundaland appeared questionable – the single species described by Marshall (1925) from East Sumatra looked out of place and more like the result of a mistake than representing a reliable record. Step by step, extensive field work revealed this picture to be a simple artifact. In fact, the islands of the Sunda Arc and Borneo each harbour a rich and so far overlooked fauna, at least in places with wet primary forest. However, unlike in New Guinea and Wallacea where many species can be collected from foliage, the fauna of the Sunda Islands is largely restricted to the litter layer more difficult to sample and thus largely neglected by collectors. Moreover, *Trigonopterus* weevils usually fall into the category of “small beetles” which have a higher chance to be undescribed than beetles of larger body size (Stork et al. 2008).

The field work for this study focused on eastern parts of Sumatra, Java and the Lesser Sunda Islands. Materials stored in museum collections neither contained *Trigonopterus* from the western parts of Sumatra, nor from the Malay Peninsula. A dedicated search in the field could possibly lead to interesting discoveries, but equally well the genus´ real area of distribution excludes this region or parts of it. More promising for future discoveries of species are Borneo and Palawan where only material from a few localities was available. Borneo obviously possesses a rich fauna of *Trigonopterus* and its species number will be magnitudes higher than the ten species recorded so far. Species discovery on Java (35 species recorded), Bali (8 species recorded), and Lombok (7 species recorded) may approach saturation, while additional species are likely to be found on Sumatra (7 species recorded), Sumbawa (12 species recorded), and Flores (18 species recorded).

Most localities sampled by us harbour at least one species of *Trigonopterus*; in some places many more were found, such as seven species each at Mount Sawal of West Java, and Mount Ranaka of Flores Island. Although these figures are dwarfed by comparable localities of New Guinea (Riedel et al. 2010), the cumulative number of highly endemic species found on the Sunda Islands is substantial. A few of the species found in the montane forests of West Java are of relatively wide distribution, (e.g. *Trigonopterus allopatricus* Riedel, sp. n., *Trigonopterus javensis* Riedel, sp. n., *Trigonopterus vulcanicus* Riedel, sp. n.), but none cover the entire island. Species with a narrower distribution range can be found on somewhat isolated mountain blocks with suitable vegetation, e.g. *Trigonopterus dimorphus* Riedel, sp. n. and *Trigonopterus halimunensis* Riedel, sp. n. on Mt. Halimun-Salak, *Trigonopterus angulicollis* Riedel, sp. n. and *Trigonopterus gedensis* Riedel, sp. n. on Mt. Gede. For lowland habitats the original degree of endemism is harder to judge since the areas of lowland primary forest remaining today are very restricted themselves. The two species recorded for Ujung Kulon National Park are found nowhere else but in this last major area of primary lowland forest of western Java. Similarly, the species found in Alas Purwo National Park and the two species found in Meru Betiri National Park are also restricted to their respective localities, although the two are separated by less than 60 km distance. Such a distance could easily straddle geological, respectively ecological boundaries effectively separating populations of flightless beetles. At the same time, it raises the question how many species once inhabiting the other areas of Java´s lowland forest have become extinct when the land was converted to agricultural use.

Wide areas of East Java and the Lesser Sunda Islands are markedly influenced by seasonal climate resulting in extensive monsoon forests, while wet evergreen forests persist on the slopes of higher mountains (Whitten et al. 1996). *Trigonopterus* species are restricted to the latter areas, and the monsoon forests are a habitat unsuitable to them, just like the vegetation altered by human influence. Even before the arrival of humans on the Sunda Islands the habitats of *Trigonopterus* were probably somewhat patchy for climatic reasons. With agriculture changing the land and human population increasing, areas of wet evergreen forests became increasingly fragmented and at times limited to a few square kilometers or less. Besides the usual risks that come with small areas of habitat for animals, additional factors pose serious hazards to the species inhabiting these forests: 1) Many of the mountains supporting these forests are active or dormant volcanoes. Eruptions may destroy major portions of the habitat. During our sampling campaign violent eruptions of Mount Merapi destroyed the last remaining area of primary forest on this mountain. 2) With increasing accessibility spots of natural beauty such as pockets of wet evergreen forests become more vulnerable to illegal logging and to disasters such as wildland fires. During times of drought a cigarette or a campfire may be enough to wipe out the remaining habitat. A wide and sealed road built for the purpose of recreation gave us easy access to a small fragment of wet montane forest on slope of Mount Wilis. It can only be hoped that the road´s impact will not be fatal to *Trigonopterus acuminatus* Riedel, sp. n., a species exclusive to this area. 3) Many of the remaining wet evergreen forests stand on fertile grounds highly suitable to cultivate vegetables. With some of the highest population densities worldwide, it is not surprising that in Java, Bali and Lombok gardens tend to expand on the mountain slopes and encroach even on protected forests. Many *Trigonopterus* species have constraints in terms of elevation: For example, *Trigonopterus argopurensis* Riedel, sp. n. was found to a maximum elevation of 1785 m. Although a larger forest area exists on the upper slopes of Mt. Argopuro, it seems to be unsuitable to this particular species which is cornered by coffee gardens in its lower range.

Looking at the distribution of *Trigonopterus* species in the Sunda Arc and the areas of remaining primary forests, it appears likely that some of these newly discovered species will become extinct in the next few decades. The blame will be largely on human activities which have reduced the once extensive areas of forest to small fragments. It is our responsibility to help preserving these remaining areas of habitat. We should intensify our efforts especially with those forests harbouring endemic species but nevertheless holding a relatively weak conservation status, as exemplified by the reserves of Mount Sawal and Mount Ranaka. In these cases, a limited investment in better protection could achieve a significant improvement for species conservation.

Herein, we provide faces to 98 hitherto unknown species by publishing their descriptions and images, in parallel also on the wiki-site Species-ID. The new names will be useful to deposit scientific data reliably and sustainably. Separate studies on the phylogeny and distribution patterns of *Trigonopterus* are in progress and will build on this taxonomic foundation. Most of all, it is hoped that knowledge of this previously unknown fauna will create an awareness for the conservation value of the remaining fragments of wet evergreen primary forest in the region. There may be a certain redundancy of conservation areas when we look at the distribution of many vertebrates – however, the larger portion of biodiversity as represented by arthropods exhibits a different picture. The message that a study of flightless weevils of the Sunda Arc conveys is clear: with almost every area of remaining primary forest being destroyed we will lose some more species – with or without names.

## Supplementary Material

XML Treatment for Trigonopterus

XML Treatment for Trigonopterus acuminatus

XML Treatment for Trigonopterus aeneomicans

XML Treatment for Trigonopterus alaspurwensis

XML Treatment for Trigonopterus allopatricus

XML Treatment for Trigonopterus allotopus

XML Treatment for Trigonopterus angulicollis

XML Treatment for Trigonopterus amphoralis

XML Treatment for Trigonopterus argopurensis

XML Treatment for Trigonopterus arjunensis

XML Treatment for Trigonopterus asper

XML Treatment for Trigonopterus attenboroughi

XML Treatment for Trigonopterus baliensis

XML Treatment for Trigonopterus batukarensis

XML Treatment for Trigonopterus bawangensis

XML Treatment for Trigonopterus binodulus

XML Treatment for Trigonopterus bornensis

XML Treatment for Trigonopterus cahyoi

XML Treatment for Trigonopterus costipennis

XML Treatment for Trigonopterus cuprescens

XML Treatment for Trigonopterus cupreus

XML Treatment for Trigonopterus dacrycarpi

XML Treatment for Trigonopterus delapan

XML Treatment for Trigonopterus dentipes

XML Treatment for Trigonopterus diengensis

XML Treatment for Trigonopterus dimorphus

XML Treatment for Trigonopterus disruptus

XML Treatment for Trigonopterus dua

XML Treatment for Trigonopterus duabelas

XML Treatment for Trigonopterus echinatus

XML Treatment for Trigonopterus empat

XML Treatment for Trigonopterus enam

XML Treatment for Trigonopterus fissitarsis

XML Treatment for Trigonopterus florensis

XML Treatment for Trigonopterus foveatus

XML Treatment for Trigonopterus fulgidus

XML Treatment for Trigonopterus gedensis

XML Treatment for Trigonopterus halimunensis

XML Treatment for Trigonopterus honjensis

XML Treatment for Trigonopterus ijensis

XML Treatment for Trigonopterus javensis

XML Treatment for Trigonopterus kalimantanensis

XML Treatment for Trigonopterus kintamanensis

XML Treatment for Trigonopterus klatakanensis

XML Treatment for Trigonopterus lampungensis

XML Treatment for Trigonopterus latipes

XML Treatment for Trigonopterus lima

XML Treatment for Trigonopterus lombokensis

XML Treatment for Trigonopterus merubetirensis

XML Treatment for Trigonopterus mesehensis

XML Treatment for Trigonopterus micans

XML Treatment for Trigonopterus misellus

XML Treatment for Trigonopterus palawanensis

XML Treatment for Trigonopterus pangandaranensis

XML Treatment for Trigonopterus paraflorensis

XML Treatment for Trigonopterus pararugosus

XML Treatment for Trigonopterus parasumbawensis

XML Treatment for Trigonopterus pauxillus

XML Treatment for Trigonopterus payungensis

XML Treatment for Trigonopterus porcatus

XML Treatment for Trigonopterus pseudoflorensis

XML Treatment for Trigonopterus pseudosumbawensis

XML Treatment for Trigonopterus punctatoseriatus

XML Treatment for Trigonopterus ranakensis

XML Treatment for Trigonopterus relictus

XML Treatment for Trigonopterus rinjaniensis

XML Treatment for Trigonopterus roensis

XML Treatment for Trigonopterus rugosostriatus

XML Treatment for Trigonopterus rugosus

XML Treatment for Trigonopterus rutengensis

XML Treatment for Trigonopterus saltator

XML Treatment for Trigonopterus santubongensis

XML Treatment for Trigonopterus sasak

XML Treatment for Trigonopterus satu

XML Treatment for Trigonopterus schulzi

XML Treatment for Trigonopterus sebelas

XML Treatment for Trigonopterus sembilan

XML Treatment for Trigonopterus sepuluh

XML Treatment for Trigonopterus seriatus

XML Treatment for Trigonopterus serratifemur

XML Treatment for Trigonopterus setifer

XML Treatment for Trigonopterus silvestris

XML Treatment for Trigonopterus singkawangensis

XML Treatment for Trigonopterus singularis

XML Treatment for Trigonopterus sinuatus

XML Treatment for Trigonopterus squalidus

XML Treatment for Trigonopterus sumatrensis

XML Treatment for Trigonopterus sumbawensis

XML Treatment for Trigonopterus sundaicus

XML Treatment for Trigonopterus suturalis

XML Treatment for Trigonopterus syarbis

XML Treatment for Trigonopterus telagensis

XML Treatment for Trigonopterus tepalensis

XML Treatment for Trigonopterus tiga

XML Treatment for Trigonopterus trigonopterus

XML Treatment for Trigonopterus tujuh

XML Treatment for Trigonopterus ujungkulonensis

XML Treatment for Trigonopterus variolosus

XML Treatment for Trigonopterus vulcanicus

XML Treatment for Trigonopterus wallacei
